# Cooperation of transposable elements to endow global networks of initiators of hybrid assembly pathways of endogenous multiprotein complexes

**DOI:** 10.3389/fcimb.2026.1793868

**Published:** 2026-07-30

**Authors:** Gennadi V. Glinsky

**Affiliations:** Institute of Engineering in Medicine, University of California San Diego, La Jolla, CA, United States

**Keywords:** protein-protein interactions (PPI), transcription factor binding sites (TFBS), pioneer transcription factors; transposable elements (TE), rapidly evolving conserved elements (RECE), human ancestral quickly evolving regions (HAQER), human accelerated regions (HAR), human-specific brain development regulatory regions (BDRR)

## Abstract

Mechanisms governing initiation steps of the assembly of endogenous multi-protein complexes (EMC) remain incompletely understood. Here, multiple lines of observations are reported describing the function-aligned initiation sequence of hybrid assembly pathways (HAP) of EMC. The first step of HAP-guided chain reactions of protein-protein interactions (PPI) of EMC assemblies constitutes the creation of cell type-specific pools of hetero and homo dimers. The molecular anatomy of HAP was elucidated by defining qualitative and quantitative characteristics of protein binding to a compendium of 200,393 distinct genomic regulatory elements (GRE), including 49,667 sequences representing control sets of genomic loci as well as 150,726 GRE of different evolutionary origins. The consensus sequence of HAP actions consists of: a) Initiation on genomic DNA of the formation of metastable hetero- and homodimers of EMCs’ protein constituents; b) Release of dimers from DNA templates for delivery to the EMC assembly compartments; c) Assembly of defined EMC by sequential on demand addition of proteins to preformed dimers serving as attractors of EMC-specific ensembles of monomers. Chromosome-naïve DNA scaffolds facilitating creation of intracellular dimer pools engage networks of ~700 transcription factors (TFs), 534 of which manifest region-specific patterns of significantly enriched expression in 1358 brain regions. HAP initiators appear to operate within nucleosome-depleted islands of transposable elements (TE) – derived sequences within heterochromatin. PPI assembly lines of EMCs operate in 2 concurrent modes: TF-TF PPI cascade and PPI HUB protein cascade. Regardless of the number of DNA-bound initiator TFs (ranging from one to 716 TFs), both modes of operations reached the equilibrium at the PPI constituents saturation levels of ~245 proteins for TF-TF PPI modes and of ~351 proteins for PPI HUB protein modes. Distinct panels of DNA-bound initiator TFs and proteins of PPI cascade ensembles are enriched in either defined sets of neuroanatomical structures (TF-TF mode) or among structural-functional constituents of synapses (HUB proteins mode). Thus, these bifurcated cascades appear biologically congruent: TF–TF constituents map to transcriptional signatures of hundreds of brain regions, whereas HUB constituents map to synaptogenesis and synaptic structures, suggesting the unified logic of genomic functions coordinating region identity and connectivity. Evidence-supported examples of default operations of PPI-guided assemblies of hetero- and homodimers of Yamanaka factors, neurogenesis constituents, and protein components of postsynaptic density of excitatory and inhibitory synaptogenesis are reported with detailed analytical focus on human Claustrum. The foundational set of observations reported in this contribution should facilitate experimental and theoretical explorations of TE-seeded genomic codes for initiators of PPI chain reactions of protein dimerization creating pools of attractors to guide and accelerate the EMC assemblies.

## Graphical Abstract

**Figure:**
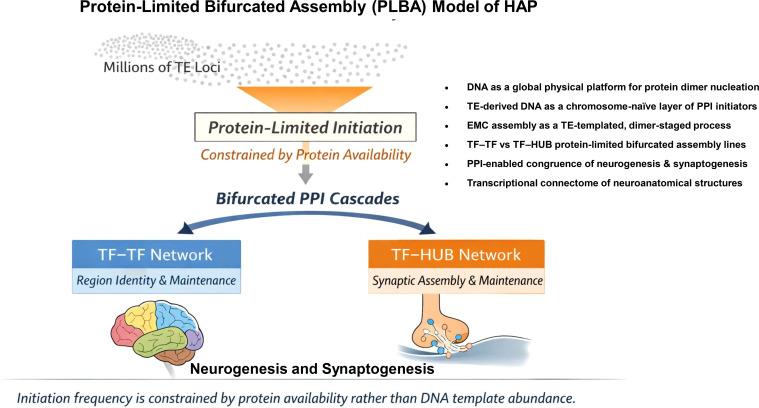

## Introduction

Multiprotein complexes are essential building blocks of life, from viruses to humans. They ensure the faithful structural–functional harmony of enzymes, phase-separated condensates, sub-cellular organelles, cells, tissues, organs, and entire organisms. Remarkable progress has been achieved in identifying and characterizing thousands of endogenous multiprotein complexes (EMCs) in hundreds of species, including humans (Malovannaya et al., 2011; Marsh et al., 2013; Wiederstein et al., 2014; Zaret, 2020; Sinha et al., 2023; Pownall et al., 2023). However, it remains unknown whether common mechanisms of EMC biogenesis exist that guide the initiation of the multistep molecular assembly of highly diverse arrays of EMC components. Therefore, understanding the fundamental principles governing the dynamics of EMC assembly remains one of the most significant challenges in contemporary biological and biomedical sciences.

DNA sequences derived from transposable elements (TEs) constitute approximately 50% of the human genome, contributing to a multitude of structural features and regulatory functions at different levels of genomic organization (Lu et al., 2014; Elbarbary et al., 2016; Sundaram and Wysocka, 2020; Hoyt et al., 2022; Rosspopoff et al., 2025). During evolution, concurrently with TE colonization of primate genomes, a highly sophisticated defense system co-evolved. This system was designed to restrict uncontrolled TE expansion and diminish potentially deleterious effects of TE insertions on genome integrity, while also providing TE family sequence-specific co-option mechanisms that integrate TE-derived sequences into cell type- and tissue-specific genomic regulatory networks (Jacobs et al., 2014; Pontis et al., 2019; Rosspopoff et al., 2025). While the potentially destructive effects of TEs on genome integrity represent their ubiquitous biological feature, defined by the very nature of transpositionally competent TEs, it remains less clear whether TE-derived DNA sequences may possess similarly ubiquitous non-deleterious functions, perhaps contributing to the evolutionary fitness of the host.

TE-derived sequences are often integrated into species’ genomic regulatory networks. In mammalian genomes, TE-derived sequences have rewired the core regulatory circuitry of embryonic stem cells (Kunarso et al., 2010). They are intrinsically activated in human preimplantation embryos (Grow et al., 2015) by operating as long-range enhancers (Fuentes et al., 2018) and providing transcription factor binding sites (TFBS) for TP53 (Wang et al., 2007), STAT1 (Schmid and Bucher, 2010), and CTCF (Schmidt et al., 2012). This includes a set of transcription factors (TFs) defined as master pluripotency regulators (Kunarso et al., 2010; Schmidt et al., 2012), with candidate human-specific TFBS for POU5F1 (OCT4), NANOG, SOX2, and CTCF (Glinsky, 2015, 2017; Glinsky et al., 2018). Overall, chromatin immunoprecipitation and sequencing (ChIP-seq) experiments have demonstrated that TE-derived loci harbor thousands of TFBS, presumably exerting global regulatory effects on gene expression in various pathophysiological conditions. However, the distribution of TFBS is non-uniform and highly variable within specific TE subfamilies that evolutionarily emerged from identical or highly similar sequences. TE sequence-dependent distribution of TFBS is likely influenced by genetic drift and other mutational processes, as well as cell type-specific epigenetic contexts affecting chromatin states of genomic regions harboring TE insertions.

Based on these considerations, it was reasonable to assume that ChIP-seq experiments could capture only a fraction of the TFBS repertoire encoded by ancestral TE loci that may have existed at the time of TE insertion and expansion in a host genome. Therefore, cataloging all TFs with potential TFBS located within genomic loci encoded by a specific TE subfamily was of interest. This would facilitate comparative analyses of TFBS encoded by distinct TE families to infer their concordant, non-overlapping, and discordant regulatory functions (Glinsky, 2022; 2025; 2026). This approach facilitated the identification and characterization of genome-wide regulatory networks encompassing ubiquitous, compositionally nearly identical, yet quantitatively distinct panels of DNA sequences of transcription factor binding sites (TFBS) for 716 proteins encoded by DNA molecules derived from diverse TE families, including LT5_Hs/HERVK; LTR7/HERVH; MLT2A1; MLT2A2; SVA; LINE; and SINE/Alu. The evolutionarily conserved foundation of these genomic regulatory networks (GRNs) appears assembled from arrays of approximately 770 TFBS. A majority of protein constituents of these GRNs (700 of 716; 98%) are defined by gene ontology (GO) features of sequence-specific double-stranded DNA binding (GO:1990837). Since TE-derived sequences constitute approximately 50% of human genomic DNA, these observations indicate that large fractions of genomic DNA sequences may share common regulatory features encoded by common sets of TFBS (Glinsky, 2022; 2025; 2026).

Genomic DNA is known to serve as a scaffold for the initiation and assembly of a wide variety of endogenous multiprotein complexes. This scaffolding function is particularly prominent in processes that directly involve the genome, such as DNA replication, transcription, and DNA repair. Specific sequences and structures of DNA molecules act as binding sites that orchestrate the recruitment and assembly of the complex protein machinery necessary for cell survival and function. The primary mechanism by which genomic DNA acts as a scaffold is by providing sequence-specific binding sites for initiator proteins. These initiator proteins are usually represented by a protein capable of binding DNA molecules in a sequence-specific manner, such as transcription factors, replication factors, and chromatin remodelers. The initiator proteins recognize and bind to unique DNA sequences, serving as the first step in initiating a cascade of protein-protein interactions (PPI).

At the DNA and chromatin levels, this initial binding event creates a localized platform that facilitates the subsequent, high-affinity binding of other proteins, ultimately leading to the formation of a fully functional complex. There are several well-known examples of DNA-templated complex assembly, with perhaps the most well-established being the assembly of the pre-replication complex (pre-RC). In eukaryotes, the binding of the origin recognition complex (ORC) to specific DNA sequences at replication origins is essential for recruiting other key proteins like Cdc6 and Cdt1, which in turn facilitate the loading of the Mcm2–7 complex. This ordered, hierarchical assembly cannot proceed flawlessly without the DNA scaffold; its absence causes a failure in DNA replication (Bell and Dutta, 2002; Waga and Stillman, 1998).

The initiation of gene transcription is another fundamental process dependent on DNA functioning as a scaffold. At the initiation step, general transcription factors (GTFs) and RNA polymerase II are recruited to gene promoters. The assembly process is often initiated by the TATA-binding protein (TBP), which binds to a specific DNA sequence, known as the TATA box, found in many promoters. TBP’s binding then acts as a nucleation point for the assembly of the complete preinitiation complex (PIC) (Buratowski et al., 1989). The regulatory impacts of chromatin information content on interactions of DNA with transcription factors are currently being investigated (D'Oliveira Albanus et al., 2021).

The dynamic regulatory role of the DNA scaffold is highlighted by topological changes in the DNA, such as supercoiling and chromatin relaxation, which influence the assembly of multiprotein complexes (Tian et al., 2025). The integrity of genomic DNA molecules is crucially dependent on a DNA scaffold function. When DNA is damaged, complexes of DNA repair proteins must quickly assemble at the site of the lesion. Notably, the site of the damaged DNA itself serves as the scaffold for this process. In base excision repair (BER), proteins like PARP1 and PARP2 recognize DNA damage and bind to the site, recruiting other BER enzymes to form a functional repair complex (Roedgaard et al., 2015; Vasil'eva et al., 2021; Tian et al., 2025).

Despite foundational knowledge unequivocally illustrating the crucial role of DNA molecules as scaffolds for initiating the assembly of endogenous multiprotein complexes (EMCs), it remains unclear to what extent the scaffolding functions of genomic DNA are relevant to EMC assembly not directly linked to DNA’s molecular functions. Specifically, it is unknown whether DNA sequences derived from TEs function as global, genome-wide scaffolds for initiating protein–protein interactions that lead to EMC assembly.

Collectively, observations in this contribution highlight a previously underappreciated function of TE-derived DNA sequences: providing PPI seed templates to initiate the assembly of protein dimer constituents for thousands of EMCs. These EMCs may play essential roles in the development, physiological processes, and pathological conditions of Modern Humans. Multiple lines of analytical evidence implicate approximately 4.5 million TE-encoded DNA loci as a massively parallel, protein-limited initiation layer for hybrid assembly pathways that continually nucleate PPI cascades in real time. These cascades congruently bifurcate into TF-TF networks, enriched in transcriptional programs defining neuroanatomical brain regions, and TF-HUB protein networks, enriched in constituents of synaptogenesis and synaptic architecture. This provides a unified genomic framework for the coordinated development and maintenance of brain regions and their connectivity.

Reported findings support a protein-limited bifurcated assembly (PLBA) HAP model, in which millions of TE-encoded DNA templates provide a non-limiting initiation layer, while protein availability constrains the real-time bifurcation of PPI cascades. These cascades split into TF-TF pathways associated with the molecular definition of brain-region identity and TF-HUB pathways facilitating synaptic assembly and neuronal connectivity.

## Results

### Patterns and consensus default features of PPI chain reactions originated at TFBS encoded by TE-derived sequences

DNA sequences encoding TFBS actively influence the PPIs of TFs and other DNA-binding proteins in several ways: (i) the number of DNA sequence elements a given TF can specifically recognize can be increased by the formation of cooperative TF-TF-DNA complexes; (ii) TFs can bind to DNA as pre-formed multimeric protein complexes, typically dimers or trimers; and (iii) DNA sequences guide and facilitate specific TF–TF interactions (Reinke et al., 2013; Jolma et al., 2015; Wolberger, 2021; Bonchuk et al., 2023; Kim et al., 2024; Xie et al., 2025; Naqvi et al., 2025). Therefore, it was of interest to examine the features of PPIs for the 716 TFs analyzed herein that have the capacity to bind to TE-seeded TFBS scattered across the human genome.

**Figure 1:**
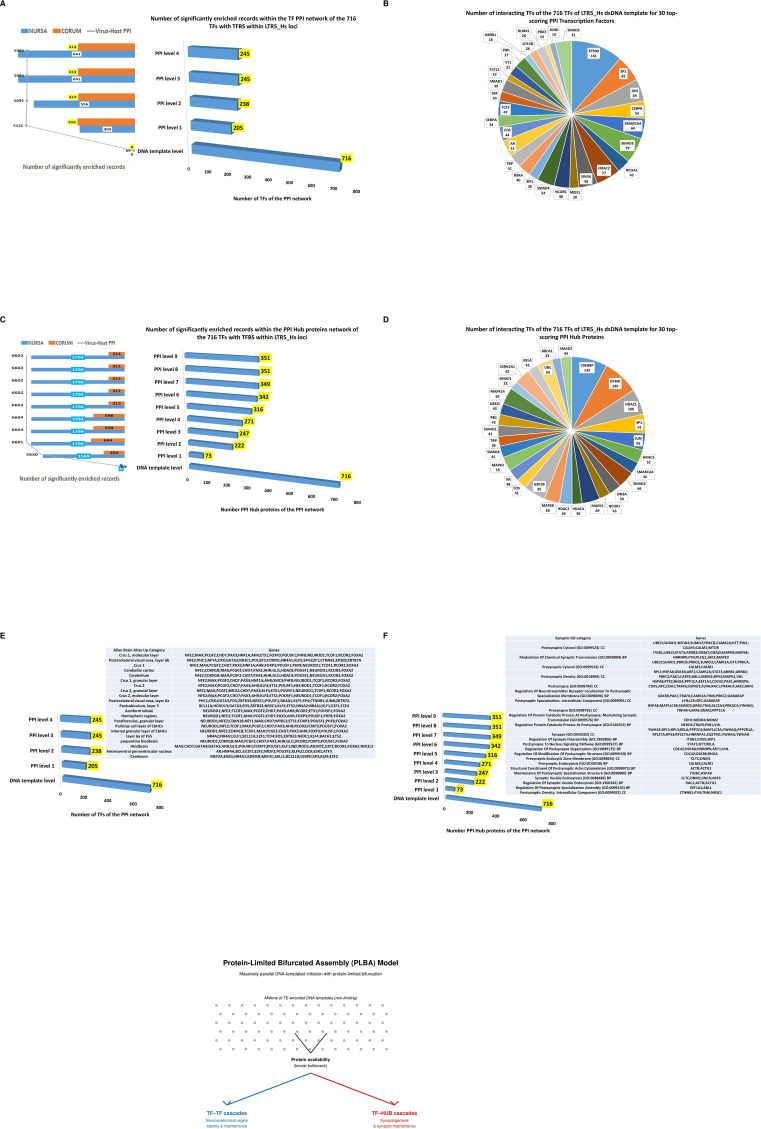
Genome-wide properties of DNA-templated protein–protein interaction (PPI) cascades and the protein-limited bifurcated assembly (PLBA) model. **(A–D)** Genome-wide analyses of DNA-templated PPI cascades initiated at transposable element (TE)–encoded loci reveal reproducible saturation behavior and distinct classes of interacting protein assemblies. **(E)** Constituents of TF–TF PPI cascades are significantly enriched among genes highly expressed across hundreds of neuroanatomically defined brain regions, consistent with roles in regional transcriptional identity. **(F)** Constituents of TF-HUB PPI cascades are significantly enriched among proteins involved in synaptogenesis and mature synaptic structure and function. **(G)** Synthesis of these findings into the PLBA model, in which millions of TE-encoded DNA templates provide a non-limiting initiation layer, while protein availability constrains the real-time bifurcation of PPI cascades into TF–TF pathways supporting brain-region identity and TF-HUB pathways supporting synaptic assembly and maintenance. A total of 716 transcription factor binding sites (TFBS) encoded by DNA sequences of different families of TEs were analyzed using gene set enrichment analyses (GSEA) applied to the database of TF–TF PPIs **(A, B, E)** or the HUB proteins’ PPI database **(C, D, F)**. GSEA identify the sets of proteins manifesting significantly enriched profiles (adjusted p-value < 0.05) of PPIs. Independent GSEA were executed sequentially with each set of significantly enriched proteins in PPIs to catalog all constituents of the PPI cascade sequence. To identify the number of EMCs manifesting significant enrichments in compositions of their protein subunits and corresponding sets of PPI constituents identified at different levels of the PPI cascade, GSEA was performed employing several databases of human endogenous complexomes (NURSA; CORUM; virus–host PPI; see the Methods section) with each set of proteins identified as significantly enriched PPI constituents of the step-wise sequence of the PPI cascade. **(B)** shows the number of interacting TFs of the panel of 716 TFs interacting with the DNA template for the 30 top-scoring PPI transcription factors (TFs) identified at the TF–TF PPI cascade level 1. **(D)** shows the number of interacting TFs of the panel of 716 TFs interacting with the DNA template for the 30 top-scoring PPI HUB proteins at the PPI cascade level 1. PPI HUB proteins are proteins that have been experimentally documented to interact via PPI with at least 50 other proteins. **(E)** reports the 21 top-scoring human brain regions and corresponding sets of significantly enriched TF-coding genes, which GSEA of the Allen human brain atlas database of upregulated genes identified by interrogating the 245 TFs engaged in the significantly enriched PPI patterns identified at the saturation levels 3 and 4 of the TF–TF PPI cascade. **(F)** reports the 23 top-scoring records of the synaptic gene ontology (GO) categories and corresponding sets of significantly enriched PPI HUB protein-coding genes, which GSEA of the synaptic GO database identified by interrogating the 351 HUB proteins engaged in the significantly enriched PPI patterns at the saturation levels 8 and 9 of the HUB protein PPI cascade.

To this end, gene set enrichment analysis (GSEA) of 716 initiator TFs was performed using a database of TF–TF protein–protein interactions (PPI) to identify all known TFs with significantly enriched propensities (adjusted p-value < 0.05) to bind to the initiator TFs via PPI initiator TF. The results were tabulated, quantified, and plotted for visualization (**Figure 1**).

Multiple TFs appear to engage in PPI with the initiator TFs, forming protein dimers (**Figure 1**; **Supplementary Data Set S1**). For instance, the top-ranked single TF constituent at PPI level 1 (EP300) could form dimers with as many as 161 initiator TFs (**Figure 1**; **Supplementary Data Set S1**). TFs identified in the first step of the PPI analytical pipeline as proteins exhibiting a significant likelihood of binding to the initiator TFs were then subjected to GSEA to identify a new set of TFs with a significantly increased propensity to engage in PPI.

Corresponding sequential steps of PPI chain reactions were designated as PPI level 1, PPI level 2, and so on (**Figure 1**). Interestingly, these analyses revealed that the TF–TF PPI cascade reached a saturation state, engaging 245 TFs at PPI level 3. The panel of TFs involved in PPIs at subsequent analytical steps remained constant (**Figure 1**). Follow-up GSEA analyses of the 245 TFs engaged in TF–TF PPIs at the saturation level identified 119 TFs whose expression is significantly enriched in 233 human brain regions within the Allen Brain Atlas database of upregulated genes (**Supplementary Data Set S1**).

Notably, the top-ranked significantly enriched record identified by GSEA of the 245 or 119 saturation-level TFs, utilizing the WikiPathways 2024 Human database, is the sudden infant death syndrome (SIDS) susceptibility pathways WP706 (**Supplementary Data Set S1**).

One of the most notable features of TFs engaging in PPIs at various levels of the PPI cascade chain reactions is the significant enrichment among protein constituents of endogenous multiprotein complexes (EMPs). This was identified by GSEA of several EMC databases: the NURSA human endogenous complexome database, the CORUM database, and the virus–host PPI P-HIPSTer 2020 database (**Figure 1**; **Supplementary Data Set S1**).

Significantly, GSEA of the identified TF panels revealed that the enrichment patterns appear to reach a plateau of consistently maintained high levels of significantly enriched records for EMC constituents at PPI levels 2-3. These comprise 313, 641, and 5,084 EMCs recorded in the CORUM, NURSA, and virus–host PPIs databases, respectively (**Figure 1**; **Supplementary Data Set S1**). These findings align with the hypothesis that PPI chain reactions originating at the initiator TFs could create protein dimers composed of EMC constituents.

Next, GSEA on 716 initiator TFs was carried out, employing the TF-HUB proteins PPI database to identify all known HUB proteins that exhibit significantly enriched predilections (adjusted p-value < 0.05) to bind the initiator TFs via PPI initiator TF. Analyses of the HUB proteins’ PPI database are of particular interest in this study because HUB proteins are defined as a category of proteins known to form physical complexes via PPIs with at least 50 other proteins. Therefore, HUB proteins likely contribute to the assembly of many EMCs. Results of the TF-HUB proteins PPI analyses were tabulated, quantified, and plotted for visualization (**Figure 1**; **Supplementary Data Set S1**). Similar to TFs, multiple HUB proteins appear to engage in PPI with the initiator TFs to form protein dimers (**Figure 1**; **Supplementary Data Set S1**). The top-ranked HUB protein constituents at PPI level 1, namely, CREBBP, EP300, and HDAC1, could form dimers with as many as 139, 140, and 100 initiator TFs, respectively (**Figure 1**; **Supplementary Data Set S1**). HUB proteins identified in the first step of the PPI analytical pipeline as proteins manifesting a significant likelihood of binding to the initiator TFs were then subjected to GSEA to identify a new set of HUB proteins with significantly increased ability to engage in PPIs. Corresponding sequential steps of TF-HUB protein PPI chain reactions were analyzed at PPI level 1, PPI level 2, etc., and the results of these analyses were plotted for visualization (**Figure 1**).

GSEA revealed that the PPI cascade of HUB proteins approached saturation at levels 6 and 7 (342 and 349 HUB proteins, respectively), reaching full saturation at PPI level 8 with 351 HUB proteins (**Figure 1**; **Supplementary Data Set S1**). This panel of 351 HUB proteins remained constant for subsequent PPI analyses, continuously recycling the same set (**Figure 1**; **Supplementary Data Set S1**).

Follow-up GSEA analyses of these 351 HUB proteins at the saturation level showed no significant enrichment in upregulated genes within human brain regions according to the Allen Brain Atlas database (**Supplementary Data Set S1**). However, GSEA utilizing the synaptic gene ontology (GO) database (SynGO 2024) identified significant enrichment of subsets of these 351 saturation-level PPI HUB proteins in 23 GO categories defined by SynGO 2024 (**Figure 1**; **Supplementary Data Set S1**). Notably, a majority (14 of 23; 61%) of these significantly enriched SynGO 2024 records describe structural/functional protein components of post synaptic density (PSD) complexes and pathways crucial for synaptogenesis and the structural–functional integrity of synapses (**Figure 1**; **Supplementary Data Set S1**).

GSEA of HUB proteins involved in the PPI cascade also revealed apparent enrichments in numerous vital multiprotein complexes, networks, and signaling pathways relevant to human health and disease. Examples include “Embryonic Lethality During Organogenesis, Complete Penetrance MP:0011098” (MGI Mammalian Phenotype Level 4–2024 database) and “Brain Derived Neurotrophic Factor BDNF Signaling WP2380” (WikiPathways 2024 Human database) (Supplementary Data Set S1).

HUB proteins exhibiting significantly enriched numbers of PPIs at various levels of the cascade also showed significant enrichment among protein constituents of endogenous multiprotein complexes (EMCs). This was identified by GSEA of several EMC databases, including the NURSA human endogenous complexome database, the CORUM database, and the virus–host PPI P-HIPSTer 2020 database (**Figure 1**; **Supplementary Data Set S1**). Significantly, GSEA of the identified HUB protein panels revealed that enrichment patterns reached a plateau of consistently high levels of significantly enriched EMC constituents at PPI levels 2-3. This encompassed 64, 1,795, and 6,604 EMCs recorded in the CORUM, NURSA, and virus–host PPIs databases, respectively (**Figure 1**; **Supplementary Data Set S1**). These findings appear to support the hypothesis that PPI chain reactions originating from initiator TFs could generate protein dimers comprising constituents of a large number of EMCs.

Observations reported in this section of the Results revealed that the sequences of the cascade of PPI chain reactions originated at the initiator TFs and progressed either along the TF–TF PPI mode or the HUB proteins PPI mode, arriving at the saturation stage of the PPI cascade. The saturation stages of the PPI cascade manifest recycling patterns of constant numbers of the PPI protein constituents, comprising 245 TFs for the TF–TF PPI progression branch or 351 HUB proteins for the HUB proteins PPI progressing branch. A PPI seed signature of TFBS for 29 genes encoding dual-function proteins acting as both TF-PPIs and PPI Hub constituents required the smallest numbers of consecutive PPI steps to achieve the saturation levels of PPI chain reactions. PPI reaction chains initiated by these 29 proteins reached saturation points of PPI assemblies in three and six consecutive PPI cycles for TF–TF PPI and PPI Hub assembly modes, respectively (**Supplementary Data Set S1**). Present analyses appear to generate novel observations in the context of the potential regulatory impacts of the PPI chain reactions originated at the initiator TFs on human brain development and functions. GSEA of the regulatory TFs and HUB proteins engaged in PPI along the sequence of the PPI cascade revealed two distinct patterns: (i) the significant enrichment of subsets of regulatory TFs among the upregulated genes in defined regions of the human brain and (ii) the significant enrichment of subsets of regulatory HUB proteins among defined sets of structural and functional proteins comprising the essential constituents of the synaptogenesis pathways and activities of mature synapses. Therefore, these observations suggest a foundational model of cooperative actions of two PPI cascade branches originated at initiator TFs, the TF–TF PPI branch and the HUB proteins PPI branch, in securing coherent regulatory frameworks for an intrinsically integrated development of both specific brain regions and essential for their functions synaptic networks. Consistent with this hypothesis, these two patterns are preserved during the analytical divergence between branches by switching the sequences of the PPI pipeline interrogation from the TF–TF PPI branch to the HUB proteins PPI branch or vice versa.

### Patterns and consensus features of PPI chain reactions originated at TFs bound to GREs created by distinct mechanisms of primate regulatory DNA evolution

It was of interest to investigate the patterns of PPI chain reactions initiated by TFs. These TFs are physically bound to DNA sequences of GREs that were created during primate evolution through distinct mechanisms and have been implicated as regulatory loci of brain development and function (Methods). This included the panel of GREs employed during the analyses of their TFBS density profiles (Glinsky, 2026). GSEA was carried out exactly as described for 716 initiator TFs by imputing TFs bound to GREs encoded by:

- Different families of human embryo regulatory LTRs (LTR5_Hs, LTR7, MLT2A1, and MLT2A2).
- A set of transpositionally active LINE1 elements in the human genome.
- Diverse panels of experimentally defined brain development GREs with different evolutionary mechanisms of origin, including human-specific brain development regulatory regions (hsBDRRs); human brain primary cell active enhancers (hBrain enhancers), human brain organoid active enhancers (hBrain organoids enhancers); the artificial intelligence (AI) agent Google Gemini-defined set of 25 master brain development regulatory TFs (AI-defined brain development TFs); highly conserved GREs with accelerated acquisition of mutations during primate evolution at the beginning of brain size expansion (rapidly evolving conserved elements, RECE); highly conserved GREs with accelerated acquisition of mutations on the human lineage (human accelerated regions, HARs; Human Ancestor Quickly Evolved Regions, HAQERs); and *de novo* created human-specific regulatory sequences (*de novo* HSRS; **Supplementary Data Set S1**).

**Figure 2:**
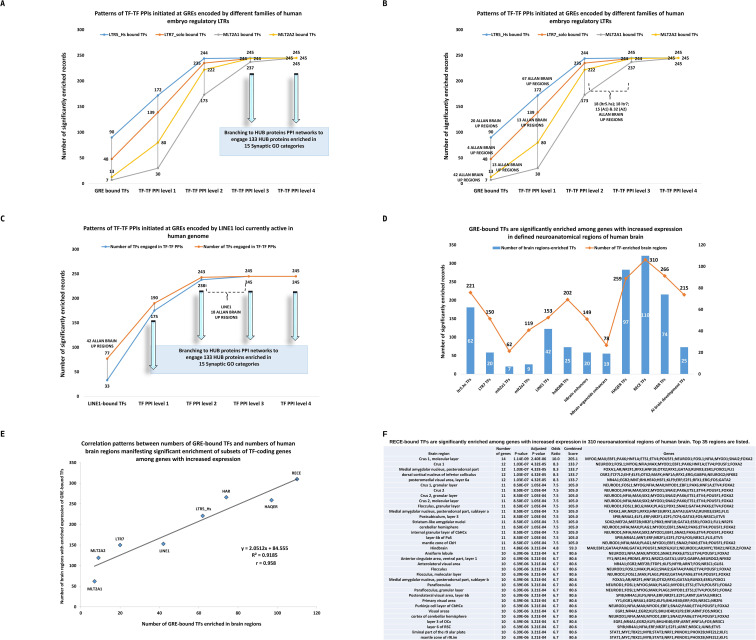
Visualization of TF–TF protein–protein interaction (PPI) networks of initiator TFs that are bound to genomic regulatory elements (GREs) encoded by different families of human embryo regulatory long terminal repeats, LTRs **(A, B)**; transpositionally active in human genome LINE1 **(C)**; and diverse panels of experimentally defined brain development GREs of different evolutionary mechanisms of origin **(D–F)**. Transcription factors (TFs) bound to specific sets of GREs were identified employing the UniBind algorithm (Methods) and analyzed using the gene set enrichment analysis (GSEA) applied to the database of TF–TF PPIs as described in the legend to the **Figure 1** and Methods. The number of proteins engaged in TF–TF PPI was defined by quantifying all proteins manifesting significantly enriched PPI profiles (adjusted p-value < 0.05) at each step of the PPI cascade sequence and plotted for visualization. Significantly enriched TFs at the indicated steps of the PPI cascade were interrogated using the GSEA applied to the Allen human brain atlas database of upregulated genes to identify human brain regions manifesting significant enrichment profiles for subsets of TFs engaged in TF–TF PPI at indicated levels of the PPI cascade **(B–F)**. **(D, E)** reports the results of these analyses for TFs bound to specific GREs at the DNA template of PPI initiators, including the following GREs: human embryo regulatory LTRs (LTR5_Hs; LTR7; MLT2A1; MLT2A2); transpositionally active in human genome LINE1; human-specific brain development regulatory regions (hsBDRRs); human brain primary cells active enhancers (hBrain enhancers); human brain organoids active enhancers (hBrain organoids enhancers); artificial intelligence agent Google Gemini-defined set of master brain development regulatory TFs (AI brain development TFs); highly conserved GREs with accelerated mutation acquisition during primate evolution (rapidly evolving conserved elements, RECE); highly conserved GREs with accelerated mutation acquisition on the human lineage (human accelerated regions, HARs; human ancestor quickly evolved regions, HAQERs). Results are plotted for visualization as the bar graphs **(D)** and the correlation plot **(E)**. Significantly enriched sets of protein identified at the saturation levels of the TF–TF PPI cascade were interrogated using the GSEA applied to the HUB proteins’ PPI database to document the “branching” effects of the PPI cascade switching from TF–TF PPIs to TF-HUB protein PPIs **(A, C)**. Follow-up GSEA of 133 HUB proteins engaged in significantly enriched PPI with TFs at the saturation level of the PPI cascade revealed significant enrichment of subsets of identified HUB proteins in 15 Synaptic GO database-defined categories **(A, C)** (**Supplementary Table 1**). **(F)** reports the 35 top-scoring human brain regions and corresponding sets of significantly enriched TF-coding genes, which were identified by GSEA of the Allen human brain atlas database of upregulated genes by interrogating the 110 TFs bound to rapidly evolving conserved elements (RECE) GREs. GSEA of 110 RECE-bound TFs employing the Allen brain atlas database of upregulated genes documented the significant enrichment of subsets of regulatory TFs in 310 neuroanatomical structures of human brain (**Supplementary Table 1**).

**Figure 2** presents visualization examples of TF–TF PPI networks. These networks originate from initiator TFs bound to GREs, which are encoded by various families of human embryo regulatory long terminal repeats (LTRs) (panels A, B), transpositionally active LINE1 in the human genome (panel C), and diverse panels of experimentally defined brain development GREs with different evolutionary origins (panels D-F).

Despite significant qualitative and quantitative differences in the sets of TFs bound to different GREs, the overall patterns of the PPI cascade chain reactions exhibit characteristics similar to the TF–TF PPI cascade profiles identified for 716 initiator TFs (compare PPI patterns in **Figures 1**, **2**). These consensus default features of the TF–TF PPI cascades include:

- An end-point saturation level of 245 recycling TFs achieved at PPI level 3.
- Enrichment of subsets of regulatory TFs among upregulated genes in specific human brain regions.
- Branching from the TF – TF PPI path to the HUB proteins PPI path, engaging 133 HUB proteins. Subsets of these HUB proteins are enriched in numerous neural synapse GO categories, as defined by the Synaptic GO 2024 database (compare patterns in **Figures 1**, **2**).

Notably, there are marked differences between the subsets of GRE-bound TFs that are enriched among upregulated genes in specific human brain regions. These differences translate into distinct qualitative and quantitative features of the human brain regions tagged by significant enrichment profiles of subsets of regulatory GRE-bound TFs (**Figure 2**; **Table 1**; **Supplementary Tables 1**, **2**; **Supplementary Data Set S1**, **S2**).

**Table 1:** Transcription factors (TFs) engaged in protein–protein interactions (PPIs) with 90 TFs bound to LTR5_Hs loci are enriched in 67 human brain regions defined by the Allen Brain Atlas database of upregulated genes.

Brain regions	Number of genes	P-value	Adjusted P-value	Odds ratio	Combined score	Genes
Crus 1, molecular layer	13	2.15E-06	0.004	5.5	72.4	MAX;PCGF2;CHD7;PAX3;AHR;HNF1A;ETS1;FOXP3;POU5F1;NEUROD1; TCOF1;RCOR2;FOXA2
Crus 1	12	1.20E-05	0.004	5.1	57.4	NEUROD1;TCOF1;MAX;PCGF2;CHD7;PAX3;AHR;HNF1A;FOXP3;RCOR2; POU5F1;FOXA2
Crus 2	12	1.20E-05	0.004	5.1	57.4	NEUROD1;TCOF1;MAX;PCGF2;CHD7;PAX3;AHR;KLF4;ETS1;RCOR2; POU5F1;FOXA2
Crus 2, granular layer	12	1.20E-05	0.004	5.1	57.4	NEUROD1;TCOF1;MAX;PCGF2;CHD7;NR1I2;PAX3;KLF4;ETS1;RCOR2; POU5F1;FOXA2
Crus 2, molecular layer	12	1.20E-05	0.004	5.1	57.4	NEUROD1;TCOF1;MAX;PCGF2;CHD7;PAX3;AHR;KLF4;ETS1;RCOR2; POU5F1;FOXA2
Postsubiculum, layer 3	12	1.20E-05	0.004	5.1	57.4	NR4A2;NR4A1;BCL11A;E2F1;TCF4;HOXD13;ZBTB33;GATA3;FOS;NR3C1; KLF4;ETS2
Ansiform lobule	11	6.17E-05	0.013	4.6	44.6	NEUROD1;TCOF1;MAX;PCGF2;CHD7;PAX3;AHR;ETS1;RCOR2;POU5F1; FOXA2
Posterolateral visual area, layer 6b	11	6.17E-05	0.013	4.6	44.6	NR4A1;PHC1;KLF5;NFYA;E2F1;EP300;CTNNB1;GATA3;NR3C1;POU2F2; FOXM1
Primary visual area, layer 6a	11	6.17E-05	0.013	4.6	44.6	NR4A1;PHC1;BHLHE40;HOXB4;E2F1;ERG;GATA3;FOS;NR3C1;KLF4;KLF2
Purkinje cell layer of CbHCx	11	6.17E-05	0.013	4.6	44.6	NEUROD1;TCOF1;MAX;PCGF2;CHD7;PAX3;AHR;FOXP3;RCOR2;POU5F1; FOXA2
Hindbrain	15	2.01E-04	0.018	3.2	27.0	MAX;CHD7;GATA4;AHR;GATA3;FOXP3;POU4F1;POU5F1;NEUROD1;AR;MYC;E2F2;RCOR2;NFE2L2;FOXA2
Anteroventral periventricular nucleus	10	2.88E-04	0.018	4.1	33.8	AR;HNF4A;BCL3;E2F1;E2F2;NR2F2;POU2F2;KLF4;ESR1;ATF3
Cerebrum	10	2.88E-04	0.018	4.1	33.8	MEF2A;EGR1;NR4A1;KDM5B;MEF2C;SALL1;BCL11B;CEBPD;SP3;E2F2
Crus 1, granular layer	10	2.88E-04	0.018	4.1	33.8	NEUROD1;TCOF1;MAX;PCGF2;CHD7;PAX3;AHR;HNF1A;RCOR2;FOXA2
Entorhinal area, medial part, ventral zone, layer 3	10	2.88E-04	0.018	4.1	33.8	NR4A2;YAP1;NCOR1;KLF5;CBX3;STAT3;E2F2;E2F4;ETS1;JUNB
Hemispheric regions	10	2.88E-04	0.018	4.1	33.8	NEUROD1;TCOF1;MAX;PCGF2;CHD7;PAX3;AHR;FOXP3;POU5F1;FOXA2
Medial preoptic nucleus, medial part	10	2.88E-04	0.018	4.1	33.8	SOX2;AR;CREB1;SETDB1;E2F1;E2F2;ERG;NR2F2;POU2F2;ESR1
Nucleus of the optic tract	10	2.88E-04	0.018	4.1	33.8	TCF7L2;CIITA;CBFB;BCL11B;SIN3B;HEY1;SP1;TCF12;PAX3;ZFPM2
Paraflocculus, molecular layer	10	2.88E-04	0.018	4.1	33.8	NEUROD1;HEY1;CHD7;PAX3;GATA4;LMX1B;ETS1;FOXP3;POU5F1;FOXA2
Posterolateral visual area, layer 6a	10	2.88E-04	0.018	4.1	33.8	NR4A1;PHC1;KLF5;CTNNB1;ZBTB33;GATA3;FOS;NR3C1;JUNB;POU5F1
Presubiculum, layer 3	10	2.88E-04	0.018	4.1	33.8	NR4A2;NR4A1;SALL1;KLF5;BCL11A;TCF4;ZBTB33;GATA3;NR3C1;ETS2
Pretectal region	10	2.88E-04	0.018	4.1	33.8	MEF2A;TCF7L2;CIITA;CBFB;BCL11B;SP1;PAX3;GATA3;ZFPM2;POU5F1
Cerebellar hemisphere	10	2.88E-04	0.018	4.1	33.8	NEUROD1;TCOF1;MAX;PCGF2;CHD7;PAX3;AHR;FOXP3;POU5F1;FOXA2
Cortex of cerebellar hemisphere	10	2.88E-04	0.018	4.1	33.8	NEUROD1;TCOF1;MAX;PCGF2;CHD7;PAX3;AHR;FOXP3;POU5F1;FOXA2
Internal granular layer of CbHCx	10	2.88E-04	0.018	4.1	33.8	NEUROD1;TCOF1;MAX;PCGF2;CHD7;PAX3;AHR;FOXP3;POU5F1;FOXA2
Layer 6a of PaS	10	2.88E-04	0.018	4.1	33.8	NR4A2;NR4A1;BCL11A;E2F1;TCF4;ZBTB33;FOS;NR3C1;KLF4;ETS2
Layer 6b of RSC	10	2.88E-04	0.018	4.1	33.8	NR4A1;PHC1;NFYA;BCL11A;E2F1;CTNNB1;FOS;NR3C1;FOXM1;KLF4
Mantle zone of CbH	10	2.88E-04	0.018	4.1	33.8	NEUROD1;TCOF1;MAX;PCGF2;CHD7;PAX3;AHR;FOXP3;POU5F1;FOXA2
Molecular layer of CbHCx	10	2.88E-04	0.018	4.1	33.8	NEUROD1;TCOF1;MAX;PCGF2;CHD7;PAX3;AHR;FOXP3;POU5F1;FOXA2
Prepontine hindbrain	10	2.88E-04	0.018	4.1	33.8	NEUROD1;MAX;PCGF2;CHD7;PAX3;AHR;FOXP3;RCOR2;POU5F1;FOXA2
r1 alar plate	10	2.88E-04	0.018	4.1	33.8	NEUROD1;TCOF1;MAX;PCGF2;CHD7;PAX3;AHR;FOXP3;POU5F1;FOXA2
Reticular formation of CoPD	10	2.88E-04	0.018	4.1	33.8	TCF7L2;CIITA;BCL11B;SIN3B;HEY1;SP1;TCF12;PAX3;CTNNB1;ZFPM2
Rhombomere 1	10	2.88E-04	0.018	4.1	33.8	NEUROD1;TCOF1;MAX;PCGF2;CHD7;PAX3;AHR;FOXP3;POU5F1;FOXA2
Sublayer 6a of OCx	10	2.88E-04	0.018	4.1	33.8	STAT5A;NR4A1;BHLHE40;HOXB4;E2F1;NR0B1;ERG;FOS;NR3C1;KLF2
Superficial stratum of cerebellar hemisphere	10	2.88E-04	0.018	4.1	33.8	NEUROD1;TCOF1;MAX;PCGF2;CHD7;PAX3;AHR;FOXP3;POU5F1;FOXA2
Basic cell groups and regions	9	0.001216	0.039	3.7	24.8	MEF2A;NEUROD1;MEF2C;SALL1;BCL11B;TCOF1;CHD7;E2F2;GATA3
Cerebellar cortex	9	0.001216	0.039	3.7	24.8	NEUROD1;MAX;PCGF2;CHD7;PAX3;AHR;RCOR2;POU5F1;FOXA2
Cerebellum	9	0.001216	0.039	3.7	24.8	NEUROD1;MAX;PCGF2;CHD7;PAX3;AHR;RCOR2;POU5F1;FOXA2
Entorhinal area, lateral part, layer 2b	9	0.001216	0.039	3.7	24.8	NR4A2;CEBPA;CBX3;HNF4A;LMX1B;ELK1;ETS1;HIF1A;EZH2
Flocculus, granular layer	9	0.001216	0.039	3.7	24.8	NEUROD1;MAX;CHD7;PDX1;GATA4;FOXP3;RCOR2;KDM6A;FOXA2
Medial amygdalar nucleus, posterodorsal part, sublayer a	9	0.001216	0.039	3.7	24.8	AR;CBX3;HNF1B;GATA3;GATA2;RUNX3;ESR1;FLI1;ESR2
Midbrain, motor related	9	0.001216	0.039	3.7	24.8	MEF2A;TCF7L2;SETDB1;SP1;PAX3;GATA3;PAX5;ZFPM2;POU4F1
Paraflocculus	9	0.001216	0.039	3.7	24.8	NEUROD1;HEY1;MAX;CHD7;LMX1B;ETS1;FOXP3;POU5F1;FOXA2
Primary visual area	9	0.001216	0.039	3.7	24.8	YY1;EGR1;NR4A1;KLF5;CEBPD;BHLHE40;FOS;NR3C1;KLF4
Retrosplenial area, dorsal part, layer 2/3	9	0.001216	0.039	3.7	24.8	ATF1;BCL11A;BHLHE40;HNF1A;ERG;ZFPM2;RCOR2;CHD1;SMARCA4
Simple lobule	9	0.001216	0.039	3.7	24.8	NEUROD1;MAX;PCGF2;CHD7;PAX3;AHR;ZFPM2;POU5F1;FOXA2
Simple lobule, granular layer	9	0.001216	0.039	3.7	24.8	NEUROD1;MAX;PCGF2;CHD7;NR1I2;PAX3;AHR;ZFPM2;FOXA2
Simple lobule, molecular layer	9	0.001216	0.039	3.7	24.8	NEUROD1;MAX;PCGF2;CHD7;PAX3;AHR;ZFPM2;POU5F1;FOXA2
Striatum-like amygdalar nuclei	9	0.001216	0.039	3.7	24.8	SOX2;MEF2A;E2F2;HNF1B;GATA3;ESR1;ETS2;FLI1;ESR2
Superior colliculus, motor related	9	0.001216	0.039	3.7	24.8	MEF2A;TCF7L2;CIITA;SP1;PAX3;GATA3;ZFPM2;POU4F1;KLF2
Superior colliculus, motor related, intermediate gray layer	9	0.001216	0.039	3.7	24.8	MEF2A;TCF7L2;CIITA;SP1;PAX3;NR0B1;GATA3;ZFPM2;KLF2
Superior colliculus, motor related, intermediate gray layer, sublayer a	9	0.001216	0.039	3.7	24.8	TCF7L2;CIITA;BCL11B;SP1;TCF12;PAX3;NR0B1;ZFPM2;KLF2
Central layers of TG	9	0.001216	0.039	3.7	24.8	TCF7L2;CIITA;TRIM28;SP1;TCF12;PAX3;ZFPM2;POU4F1;KLF2
Intermediate stratum of TG	9	0.001216	0.039	3.7	24.8	TCF7L2;CIITA;TRIM28;SP1;TCF12;PAX3;ZFPM2;POU4F1;KLF2
Layer 3 of CCx	9	0.001216	0.039	3.7	24.8	NR4A1;MEF2C;SOX17;BCL11A;BHLHE40;STAT4;NR0B1;HNF1A;ZFPM2
Layer 3 of OCx	9	0.001216	0.039	3.7	24.8	EGR1;NR4A1;KLF5;BHLHE40;HNF1A;FOS;NR3C1;KLF4;SMARCA4
Layer 3 of PrS	9	0.001216	0.039	3.7	24.8	NR4A2;NR4A1;SALL1;BCL11A;BHLHE40;TCF4;ZBTB33;NR3C1;ZFPM2
Layer 5 of PaS	9	0.001216	0.039	3.7	24.8	NR4A2;NR4A1;KLF5;BCL11A;TCF4;HOXD13;ZBTB33;NR2F2;NR3C1
Layer 6 of PaS	9	0.001216	0.039	3.7	24.8	NR4A2;NR4A1;BCL11A;E2F1;TCF4;HOXD13;ZBTB33;NR3C1;JUNB
Layer 6 of RSC	9	0.001216	0.039	3.7	24.8	NR4A1;PHC1;NFYA;BCL11A;E2F1;CTNNB1;NR3C1;FOXM1;JUNB
Mantle zone of TG	9	0.001216	0.039	3.7	24.8	TCF7L2;CIITA;TRIM28;SP1;TCF12;PAX3;NR0B1;ZFPM2;KLF2
r10 part of gigantocellular reticular area	9	0.001216	0.039	3.7	24.8	STAT5A;GTF2B;MYC;LMO2;RARA;HNF1A;POU4F1;RUNX1;NFE2L2
r9 part of paragigantocellular nucleus	9	0.001216	0.039	3.7	24.8	STAT5A;TRIM28;HNF4A;LMO2;MAFK;NANOG;ETS1;POU4F1;NFE2L2
Root	9	0.001216	0.039	3.7	24.8	MEF2A;NEUROD1;MEF2C;SALL1;BCL11B;TCOF1;CHD7;E2F2;GATA3
Superficial stratum of CoPD	9	0.001216	0.039	3.7	24.8	TCF7L2;ZEB1;CBFB;BCL11B;SIN3B;HEY1;TCF12;PAX3;ZFPM2
Superficial stratum of r9BI	9	0.001216	0.039	3.7	24.8	STAT5A;TRIM28;HNF4A;LMO2;MAFK;NANOG;ETS1;POU4F1;NFE2L2
Tectal gray formation	9	0.001216	0.039	3.7	24.8	TCF7L2;CIITA;TRIM28;SP1;TCF12;PAX3;NR0B1;ZFPM2;KLF2

A total of 172 TFs engaged in PPI with 90 TFs bound to LTR5_Hs loci at the TF–TF PPI level 1 (**Figure 2**) were subjected to gene set enrichment analysis (GSEA) using the Allen Human Brain Atlas database of upregulated genes to identify brain regions manifesting significant enrichment of subsets of TFs among genes with increased expression in specific neuroanatomical structures.

However, a significant direct correlation was observed between the number of GRE-bound TFs enriched among upregulated genes in specific human brain regions and the number of human brain regions manifesting significant enrichment (adjusted p-value < 0.05) of subsets of regulatory GRE-bound TFs among genes with increased expression levels in these brain regions (**Figure 2**; **Supplementary Data Set S2**).

**Figure 3:**
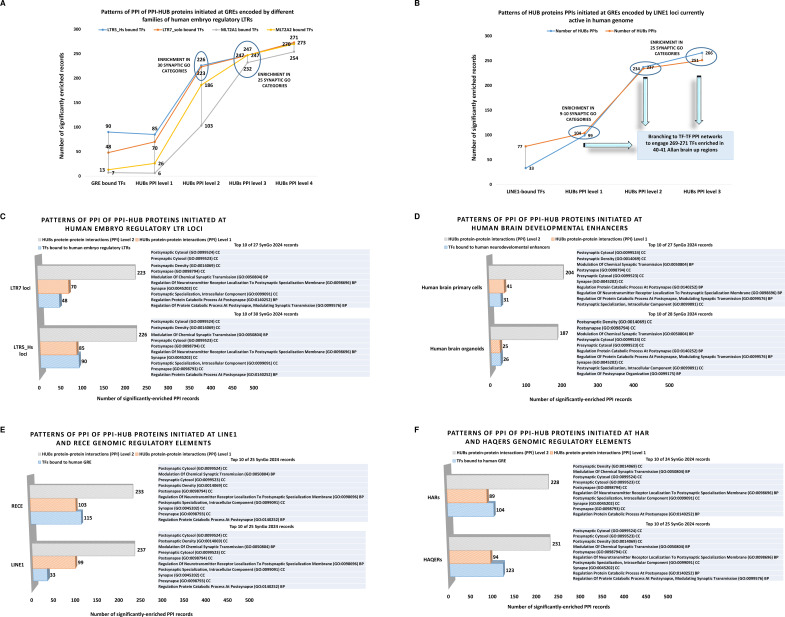
Visualization of TF-HUB protein–protein interaction (PPI) networks of initiator TFs that are bound to genomic regulatory elements (GREs) encoded by different families of human embryo regulatory long terminal repeats, LTRs (**A, C)**; transpositionally active in human genome LINE1 **(B, E)**; and experimentally defined brain development GREs of different evolutionary mechanisms of origin **(D–F)**. Transcription factors (TFs) bound to the indicated panels of GREs were identified employing the UniBind algorithm (Methods) and analyzed using the gene set enrichment analysis (GSEA) applied to the database of TF-HUB protein PPIs as described in the legend to the **Figures 1** and **2**. The number of proteins engaged in TF-HUB protein PPIs was defined by quantifying all proteins manifesting significantly enriched PPI profiles (adjusted p-value < 0.05) at each step of the HUB protein PPI cascade sequence and plotted for visualization. Significantly enriched proteins at the indicated steps of the PPI cascade were interrogated using the GSEA applied to the synaptic GO database **(A–F)** to identify significant enrichment of subsets of HUB proteins in the synaptic GO database-defined categories. Significantly enriched sets of protein identified near the saturation levels of the TF-HUB protein PPI cascade were interrogated using the GSEA applied to the TF–TF PPI database to document the “branching” effects of the PPI cascade switching from the TF-HUB protein PPIs to the TF–TF PPIs chains **(B)**. Follow-up GSEA of 269–271 TFs engaged in significantly enriched PPIs with HUB proteins near the saturation level of the PPI cascade revealed significant enrichment of subsets of identified TFs in the 40–41 neuroanatomical brain regions defined by the Allen human brain atlas database of upregulated genes **(B)**. **(C–F)** reports the results the GSEA of HUB proteins engaged in significantly enriched PPIs at the level 2 of the TF-HUB protein PPI cascade applied to the synaptic GO database to identify significant enrichment of subsets of identified HUB proteins in the synaptic GO database-defined categories for distinct sets of GREs, namely, LTR5_Hs and LTR7 **(C)**; human brain developmental enhancers of primary brain cells and brain organoids **(D)**; RECE and transpositionally active in human genome LINE1 **(E)**; HARs and HAQERs regulatory loci **(F)**.

**Figure 3** presents visualization examples of TF-HUB protein PPI networks. These networks originate from initiator TFs bound to GREs, which are encoded by different families of human embryo regulatory LTRs (panels A, C), transpositionally active LINE1 in the human genome (panels B, E), and experimentally defined brain development GREs with various evolutionary origins (panels D–F). A notable feature of the HUB proteins PPI chain reactions’ cascade is the uniform and significant enrichment of HUB protein subsets involved in protein complex formations at PPI level 2. This enrichment is observed across numerous neural synapse GO categories, as defined by the Synaptic GO 2024 database (**Figure 3**; **Table 2**; **Supplementary Data Sets S1**, **S2**). These findings suggest the essential roles of these proteins in synaptogenesis and in maintaining the structural protein ensembles and biological functions of mature synapses. Branching from the HUB proteins PPI path to the TF–TF PPI path revealed the engagement of 269–271 TFs. Subsets of these TFs are significantly enriched among genes whose expression is upregulated in defined neuroanatomical structures of the human brain (**Figure 3**; **Supplementary Data Set S2**).

**Table 2:** PPI Hub proteins engaged in the cascade of protein–protein interactions (PPI) with 90 transcription factors (TFs) bound to LTR5_Hs loci are enriched among the structural–functional components of multiprotein complexes of neuronal synapses recorded in the synaptic gene ontology (SynGO) and annotations database.

GO category	Number of genes	P-value	Adjusted P-value	Odds ratio	Combined score	Genes
Postsynaptic cytosol (GO:0099524) CC	11	9.62E-12	7.03E-10	25.2	640.3	UBE2I;SUMO1;NEDD4;SUMO2;PRKCD;CAMK2A; HTT;PIN1;CALM3;CALM1;MTOR
Postsynaptic density (GO:0014069) CC	16	4.40E-11	1.61E-09	10.3	246.0	RPL5;HSPA8;GSK3B;ARF1;CAMK2A;STAT3;ARRB1;ARRB2;PRKCZ;PAK1;CASP3;ABL1;MDM2; RPS3; MAPK1;VHL
Modulation of chemical synaptic transmission (GO:0050804) BP	13	9.10E-11	2.21E-09	14.0	323.0	ITGB1;UBE2I;STAT3;ARRB2;DNM1;GNAI2;MAPK9; MAPK8;HNRNPK;FYN;PLCG1;JAK2;MAPK3
Presynaptic cytosol (GO:0099523) CC	10	3.55E-10	6.47E-09	21.2	462.3	UBE2I;SUMO1;PRKCB;PRKCE;SUMO2;CAMK2A; HTT;PRKCA;CALM3;CALM1
Postsynapse (GO:0098794) CC	17	7.66E-09	1.12E-07	6.5	121.7	HSPA8;PTK2;RHOA;PPP1CA;EEF1A1;CDC42; PAK1;HNRNPK;CDK5;APC;CDH1;TRAF6; SUMO2;FLNA; RAC1;PRKACA;JAK2
Regulation of neurotransmitter receptor localization to postsynaptic specialization membrane (GO:0098696) BP	7	5.67E-08	6.90E-07	25.2	421.3	GSK3B;PAK1;TRAF6;CAMK2A;TNIK;PRKCZ; GABARAP
Synapse (GO:0045202) CC	15	1.11E-06	1.16E-05	5.1	69.4	YWHAE;RPL5;RPL3;RELA;PPP2CA;MAP1LC3A;YWHAQ;PPP2R1A;RPL27A;RPS3;RPS27A; HNRNPA1; SQSTM1;YWHAG;YWHAH
Postsynaptic specialization, intracellular component (GO:0099091) CC	4	4.92E-06	4.49E-05	50.9	621.8	LYN;LCK;SRC;GABARAP
Presynapse (GO:0098793) CC	11	7.40E-06	6.01E-05	5.9	69.3	HSPA8;MAP1LC3B;SUMO2;GRB2;TNIK;SLC2A4; PRKACA;YWHAG;YWHAH;GNAI2;PPP1CA
Regulation protein catabolic process at postsynapse (GO:0140252) BP	4	1.97E-05	1.43E-04	32.4	350.8	NEDD4;TRAF6;PIN1;VHL
Regulation of protein catabolic process at postsynapse, modulating synaptic transmission (GO:0099576) BP	3	2.78E-05	1.84E-04	88.7	930.2	CDH1;NEDD4;MDM2
Regulation pf synapse disassembly (GO:1905806) BP	3	2.20E-04	0.001	33.2	280.0	ITGB1;CDK5;SKP1
Regulation of postsynapse organization (GO:0099175) BP	5	3.55E-04	0.002	9.1	72.3	CDC42;HSPA8;HNRNPK;AKT1;VHL
Postsynapse to nucleus signaling pathway (GO:0099527) BP	3	4.73E-04	0.002	24.2	185.1	STAT3;HTT;RELA
Regulation of modification of postsynaptic structure (GO:0099159) BP	3	0.0012	0.006	16.6	111.6	CDC42;GSK3B;RHOA
Presynaptic endocytic zone membrane (GO:0098835) CC	2	0.0019	0.008	44.1	277.7	CLTC;DNM1
Presynaptic endocytosis (GO:0140238) BP	2	0.0019	0.008	44.1	277.7	CALM3;CALM1
Structural constituent of postsynaptic actin cytoskeleton (GO:0098973) BP	2	0.0019	0.008	44.1	277.7	ACTB;ACTG1
Regulation of synaptic vesicle endocytosis (GO:1900242) BP	3	0.0024	0.009	12.7	76.2	RAC1;ACTB;ACTG1
Postsynaptic density, intracellular component (GO:0099092) CC	4	0.0024	0.009	7.7	46.5	CTNNB1;FYN;TNIK;NR3C1
Maintenance of postsynaptic specialization structure (GO:0098880) BP	2	0.0026	0.009	35.3	210.5	ITGB1;HSPA8
Regulation of postsynaptic specialization assembly (GO:0099150) BP	2	0.0043	0.014	25.2	137.1	EEF1A1;ABL1
Postsynaptic ribosome CC	4	0.0077	0.025	5.5	26.6	RPL5;RPL27A;RPS3;RPS27A
Regulation of modification of postsynaptic actin Cytoskeleton (GO:1905274) BP	2	0.0121	0.037	13.6	59.9	PTK2;RHOA
Synaptic vesicle endocytosis (GO:0048488) BP	3	0.0126	0.037	6.6	29.0	CLTC;DNM1;SNCA
Presynaptic modulation of chemical synaptic transmission (GO:0099171) BP	3	0.0179	0.048	5.8	23.2	GSK3B;PRKCE;YWHAH
Translation at presynapse (GO:0140236) BP	3	0.0189	0.048	5.6	22.4	RPL5;RPL27A;RPS27A
Integral component of synaptic membrane (GO:0099699) CC	2	0.0192	0.048	10.4	41.0	ITGB1;EGFR
Translation at postsynapse (GO:0140242) BP	3	0.0199	0.048	5.5	21.7	RPL5;RPL27A;RPS27A
Presynaptic ribosome CC	3	0.0199	0.048	5.5	21.7	RPL5;RPL27A;RPS27A

A total of 226 HUB proteins that interact with 90 transcription factors (TFs) bound to LTR5_Hs loci at protein–protein interaction (PPI) network level 2 (**Figure 3**) were subjected to gene set enrichment analysis (GSEA) using the synaptic gene ontology (SynGO) database to identify SynGO records exhibiting significant enrichment of HUB protein subsets among genes with increased expression.

Collectively, these observations strongly support a foundational model of cooperative actions between two branches of PPI cascades originating from initiator TFs: the TF–TF PPI branch and the TF-HUB proteins PPI branch. These branches facilitate the operation of coherent regulatory frameworks, enabling the intrinsically integrated and congruent development of both specific neuroanatomical structures and the synaptic networks essential for their functions.

**Figure 4:**
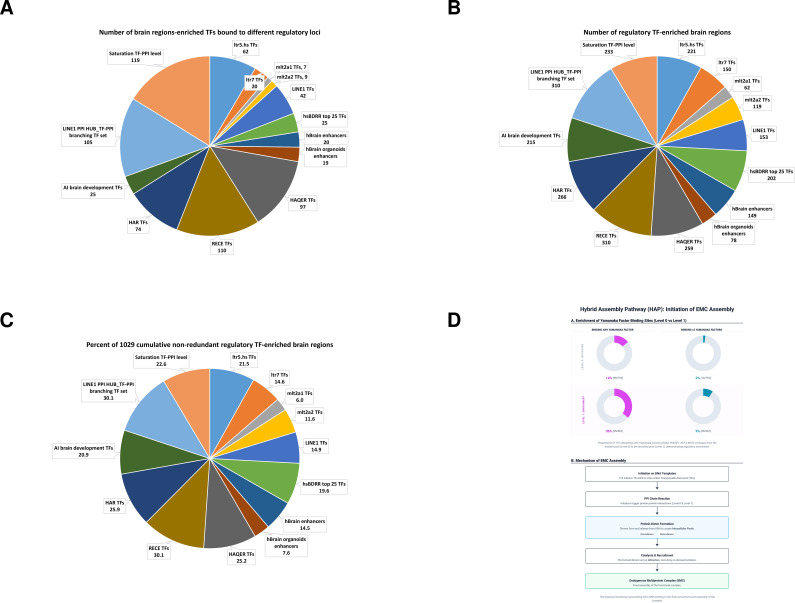
Summaries of the results of the gene set enrichment analysis (GSEA)-defined quantitative characteristics of the enrichment profiles of PPI cascades originating at initiator TFs bound to genomic regulatory elements (GREs), including GREs encoded by different families of human embryo regulatory long terminal repeats, LTRs (LTR5_Hs; LTR7; MLT2A1; MLT2A2); transpositionally active in human genome LINE1; and experimentally defined brain development GREs of different evolutionary mechanisms of origin, including human-specific brain development regulatory regions (hsBDRRs); human brain primary cells active enhancers (hBrain enhancers); human brain organoids active enhancers (hBrain organoids enhancers); artificial intelligence LLM agent Google Gemini-defined set of master brain development regulatory TFs (AI brain development TFs); highly conserved GREs with accelerated mutation acquisition during primate evolution (rapidly evolving conserved elements, RECE); highly conserved GREs with accelerated mutation acquisition on the human lineage (human accelerated regions, HARs; human ancestor quickly evolved regions, HAQERs). **(A)** shows the number of brain regions-enriched initiator TFs bound to different GREs. **(B)** shows the number of brain regions significantly enriched for subsets of initiator TFs bound to different GREs. **(C)** reports percentile values of initiator TFs-enriched brain regions attributed to the different GRE-bound TFs that were calculated as the percentage of 1,029 cumulative non-redundant initiator TF-enriched brain regions. **(D)** depicts the infographic summary of the key features identified herein of a hybrid assembly pathway (HAP) of EMCs. The top figure reports the observed pattern of enrichment of proteins binding the Yamanaka factors during the transition from initiator TFs bound to the DNA template (level 0) to the set of TFs engaged in PPIs with the initiator TFs at level 1 of the TF–TF PPI cascade. The bottom figure depicts the sequence of HAP facilitating EMC assembly via the creation of protein dimers and the on-demand recruitment of protein monomers to pre-assembled dimers.

Next, we performed integrative analyses of the GSEA-defined enrichment profiles of initiator TFs bound to multiple families of GREs (**Figure 4**; **Supplementary Data Set S2**). These analyses included GREs encoded by different families of human embryo regulatory LTRs (LTR5_Hs; LTR7; MLT2A1; MLT2A2) and transpositionally active LINE1 in the human genome. We also analyzed three distinct panels of experimentally validated brain development GREs of different evolutionary origins: hsBDRRs, human brain primary cell active enhancers (hBrain enhancers), and human brain organoid active enhancers (hBrain organoid enhancers). The analysis further incorporated AI brain development TFs, a set of highly conserved GREs with accelerated mutation acquisition during primate evolution coinciding with the onset of brain size expansion on the primate lineage (rapidly evolving conserved elements, RECE), and two distinct panels of highly conserved GREs with accelerated mutation acquisition on the human lineage (human accelerated regions, HARs; human ancestor quickly evolved regions, HAQERs).

The results demonstrate that different GRE families have made consistent yet highly variable contributions to genomic regulatory networks governing human brain development. This is reflected in the distinct spectrum and the number of initiator TFs bound to specific GREs (**Figure 4**; **Supplementary Data Set S2**). Despite markedly different genomic contexts and timelines of the evolutionary origins of the analyzed GREs, subsets of specific panels of GRE-bound regulatory TFs are consistently and significantly enriched among genes overexpressed in diverse ensembles of hundreds of defined neuroanatomical structures of the human brain (**Figures 3**, **4**; **Supplementary Data Set S2**). Collectively, these observations strongly suggest that the GRE-bound initiator TFs and TFs engaged in PPI at various stages of the TF–TF PPI cascade, as reported in this contribution, may operate as regulators of brain development and as transcriptional regulators maintaining the structural–functional and morphological integrity of mature neuroanatomical regions.

One of the main postulates of the HAP concept is that initiator TFs should be detectable as DNA**–**TF–TF complexes *in vivo*. Xie et al. (2025) attempted to address the challenging problem of identifying TFs involved in forming high-confidence DNA**–**TF–TF complexes. They identified 106 TFs as constituents of these high-confidence complexes. Our analyses demonstrate that 103 of these 106 TFs (97%) reported by Xie et al. (2025) are members of the consensus panel of 716 TFs having TE-seeded TFBS (**Supplementary Data Set S1**). GSEA, using the Allen Brain Atlas database of upregulated genes, revealed that subsets of these 103 initiator TFs are significantly enriched in 53 distinct neuroanatomical structures, while subsets of the full set of 106 TFs are enriched in 65 brain regions (**Supplementary Data Set S1**). In contrast, GSEA using the Allen Brain Atlas database of downregulated genes identified no brain regions significantly enriched in the subsets of either 103 or 106 TFs (**Supplementary Data Set S1**). These observations provide independent confirmation of the validity of the HAP model.

### Features of the PPI cascade profiles of the YF-associated TF–TF PPI chain reactions emanating from the initiator TFs bound to TE-seeded DNA templates

Notably, follow-up analyses of TF panels involved in PPIs at various steps of the TF–TF PPI cascade revealed an enrichment pattern of proteins binding the Yamanaka factors. This enrichment was observed during the transition from initiator TFs bound to a DNA template (PPI level 0) to the set of TFs engaged in PPIs with initiator TFs at PPI level 1 of the TF–TF PPI cascade (**Figure 4C**; top panel). The initial observations regarding the prominent participation of the YF-associated PPI network in the TF–TF PPI cascade can be summarized as follows:

1. Four of the 716 initiator TFs are YFs (SOX2, POU5F1, KLF4, and MYC). Additionally, 99 of the 716 initiator TFs (14%) bind at least one YF via PPI, and 14 of these 99 YF-binding TFs (2% of 716 TFs and 14% of 99 TFs) can bind at least two YFs via PPI.
2. In total, initiator TFs bound to the DNA template can bind 261 different TFs via PPI at PPI level 1, and all four YFs are constituents of the PPI level 1 TF panel.
3. The four YFs can bind 95 TFs (36% of 261) via PPI at PPI level 1. Furthermore, 23 of the 261 PPI level 1 TFs (9% of 261 TFs and 24% of 95 TFs) can bind at least two YFs via PPI.
4. A total of 166 TFs (23% of 716 initiator TFs) from the PPI level 1 layer are selected from the 716 initiator TFs, including all four YFs. Moreover, 45 TFs (45% of 99 TFs) from the PPI level 1 layer are selected from the 99 initiator TFs that can bind at least one YF via PPI.

Consistent with the idea that YF-associated transcriptional regulatory networks and signaling pathways are involved in the chain reactions of the TF–TF PPI cascade, GSEA of initiator TFs and constituent TFs at different PPI cascade levels revealed consistent patterns of their enrichment among genes identified as markers of multiple types of human and mouse stem and progenitor cells. These include embryonic stem cells; neural stem cells and neural progenitor cells; cardiac progenitor cells; human heart cardiovascular progenitor cells; skeletal muscle pluripotent stem cells; hematopoietic stem cells; human blood circulating fetal cells; pancreatic progenitor cells; liver stem cells; endothelial progenitor cells; human brain radial glia cells; human ovary and testis fetal and adult germ cells; and various types of cancer stem cells, among others (**Supplementary Data Set S1**; GSEA tables of the CellMarker 2024 database). These findings suggest that in-depth analyses focused on the YF-associated TF–TF PPI cascade could be of significant interest.

**Figure 5:**
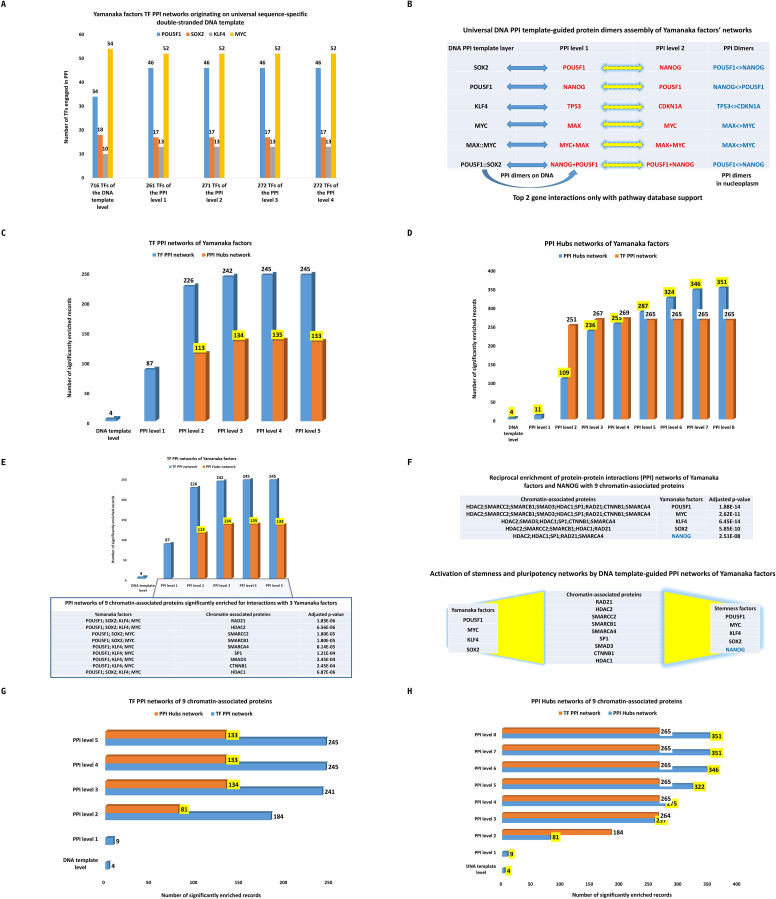
Overview of the quantitative features and the protein–protein interaction (PPI) cascade profiles of the Yamanaka Factors (YFs) – associated TF–TF PPI chain reactions emanating from the initiator TFs bound to TE-seeded DNA templates. Bar graphs shown in **(A)** reflect the total counts of different TFs engaged in PPI with specific YFs at different levels of TF–TF PPI chain reaction cascade, indicating that the maintenance of the highly consistent pattern YF-binding profiles represents a hallmark feature of the TF–TF PPI cascade. **(B)** depicts the pairs of TFs that interact via PPI to create the protein-protein dimers containing the YFs at the indicated TF-PPI levels. In the analyses reported in the **(C, D)** four YFs were imputed as the initiator TFs and analyzed using the gene set enrichment analysis (GSEA) applied to the database of TF–TF PPIs **(C)** or the database of TF-HUB protein PPIs **(D)** as described in the legend to the **Figures 1** and **2**. The number of proteins engaged in TF–TF PPIs and TF-HUB protein PPIs were defined by quantifying all proteins manifesting significantly enriched PPI profiles (adjusted p-value < 0.05) at individual steps of the corresponding PPI cascade sequence and plotted for visualization. **(E)** reports data documenting the discovery of nine chromatin-associated proteins, each of which is significantly enriched in the formation of protein-protein complexes with three YFs. These nine chromatin-associate proteins remain among constituents of the significantly enriched proteins at the level 1 of the PPI cascade and all subsequent steps of the PPI chain reactions. **(F)** reports observations that five of nine YF-binding chromatin-associated proteins are significantly enriched in PPI with the transcription factor NANOG that is one the essential master transcriptional regulators of stemness and pluripotency phenotypes. These findings suggest that if the NANOG protein is readily available for engagement in the TF–TF PPI network, the stemness and pluripotency phenotypes may constitute the default cellular state. In the analyses reported in the panels G and H, four YFs were imputed as the initiator TFs and nine YF-binding chromatin-associated proteins were imputed as the only proteins available for PPI at the level 1 of the PPI cascade and PPI chain reactions were analyzed using the GSEA applied to the database of TF–TF PPIs **(G)** or the database of TF-HUB protein PPIs (panel **(H)**) as described in the legend to the **Figures 1** and **2**. The number of proteins engaged in TF–TF PPIs and TF-HUB protein PPIs was defined by quantifying all proteins manifesting significantly enriched PPI profiles (adjusted p-value < 0.05) at individual steps of the corresponding PPI cascade sequence and plotted for visualization. These observations illustrate that the PPI network limited at the initiator TFs level to four YFs and/or at the PPI level 1 to the nine YF-binding chromatin-associate proteins consistently recapitulate the essential features of PPI cascades initiated at the TE-seeded DNA templates.

Results of the analyses of the PPI chain reactions centered on the YFs and YF-binding proteins are summarized in **Figure 5**. Notably, the number of TFs with a predilection for binding specific YFs remains constant from TF–TF PPI cascade level 1 through the TF–TF PPI cascade saturation stage of recycling 245 TFs engaged in the PPI chain reactions (**Figure 5A**). The constitutive patterns of YF-binding TFs consist of 46, 17, 13, and 52 TFs with a predilection for binding the POU5F1, SOX2, KLF4, and MYC proteins, respectively. Considering the top two gene interactions (Methods) with evidence requiring support from pathway database records (UCSC Genome Browser Gene Interaction Graph), these observations indicate that the YF-associated PPI chain reactions may generate protein-protein dimers of the following compositions: POU5F1<>NANOG, MAX<>MYC, and TP53<>CDKN1A (**Figure 5B**), thus implying the engagement of stemness and cell cycle progression regulatory networks. Significantly, the patterns of either TF–TF PPI or TF-HUB proteins PPI cascades initiated by 4 YFs fully recapitulate the corresponding PPI cascade profiles originated by 716 initiator TFs (compare **Figures 1A** and **5C** for TF–TF PPI cascades, and **Figures 1C** and **5D** for TF-HUB proteins PPI cascades). Altogether, these observations suggest that the YF-associated PPI chain reactions may represent the foundational core of the evolutionary origin of the Hybrid Assembly Pathways (HAP) of EMCs by driving the recruitment of more diverse panels of initiator TFs to fulfill the evolutionary needs for increasing complexity of the PPI cascade of chain reactions in various types of differentiated cells.

In agreement with this hypothesis, follow-up analyses of protein constituents at PPI levels 1 through 5 of the PPI cascade identified nine chromatin-associated proteins (**Figures 5E, F**). Each of these proteins exhibits a propensity to bind three to four YFs, with each YF protein, in turn, showing a predilection to bind five to nine of these chromatin-associated proteins (**Figure 5F**). Notably, five of these YF-binding chromatin-associated proteins tend to bind the transcription factor NANOG, a master regulator of the stemness phenotype. These findings suggest the potential for distinct regulatory networks to emerge in cells where NANOG is expressed and readily available for PPI engagement. Such cells may experience a realignment of HAP operation toward a stemness regulatory state, potentially transitioning to a stem cell-like phenotype. Conversely, in the absence of NANOG expression, HAP operation in cells may realign toward the acquisition of differentiated phenotypes. These observations align with previous findings illustrating how prominent human-specific features of pluripotency regulatory networks link NANOG with fetal and adult brain development (Glinsky, 2017).

Consistent with this reasoning, the PPI cascade patterns initiated by four YFs, but limited to the engagement of nine chromatin-associated proteins at PPI level 1, appear to fully recapitulate the corresponding PPI cascade profiles originated by 716 initiator TFs (compare **Figures 1A**, **5G** for TF–TF PPI cascades, and **Figures 1C** and **5H** for TF-HUB proteins PPI cascades). Notably, direct experimental evidence has been reported for cognitive rejuvenation through targeted partial reprogramming of engram neurons by three Yamanaka factors: POU5F1 (OCT4), SOX2, and KLF4 or OSK (Berdugo-Vega et al., 2026). Reportedly, OSK-mediated gene therapy delivered to engram neurons reversed the expression of cellular hallmarks of aging and Alzheimer’s disease (AD), normalized synaptic plasticity-associated epigenetic and transcriptional profiles, and improved neuronal hyperexcitability typical of AD. Consistent with cognitive rejuvenation, OSK-mediated reprogramming of engram neurons appears to restore learning and memory capacities to levels observed in healthy young animals (Berdugo-Vega et al., 2026).

### Pioneer TFs as core constituents of chromosome-naive networks of initiators of PPI cascades facilitating creation of protein dimers for EMC assembly

Yamanaka factors (POU5F1, SOX2, and KLF4, act as pioneer TFs. They achieve this by binding to partially exposed sequences on nucleosome-wrapped DNA within compacted chromatin. This interaction influences the mobility of nucleosomes and the stability of histone-DNA complexes, thereby making nucleosome-free DNA sequences accessible to other TFs and chromatin remodelers (Zaret, 2020; Soufi et al., 2012; 2015; Sinha et al., 2023). By binding to specific target sites in compacted chromatin, YFs promote the transition from silent to accessible chromatin domains and recruit other TFs. This allows them to function as master regulators in embryonic development, cell differentiation, and cellular reprogramming, with POU5F1 appearing to have the most significant impact on these processes (Takahashi and Yamanaka, 2006; Kim et al., 2009; Wu et al., 2011; Soufi et al., 2012; 2015).

It is possible that an underappreciated mechanism of biological activity for specific TFs capable of binding nucleosome-wrapped DNA (Zhu et al., 2018; Fernandez Garcia et al., 2019), including YFs and other pioneer TFs, involves their role as the foundational core of initiator TFs for the HAP of EMCs. Supporting this hypothesis, numerous TFs known to bind nucleosome-wrapped DNA (FOXA1, FOXA2, ASCL1, GATA4, GATA6, PBX1, PU.1/SPI1, PAX7, FOXD3, ISL1, CLOCK, EBF1, CEBPA, SOX11, and TBX factors) are among the 716 initiator TFs identified in this study.

**Figure 6:**
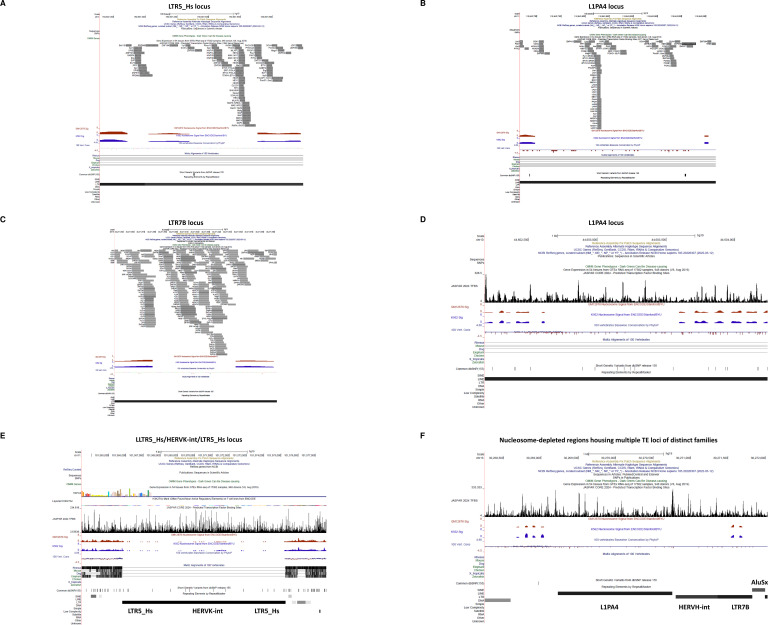
The UCSC genome browser visualization of nucleosomes’ distribution profiles and placements of TFBS clusters within nucleosome-devoided regions located within different GREs encoded by distinct families of TEs, namely, LTR5_Hs locus on chr1 **(A)**; L1PA4 locus on vhr11 **(B)**; LTR7B locus on chr16 **(C)**; L1PA4 locus on chr10 **(D)**; LTR5_Hs/HERVK-int/LTR5_Hs locus on chr11 **(E)**; composite nucleosome-depleted regions on chr16 within the continuous DNA sequence encoded by TE loci of distinct families, including L1PA4; HERVH-int; LTR7B; AluSx **(F)**.

Given that pioneer TF activity is linked to chromatin decompaction, H2A.Z histone recruitment to nucleosomes, and nucleosome loss (summarized in Zaret, 2020), it may be possible to identify genomic context signatures of pioneer TF activity within TE-encoded GREs harboring initiator TF binding sites by analyzing nucleosome distribution patterns. The results of these analyses are presented in **Figure 6** and **Supplementary Data Set S2**. A visualization survey using the UCSC Genome Browser on subsets of TE-encoded GREs revealed multiple striking examples of large, continuous nucleosome-depleted regions within sequences of various retrotransposon families that have been repurposed to function as GREs in human cells (**Figure 6**).

Next, systematic analyses of nucleosome density distribution patterns were performed on previously reported datasets, including ATAC-seq-defined differentially accessible regulatory regions, control ATAC-seq regions, and 1,550 TE-encoded GREs involved in TE-governed human hippocampal neurogenesis regulatory pathways (Patoori et al., 2022; Glinsky, 2026). The number of nucleosomes and nucleosome-devoid regions were cataloged for individual, structurally related sets of GREs. These included 3,000 ATAC-seq control regions, 3,000 ATAC-seq-defined differentially accessible regulatory regions, and 1,550 TE-encoded GREs, which were further segregated into subsets derived from LINE, LTR, SINE/Alu, SINE/MIR, and DNA families of TEs. For each set of GREs, corresponding records were computationally processed to calculate the number of nucleosomes and continuous nucleosome-devoid regions. The statistical significance of the differences between observed values was computed using the two-tailed p-value of Fisher’s exact test (Methods; **Supplementary Data Set S2**). To visualize the distribution patterns of nucleosomes and continuous nucleosome-devoid regions, measurements were normalized to calculate two sets of values for each GRE family: per-locus values and values per 1-Kb length of the GRE sequences.

**Figure 7:**
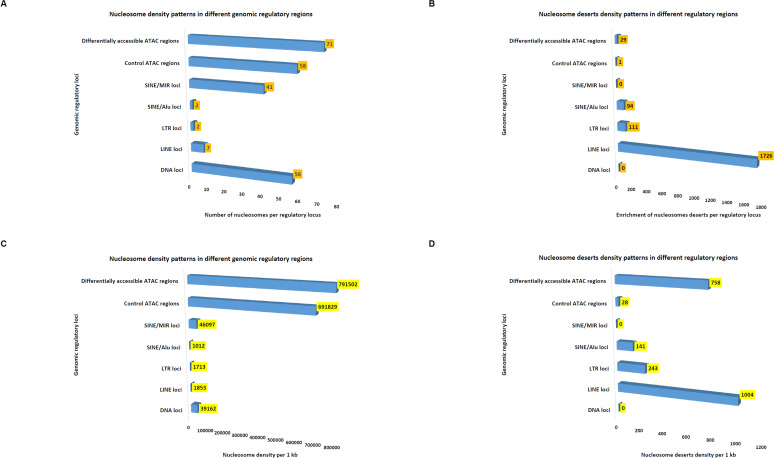
Patterns of distributions of nucleosomes and nucleosome deserts within different families of GREs. Summaries of the results of systematic analyses of patterns of nucleosome density distributions are shown. Analyses were carried out on previously reported data sets of ATAC-seq-defined differentially accessible regulatory regions, control ATAC-seq regions, and 1,550 TE-encoded GREs engaged in TE-governed human hippocampal neurogenesis regulatory pathways (Methods; **Supplementary Data Set S2**). The number of nucleosomes and the number of continuous nucleosome-devoid regions of various lengths were cataloged for individual structurally related sets of GREs, including 3,000 ATAC-seq control regions and 3,000 ATAC-seq-defined differentially accessible regulatory regions, as well as 1,550 TE-encoded GREs segregated into subsets of loci derived from LINE; LTR; SINE/Alu; SINE/MIR; and DNA families of TEs. The corresponding records were computationally processed for each set of GREs. The number of nucleosomes and the number of continuous nucleosome-devoid regions were computed and the statistical significance of the differences between the observed values were estimated using the 2-Tail p-value of the Fisher’s exact test (Methods; **Supplementary Data Set S2**). To visualize the distribution patterns of nucleosomes **(A, C)** and continuous nucleosome-devoid regions **(B, D)**, mean values of corresponding measurements were normalized to identify two sets of metrics for each family of GREs: the per-locus values **(A, B)** and values per 1-Kb length of the GRE sequences **(C, D)**. The continuous nucleosome-devoid regions of the 50–1,000 nt in length were designated the nucleosome deserts.

Results from these analytical experiments clearly demonstrate the consistent presence of continuous nucleosome-devoid regions, 50–1,000 nt in length (hereafter designated “nucleosome deserts”), within the DNA sequences of GREs encoded by LINE, LTR, or SINE/Alu families of TEs (**Figure 7**; **Supplementary Data Set S2**). In contrast, no nucleosome deserts were detected within GREs encoded by SINE/MIR or DNA families of TEs (**Figure 7**; **Supplementary Data Set S2**). On a per-locus basis, GREs encoded by LINE-derived sequences showed the highest enrichment levels (1,726-fold) of nucleosome deserts compared to control ATAC-seq-defined regions, followed by LTR-derived and SINE/Alu-derived DNA sequences (111-fold and 94-fold enrichments, respectively). Interestingly, the density of nucleosome deserts was 29-fold higher in ATAC-seq-defined differentially accessible regulatory regions compared to control ATAC-seq regions, likely reflecting the relative enrichment of TE-derived GREs within the ATAC-seq-defined differentially accessible regulatory regions of human hippocampal neurogenesis (Patoori et al., 2022).

Accounting for differences in GRE loci lengths, estimates on a per 1-Kb basis of GRE sequence length revealed that GREs encoded by LINE-derived sequences exhibited the highest enrichment levels (36-fold) of nucleosome deserts, followed by LTR-derived and SINE/Alu-derived DNA sequences (nine-fold and five-fold, respectively). Consistently, the density of nucleosome deserts was 27-fold higher in ATAC-seq-defined differentially accessible regulatory regions compared to control ATAC-seq regions (**Figure 7**; **Supplementary Data Set S2**).

The opposite enrichment patterns were observed in the nucleosome density analyses. Specifically, evaluations of nucleosome density profiles demonstrated that GREs encoded by SINE/Alu, LTR, and LINE sequences exhibit the lowest nucleosome densities compared to other GREs (**Figure 7**; **Supplementary Data Set S2**). These differences were consistently detected regardless of the normalization methodology used for the estimates, whether based on the number of events per GRE locus or per 1 Kb of GRE sequence length.

Collectively, these observations define a genomic context signature for TE-encoded GRE loci, characterized by two concurrently emerging features: a marked increase in the number and density of nucleosome deserts and a decrease in nucleosome density. This epigenetic signature is most prominent within the genomic contexts of GREs derived from LINE, LTR, and SINE/Alu families of TEs. These findings support the hypothesis that pioneer TFs, upon binding to nucleosome-wrapped DNA sequences, trigger a cascade of molecular events. This cascade results in an increased density of continuous nucleosome deserts exceeding 50 nt in length, where the initiator TFs of HAP of EMCs reside and operate. Based on current knowledge, the most likely pioneer TF-triggered molecular scenario leading to the creation of nucleosome deserts involves the recruitment of the H2A.Z histone to nucleosomes, alterations in nucleosome structure, changes in nucleosome-DNA binding dynamics, and ultimately nucleosome loss.

### Inferences of potential mechanistic impacts of HAP and initiator TFs on the development and functions of the central nervous system

It was of interest to ascertain how the concept of the HAP of EMCs may impact our understanding of the exceedingly complex and highly coordinated networks governing the development and functions of thousands of neuroanatomical regions in the central nervous system (CNS). Synaptogenesis and the operation of mature synapses are indispensable for the functionally coherent integration of thousands of neuroanatomically distinct structures within the central and peripheral nervous systems. Therefore, it was of interest to determine whether experimentally tractable models of synaptogenesis could be inferred based on the mechanisms described herein, specifically the PPI cascade chain reactions initiated by initiator TFs to kick-start the operation of the HAP of EMCs.

**Figure 8:**
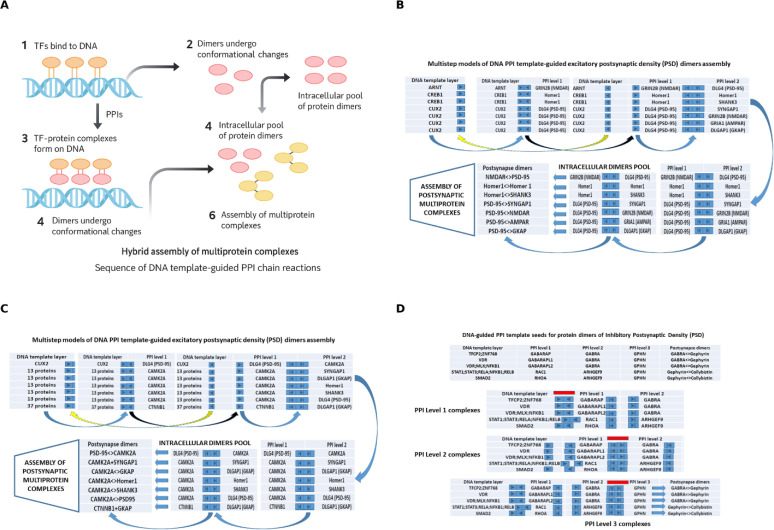
A simplified infographic model depicting the sequence of protein–protein interaction (PPI) chain reactions of the hybrid assembly pathway (HAP) of endogenous multiprotein complexes **(A)** and examples of multistep sequences of DNA PPI template-guided postsynaptic density (PSD) protein dimers assembly of excitatory **(B, C)** and inhibitory **(D)** synapses. **(A)** shows a simplified infographic model of the sequence of DNA template-guided PPI chain reactions of the HAP of endogenous multiprotein complexes. According to the HAP hypothesis, the creation of protein dimers by PPI cascades begins on DNA templates occupied by initiator TFs (1), followed by the sequential recruitment by DNA–TF complexes of the first set of protein constituents of EMCs (EMC protein dimerization primers that are known to form protein-protein complexes with the initiator TFs) and the second sets of protein constituents of EMCs that are known to form protein-protein complexes with the EMC protein dimerization primers. Newly formed protein–protein dimers undergo conformational changes, facilitating their release from DNA templates and creation of intracellular pools of protein dimers. The release of dimers from the DNA–TF complexes enables the initiation of a new PPI cascade cycle and the delivery of protein dimers to the specific EMC assembly compartments, where dimers function as attractors accelerating the on-demand recruitment of protein monomers required for the EMC assembly completion. **(B–D)** show working models of the PPI cascade analyses-supported specific examples the initiation steps of formation protein dimers serving as catalysts of the assembly of postsynaptic density (PSD) multiprotein complexes of the excitatory **(B, C)** and inhibitory **(D)** synapses. Proposed sequential steps of the cascade of PPIs starting at DNA template PPI initiation stage (the box designated DNA template layer) where bound to DNA specific transcription factors (TFs) function as the initiator TFs recruiting designated PSD proteins (defined as the PSD dimer primers that are known to form protein-protein complexes with initiator TFs) to form the PPI level 1 complexes. This process constitutes the PSD dimers assembly initiation stage at DNA template, which is followed by the recruitment of the second sets of PSD proteins that are known to form protein-protein complexes with the proteins designated PSD dimer primers. The postulated biological function of these sets of PPIs is to facilitate the PSD dimers assembly at DNA template. Interactions of 2 protein components of PSD constitute the PPI level 2 stage, where PSD dimers components recruitment stage at DNA template is completed, PSD dimers are assembled. Thus, recruitment of the second-stage PSD proteins enables assembly of PPI level 2 complexes and completion of PSD dimer formation on the DNA template. Upon conformational rearrangement, PSD dimers are released from the DNA–TF complexes, initiating a new PPI cycle while simultaneously generating the intracellular pool of PSD dimers. These preformed protein dimers are delivered to synaptic assembly compartments, where they promote postsynaptic density assembly by acting as attractors catalyzing the on-demand recruitment of additional protein monomers to complete the assembly of multiprotein PSD complexes.

To this end, the proteins comprising structural components of the post-synaptic densities (PSD) of excitatory and inhibitory synapses were identified among the initiator TFs, as well as the sets of proteins engaged in PPI from level 1 through level 3 of the PPI cascades. Next, a catalog of protein–protein dimers was assembled based on experimentally documented PPIs between the identified sets of proteins, and models for the creation of protein-protein dimers designed to facilitate synaptogenesis were built. The results of these analytical experiments are summarized in **Figure 8**. Multiple combinations of protein-protein dimers have been inferred as constituents of the intracellular pools of protein dimers, serving as potential precursors of the PSD multiprotein complexes of excitatory synapses (**Figures 8B, C**). Protein dimers designated as precursors for the assembly of PSD multiprotein complexes, upon arrival at the synaptogenesis cellular compartments, function as attractors for the on-demand recruitment of additional proteins required to complete the assembly of fully functional PSD structures. Notably, a more limited selection of protein-protein dimers was discerned as constituents of the intracellular pools of protein dimers, serving as potential precursors of the PSD multiprotein complexes of inhibitory synapses (**Figure 8D**). This yielded an estimated inhibitory to excitatory synaptic ratio of 1 to 7 for the PSD precursor protein dimers. These findings appear to agree with the reported excitatory to inhibitory neuron synaptic ratio range of at least 2/1, which reportedly increased from 4.5/1 to 10/1 in different layers of the human brain (Jorstad et al., 2023).

Previous work described a panel of 25 regulatory TFs that showed human-specific enrichment of binding to brain development GREs. These TFs were identified using the UniBind differential-enrichment analysis algorithm (Methods) on TFs bound to 8,099 human-specific BDRRs versus 9,836 chimpanzee-specific BDRRs (Glinsky, 2026). Notably, follow-up GSEA demonstrated that expression levels of subsets of these 25 TFs are significantly enriched among genes upregulated in 202 human brain regions. This suggests that these brain development regulatory TFs may play a role in executing transcriptional programs responsible for maintaining the structural–functional integrity of mature neuroanatomical regions (Glinsky, 2026).

Significantly, present analyses revealed that all 25 brain development regulatory TFs are constituents of the foundational panel of 716 initiator TFs characterized herein. In response to a request to explore the validity of the hypothesis that a small panel of 25 TFs regulates brain development across numerous brain regions, the AI assistant Google Gemini reported the discovery of such a master panel of 25 TFs and generated an expert-level analysis of them (**Supplementary Data Set S3**). Interestingly, while only three TFs (SOX2, OTX2, and EOMES) are common to both sets of brain development regulatory TFs, 21 of the 25 (84%) brain development regulatory TFs identified by the AI assistant are constituents of the foundational panel of 716 initiator TFs characterized herein.

In agreement with the concept that brain development regulatory TFs play a role in executing transcriptional programs responsible for maintaining the structural–functional integrity of mature neuroanatomical regions (Glinsky, 2026), follow-up GSEA demonstrated that expression levels of subsets of the AI assistant’s discovered panel of 25 brain regulatory TFs are significantly enriched among genes upregulated in 215 human brain regions (**Supplementary Data Set S3**). An expert-level analysis of the main structural–functional characteristics and developmental trajectories of these 215 brain regions is reported in **Supplementary Data Set S3**. Of note, only 17 brain regions are common between the 202 brain regions identified by GSEA of 25 TFs differentially bound to human versus chimpanzee BDRRs (Glinsky, 2026) and the 215 brain regions identified by GSEA of the 25 brain development regulatory TFs identified by the AI assistant.

**Figure 9:**
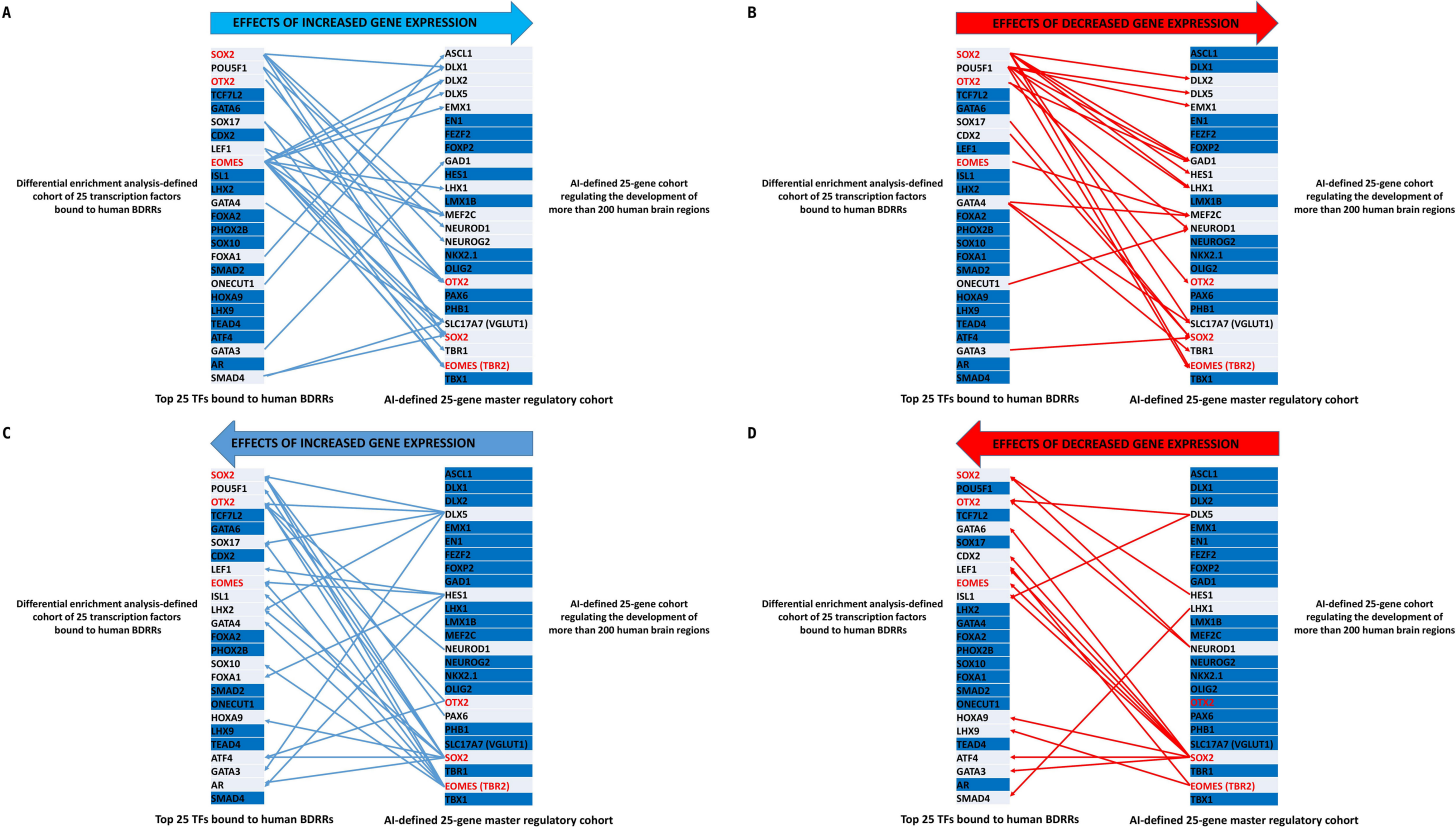
Transcriptional regulatory crosstalks between the network of 25 transcription factors (TFs) defined by the AI LLM model Google Gemini as the master transcriptional regulators of human brain neuroanatomical structures and the set of 25 TFs identified as human-specific transcriptional regulators of brain development by comparisons of TFs bound to human-specific brain development regulatory regions (BDRRs) vis-à-vis TFs bound to chimpanzee-specific BDRRs (Glinsky, 2025). Employing the Gene Expression Omnibus databases of upregulated and downregulated genes recorded in response to targeted gene-specific manipulations to achieve the knockout, downregulation, or overexpression, each individual TF constituting the corresponding 25-gene TF sets was analyzed for experimental evidence documenting its regulatory impacts on expression of all TFs in the counterpart set. Statistically significant findings were tabulated and plotted for visualization. **(A, B)** report the results documenting effects of increased gene expression **(A)** or decreased gene expression **(B)** of individual TFs comprising the panel of 25 TFs preferentially bound to human-specific BDRRs on TFs of the AI LLM Gemini-defined master transcriptional regulators of brain development. Conversely, **(C, D)** reports report the results documenting effects of increased gene expression **(C)** or decreased gene expression **(D)** of individual TFs comprising the cohort of AI LLM Gemini-defined master transcriptional regulators of brain development on TFs comprising the panel of 25 TFs preferentially bound to human-specific BDRRs. Genes encoding the SOX2; EOMES; and OTX2 TFs are shared by both 25-gene panels, while all 25 TFs bound to the human-specific BDRRs and 21 of 25 TFs (84%) of the AI LLM Gemini-defined master transcriptional regulators of brain development are constituents of the cohort of 716 initiator TFs.

The expert-level analysis of the key structural–functional features of the AI assistant-defined panel of 25 brain development regulatory TFs is reported in **Supplementary Data Set S3**. This analysis highlights their overlapping and distinct regulatory characteristics compared with the set of 25 TFs identified by the UniBind differential-enrichment analyses of TFs bound to human-specific versus chimpanzee-specific BDRRs (Glinsky, 2026). Significantly, follow-up analyses employing the Gene Expression Omnibus (GEO) databases of upregulated and downregulated genes revealed apparent extensive transcriptional regulatory crosstalks between two independently identified cohorts of 25 TFs contributing to brain development (**Figure 9**). Interestingly, most of the effects of both increased and decreased expression appear mediated by relatively small subsets of TFs in each panel, particularly a set of three TFs common to both panels: SOX2, OTX2, and EOMES (**Figure 9**). Collectively, these observations imply that transcriptional regulatory crosstalks between the two panels of 25 TFs described herein may impact the development and functions of hundreds of neuroanatomical structures in the human brain.

### Insights into human-specific features of brain development regulatory programs

**Figure 10:**
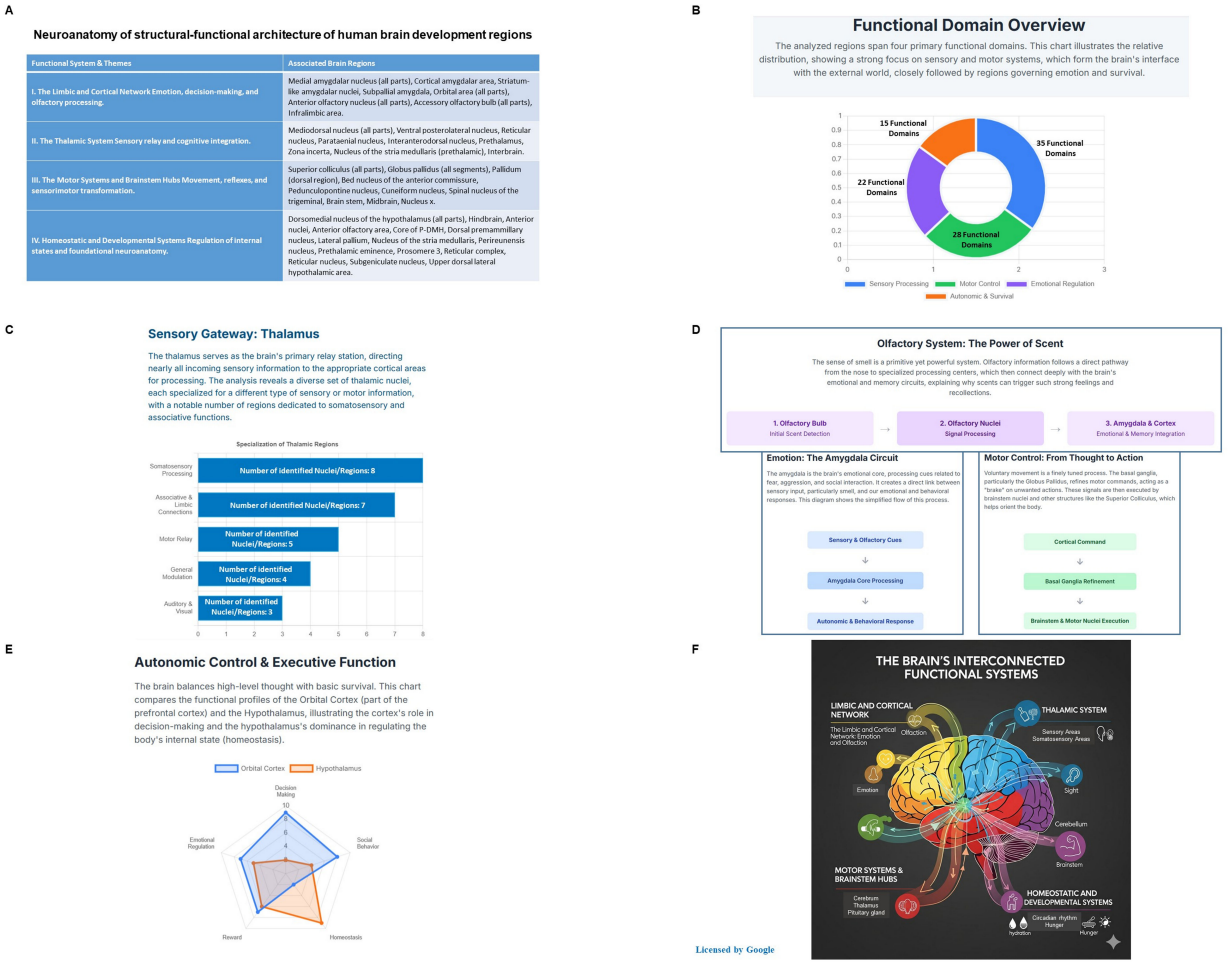
Infographic overview of 100 human brain regions identified by the gene set enrichment analysis (GSEA) of the 37 human-specific TFs differentially bound to human-specific brain development regulatory regions (BDRRs) vis-à-vis chimpanzee-specific BDRRs. The identified 100 human brain regions are presented as the conceptual flow diagram of the interconnected neural networks. The infographic is composed of interconnected diagrams to reflect the complexity of human brain’s neural networks. A high-level integrative overview has been conceived to describe how major functional neuroanatomical hubs are interconnected: (i) The limbic and olfactory hub receives chemosensory inputs and processes emotion and innate behavior. It connects to the hypothalamic hub. (ii) The Orbitofrontal Cortex (OFC) processes reward, decision-making, and top-down cognitive control. It has a reciprocal link with the thalamic hub. (iii) The thalamic hub acts as a gatekeeper and relay station for sensory and motor information. It is centrally positioned, with connections to all other major hubs. (iv) Motor and brainstem hubs executes movement, controls reflexes, and regulates sensory-motor Integration and vital life functions. It connects to the somato-sensory pathways. (v) Hypothalamic and homeostatic hub regulates internal states like feeding, stress, and body temperature. It has a direct link to the limbic hub and sends outputs to the rest of the body. **(A)** presents a tabular summary of the structural-functional architecture of identified human-biased brain development regions linking four human-specific functional brain systems to associated neuroanatomical structures. **(B)** displays a donut chart dividing a total of 100 human-specific brain development functional domains into sensory processing (35 domains), motor control (28 domains), emotion (22 domains), and autonomic and survival functions (15 domains). **(C)** features a graphical summary of Thalamus sensory gateway functions within the context of human-specific brain development regions. It displayed as a bar chart showing thalamic nuclei numbers specialized for somatosensory processing (8 nuclei/regions), associative and limbic connections (7 nuclei/regions), motor relay (5 nuclei/regions), general modulation (4 nuclei/regions), and auditory and visual functions (3 nuclei/regions). **(D)** uses boxes and arrows to map human-specific brain development aspects of olfactory system pathways and functional links to amygdala circuits, cortical command centers, defined cerebral nuclei, brainstem, and motor nuclei execution. Collectively, the panel **(D)** visualization is highlighting powerful connections of sensory, emotional, and motor domains evolving during human-specific aspects of brain development. **(E)** shows a radar chart comparing the relative quantitative impacts of the orbital cortex and hypothalamus across five functional domains, including decision making, social behavior, homeostasis, reward system, and emotional regulation. Collectively, the panel **(E)** illustrates the cortex’s powerful role in decision-making processes and hypothalamus’s dominance in regulating the body’s homeostasis. **(F)** offers a labeled brain infographic illustration with colored anatomical regions indicating interconnected functional brain systems components of which are engaged by 37 human-specific initiator TFs bound in a species-specific manner to brain development regulatory regions (BDRRs). Sub-sets of these 37 initiator TFs are significantly enriched among highly-expressed genes in 100 neuroanatomical regions of human brain.

It was of interest to explore whether applying these concepts would be informative for elucidating human-specific patterns of brain development. The UniBind algorithm, in the robust setting of differential-enrichment analyses (Methods), was employed to identify all TFs manifesting significantly distinct enrichment profiles in binary comparisons of TFs bound to human-specific BDRRs vis-à-vis chimpanzee-specific BDRRs (**Supplementary Data Set S4**). Consistent with the observations reported above, 97.3% (36 of 37 records) and 99.6% (229 of 230 records) of TFs preferentially bound to human-specific and chimpanzee-specific BDRRs are constituents of the core panel of 716 initiator TFs (**Supplementary Data Set S4**). Next, differentially enriched panels of species-specific TFs were subjected to GSEA employing the Allen Human Brain Atlas database of upregulated genes to catalog the corresponding sets of neuroanatomically defined brain regions that harbor significantly enriched subsets of regulatory TF-coding genes among genes with significantly increased expression in specific brain regions (**Supplementary Data Set S4**). Based on these analyses, two sets of brain regions were identified that manifest significant enrichments of increased expression patterns of genes encoding species-specific brain development regulatory TFs: a human-specific set of 100 brain regions and a chimpanzee-specific set of 48 brain regions. Notably, only 3 brain regions are common between the human-specific set of 100 brain regions and the chimpanzee-specific set of 48 brain regions, suggesting that developmental trajectories of core neuroanatomical regions of human and chimpanzee brains are markedly different. To test this hypothesis, the AI assistant Google Gemini was requested to carry out expert-level analyses of developmental and structural–functional features of the human-specific set of 100 brain regions and the chimpanzee-specific set of 48 brain regions. Full text reports of these analyses are available in **Supplementary Data Set 4**. The executive summary and infographic visual summary of the findings of expert-level analyses of 100 human-specific brain regions are reported below and shown in **Figure 10**.

### Analysis of human-specific brain development regions

The list of 100 brain regions encompasses a wide range of structures across the central nervous system, from the cortex to the brainstem (**Figure 10**). Collectively, these regions are heavily involved in four primary functional domains: sensory processing, emotional regulation and social behavior, motor control, and basic autonomic and survival functions. Many of the listed sub-regions and layered structures underscore the brain’s highly specialized and hierarchical organization, where information is processed and refined at multiple levels.

### Amygdala, limbic system, and emotion

The amygdala is a critical hub for emotional processing, particularly for fear, aggression, and social behavior. The list contains several detailed sub-regions, highlighting the complexity of its function.

- Medial amygdalar nucleus, posterodorsal part: This region and its sublayers (a, b, and c) are key components of the accessory olfactory system. They are crucial for processing olfactory and pheromonal cues directly linked to social and reproductive behaviors.
- Cortical amygdalar area: This area processes olfactory information, directly linking smells to emotional and behavioral responses. Its medial zone and layer 3 are involved in this sensory-emotional integration.
- Posterior amygdalar nucleus: Involved in the autonomic and motor expressions of emotion.
- Striatum-like amygdalar nuclei: These regions bridge emotional processing and motor function, integrating emotional states with the basal ganglia to influence action.
- Bed nucleus of the anterior commissure and subgeniculate nucleus: These are parts of the extended amygdala, involved in anxiety, stress, and reward circuits.

### Thalamus and sensory relay

The thalamus serves as the brain’s primary relay station, directing most sensory and motor information to the cerebral cortex. The list includes several nuclei that highlight its role as a central hub.

- Mediodorsal nucleus (MD): This nucleus and its lateral part connect the prefrontal cortex with limbic structures. It plays a significant role in attention, memory, and high-level executive functions, particularly those with an emotional component.
- Ventral posterolateral nucleus of the thalamus, parvicellular part: This is a key somatosensory relay. It transmits information about touch, pressure, and proprioception from the body to the primary somatosensory cortex.
- Reticular nucleus of the thalamus (reticular complex): This nucleus acts as a “gatekeeper” for the thalamus. It modulates the flow of information, helping to regulate states of consciousness, attention, and sleep.
- Interanterodorsal nucleus of the thalamus and parataenial nucleus: These are parts of the anterior thalamic nuclei, which are crucial for spatial memory and navigation.
- The analysis emphasizes the functions of the thalamus as the brain’s primary relay station, directing nearly all incoming sensory information to the appropriate cortical areas for processing. It reveals a diverse set of thalamic nuclei, each specialized for a different type of sensory or motor information, with a notable number of regions dedicated to somatosensory and associative functions.

### Motor control and basal ganglia

Motor control is a distributed process involving the cortex, basal ganglia, and brainstem. Several listed regions are integral to this system.

- Globus pallidus (external and internal segments): Core components of the basal ganglia, the globus pallidus refines and regulates voluntary movements by acting as a brake on unwanted motions. The internal segment sends the final output signal from the basal ganglia.
- Superior colliculus, motor-related: This region is involved in orienting movements, particularly those of the eyes, head, and body, in response to visual, auditory, and somatosensory stimuli. Its deep gray and deep white layers are key to this function.
- Pedunculopontine nucleus: Located in the brainstem, this nucleus is involved in initiating locomotion and regulating posture and gait.
- Zona incerta: A subthalamic nucleus with widespread connections to motor and limbic systems, involved in movement and arousal.

### Olfactory systems

The processing of smell is a central theme, with many regions dedicated to this function.

- Anterior olfactory nucleus: Receives input from the olfactory bulb and refines the olfactory signal before it is sent to other brain regions. Its dorsal and external parts, and layer 1, are involved in this initial processing.
- Accessory olfactory bulb (AOB): A specialized olfactory structure for processing pheromones. Its outer plexiform layer and intermediate stratum are involved in initial signal processing before information is relayed to the medial amygdala.

### Hypothalamus and autonomic regulation

The hypothalamus is a vital structure for maintaining homeostasis and orchestrating basic survival behaviors.

- Dorsomedial nucleus of the hypothalamus: This list includes the ventral part and core of this nucleus, which is involved in regulating feeding, body temperature, blood pressure, and circadian rhythms.
- Dorsal premammillary nucleus and upper dorsal lateral hypothalamic area: These regions are part of the feeding and aggression circuits of the hypothalamus.
- Peduncular part of dorsomedial hypothalamic nucleus: Also involved in integrating autonomic and behavioral responses.

### Cortex and executive function

The cortex is the center for higher-order cognitive functions.

Orbital area: A part of the prefrontal cortex, the orbital area is critical for decision-making, social behavior, and evaluating the emotional significance of stimuli. Its ventrolateral part and various layers are involved in these complex processes.

Infralimbic area, layer 2: A part of the medial prefrontal cortex involved in fear extinction and emotional regulation.

### Brainstem and foundational functions

The brainstem is the most evolutionarily ancient part of the brain and controls many fundamental functions.

- Cuneiform nucleus: Involved in motor control, particularly the coordination of gait.
- Spinal nucleus of the trigeminal: Receives sensory information from the face and head, including pain, temperature, and touch.
- Pretectal region: Involved in visual reflexes, such as the pupillary light reflex.
- Midbrain reticular nucleus: Part of the reticular formation, which is crucial for regulating consciousness, sleep-wake cycles, and arousal.

### Summary of the key findings

The analysis of diverse brain regions reveals a highly integrated and functionally specialized neuroanatomical architecture. Rather than operating in isolation, the brain’s systems engage in dynamic, reciprocal communication that orchestrates complex behaviors and maintains homeostasis. For instance, the limbic and cortical network, anchored by the amygdala and orbitofrontal cortex, links chemosensory and emotional states to innate and learned behaviors. The Thalamic System, far from being a passive relay, acts as a dynamic gatekeeper; nuclei like the MD actively partner with the cortex to facilitate executive functions and cognitive flexibility. Similarly, the motor systems and brainstem hubs, including the basal ganglia and superior colliculus, provide a sophisticated infrastructure for both deliberate and reflexive actions, governed by a push-pull mechanism that prevents chaotic movement. The most profound understanding gained from this synthesis is the direct and often subtle connections between these systems. The limbic system’s emotional decisions are intimately tied to the basal ganglia’s motor planning and the hypothalamus’s homeostatic outputs. The discovery of a direct circuit from the medial amygdala to the liver, routed through the hypothalamus, offers a new paradigm for understanding the physiological consequences of psychological stress and opens a new avenue for research into stress-related diseases. The analysis also highlights that an understanding of brain function is incomplete without a focus on the microcircuit and laminar levels, where the specific properties of individual neurons define the capabilities of entire systems. This report aligns with a foundational principle of neuroanatomy: the brain’s complexity emerges from the elegant and efficient organization of highly specialized, interconnected neural circuits. While many details about individual sub-layers and developmental strata remain to be fully elucidated, the established framework provides a clear direction for future inquiry, underscoring the continuously evolving nature of our understanding of the brain.

### Comparative analyses of modern human vis-à-vis chimpanzee brain development trajectories highlight some common features and major differences of potential functional significance

In the context of brain development, a comparison between species-specific brain development trajectories may highlight evolutionary differences between Chimpanzee and Modern Human brains, as noted in analyses of the top 25 differentially enriched TFs bound to human-specific BDRRs (Glinsky, 2026; **Supplementary Data Set S5**). Chimpanzee CNS development-related brain regions are primarily associated with innate, foundational functions for survival. In contrast, Modern Human CNS development-associated species-specific brain regions emphasize complex, higher-order cognitive abilities linked to sensory information acquisition, processing, and translation into diverse motor functions (Glinsky, 2026).

### Common features reflecting a common evolutionary heritage

Despite their apparent differences, both sets of species-specific brain regions are fundamental for survival and adaptation, reflecting a shared evolutionary heritage.

Foundation for survival: Both sets of regions include core structures for basic survival and homeostatic regulation. For chimpanzees (Report 1), this includes the cerebellar system for motor control and the limbic system for spatial memory. For humans (Report 2), this involves the hypothalamus and brainstem nuclei for vital functions like heart rate and breathing. This overlap underscores that a stable homeostatic and motor foundation is crucial for the development of both species.

Laminar specialization: Both analyses highlight the importance of layered brain structures (laminae). This suggests that the organization of neural circuits into specialized layers is a conserved feature of primate brain development, allowing for the segregation of input, output, and intrinsic processing within each region.

### Major species-specific differences revealed by comparative analyses of modern human-specific versus chimpanzee-specific brain development regions

These analyses indicate that the most significant differences lie in the developmental priorities of each species, leading to functional specialization.

### Distinct focus on innate behavior versus learned behavior

Analysis of chimpanzee development regions documents an emphasis on the visual system and motor control. The numerous visual areas (primary, posterolateral, anterolateral) and cerebellar regions (Crus 1, Crus 2, flocculus) suggest a developmental priority for processing complex visual stimuli and executing skilled, rapid movements. This is critical for navigating a natural environment, foraging, and social interactions that rely on immediate sensory-motor responses.

Analysis of modern human brain development regions revealed an emphasis on higher-order cognition and emotional regulation. Regions like the mediodorsal thalamus, orbitofrontal cortex, and superior colliculus are associated with abstract thinking, executive function, and rapid sensory-motor transformation. These regions are crucial for complex planning, language, and cultural learning—abilities that distinguish humans.

### Distinct features of thalamic and amygdalar specialization

Analysis of chimpanzee brain development regions indicated that thalamic regions are not as prominent, and the amygdala’s role is captured in the context of the global impact of the amygdalar system. Analysis of modern human brain development regions gives special attention to the thalamus as a “dynamic gatekeeper” for cognitive control and the amygdala for its role in integrating chemosignals for social and emotional responses. This suggests a more sophisticated developmental trajectory for social and cognitive information processing in humans. Collectively, the results of the present analyses highlight major distinctions between the evolutionary trajectories of brain development in modern humans and chimpanzees. Chimpanzee brain development regions can be seen as representing the foundational neuroanatomy shared by primates, optimized for survival in a “less abstract” world. In contrast, modern human brain development regions appear to reflect derived traits of human-specific brain development, where ancestral circuits have been adopted, refined, and expanded to support uniquely human cognitive abilities like complex social behavior, abstract thought, and the creation and use of increasingly sophisticated tools. Within this developmental context, it is important to underscore the observations reported in this contribution, which document HAP-associated mechanisms of human brain development and functions. These mechanisms constitute the apparently congruent regulations of gene expression developmental patterns across hundreds of neuroanatomical structures and the creation of functional synapses. This is enabled by the coordinated actions of the HAP PPI cascades operating in two concurrent modes: the TF–TF PPI chain reactions enabling neurogenesis and the HUB-PPI chain reactions facilitating synaptogenesis.

### Assessments of regulatory impacts on human-specific and chimpanzee-specific brain development regions by initiator transcription factors bound to GREs created by distinct mechanisms of primate regulatory DNA evolution

**Figure 11:**
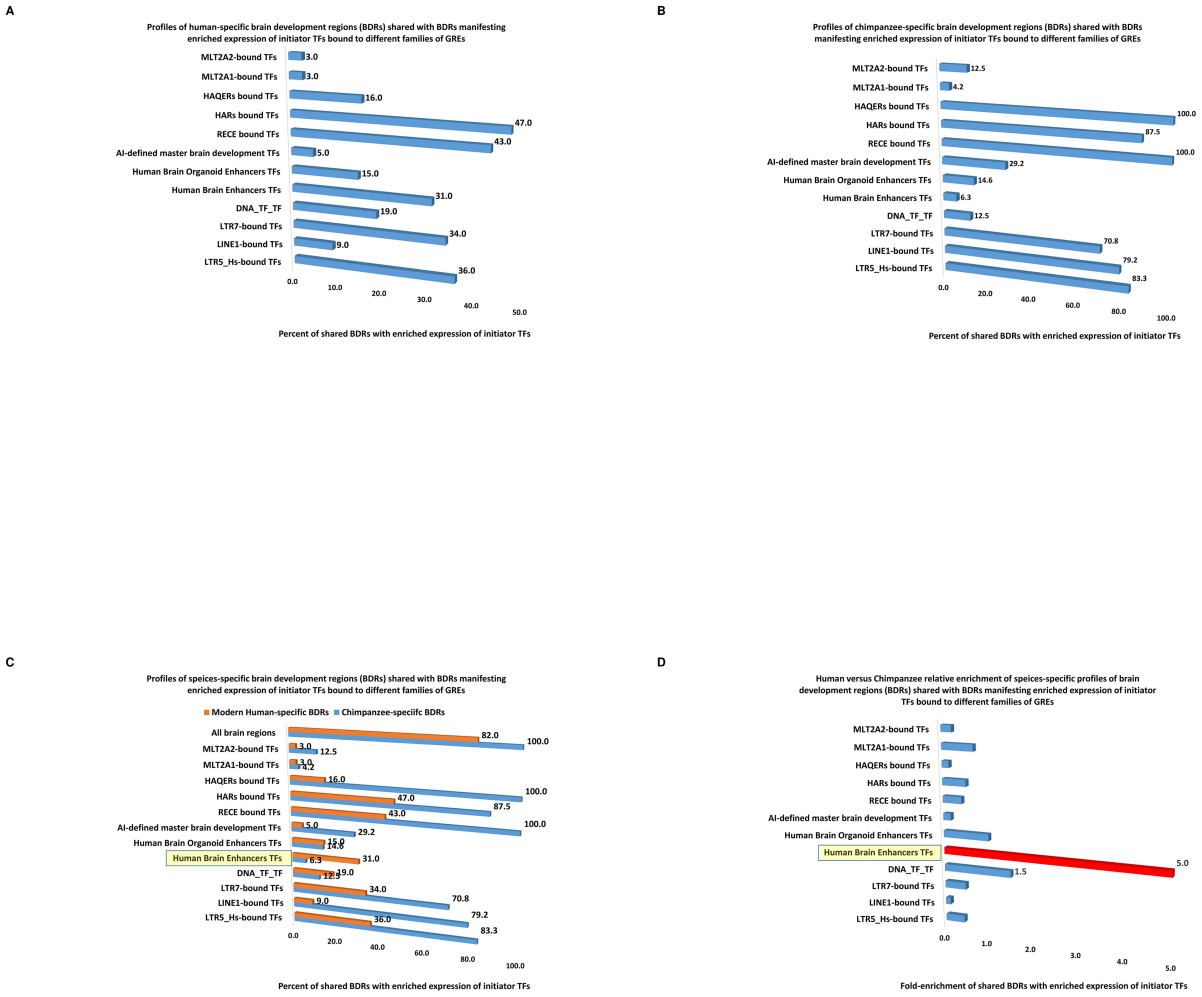
Comparisons of regulatory impacts on human-specific and chimpanzee-specific brain development regions of initiator transcription factors (TFs) bound to genomic regulatory elements (GREs) created by distinct mechanisms of regulatory DNA evolution. Panels of brain regions associated by gene set enrichment analysis (GSEA) with regulatory effects of TFs bound to specific families of GREs were compared to 100 human-specific brain development regions (BDRs) and 48 chimpanzee-specific BDRs to determine the fractions of species-specific BDRs overlapping with brain regions significantly enriched for TFs bound to a given GRE family. **(A)** Profiles of human-specific brain development regions (BDRs) shared with BDRs manifesting enriched expression of initiator TFs bound to different families of genomic regulatory elements (GREs). **(B)** Profiles of chimpanzee-specific brain development regions (BDRs) shared with BDRs manifesting enriched expression of initiator TFs bound to different families of GREs. **(C)** Combined profiles of human-specific and chimpanzee-specific brain development regions (BDRs) shared with BDRs manifesting enriched expression of initiator TFs bound to different families of GREs, and cumulative values of all overlapping BDRs reaching 82% of human-specific BDRs and 100% of chimpanzee-specific BDRs. **(D)** Relative regulatory impacts of different GRE families on human-specific and chimpanzee-specific brain development regions (BDRs) reported as the ratios of inferred regulatory effects on human-specific BDRs versus inferred regulatory effects on chimpanzee-specific BDRs. Note the apparent preferential regulatory effects of initiator TFs bound to human brain enhancers on human-specific BDRs.

Human-specific and chimpanzee-specific brain development regions (BDRs) were identified based on the significant enrichment (adjusted p-value < 0.05) of subsets of TFs bound to corresponding species-specific brain development regulatory regions (BDRRs). Since BDRRs were identified using the ATAC-seq method, the exact molecular identities of GREs exerting regulatory effects on brain development remain unknown. Therefore, it was of interest to investigate whether the distinct families of GREs analyzed in this contribution may have contributed to the emergence of species-specific patterns of brain development. To accomplish this, sets of brain regions associated with specific families of GREs by GSEA were compared to 100 human-specific BDRs and 48 chimpanzee-specific BDRs to determine what fractions, if any, of species-specific BDRs overlap with brain regions significantly enriched for TFs bound to a given GRE family. These analyses include GREs encoded by different families of human embryo regulatory LTRs (LTR5_Hs; LTR7; MLT2A1; MLT2A2); LINE1, which is transpositionally active in the human genome; two experimentally validated brain development GREs of different evolutionary origins, including human brain primary cells active enhancers (hBrain enhancers) and human brain organoids active enhancers (hBrain organoids enhancers); AI-defined brain development TFs; rapidly evolving conserved elements (RECE), representing highly conserved GREs with accelerated mutation acquisition during primate evolution, coincident with the beginning of brain size expansion on the primate lineage; as well as highly conserved GREs with accelerated mutation acquisition on the human lineage (human accelerated regions, HARs; human ancestor quickly evolved regions, HAQERs); and a set of 103 initiator TFs defined by high-confidence DNA**–**TF–TF interactions *in vivo*. Human-specific and chimpanzee-specific brain development regulatory regions (hsBDRRs and csBDRRs) were excluded from these analyses because the panels of 100 human-specific brain development regions and 48 chimpanzee-specific BDRs were defined based on the enrichment patterns of TFs bound to these two sets of BDRRs (Methods).Results of these analytical experiments demonstrate that subsets of initiator TFs bound to all GREs analyzed in this study appear significantly enriched among human-specific BDRs (**Figure 11A**) and chimpanzee-specific BDRs (**Figure 11B**). These observations support the hypothesis that multiple distinct GRE families may have contributed to regulatory networks shaping species-specific development of brain regions. However, the magnitudes of the potential regulatory impacts of distinct GRE families appear markedly different. Profiles of human-specific brain development regions (BDRs) shared with BDRs manifesting enriched expression of initiator TFs bound to different families of GREs demonstrate that development of only 3% of human-specific BDRs may be affected by TFs bound to either MLT2A1 or MLT2A2 loci (**Figure 11A**). In contrast, TFs bound by RECE or HARs appear linked to inferred regulatory impacts on 43% and 47% of human-specific BDRs (**Figure 11A**). Similarly, initiator TFs bound to human brain enhancers, LTR7 loci, and LTR5_Hs loci are associated with inferred regulatory effects on 31%, 34%, and 36% of human-specific BDRs, respectively (**Figure 11A**). Overall, 82 of 100 human-specific BDRs identified in this study (**Figure 11C**) are linked to potential regulatory impacts of initiator TFs bound to the diverse families of GREs analyzed herein, which are markedly distinct in their evolutionary origins and emerged millions of years apart in primate evolution. Profiles of chimpanzee-specific brain development regions (BDRs) that share enriched expression of initiator TFs bound to different families of GREs suggest that the development of chimpanzee-specific BDRs may have been influenced by multiple distinct GRE families (**Figure 11B**). Notably, initiator TFs bound to several GRE families (LTR7, LINE1, LTR5_Hs, and HARs) appear linked to inferred regulatory impacts on 70.8% to 87.5% of chimpanzee-specific BDRs (**Figure 11B**). Furthermore, initiator TFs bound to either RECE or HAQERs appear engaged in regulatory circuitry contributing to the development of all 48 chimpanzee-specific BDRs (**Figure 11B**). These observations indicate that multiple GRE families are involved in multilayered and seemingly redundant regulatory impacts on brain regions defined as chimpanzee-specific BDRs. This feature of multiple GRE families contributing to the governance of human brain development seems particularly important given the crucial role of several neuroanatomical structures, defined as components of chimpanzee-specific BDRs, in species survival and adaptation. Chimpanzee-specific BDRs include numerous visual areas (primary, posterolateral, anterolateral) and cerebellar regions (Crus 1, Crus 2, flocculus), suggesting a developmental focus on processing complex visual stimuli and executing skilled, rapid movements. The functionalities of these neuroanatomical structures are essential for navigating a natural environment, foraging, and social interactions supported by immediate sensory-motor responses. Chimpanzee-specific BDRs reflect an emphasis on the visual system and motor control, fundamental for survival and adaptation, and reflecting a shared evolutionary heritage of primates. A side-by-side visualization of the relative contributions of distinct GRE families to human-specific and chimpanzee-specific BDRs demonstrated that TFs bound to human brain enhancers exhibit higher levels of enrichment among human-specific BDRs compared to chimpanzee-specific BDRs (**Figures 11C, D**). In contrast, TFs bound to HAQERs, RECE, HARs, LTR7, LINE1, and LTR5_Hs loci show relatively higher enrichment levels among chimpanzee-specific BDRs (**Figures 11C, D**). Significantly, regulatory controls potentially exerted by human-specific GREs (HAQERs and HARs) on cerebellar regions (Crus 1, Crus 2, cerebellar hemisphere) could be particularly intriguing, considering the reported important roles of cerebellar components in human language networks (Casto et al., 2026). One cerebellar region spanning Crus 1/2/VIIb seems especially important because it is highly selective for language compared to non-linguistic inputs and because this cerebellar region mirrors the neocortical language network (Casto et al., 2026).Collectively, these findings strongly argue that a compendium of GREs contributing to the transcriptional regulation of human-specific BDRs was continually acquired and retained during millions of years of primate evolution. This ensured the proper functions of the chromosome-naive, multi-layered GRNs governing human brain development, as well as the maintenance of the structural–functional integrity of hundreds of neuroanatomical structures of the mature human brain. Congruently, multiple families of GREs are engaged in a multilayered, redundant regulatory control of brain regions reflecting the primates’ common evolutionary origin (defined as constituents of chimpanzee-specific BDRs), including multiple neuroanatomical structures essential for species adaptation and survival.

### Experimental validation pipelines for exploring transcriptional connectomes of human brain regions

Defining molecular features of the GSEA of initiator TFs, utilizing the Allen Brain Atlas database of upregulated genes, revealed that subsets of initiator TFs are significantly enriched among genes with increased expression levels across hundreds of human brain regions. Given the ready availability of gene expression atlases for thousands of human brain regions, these observations suggest a feasible experimental program aimed at exploring the transcriptional connectomes of individual neuroanatomical structures. Within this program, the following analytical and experimental steps should be executed for each brain region of interest:

1. Define the region-specific expression signature of upregulated genes using existing brain atlases of region-specific gene expression profiling.
2. Intersect the constituents of the brain region-specific expression signature of upregulated genes with sets of initiator TFs, TF–TF PPI cascade constituents, or HUB protein PPI cascade constituents to identify the core constituents of the HAP of EMCs.
3. Predict a small subset of initiator TFs that represent the top-tier candidate region-specifying or region-maintaining regulators.
4. Validate the functional significance of these initiator TFs using targeted genetic perturbation approaches (e.g., CRISPRi/a-based technologies, degrons, TF knockdown, haploinsufficiency models) in relevant cell types or organoid models. Validation will involve measuring (i) erosion of region-specific genetic markers; (ii) alterations in structural and/or functional connectivity phenotypes; and (iii) shifts in synaptic gene expression potentially associated with an altered balance of GABAergic and glutamatergic firing.

**Figure 12:**
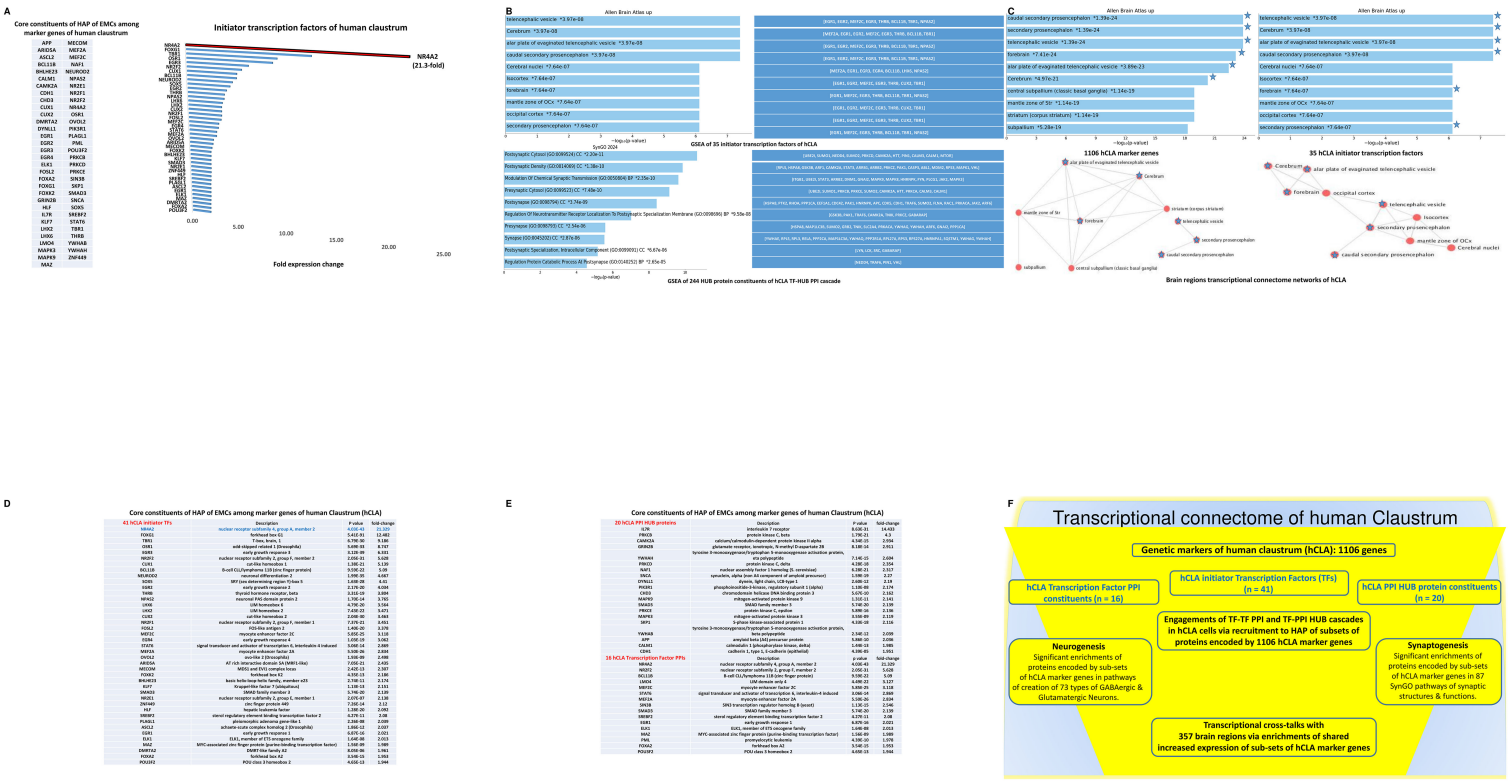
Shedding light on the transcriptional connectome of the human claustrum. Visual summary of the exploration of the feasibility of the hybrid assembly pathway (HAP) concept in a series of analytical experiments designed to define the transcriptional connectome of the human claustrum (see text and **Supplementary Data Set 6** for details of experimental protocols and the extended reporting of the results). **(A)** shows the list of genes comprising the core constituents of HAP of endogenous multiprotein complexes (EMCs) among marker genes of the human claustrum and the bar graphs reporting the 41 initiator transcription factors (TFs) of the human claustrum sorted in a descending order of the fold expression changes defined as the ratios of the expression values in the human claustrum to the expression values in the human brain. **(B)** top rows depict the results of the gene set enrichment analysis (GSEA) of human claustrum initiator TFs employing the Allen Brain Atlas database of upregulated genes (the top-scoring 10 significantly enriched records are shown). **(B)** bottom rows depict the results of GSEA of the 244 proteins of PPI-HUB protein cascade constituents of the protein–protein interaction (PPI) networks of human claustrum initiator TFs employing the synaptic GO database (the SynGO 2024 database) of genes implicated in the development and function of synapses (the top-scoring 10 significantly enriched records are shown). **(C)** shows a side-by-side comparison of the results of GSEA of 1,106 human claustrum marker genes (left) and 35 human claustrum initiator TFs (right) employing the Allen Brain Atlas database of upregulated genes (the top-scoring 10 significantly enriched records are reported). Visualization of brain regions transcriptional connectome networks of the human claustrum is depicted in the bottom two figures. Stars denote the identical records of significantly enriched brain regions identified by the GSEA of either 1,106 human claustrum marker genes (left) or 35 initiator TFs of the human claustrum. **(D)** and **(E)** report the detailed records of the analyses on the identification and characterization of the core constituents of the HAP of EMCs among genetic markers of the human claustrum. The records are shown pertaining to 41 initiator TFs of the human claustrum **(D)**; 20 proteins of the PPI-HUB protein cascade and 16 TFs of the TF–TF PPI cascade of the human claustrum **(E)**. **(F)** depicts the visual summary of the key elements of the analytical experiments on the identification and characterization of the transcriptional connectome of the human claustrum. Overall, the transcriptional connectome of the human claustrum reflects the transcriptional crosstalks of the human claustrum with 357 brain regions, manifesting the significant enrichments of the subsets of the human claustrum marker genes among the brain region-specific sets of upregulated genes (see text and the **Supplementary Data Set 6** for details of experimental protocols and the extended reporting of the results).

**Figure 13:**
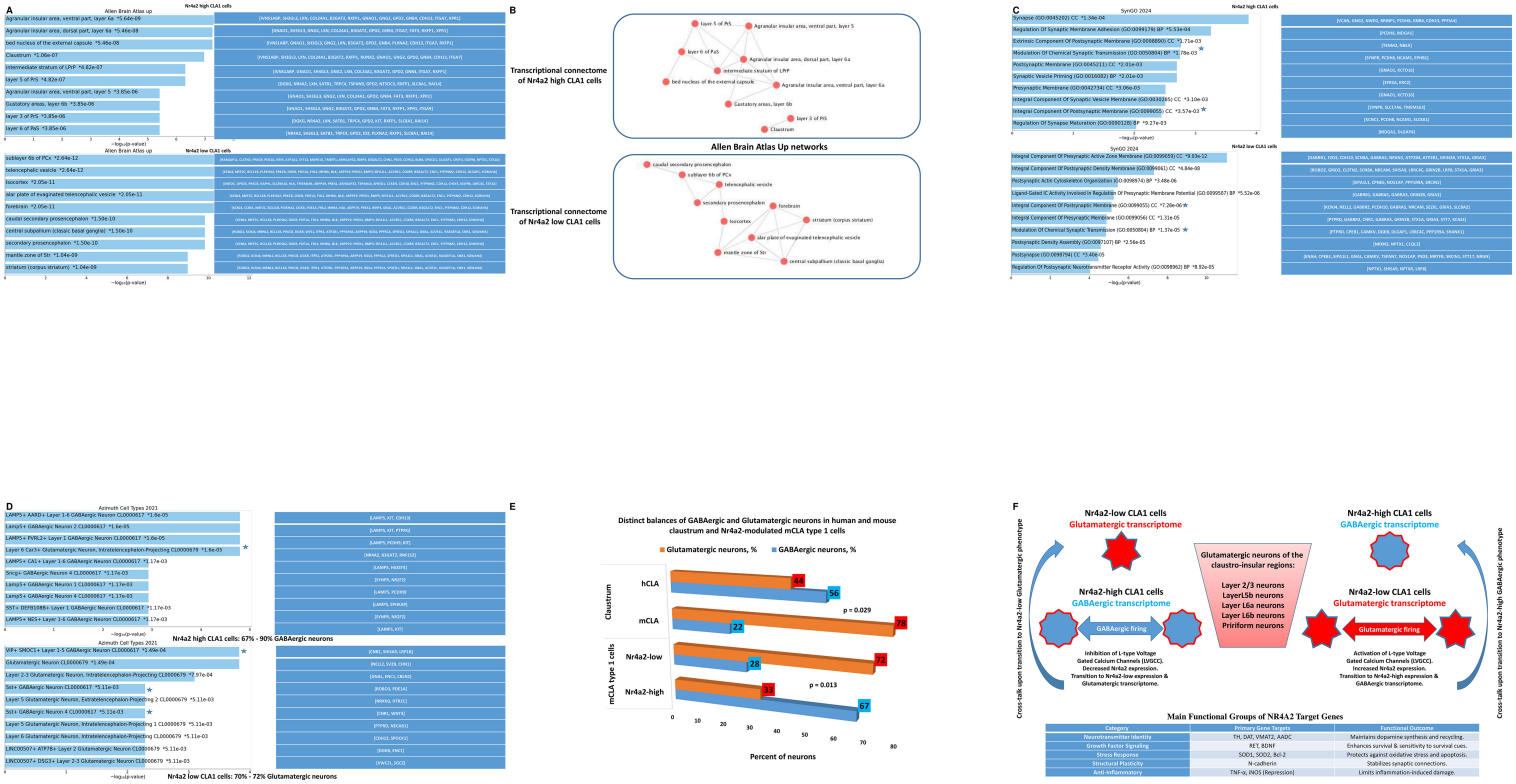
Comparative analyses of transcriptional connectomes of two types of mouse claustrum 1 cells by interrogating the 220 gene signature of Nr4a2-low CLA1 cells versus the 104 gene signature of Nr4a2-high CLA1 cells. Details of the experimental protocols and the extended documentation of the results are reported in the text and in **Supplementary Data Set 7**. **(A)** depicts a side-by-side comparison of the results of the gene set enrichment analysis (GSEA) of the 104 gene signature of Nr4a2-high cells (top) and the 220 gene signature of Nr4a2-low cells (bottom) employing the Allen Brain Atlas database of upregulated genes (the top 10 significantly enriched records of brain regions are reported). **(B)** shows a side-by-side comparison of transcriptional connectome networks of Nr4a2-high CLA1 cells (top) and Nr4a2-low cells (bottom) defined by the GSEA employing the Allen Brain Atlas database of upregulated genes. **(C)** presents a side-by-side comparison of results of GSEA of the 104 gene signature of Nr4a2-high CLA1 cells (top) and the 220 gene signature of Nr4a2-low CLA1 cells (bottom) employing the synaptic GO database (the SynGO 2024 database) of genes implicated in development and function of synapses (the top-scoring 10 significantly enriched records are shown). Stars denote the identical significantly enriched records of the SynGO 2024 database identified by the independent analyses of the two populations of the CLA1 cells. **(D)** reports a side-by-side comparison of results of GSEA of the 104 gene signature of Nr4a2-high CLA1 cells (top) and the 220 gene signature of Nr4a2-low CLA1 cells (bottom) employing the Azimuth Cell types 2021 database of cell type-specific genetic markers (the top 10 significantly enriched records of brain regions are reported). A single star in the top figures denotes the only glutamatergic neuron identified among the top 10 significantly enriched records of cell types having overlapping marker genes with Nr4a2-high CLA1 cells. The remaining 9 of 10 significantly enriched records are the various types of the GABAergic neurons. Three stars in the bottom figures mark the GABAergic neurons identified among the top 10 significantly enriched records of cell types having overlapping marker genes with Nr4a2-low CLA1 cells. The remaining 7 of 10 significantly enriched records are the various types of the glutamatergic neurons. **(E)** reports the patterns of distinct balances of GABAergic and glutamatergic neurons in human claustrum compared to mouse claustrum and 2 types of mouse CLA1 cells. Details of the experimental protocols and the extended documentation of the results are reported in the text and in **Supplementary Data Set 7**. Significantly enriched records of the GSEA employing the Azimuth Cell types 2021 database of cell type-specific genetic markers were included in quantitative analyses requiring at least two genes per record. Reported p-values were estimated using the one-tail Chi-square test. **(F)** summarizes observations characterizing molecular mechanisms of the autonomously recurring transition cycles of Claustrum neurons inferred from transcriptome analyses of Nr4a2-low and Nr4a2-high CLA1 cells. Note that Nr4a2-low CLA1 cells exhibit a glutamatergic transcriptome, while Nr4a2-high CLA1 cells manifest a GABAergic transcriptome. Nr4a2-high CLA1 cells through GABAergic firing acquire the inhibited state of L-type Voltage Gated Calcium Channels (LVGCC) and decreased Nr4a2 expression, thus transitioning to the state of Nr4a2-low expression and a glutamatergic transcriptome. Conversely, Nr4a2-low CLA1 cells through glutamatergic firing acquire the activated state of the LVGCC and increased Nr4a2 expression, thus completing the reverse transition to the state of Nr4a2-high CLA1 cells manifesting a GABAergic transcriptome. Listing of main functional groups of NR4A2 down-stream target genes in human cells illustrates the putative biological significance of the phenotypic distinctions between neurons manifesting different expression levels of the *NR4A2* gene.

The feasibility of this approach was explored in a series of analytical experiments designed to define the transcriptional connectome of the human claustrum. Initially, the top 2,000 transcripts with significantly increased expression levels in the human claustrum, compared to other human brain regions, were retrieved from the web-accessible portal of the Allen Brain Atlas database (**Supplementary Data Set S6**; Microarray data: Allen Brain Atlas: Human Brain). Processing this dataset to remove redundant records and non-RefSeq entries resulted in the identification of 1106 genes, herein defined as genetic markers of the human claustrum (**Figure 12**; **Supplementary Data Set S6**). In agreement with the HAP model, the panel of genetic markers for the human claustrum includes 41 initiator TFs, 16 genes encoding TFs of the TF–TF PPI cascade branch, and 21 genes encoding HUB proteins of the TF-HUB proteins PPI cascade branch (**Figure 12**; **Supplementary Data Set 6**). These subsets of human claustrum marker genes can be defined as the core constituents of the HAP of EMCs in the human claustrum (**Figure 12**). Next, GSEA was performed on both the human claustrum HAP core constituents and all human claustrum marker genes, employing multiple genomic and proteomic databases. In accord with HAP model expectations, a majority of the human claustrum initiator TFs (35 of 41 TFs; 85%) showed significant enrichments among genes upregulated in human brain regions (**Figure 12**; **Supplementary Data Set 6**; The Allen Brain Atlas database of upregulated genes). Furthermore, constituents of the TF-HUB proteins PPI cascade are significantly enriched among genes implicated in synaptogenesis and the structural–functional components of mature synapses (**Figure 12**; **Supplementary Data Set S6**; The SynGo 2024 database). Side-by-side comparisons of GSEA records for human claustrum initiator TFs versus a panel of human claustrum marker genes revealed that only the initiator TFs exhibited patterns of significant enrichment for constituents of the TF–TF PPI cascade. No significant enrichments were detected for human claustrum marker genes, whereas 42 TFs and 198 TFs were engaged with claustrum initiator TFs at TF–TF PPI levels 1 and 2, respectively. These findings suggest that human claustrum initiator TFs may function as the predominant driver of the initiation steps of the TF–TF PPI cascade, presumably operating in the human claustrum. In contrast, proteins encoded by human claustrum marker genes may serve as protein constituents for subsequent steps in the assembly of EMCs. In agreement with this hypothesis, panels of top-scoring brain regions identified by independent GSEA of either human claustrum initiator TFs or human claustrum marker genes appeared markedly similar, demonstrating a 60% overlap of identified neuroanatomical structures and visual resemblance of inferred transcriptional connectome networks (**Figure 12**; **Supplementary Data Set S6**). Overall, the observations from these analyses support the hypothesis of a transcriptional connectome of the human claustrum, reflecting apparent transcriptional crosstalk between the claustrum and 357 brain regions. This crosstalk is inferred from statistically significant enrichments (adjusted p-value < 0.05) of shared increased expression of subsets of human claustrum marker genes (**Figure 12**; **Supplementary Data Set 6**).It was also of interest to determine whether human claustrum initiator TFs exhibit increased expression in brain regions experimentally documented to have bilateral and reciprocal connections with the claustrum, based on experiments using anterograde viral tracer injections in mice (Wang et al., 2017). These analyses demonstrated that 27 of 34 (79.4%) brain regions with ipsilateral structural connections to the claustrum showed increased expression of human claustrum initiator TFs (**Supplementary Data Set 6**). Similarly, increased expression of human claustrum initiator TFs was identified in 8 of 9 (88.9%) brain regions with contralateral structural connections to the claustrum (**Supplementary Data Set 6**). Lei et al. (2025) reported a single-cell spatial transcriptome atlas and whole-brain connectivity profiles of the macaque claustrum. Therefore, it was relevant to identify human claustrum initiator TFs expressed in entorhinal, visual, insular, and somatosensory brain areas, which were identified as neuroanatomical regions of the macaque claustrum connectome (**Supplementary Data Set S6**). These findings imply a potential mechanistic convergence of transcriptional and structural connectomes of the claustrum with distant brain regions and suggest that initiator TFs may play a role in the development of connectivity patterns in human brain regions. The hypothesis that specific initiator TFs contribute to maintaining the transcriptional, structural, and functional identities of claustrum cells could be tested by targeted genetic manipulations of key initiator TF-coding genes in the claustrum. For example, selective targeting of *NR4A2* (**Figure 12**), a top-scoring gene highly expressed in the claustrum, would be a suitable approach. Investigations into the functions of the NR4A2 protein in the claustrum are particularly interesting because it is the only initiator TF identified as a common genetic marker in the mouse, macaque (Lei et al., 2025), and human claustrum (unpublished observations and **Supplementary Data Sets 6**, **7**). *Nr4a2* (Nurr1) expression in CLA neurons is characterized by an early developmental onset, with sustained high levels throughout development and in mature CLA neurons (Zetterström et al., 1996; Arimatsu et al., 2003; Fang et al., 2021; Fodoulian et al., 2025). A mouse model of *Nr4a2* haploinsufficiency was utilized to comprehensively characterize neuronal populations of the claustro-insular region, including the identification and characterization of Claustrum 1 type (CLA1) neurons (Fodoulian et al., 2025). In this model, only claustrum neurons exhibited changes in molecular identity and functional properties, exemplified by the activation of a transcription factor cascade and alterations in neuronal firing activity (Fodoulian et al., 2025). These experimental observations, therefore, highlight the key role of NR4A2, the top-scoring human claustrum initiator TF, in maintaining the transcriptional and functional identities of claustrum projection neurons (Fodoulian et al., 2025). This conclusion aligns with the proposed biological functions of initiator TFs in neurogenesis and the maintenance of neuronal identities in mature neurons. A model of *Nr4a2* haploinsufficiency enabled the identification of 324 genes whose expression is significantly affected in CLA1 cells with distinct levels of *Nr4a2* expression (Fodoulian et al., 2025; **Supplementary Data Set 7**). It was of interest to compare the patterns of transcriptional connectomes in CLA1 cells with relatively higher and lower *Nr4a2* expression, designated CLA1 Nr4a2-high and CLA1 Nr4a2-low cells, respectively (**Figure 13**). Consistent with experimentally documented changes in the neuronal identities of claustrum neurons with distinct levels of *Nr4a2* expression, the transcriptional connectomes of CLA1 Nr4a2-high and CLA1 Nr4a2-low cells are markedly different (**Figure 13**; **Supplementary Data Set 7**). Constituents of both CLA1 Nr4a2-high and CLA1 Nr4a2-low gene expression signatures are significantly enriched among genes implicated in various synaptogenesis pathways, indicating that both cell types may cooperate by contributing different sets of genes to the development and functions of synaptic networks in the claustro-insular brain region (**Figure 13**; **Supplementary Data Set 7**). Further supporting the CLA neuron subtype cooperation hypothesis, GSEA employing the Azimuth Cell Type 2021 database revealed that the transcriptomes of CLA1 Nr4a2-high and CLA1 Nr4a2-low neurons differ markedly in the expression of genetic markers for either GABAergic or glutamatergic neurons (**Figure 13**; **Supplementary Data Set 7**). Glutamatergic neurons constitute 70%–72% of neurons showing significant enrichment of gene subsets with increased expression in CLA1 Nr4a2-low cells. In contrast, GABAergic neurons comprise 67%–90% of neurons showing significant enrichment of gene subsets with increased expression in CLA1 Nr4a2-high cells. GSEA employing the Azimuth Cell Type 2021 database, applied to all 324 genes whose expression is modulated by *Nr4a2*in CLA1 neurons, demonstrated more balanced enrichment profiles of CLA1 transcriptomes, resulting in 42.5% and 57.5% of significantly enriched records for GABAergic and glutamatergic neurons, respectively (**Supplementary Data Set 7**).Collectively, these findings provide a foundation for a homeostatic model of transcriptional identity maintenance and survival of claustrum projection neurons (**Figure 13**; **Supplementary Data Set 7**). This model is further supported by the following observations: (i) Glutamatergic firing activates L-type voltage-gated calcium channels (LVGCC), which, in turn, increase *Nr4a2* expression. (ii) GABAergic firing tends to inhibit LVGCC and decrease *Nr4a2* expression. (iii) The claustro-insular region is populated by multiple types of glutamatergic neurons, including layer 2/3 neurons, layer 5b neurons, layer 6a neurons, layer 6b neurons, and piriform neurons. Notably, the principal genetic constituent of this model is the initiator TF NR4A2 (NURR1).

## Discussion

### Consensus regulatory features of DNA sequences derived from distinct families of transposable elements

This study reports the identification and characterization of a genome-wide, DNA-templated mechanism that initiates protein-protein interaction (PPI) cascades. The HAP model proposes that PPI cascade initiation operates continuously in real time, constrained primarily by protein availability rather than by the scarcity of DNA templates. Approximately 4.5 million transposable element (TE)-encoded loci in the human genome of every cell provide an effectively non-limiting reservoir of initiation templates, largely embedded within heterochromatic genomic contexts. Under these conditions, the DNA-templated initiation of hybrid assembly pathways bifurcates into two biologically congruent modes of PPI propagation: TF-TF cascades, enriched in transcriptional programs that define the structural–functional features of neuroanatomically distinct brain regions, and TF-HUB cascades, enriched in synaptogenesis and components of mature synaptic structures. Together, these bifurcated branches of PPI cascades offer a unified genomic framework of HAP activities, orchestrating the coordinated development and maintenance of brain-region identity alongside synaptic architecture and connectivity. DNA sequences derived from different TE families collectively occupy approximately 50% of the human genome, seeding over 4.5 million confidently mapped TE sequences (Hoyt et al., 2022). Consequently, evidence of common structural–functional features across these vastly diverse TE families, which comprise at least half of human genomes, may indicate the existence of previously unrecognized global genomic regulatory networks (GRNs). This perspective aligns well with the idea that TEs serve as powerful agents of evolutionary innovation by creating new GRNs and rewiring existing ones. A prime mechanistic example supporting this concept is the co-evolution of TEs and KRAB Zinc Finger proteins (ZFPs). In humans, almost all TE subfamilies are preferentially bound by at least one of approximately 400 KRAB-ZFPs, which primarily function to temper TE activities (Pontis et al., 2019; Rosspopoff et al., 2025), thereby facilitating the functional integration of TEs into GRNs. Because the human genome harbors approximately 4.5 million TE-encoded regulatory loci in every cell, the initiation of DNA-templated PPI cascades is unlikely to be limited by template availability. Instead, the primary constraint is the availability and compatibility of protein constituents, implying that bifurcated TF-TF and TF-HUB PPI cascades may operate continuously in real time across globally distributed heterochromatic initiation domains. Thus, TE-encoded sequences endow the genome with a persistent, chromosome-naive PPI initiation landscape upon which cellular state is projected via cell type-specific protein availability to exploit a global, massively parallel assembly engine. Investigations of approximately 10,000 DNA loci, encoded by remnants of LTR7/HERVH, LTR5_Hs/HERVK, MLT2A1/HERVL, and MLT2A2/HERVL retroviruses (collectively defined as human embryo regulatory LTRs; Glinsky, 2022; 2025; 2026), have revealed common regulatory features among their LTR elements. This is notable given their clearly distinguishable DNA sequences and millions of years of evolutionary separation. In contrast to human embryos, the majority of LTR/HERV-derived DNA sequences, particularly those encoded by human embryo regulatory LTRs, are epigenetically silenced in differentiated human cells. Only a relatively limited number of loci appear to be expressed in a site-specific manner within the human body (She et al., 2022). These observations support the concept of a developmental genetic blueprint for the creation of ubiquitous, genome-wide heterochromatin patterns that reproducibly emerge during the ontogenesis of tissues and organs in differentiated human cells (Glinsky, 2025). Recently, the identification and characterization of genome-wide regulatory networks have been reported, comprising quantitatively distinct panels of DNA sequences for transcription factor binding sites (TFBS) for 716 proteins (Glinsky, 2026). The sequences of these TFBS are encoded by DNA molecules derived from diverse TE families, including LT5_Hs/HERVK, LTR7/HERVH, MLT2A1, MLT2A2, SVA, LINE, and SINE/Alu. The evolutionarily conserved foundation of these GRNs appears to be assembled from arrays of approximately 770 TFBS (Glinsky, 2026). It has been observed that the majority of protein constituents of these GRNs (700 of 716; 98%) are defined by Gene Ontology (GO) features of sequence-specific double-stranded DNA binding (GO: 1990837), suggesting that these TFBS may retain their TF-binding properties within heterochromatin compartments. Continuing this line of investigation, observations reported in this contribution enabled the identification and characterization of genomic DNA templates for initiating chain reactions of protein dimerization, which create protein dimers that guide protein-protein interactions (PPI) during the assembly of EMCs. TE-derived loci harbor large nucleosome-free islands suitable for housing PPI initiation seeds and facilitating the initial steps of DNA template-guided assembly lines of EMCs. The distinctive features of the identified protein constituents of these DNA template-guided PPI assembly lines of EMCs are documented by records of significant enrichments (adjusted p-value < 0.05) in the following (i) thousands of endogenous macromolecular complexes reported in the NURSA Protein Complexes database (1,792 records), CORUM database (383 records), virus–host PPI P-HIPSTer 2020 database (6,607 records), and Jensen Compartments database (477 records) and (ii) an exceedingly broad spectrum of human common and rare diseases reported in the DisGeNET database (3,257 records), the Orphanet Augmented 2021 database (266 records), the OMIM Expanded database (181 records), and the Rare Diseases AutoRIF Gene Lists database (2,050 records).

### Diverse TE families seed chromosome-naive networks of TFBS, contributing to a genome-wide compendium of DNA sequences underlying the genetic cis-regulatory code

The genome-wide compendium of DNA sequences encoding binding sites for over 1,600 TFs, which interact with specific TFBS and each other, represents a key structural element of the global genomic cis-regulatory code (Kim and Wysocka, 2023; De Boer and Taipale, 2024; Xie et al., 2025). Given that the human genome contains more than 4.5 million individual sequences derived from various transposable element (TE) families, TE-encoded TFBS are significant contributors to the formation of this global genomic cis-regulatory code.A multi-step analytical approach was developed and implemented to catalog all TFs with potential TFBS within DNA sequences originating from distinct families of TEs domesticated in human genomes. To detect and quantify TFBS within genomic loci encoded by a specific TE subfamily, the Jaspar algorithm was used. This algorithm identified all TFBS located within 606 LTR5_Hs loci, which have been previously characterized as functionally active regulatory elements in human cells (Fuentes et al., 2018; Glinsky, 2022; 2025; 2026). LTR5_Hs loci are expected to show relatively minor divergence in TFBS profiles because LTR5_Hs/HERVK successfully infected and colonized primate germlines more recently compared to other HERVs with documented regulatory functions in human embryogenesis (Glinsky, 2022; 2025; 2026). The Jaspar algorithm’s output was processed to identify, at single-nucleotide resolution, all TFs with predicted TFBS within LTR5_Hs loci, and the number of TFBS for each TF was calculated (Glinsky, 2026). Overall, LTR5_Hs loci harbor 771 distinct TFBS for 716 individual TFs, including 65 paired TFBS features for 68 TFs, which comprise immediately adjacent TFBS for two different TFs. Since individual LTR5_Hs loci contain subsets of this composite set of 771 TFBS, this was designated a consensus ancestral set of TFBS encoded by LTR5_Hs loci (Glinsky, 2026). Gene set enrichment analyses (GSEA), performed using the ENRICHR bioinformatics platform, documented the exceptionally broad involvement of LTR5_Hs loci-residing TFs in various physiological phenotypes and pathological conditions of Homo sapiens (Glinsky, 2026). Notably, 700 of 716 (98%) of TFs with TFBS within LTR5_Hs loci belong to the single Gene Ontology classification category designated “Sequence-Specific Double-Stranded DNA Binding” (GO: 1990837). These observations suggest that the identified set of LTR5_Hs-residing TFs may constitute a sequence-specific double-stranded DNA binding pathway whose protein constituents appear to exert exceptionally broad regulatory impacts on the phenotypes of modern humans. It was of interest to expand this line of investigation to characterize the spectrum of TFBS encoded by other LTR families known to play a regulatory role during human embryogenesis, collectively defined as human embryo regulatory LTRs (Glinsky, 2022; 2025; 2026). To this end, all potential TFBS encoded by LTR7, MLT2A1, and MLT2A2 loci were identified using the Jaspar algorithm, and corresponding quantitative metrics were computed for each set of TFBS (Glinsky, 2026). Intriguingly, despite clearly discernible marked divergence of DNA sequences, all regulatory LTR elements analyzed herein appear to harbor nearly identical sets of TFBS. Since the introductions of different families of regulatory LTRs into primate germlines were separated by millions of years, these observations are consistent with the hypothesis of evolutionary conservation of these LTR-associated GRNs. Despite overall qualitative similarities in TFBS density profiles, human embryo regulatory LTRs exhibit notable quantitative differences in the number of TFBS for specific TFs (Glinsky, 2026). These differences translate into loci-specific compositions of TFs bound to specific TE-encoded regulatory sequences (Glinsky, 2026). Downstream analyses demonstrated that the sets of TFBS and panels of corresponding TFs constituting the Sequence-Specific Double-Stranded DNA Binding pathway of TFBS, seeded by human embryo regulatory LTRs, are (i) common for regulatory sequences encoded by other TE families, including SINE/Alu, LINE, HERVs, and SVA loci; (ii) bind DNA sequences at specific regulatory loci; and (iii) appear consistently engaged in genome-wide regulatory networks known to contribute to the governance of human and chimpanzee brain development and functions (Glinsky, 2026). Notably, Gene set enrichment analyses (GSEA) of the Allen Brain Atlas database of upregulated genes demonstrate that 534 of 716 TFs (76%) of this TE-seeded network are significantly enriched (adjusted p-value < 0.05) among genes whose increased expression distinguishes 1,358 human brain regions (**Supplementary Data Set S1**). These observations underscore that a hallmark feature of the biological significance of TE-seeded genome-wide TFBS networks and associated TFs is the regulation of central nervous system development and functions. In agreement with the hypothesis that TE-seeded TFBS are intrinsic components of the genome-wide cis-regulatory code, the present analyses demonstrate that 103 of 106 TFs (97%) engaged in high-confidence DNA**–**TF–TF interactions reported by Xie et al. (2025) are members of the consensus panel of 716 TFs having TE-seeded TFBS (**Supplementary Data Set S1**). Observations reported in this contribution indicate that TFs engaged in high-confidence DNA**–**TF–TF interactions (Xie et al., 2025) may function as transcriptional regulators of human brain development and functions in defined brain regions, thus recapitulating one of the main biological features of the TE-bound 716 initiator TFs (Glinsky, 2026). One of the central elements of the HAP concept is the involvement of pioneer transcription factors (TFs), especially the Yamanaka factors, in the development and function of the human brain. Remarkably, cognitive rejuvenation has been observed through targeted partial reprogramming of engram neurons by three Yamanaka factors (OSK): POU5F1 (OCT4), SOX2, and KLF4 (Berdugo-Vega et al., 2026). Reportedly, OSK selectively delivered to engram neurons reversed the expression of molecular features considered hallmarks of aging and Alzheimer’s disease (AD). Furthermore, gene therapy mediated by targeted delivery of OSK appears to normalize synaptic plasticity-associated epigenetic and transcriptional profiles and improve neuronal hyperexcitability, which is typically associated with AD. Direct evidence of cognitive rejuvenation was demonstrated by observations that OSK-mediated reprogramming of engram neurons seems to recover learning and memory capacities to levels observed in healthy young animals (Berdugo-Vega et al., 2026).

### Retrotransposon-derived DNA provides PPI seed templates for protein dimerization, facilitating the HAP of endogenous multiprotein complexes

One of the principal findings reported in this contribution is that DNA sequences derived from distinct families of TEs seed millions of TFBS for a consensus set of ~700 TFs that may function as initiators of protein hetero- and homo-dimerization. Thus, PPI chain reactions originating at these initiators may facilitate the creation of intracellular pools of protein dimers, serving as constituents of the HAP of multiprotein complexes. In support of this model, notable features of the protein constituents of HAPs identified herein are documented by significant enrichments (adjusted p-value < 0.05) in thousands of endogenous macromolecular complexes (EMCs) reported in numerous databases, namely, 1,792 records from the NURSA Protein Complexes database; 383 records from the CORUM database; 6,607 records from the virus–host PPI P-HIPSTer 2020 database; and 477 records from the Jensen Compartments database. The efficiency of multiprotein complex assembly significantly depends on whether the process initiates from monomer components or pre-formed dimers (Supplementary Text “Comparative analysis of the relative efficiency of the multiprotein complexes assembly pathways distinguished by the initiation from the monomers versus dimers”). Detailed examination of the importance of assembly order and the role of pre-formed sub-complexes has demonstrated that assembly pathways are not always linear, and certain readily available sub-complexes can greatly increase the speed and accuracy of final complex formation (Marsh and Teichmann, 2011; 2015). Analysis of the complexity of protein interactions and complex assembly supports the concept that the number of assembly steps, which is highly dependent on whether complex assembly starts with monomers or dimers, greatly impacts the probability of successful complex formation (Levy et al., 2006). However, the ultimate outcome of the assembly process varies depending on the properties of the specific interacting proteins, as well as the conditions and nature of their interactions. The observations reported herein add credibility to arguments supporting the feasibility of the HAP concept by demonstrating how TE-derived DNA sequences encoding TFBS could function as initiators of protein dimerization reactions, thereby facilitating the formation of an intracellular pool of protein dimers serving as HAP facilitators. Conceptually, a hybrid pathway of multiprotein complex assembly, where pre-formed dimers initiate the process, followed by fine-tuning with monomer additions at subsequent steps, could indeed be a highly efficient strategy. Arguably, this hybrid pathway combines the strengths of both monomer and dimer complex assembly pathways, minimizing their respective weaknesses. Advantages of a hybrid pathway of multiprotein complex assembly include (i) Increased speed, reliability, and stability: Forming dimers as a first step provides a rapid, reliable, and relatively stable foundation for complex assembly. This bypasses the potentially slower, less reliable, and less stable initial interactions of individual monomers. (ii) Enhanced regulatory control: While dimer formation provides a base structure, the addition of individual monomers allows for fine-tuned regulation of the complex’s final composition and activity. This enables responses to subtle changes in cellular conditions, as individual monomer concentrations can be rapidly adjusted. (iii) Reduced likelihood of misassembly: By establishing a dimer core, the number of possible incorrect interactions in subsequent steps is reduced. The dimer acts as a sort of “scaffold,” guiding the correct assembly pathway. (iv) Adaptability through diversification: This pathway allows for complexes that have a core set of proteins (the dimers) and then incorporate variable proteins (the monomers) as needed. This enables great adaptability to differing cellular requirements. According to one plausible scenario for the HAP of multiprotein complexes, the cell could maintain a pool of pre-formed dimers, ready to initiate complex assembly when needed. Defined regulatory signals could then control the addition of specific monomers, allowing for precise control over the complex’s final structure and function. This strategy could be particularly advantageous for large, intricate multiprotein complexes where both assembly speed and accuracy are essential. One determinant favoring a hybrid pathway is the increased speed and stability of multiprotein complex assembly when pre-formed dimers serve as a rapid foundation. Pre-formed complex assembly intermediates, such as dimers, can significantly accelerate assembly by reducing the search space for interacting partners and providing the kinetic advantages of pre-nucleation (Knowles et al., 2009). These pre-assembled intermediates facilitate efficient complex formation by increasing stability and reducing the entropic cost of assembly (Marsh and Teichmann, 2011; 2015). Dimer formation often involves extensive hydrophobic interactions, which enhance the stability of dimer cores and contribute to the increased stability of the resulting multiprotein complexes (Nooren and Thornton, 2003). Fine-tuning, through the addition or removal of monomer subunits to pre-formed cores, facilitates dynamic control over the composition and activity of multiprotein complexes (Pawson and Nash, 2003). The concept of fine-tuned regulation of macromolecular complexes through dynamic monomer additions is supported by evidence that post-translational modifications, such as phosphorylation, can regulate multiprotein complex assembly by affecting interactions between individual subunits (Cohen, 2000). Monomer additions can also cause allosteric shifts within the multiprotein complex, modifying its activity (Ollivier et al., 2010). Another major advantage of a hybrid pathway for multiprotein complex assembly is the apparent reduced likelihood of misassembly, in part, because pre-formed dimers could function as complex-specific scaffolds or self-chaperones. The importance of chaperone proteins and other quality control mechanisms in preventing misassembly has been comprehensively characterized (Frydman, 2001). Importantly, analysis of the checks and balances cells employ to ensure proper assembly of multiprotein complexes suggests that a pre-formed dimer core would serve as an important early checkpoint, reducing the likelihood of misassembly (Ferrell, 2002). The assembly of large, intricate complexes like the ribosome or the spliceosome provides examples of how cells use hierarchical assembly pathways to ensure accuracy and efficiency. A classic example is the ribosome assembly pathway, which involves the ordered addition of ribosomal proteins and RNA molecules, highlighting the importance of hierarchical assembly for large complexes (Nomura, 1973). The spliceosome assembly pathway is another example of a complex process that relies on ordered assembly steps to ensure accuracy (Wahl et al., 2009).Regulatory signals that control monomer additions during the assembly of multiprotein complexes are crucial for intricate regulation and adaptability. The modular nature of protein evolution allows for the addition or removal of individual protein domains or subunits, leading to complexes with diverse functions (Marsh and Teichmann, 2011; 2015). Cells frequently employ combinatorial control to generate a wide range of signaling outputs from a limited number of components (Alon, 2007). This supports the concept of using a core dimer structure with variable monomer additions to create diverse complexes. Studies on signaling pathways and gene regulation offer numerous examples of how various regulatory signals, such as site-specific protein phosphorylation or ligand binding, can control the addition of specific monomers to protein complexes. Classic examples demonstrate how phosphorylation can regulate protein-protein interactions and complex assembly (Hunter, 1995). Membrane-associated signaling complexes often involve pre-formed dimers or oligomers (Cock et al., 2002). The pre-assembly of core complexes, including dimers, is frequently engaged during responses to cellular stimuli. For instance, studies on receptor tyrosine kinase signaling have firmly established that receptor dimerization is an obligatory step in activating signaling pathways.

The availability of genome-wide, chromosome-naive networks of TE-seeded TFBS, which serve as initiators of protein dimerization reactions to create intracellular pools of dimeric protein constituents of various EMCs, should facilitate the operation of HAPs in human cells. The compositions of pre-assembled protein dimers on DNA templates and the availability of the ensemble of monomeric protein constituents of EMCs would then determine both the housekeeping constitution and cell type-specific configurations of EMCs’ architectural designs and their functional frameworks in a given cell type.

### Integrated view of the biological significance of hybrid assembly pathways of EMCs

A defining feature of the proposed hybrid assembly pathways is that their initiation is not constrained by the availability of genomic template sites. Approximately 4.5 million TE-derived loci are present in the human genome of every cell, providing an effectively non-limiting reservoir of DNA templates. Under these conditions, the initiation and propagation of PPI cascades are expected to be governed primarily by the availability and intracellular dynamics of protein constituents rather than by DNA template scarcity. This configuration supports the continuous, real-time operation of hybrid assembly pathways, with initiation events distributed across vast numbers of TE-encoded loci, many of which reside within heterochromatic genomic contexts. Importantly, the DNA-templated initiation of these pathways bifurcates into two biologically congruent modes of PPI cascade propagation. TF–TF cascades are enriched among genes highly expressed across hundreds of neuroanatomically defined brain regions, consistent with a role in establishing and maintaining region-specific transcriptional identity. In parallel, TF-HUB cascades are enriched for protein constituents of synaptogenesis and mature synaptic structures, implicating this branch in the assembly and maintenance of synaptic connectivity. Together, these bifurcated cascades provide a unified genomic framework linking region-level identity and synaptic architecture, enabling coordinated development and maintenance of brain regions and their connectivity. Notably, subsets of TFs that initiate PPIs are significantly enriched during brain development and within the transcriptional signatures of mature neuroanatomical regions, suggesting their role in maintaining regional integrity. The availability of high-resolution gene expression maps across thousands of brain regions allows this framework to generate experimentally testable predictions. These include identifying small TF subsets responsible for specifying and maintaining distinct brain regions and their associated synaptic programs. During primate evolution, emerging human-specific GREs, namely, HAQREs and HARs, appear to acquire regulatory control over the development and function of brain regions. Some of these regions were previously considered chimpanzee-specific, based on genomic analyses of developmental trajectories revealed by species-specific brain organoid experiments. These potential regulatory effects of human-specific GREs are particularly intriguing in multiple cerebellar regions, including Crus 1, Crus 2, the cerebellar hemisphere, Crus 1 granular layer, Crus 1 molecular layer, Crus 2 granular layer, and Crus 2 molecular layer. This notion is underscored by recent discoveries of the important functional roles of cerebellar components in human language networks (Casto et al., 2026). One cerebellar region, spanning Crus ½/VIIb, seems especially important due to its high selectivity for language over non-linguistic inputs and its mirroring of the neocortical language network (Casto et al., 2026).

### General significance, limitations, and future directions

This contribution introduces the novel molecular biology concept of Hybrid Assembly Pathways (HAPs) of endogenous multiprotein complexes (EMCs) and highlights previously unexplored fundamental and translational areas of scientific inquiry. For example, to date, only a small fraction of initiator TFs (~14%) has been experimentally defined as constituents of DNA-bound TF–TF complexes. Studies on the precise molecular tracing of protein dimer formation on DNA templates, the release of protein dimers from DNA scaffolds, and their delivery to EMC assembly sites are lacking. The molecular principles governing the selection of protein constituents for dimer formation on DNA templates and the postulated functions of protein dimers as powerful attractors for monomers during EMC assembly catalysis remain unknown. Single-molecule level structure-function analyses of kinetics, affinity constants, and 3D-conformation dynamics of protein dimers in native chromatin contexts are highly desirable. How these processes are governed and executed within cell type-specific differentiation programs also remains unknown. Since protein dimer formation is considered an obligate, perhaps essential, molecular event during EMC assembly, it is possible that protein dimers may catalyze the formation of liquid-liquid phase-separated condensates at intermediate stages of EMC assembly maturation. Investigation of all these exciting opportunities is now feasible and could be readily explored to dissect the molecular anatomy of specific EMC assembly, with selections tailored to tissue- and organ-specific features of cellular differentiation programs across the human body. One of the key concepts introduced in this study is the recognition of the fundamental contribution of endogenous retroviruses and other families of Tes to the evolution of regulatory DNA sequences in the genomes of modern humans and non-human primates. This occurs by providing millions of binding sites for TFs, which serve as initiators of HAPs of thousands of EMCs. However, observations reported in this contribution highlight additional novel aspects of potential fundamental and translational relevance to our understanding of infection biology and virus–host interactions. Among the notable observations documented is the apparent engagement of HAP protein constituents in PPIs with a broad spectrum of viral proteins recorded in the VirusMINT database. These include viral proteins of Ebola virus (Mayinga variant), Epstein–Barr virus, hepatitis C virus, human cytomegalovirus, human adenoviruses, human herpes viruses, human papillomaviruses, and human immunodeficiency virus type 1. These findings suggest that molecular interference with HAP functions in host cells may constitute one of the common mechanisms of pathogenesis of viral infections. GSEA of HAP protein constituents revealed that PPI enrichment patterns appear to reach a plateau at PPI levels 2–3, as illustrated by the consistently maintained high level of significant enrichment of TF-HUB constituents implicated in 6,604 instances of virus–host PPIs recorded in the virus–host PPI P-HIPSTer 2020 database (**Figure 1**). This magnitude of potential engagements of HAP protein constituents in virus–host PPIs suggests that viruses may have significant and multifaceted impacts on the evolution of PPI networks in human cells.

## Conclusion

TE-derived DNA sequences occupy approximately 50% of the human genome, collectively seeding more than 4.5 million confidently mapped TE sequences (Hoyt et al., 2022). The structural–functional features common to regulatory DNA sequences seeded by vastly diverse TE families, identified and characterized herein, may reflect the existence of previously underappreciated global genomic regulatory networks (GRNs) operating within chromosome-naive global genomic contexts. One of the principal findings reported in this contribution is that DNA sequences derived from distinct families of Tes seed millions of TFBS for a consensus set of approximately 700 TFs. These TFs may function, upon binding to DNA, as initiators of protein hetero- and homo-dimerization. It is postulated that cascades of PPI chain reactions, originating at the initiator TFs, may facilitate the creation of cell-type-specific intracellular pools of protein dimers. These dimers would serve as constituents of hybrid assembly pathways (HAP) for endogenous multiprotein complexes. In support of this model, this report documents notable biologically significant structural–functional features and mechanisms of action of the identified protein constituents of HAPs, including the crucial role of pioneer TFs such as the Yamanaka factors. An interesting observation, reflecting on HAP-facilitated mechanisms of human brain development and function, is the apparently congruent regulation of developmental patterns of hundreds of neuroanatomical structures and the creation of functional synapses. This is afforded by coordinated activities of PPI cascades along two concurrent modes of operation: TF–TF PPI chain reactions enabling neurogenesis and HUB-PPI chain reactions facilitating synaptogenesis. Under this regime, the bifurcation of DNA-initiated PPI cascades into TF–TF and TF-HUB branches is not a sequential developmental choice but a parallel, congruent process, coordinating regional transcriptional identity and synaptic structural assembly within the same cellular and neuroanatomical contexts. Accordingly, cell-type specificity, regional identity, and synaptic architecture are predicted to emerge from differential protein expression and their interaction propensities, rather than from selective activation of a limited number of genomic loci. It should be emphasized that there are major distinctions between the HAP concept and classical DNA-templated assemblies, exemplified by transcription regulatory complexes or DNA repair complexes. Classical DNA-templated assemblies focus on multiprotein complexes that are assembled and function on the DNA template. The HAP of EMCs; however, identifies DNA as a template for protein dimers that operate upon release from the DNA template to facilitate the accelerated assemblies of EMCs. This contribution reports multiple independent lines of evidence supporting the concept that brain development regulatory TFs may play a significant role in executing transcriptional programs responsible for maintaining the structural and functional integrity of mature neuroanatomical regions (Glinsky, 2026). The first lines of evidence are based on follow-up GSEA of brain development regulatory TFs, demonstrating that subsets of the AI assistant-discovered panel of 25 master brain regulatory TFs are significantly enriched among genes upregulated in 215 human brain regions. Simultaneously, subsets of the top 25 TFs, identified by UniBind differential-enrichment analyses of TFs bound to human-specific versus chimpanzee-specific BDRRs, are significantly enriched among genes upregulated in 202 human brain regions (Glinsky, 2026). Subsequent evidence supporting this concept was reported based on analyses of numerous independently identified panels of candidate brain development regulatory TFs bound to multiple families of GREs of distinct evolutionary origins. Third, evidence was documented based on analyses of all TFs manifesting significantly distinct enrichment profiles in binary comparisons of TFs bound to human-specific BDRRs versus chimpanzee-specific BDRRs, further supporting the validity of this concept. Since differentially expressed sets of genes, whose expression is significantly upregulated in many thousands of precisely neuroanatomically defined brain structures, are well documented, these observations offer simple analytical and experimental strategies for identifying specific sets of TFs likely responsible for the precise regulation of defined brain regions’ development, as well as the transcriptional control of their structural and functional integrity maintenance in the adult brain. Examples of practical implementations of these concepts are reported in a series of analytical experiments aimed at identifying and characterizing the molecular features of the human claustrum’s transcriptional connectome. The results of these analyses provided a foundation for a homeostatic model of transcriptional identity maintenance and survival of claustrum projection neurons, highlighting the principal regulatory role of the top-scoring initiator transcription factor NR4A2 (NURR1).The proposed DNA-templated initiation of hybrid assembly pathways is not constrained by the availability of genomic template sites. Approximately 4.5 million TE-derived loci are present in the human genome of every cell, providing an effectively non-limiting reservoir of initiation templates. Under these conditions, the rate and extent of PPI cascade initiation are expected to be governed primarily by the availability and intracellular dynamics of protein constituents rather than by DNA template scarcity. This configuration supports the notion that hybrid assembly pathways may operate continuously and in real time, with initiation events distributed across vast numbers of TE-encoded loci. The enrichment of TE-derived initiation loci within heterochromatic genomic contexts may be functionally advantageous rather than inhibitory. Heterochromatin-associated TE loci frequently harbor nucleosome-depleted islands capable of supporting transient protein binding while remaining insulated from transcriptional interference. Such environments are well suited for repeated cycles of protein dimer nucleation and release, enabling chromosome-naive, location-independent initiation of PPI cascades. Within this genomic framework, the bifurcation of DNA-templated PPI cascades into TF–TF and TF-HUB protein branches likely occurs concurrently and continuously. The TF–TF branch preferentially reinforces transcriptional programs that define neuroanatomical region identity, partly by encoding sets of initiator TFs. In contrast, the TF-HUB branch enriches protein constituents essential for synaptogenesis and the structural–functional maintenance of synapses. Together, these congruent cascades provide a unified mechanism for the coordinated maintenance of regional identity and synaptic architecture in the mature brain. Therefore, TE-encoded DNA templates offer a massively parallel, protein-limited initiation layer that continuously bifurcates PPI cascades into TF–TF and TF-HUB branches, enabling the coordinated development and maintenance of neuroanatomical brain regions and synaptic connectivity.

## Methods

### Data source and analytical protocols

Publicly available datasets and web-based resources were utilized in this study. Initial analyses of PPI networks focused on human embryo regulatory LTR loci (see the Introduction) identified as highly conserved pan-primate regulatory sequences, having been present in primate genomes for at least ~15 MYA (Glinsky, 2022; 2025; 2026). Specifically, four distinct LTR families meeting these criteria, namely, MLT2A1 (2,416 loci), MLT2A2 (3,069 loci), LTR7 (3,354 loci), and LTR5_Hs (606 loci) were analyzed (Glinsky, 2022; 2025; 2026). Thus, a total of 9,445 fixed, non-polymorphic sequences of human embryo regulatory LTR elements residing in the genomes of all modern humans (hg38 human reference genome database) were retrieved and analyzed as described in recent studies (Hashimoto et al., 2021; Carter et al., 2022; Glinsky, 2022; 2025; 2026). The number of highly conserved orthologous loci in the genomes of sixteen non-human primates (NHP) was determined precisely as previously reported (Glinsky, 2022; 2025; 2026). Fixed, non-polymorphic regulatory LTR loci and other families of genomic regulatory elements (GRE) in the human genome (hg38 human reference genome database) were considered highly conserved in the NHP genome only if the following two requirements were met (Glinsky, 2025; 2026):During the direct LiftOver test (https://genome.ucsc.edu/cgi-bin/hgLiftOver), the human LTR sequence mapped to a single orthologous locus in the NHP genome with a threshold of at least 95% sequence identity. During the reciprocal LiftOver test, the NHP sequence identified in the direct LiftOver test remapped with at least 95% sequence identity threshold to the exact same human orthologous sequence queried during the direct LiftOver test. To investigate the dynamics and qualitative and quantitative characteristics of TFs binding to regulatory loci, as well as TFBS density changes within defined sets of genomic regulatory elements, a total of 200,393 distinct genomic sequences were interrogated. This included 49,667 sequences representing control sets of genomic loci and 150,726 genomic regulatory elements (GREs) (**Supplementary Data Sets S1**, **S2**; Data Sets Summary sheet). For assessments of TF binding and TFBS density changes within brain development regulatory regions (BDRRs) during primate evolution, we used a set of 37,205 Rhesus Macaque BDRRs as a reference. These BDRRs were identified using ATAC-seq of brain organoids and re-mapped to the hg19 human reference genome database (Kanton et al., 2019; Glinsky, 2025; 2026). To identify TFBS within genomic regulatory loci known to contribute to human and chimpanzee brain development, we analyzed 17,935 genomic regulatory regions reported in the organoid single-cell genomic atlas of human and chimpanzee brain development (Kanton et al., 2019). For interspecies comparative analyses, Kanton et al. (2019) mapped 8099 human-biased and 9836 chimpanzee-biased ATAC-seq defined differentially accessible DNA sequences to the hg19 human reference genome database. The 39,994 Rhesus Macaque BDRRs, identified using ATAC-seq of brain organoids (accessible peaks in macaque cerebral organoids mapped to rheMac8), were converted to hg19 coordinates using the LiftOver algorithm at 95% sequence identity thresholds, resulting in 37,205 Rhesus Macaque BDRRs mapped to hg19. A catalog of TFs with putative TFBS within 8099 human-specific and 9836 chimpanzee-specific BDRRs was compiled (Glinsky, 2026). Kanton et al. (2019) identified BDRRs using the Assay for Transposase-Accessible Chromatin with high-throughput sequencing (ATAC-seq), a DNA sequence unbiased open chromatin accessibility screening method (Buenrostro et al., 2013; 2015).We analyzed a set of 751 SVA family TE loci co-opted as functional cis-regulatory elements in human induced pluripotent stem cells (Barnada et al., 2022) and a set of 3,265 TE loci involved in TE-governed hippocampal neurogenesis regulatory pathways in humans and chimpanzees (Patoori et al., 2022). Patoori et al. (2022) reported these TE-governed hippocampal neurogenesis regulatory pathways using a model of human and chimpanzee induced pluripotent stem cell differentiation into TBR2 (or EOMES)-positive hippocampal intermediate progenitor cells. Analyses of TFBS in human and non-human primate BDRRs were performed using DA-ATAC sequence alignments in the hg19 reference genome database. Sequence coordinates of Modern Human and Chimpanzee DA-ATAC-seq-defined BDRRs were reported in the hg19 human reference genome database in the original publication (Kanton et al., 2019). The 37,205 Rhesus Macaque BDRRs (hg19 coordinates) identified using ATAC-seq of brain organoids were derived by converting 39,994 sequences reported in rheMac8 coordinates (Kanton et al., 2019) to hg19 coordinates using the LiftOver algorithm at 95% sequence identity thresholds. Proteins comprising structural components of the post-synaptic densities (PSD) of excitatory and inhibitory synapses were identified by analyzing reported sets of proteins that constitute multiprotein complexes of excitatory and inhibitory synapses (Miski et al., 2022; and references therein).Transcription factor binding sites (TFBS) within candidate genomic regulatory loci were identified using the Jaspar algorithm (Jaspar Transcription Factors), accessible via the UCSC Genome Browser Table Browser functions (https://genome.ucsc.edu/cgi-bin/hgTables). This tool facilitates data downloading, filtering, analysis, and retrieval from the Genome Browser (Castro-Mondragon et al., 2022; Rauluseviciute et al., 2024). TFBS identification and data retrieval employed default threshold settings, imputing up to 1,000 loci per screen to ensure full coverage of specified genomic regions of interest based on hg38 and hg19 human reference genome coordinates. All identified TFBS were retrieved, and individual transcription factors (TFs) associated with these sites were cataloged. For each set of TFBS, quantitative features were documented by calculating the number of events for each distinct TFBS. These values were reported as TFBS frequency (number of TFBS per regulatory locus) and TFBS density (TFBS frequency normalized to 1 Kb of locus length). TFs bound to defined sets of genomic regulatory elements (GREs) were identified using the UniBind algorithm (https://unibind.uio.no/) in the “robust” mode of the differential enrichment setting, following the development and implementation requirements reported by Puig et al. (2021) and specified on the UniBind website. Identities of TFs bound to specific GREs were defined at several statistical thresholds, including a nominal p-value less than 0.05, after correction for multiple hypothesis testing using Benjamini–Hochberg and Bonferroni protocols at a false discovery rate (FDR) of 5%. Gene interactions were analyzed using publicly available web-based resources for gene interactions and pathways from curated databases and text mining, focusing only on interactions with database support (UCSC Genome Browser Gene Interaction Graph), following the recommendations of the user’s guide (Gene and Pathways Interaction Graph User’s Guide).To define the transcriptional connectome of the human claustrum, the top 2,000 transcripts with significantly increased expression levels in the human claustrum, compared to other human brain regions, were retrieved from the web-accessible portal of the Allen Brain Atlas database (**Supplementary Data Set S6**; Microarray Data:: Allen Brain Atlas: Human Brain). The dataset was processed to remove redundant records and non-RefSeq entries, resulting in a panel of 1106 genes defined herein as genetic markers of the human claustrum (**Figure 12**; **Supplementary Data Set S6**). Detailed descriptions of the analytical steps for defining the molecular features of the mouse claustrum transcriptome are reported in **Supplementary Data Set 7**. Results of experiments on the development and characterization of the mouse model of *Nr4a2* haploinsufficiency were reported elsewhere (Fodoulian et al., 2025). Additional details of these experimental pipelines are reported in **Supplementary Data Sets S6** and **S7**.The significance of differences between expected and observed event numbers was calculated using a two-tailed Fisher’s exact test (https://www.langsrud.com/fisher.htm; Agresti, 1992). Multiple proximity placement enrichment tests were conducted for individual families and subsets of LTRs, BDRRs, and human-specific regulatory regions (HSRS). These tests accounted for the base pair size of corresponding genomic regions, size distributions of topologically associating domains in human cells, distances to putative regulatory targets, and bona fide regulatory targets identified in targeted genetic interference and/or epigenetic silencing experiments. Additional methodological and analytical details are provided in the text, **Supplementary Materials**, and previously reported contributions (Barakat et al., 2018; Fuentes et al., 2018; Glinsky, 2015; 2016a; b; c; 2018; 2020a; b; c; 2021; 2022; Guffanti et al., 2018; Glinsky and Barakat, 2019; McLean et al., 2010; 2011; Pontis et al., 2019; Wang et al., 2014).

### Gene set enrichment and genome-wide proximity placement analyses

Gene set enrichment analyses were performed using the Enrichr bioinformatics platform, which provides access to nearly 200,000 gene sets from over 100 gene set libraries. The Enrichr API (January 2018 through January 2023 releases) (Chen et al., 2013; Kuleshov et al., 2016; Xie et al., 2021) was utilized to test genes linked to regulatory LTR elements, HSRS, or other regulatory loci of interest for significant enrichment in numerous functional categories. Patterns of TF–TF PPI cascades and HUB protein PPI cascades were analyzed via web-based links to corresponding databases on the Enrichr website. When technically feasible, larger gene sets comprising several thousand entries were analyzed. Regulatory connectivity maps between HSRS, regulatory LTRs, and coding genes, along with additional functional enrichment analyses, were performed using the Genomic Regions Enrichment of Annotations Tool (GREAT) algorithm (McLean et al., 2010; 2011) at default settings. The reproducibility of the results was validated by implementing two releases of the GREAT algorithm: GREAT version 3.0.0 (02/15/2015 to 08/18/2019) and GREAT version 4.0.4 (08/19/2019), applying default settings at differing maximum extension thresholds as previously reported (Glinsky, 2020a, b, c; 2021; 2022; 2025; 2026). The GREAT algorithm enables investigators to identify and annotate genome-wide connectivity networks of user-defined distal regulatory loci and their putative target genes. Concurrently, it performs functional Gene Ontology (GO) annotations and statistical enrichment analyses of GO annotations for identified genomic regulatory elements (GREs) and target genes, thereby allowing the inference of potential biological impacts of interrogated genomic regulatory networks. The GREAT algorithm was employed to identify putative downstream target genes of human embryo regulatory LTRs. Simultaneously with the identification of putative regulatory target genes of GREs, the GREAT algorithm conducts stringent statistical enrichment analyses of functional annotations for identified downstream target genes, enabling the inference of potential significance of phenotypic impacts of interrogated GRNs. Importantly, assigning phenotypic traits as putative statistically valid components of GRN actions requires assessing the statistical significance of enrichment for both GREs and downstream target genes using independent statistical tests.

### Genome-wide proximity placement analysis

GPPA was performed on downstream target genes and distinct genomic features that co-localize with regulatory LTRs, HSRS, BDRRs, and other regulatory loci. This analysis was conducted as previously described and originally implemented for human-specific transcription factor binding sites (Glinsky et al., 2018; Glinsky, 2015; 2016a; b; c; 2017; 2018; 2020a; 2020b; 2020c; 2021; 2022; 2025, 2026; Guffanti et al., 2018).The validity of statistical definitions for genomic regulatory networks (GRNs) and genomic regulatory modules (GRMs), based on binomial (regulatory elements) and hypergeometric (target genes) FDR Q values, was assessed using a directed acyclic graph (DAG) test. This test is based on enriched terms from a single ontology-specific table generated by the GREAT algorithm (Glinsky, 2025). The DAG test identifies patterns and directions of connections between significantly enriched GO modules, drawing upon the experimentally documented temporal logic of developmental processes and structural/functional relationships between statistically significant terms defined by gene ontology enrichment analysis. A specific DAG test utilizes a subset of statistically significant GRMs from a single gene ontology-specific table generated by the GREAT algorithm. It achieves this by extracting GRMs that exhibit connectivity patterns defined by experimentally documented developmental and/or structure/function/activity relationships. These GRMs are considered valid observations and are visualized as a consensus hierarchy network of the ontology-specific DAGs (Glinsky, 2025). Based on these considerations, the DAG algorithm constructs a hierarchy of connectivity between statistically significant gene ontology-enriched GRMs, guided by developmental and structure/function/activity relationships.

### Differential GSEA to infer the relative contributions of distinct subsets of GREs and downstream target genes on phenotypes of interest

When technically and analytically feasible, different sets of regulatory LTRs and other families of GREs (**Supplementary Data Sets S1**, **S2**), along with candidate downstream target genes defined at several significance levels of statistical metrics (comprising dozens to several thousand individual genetic loci), were analyzed using differential GSEA. This approach was employed to gain insights into the biological effects of regulatory LTRs and downstream target genes, and to infer potential mechanisms of phenotype-affecting activities. Previously, this approach was successfully implemented for the identification and characterization of human-specific regulatory networks governed by human-specific transcription factor-binding sites (Glinsky et al., 2018; Glinsky, 2015, 2016a, 2016b, 2016c, 2017, 2018, 2020a, 2020b, 2020c, 2021; 2022; 2025, 2026; Guffanti et al., 2018; Glinsky and Barakat, 2019) and functional enhancer elements (Barakat et al., 2018; Glinsky et al., 2018; Glinsky and Barakat 2019; Glinsky, 2015, 2016a, 2016b, 2016c, 2017, 2018, 2020a, 2020b, 2020c, 2021; 2022; 2025; 2026). The differential GSEA approach has been utilized for the characterization of phenotypic impacts of 13,824 genes associated with 59,732 human-specific regulatory sequences (Glinsky, 2020a), 8,405 genes associated with 35,074 human-specific neuroregulatory single-nucleotide changes (Glinsky, 2020b), 8,384 genes regulated by stem cell-associated retroviral sequences (SCARS) (Glinsky, 2021), as well as human genes and medicinal molecules affecting susceptibility to SARS-CoV-2 coronavirus (Glinsky, 2020c). Initial GSEA entails interrogating each specific set of candidate downstream target genes using approximately 70 distinct genomic databases, including comprehensive pathway enrichment gene ontology (GO) analyses. These analyses were followed by in-depth interrogations of the identified significantly enriched gene sets, employing selected genomic databases deemed most statistically informative from the initial GSEA. In all reported tables and plots (unless stated otherwise), in addition to the nominal p-values and adjusted p-values, the Enrichr software calculates the “combined score,” which is a product of the significance estimate and the magnitude of enrichment (combined score c = log(p) * z, where p is the Fisher’s exact test p-value and z is the z-score deviation from the expected rank).

### Statistical analyses of datasets

The significance of differences in the number of events between groups was calculated using two-sided Fisher’s exact and Chi-square tests. The significance of the overlap between events was determined using the hypergeometric distribution test (Tavazoie et al., 1999).All statistical analyses of publicly available genomic datasets, including error rate estimates, background and technical noise measurements and filtering, feature peak calling, feature selection, assignments of genomic coordinates to the corresponding builds of the reference human genome, and data visualization, were performed exactly as reported in the original publications and associated references linked to the corresponding data visualization tracks (http://genome.ucsc.edu/). Additional elements or modifications of statistical analyses are described in the corresponding sections of the Results. Statistical significance of the correlation coefficients was determined using GraphPad Prism version 6.00 software. Nominal p-values, along with Benjamini-Hochberg and Bonferroni adjusted statistical threshold values (at FDR 5%), were estimated and considered as reported in corresponding sections of the Results. A nominal p-value of less than 0.05, as well as Q-values after correction for multiple hypothesis testing employing the Benjamini-Hochberg and Bonferroni protocols at a false discovery rate of 5%, were computed and considered to draw inferences of potential biological significance from reported observations.

## Data Availability

The original contributions presented in the study are included in the article/**Supplementary Material**. Further inquiries can be directed to the corresponding author.
