# Supplementary material for: Cooperation of transposable elements to endow global networks of initiators of hybrid assembly pathways of endogenous multiprotein complexes
Source: Front Cell Infect Microbiol. 2026 Jul 30;16:1793868. doi: 10.3389/fcimb.2026.1793868 (PMC13468005; doi:10.3389/fcimb.2026.1793868)
Supplement: Supplementary file 1 [file DataSheet1.zip › Supplementary Table S2. LTR5_Hs and LTR7 12 brain regions.docx]

**Table 3.** Transcription factors (TFs) engaged in protein-protein interactions (PPIs) with 35 TFs bound to both LTR5_Hs and LTR7 loci are enriched in 12 human brain regions defined by the Allan Brain Atlas database of up-regulated genes.

| Brain regions | Number of genes | P-value | Adjusted P-value | Odds Ratio | Combined Score | Genes |
| --- | --- | --- | --- | --- | --- | --- |
| Postsubiculum, layer 3 | 11 | 7.90E-06 | 0.016 | 5.8 | 68.7 | NR4A2;NR4A1;BCL11A;E2F1;TCF4;ZBTB33;GATA3;FOS;NR3C1; KLF4;ETS2 |
| Posterolateral visual area, layer 6a | 10 | 4.65E-05 | 0.019 | 5.3 | 52.4 | NR4A1;PHC1;KLF5;CTNNB1;ZBTB33;GATA3;FOS;NR3C1;JUNB; POU5F1 |
| Posterolateral visual area, layer 6b | 10 | 4.65E-05 | 0.019 | 5.3 | 52.4 | NR4A1;PHC1;KLF5;NFYA;E2F1;EP300;CTNNB1;GATA3;NR3C1; POU2F2 |
| Presubiculum, layer 3 | 10 | 4.65E-05 | 0.019 | 5.3 | 52.4 | NR4A2;NR4A1;SALL1;KLF5;BCL11A;TCF4;ZBTB33;GATA3;NR3C1;ETS2 |
| layer 6a of PaS | 10 | 4.65E-05 | 0.019 | 5.3 | 52.4 | NR4A2;NR4A1;BCL11A;E2F1;TCF4;ZBTB33;FOS;NR3C1;KLF4; ETS2 |
| Anteroventral periventricular nucleus | 9 | 2.47E-04 | 0.041 | 4.7 | 38.8 | AR;HNF4A;BCL3;E2F1;NR2F2;POU2F2;KLF4;CLOCK;ESR1 |
| Crus 1, molecular layer | 9 | 2.47E-04 | 0.041 | 4.7 | 38.8 | TCOF1;CHD7;AHR;ETS1;FOXP3;RCOR2;POU5F1;PHF8;FOXA2 |
| Dentate gyrus, granule cell layer | 9 | 2.47E-04 | 0.041 | 4.7 | 38.8 | NCOA1;SUZ12;UPF1;NCOR1;CBFB;MAZ;ZFPM2;CLOCK;HCFC1 |
| Medial amygdalar nucleus, posterodorsal part, sublayer a | 9 | 2.47E-04 | 0.041 | 4.7 | 38.8 | AR;CBX3;HNF1B;GATA3;GATA2;RUNX3;ILF2;ESR1;FLI1 |
| Primary visual area, layer 6a | 9 | 2.47E-04 | 0.041 | 4.7 | 38.8 | NR4A1;PHC1;E2F1;ERG;GATA3;FOS;NR3C1;KLF4;KLF2 |
| layer 6b of RSC | 9 | 2.47E-04 | 0.041 | 4.7 | 38.8 | NR4A1;PHC1;NFYA;BCL11A;E2F1;CTNNB1;FOS;NR3C1;KLF4 |
| sublayer 6a of OCx | 9 | 2.47E-04 | 0.041 | 4.7 | 38.8 | STAT5A;NR4A1;E2F1;NR0B1;SIRT6;ERG;FOS;NR3C1;KLF2 |
| Crus 1 | 8 | 0.001175 | 0.068 | 4.1 | 27.7 | TCOF1;CHD7;AHR;FOXP3;RCOR2;POU5F1;PHF8;FOXA2 |
