# Supplementary material for: Cooperation of transposable elements to endow global networks of initiators of hybrid assembly pathways of endogenous multiprotein complexes
Source: Front Cell Infect Microbiol. 2026 Jul 30;16:1793868. doi: 10.3389/fcimb.2026.1793868 (PMC13468005; doi:10.3389/fcimb.2026.1793868)
Supplement: Supplementary file 1 [file DataSheet1.zip › Supplementary Table S1. LTR7 13 brain regions.docx]

**Table 2.** Transcription factors (TFs) engaged in protein-protein interactions (PPIs) with LTR7-bounf TFs are enriched in 13 human brain regions defined by the Allan Brain Atlas database of up-regulated genes.

| Brain regions | Number of genes | P-value | Adjusted P-value | Odds Ratio | Combined Score | Genes |
| --- | --- | --- | --- | --- | --- | --- |
| Anteroventral periventricular nucleus | 10 | 4.95E-05 | 0.033 | 5.2 | 51.7 | AR;HNF4A;BCL3;E2F1;E2F2;NR2F2;POU2F2;KLF4;CLOCK;ESR1 |
| Posterolateral visual area, layer 6b | 10 | 4.95E-05 | 0.033 | 5.2 | 51.7 | NR4A1;PHC1;KLF5;NFYA;E2F1;EP300;CTNNB1;GATA3;NR3C1; POU2F2 |
| Postsubiculum, layer 3 | 10 | 4.95E-05 | 0.033 | 5.2 | 51.7 | NR4A2;NR4A1;BCL11A;E2F1;TCF4;GATA3;FOS;NR3C1;KLF4; ETS2 |
| Dentate gyrus, granule cell layer | 9 | 2.61E-04 | 0.040 | 4.6 | 38.3 | NCOA1;SUZ12;UPF1;NCOR1;CBFB;MAZ;ZFPM2;CLOCK;HCFC1 |
| Entorhinal area, medial part, ventral zone, layer 3 | 9 | 2.61E-04 | 0.040 | 4.6 | 38.3 | NR4A2;YAP1;NCOR1;KLF5;CBX3;STAT3;E2F2;ETS1;JUNB |
| Medial preoptic nucleus, medial part | 9 | 2.61E-04 | 0.040 | 4.6 | 38.3 | SOX2;AR;CREB1;E2F1;E2F2;ERG;NR2F2;POU2F2;ESR1 |
| Posterolateral visual area, layer 6a | 9 | 2.61E-04 | 0.040 | 4.6 | 38.3 | NR4A1;PHC1;KLF5;CTNNB1;GATA3;FOS;NR3C1;JUNB;POU5F1 |
| Presubiculum, layer 3 | 9 | 2.61E-04 | 0.040 | 4.6 | 38.3 | NR4A2;NR4A1;SALL1;KLF5;BCL11A;TCF4;GATA3;NR3C1;ETS2 |
| Primary visual area, layer 6a | 9 | 2.61E-04 | 0.040 | 4.6 | 38.3 | NR4A1;PHC1;E2F1;ERG;GATA3;FOS;NR3C1;KLF4;KLF2 |
| anteroventral periventricular preoptic nucleus | 9 | 2.61E-04 | 0.040 | 4.6 | 38.3 | AR;ILF3;HNF4A;BCL3;NR2F2;KLF4;CLOCK;ESR1;NFE2L2 |
| layer 6a of PaS | 9 | 2.61E-04 | 0.040 | 4.6 | 38.3 | NR4A2;NR4A1;BCL11A;E2F1;TCF4;FOS;NR3C1;KLF4;ETS2 |
| layer 6b of RSC | 9 | 2.61E-04 | 0.040 | 4.6 | 38.3 | NR4A1;PHC1;NFYA;BCL11A;E2F1;CTNNB1;FOS;NR3C1;KLF4 |
| sublayer 6a of OCx | 9 | 2.61E-04 | **0.040** | 4.6 | 38.3 | STAT5A;NR4A1;E2F1;NR0B1;SIRT6;ERG;FOS;NR3C1;KLF2 |
