# Supplementary material for: Cooperation of transposable elements to endow global networks of initiators of hybrid assembly pathways of endogenous multiprotein complexes
Source: Front Cell Infect Microbiol. 2026 Jul 30;16:1793868. doi: 10.3389/fcimb.2026.1793868 (PMC13468005; doi:10.3389/fcimb.2026.1793868)
Supplement: Supplementary file 3 [file DataSheet3.zip › Expert Report Summary 215 brain regions.docx]

**Expert Report Summary: Neuroanatomical, Structural-Functional, and Developmental Analysis of 215 Brain Regions**

This report provides a comprehensive analysis of 215 brain regions, detailing their neuroanatomical features, functional roles, and developmental trajectories. The analysis is organized by the three primary embryonic brain vesicles: the Prosencephalon (Forebrain), Mesencephalon (Midbrain), and Rhombencephalon (Hindbrain).

**I. Prosencephalon (Forebrain)** The forebrain, derived from the prosencephalon, is the largest and most complex division. It includes the **Cerebral Cortex**, which forms from the dorsal pallium and is responsible for higher-order cognition, memory, and perception. Its development is characterized by an "inside-out" pattern of **radial migration**, forming its distinctive six-layered structure. The **Olfactory System**, including the Main and Accessory Olfactory Bulbs, processes scent and pheromonal information. This system exhibits a **divergent developmental trajectory** where interneurons undergo a unique form of **tangential migration** from the subventricular zone, a process that continues into adulthood. The **Septal and Basal Forebrain regions** (e.g., Lateral Septal Complex, Bed Nuclei of the Stria Terminalis) act as limbic hubs, mediating emotion, reward, and arousal. The **Hypothalamus**, a diencephalic structure, is the central control hub for homeostatic functions, including temperature, hunger, and endocrine regulation.

**II. Mesencephalon (Midbrain)** The midbrain develops from the mesencephalon. Key structures include the **Inferior Colliculus**, a critical relay for auditory processing, and the **Periaqueductal Gray (PAG)**, a central column of gray matter crucial for pain modulation and defensive behaviors. The development of the midbrain is guided by the isthmic organizer, which patterns it and the adjacent hindbrain.

**III. Rhombencephalon (Hindbrain)** The hindbrain, originating from the rhombencephalon, gives rise to the **Cerebellum** and the **Brain Stem**. The cerebellum is a key structure for motor coordination, balance, and motor learning. Its cellular architecture is defined by a three-layered cortex. The development of the cerebellum is a prime example of **developmental divergence**: while its Purkinje cells undergo **radial migration**, its numerous granule cells originate from the rhombic lip and migrate tangentially, a unique and highly specialized process. The **Brain Stem** (e.g., Parabrachial Nucleus, Dorsal Tegmental Nucleus) contains vital relays for sensory information and autonomic functions.

**Common and Divergent Developmental Trajectories**

At the highest level, all brain regions share a common origin from the embryonic **neural plate** and its subsequent folding into the neural tube. However, significant divergence occurs in later stages:

- **Migration Patterns:** The most notable divergence is in the mode of neuronal migration. While most regions are built by **radial migration** along glial fibers, a select few—like the cerebellar granule cells and olfactory bulb interneurons—utilize **tangential migration** from distinct progenitor zones.
- **Structural Lamination:** Different developmental programs lead to distinct layered architectures. The six-layered cerebral cortex, the three-layered cerebellar cortex, and the concentric layers of the olfactory bulb are all results of divergent developmental processes.
- **Functional Specialization:** These developmental and structural differences ultimately lead to highly specialized functions. The cerebellar system is optimized for precise motor control, while the cerebral cortex is configured for complex cognition and the hypothalamus for homeostatic regulation.
