# Supplementary material for: Cooperation of transposable elements to endow global networks of initiators of hybrid assembly pathways of endogenous multiprotein complexes
Source: Front Cell Infect Microbiol. 2026 Jul 30;16:1793868. doi: 10.3389/fcimb.2026.1793868 (PMC13468005; doi:10.3389/fcimb.2026.1793868)
Supplement: Supplementary file 3 [file DataSheet3.zip › 215 brain regions Revised 2_parts Report w tables.docx]

**An expert-level analysis of the neuroanatomical and structural-functional features of the 215 brain regions.**

The first part of the expert-level analysis report of the 215 brain regions include tables within each of the three main developmental sections: **Prosencephalon**, **Mesencephalon**, and **Rhombencephalon**. Each table will list the brain regions and their specific structural and functional assignments as discussed in the original report.

The second part of the report describes an expert-level analysis of the neuroanatomical and structural-functional features of the 215 brain regions, highlighting their common and divergent developmental trajectories.

**Part 1 of the Expert Report: Neuroanatomical and Structural-Functional Analysis**

This part of the report details the neuroanatomical and structural-functional features of 215 brain regions, including new tables to clearly list each region and its corresponding assignment. The analysis is organized by developmental origin to highlight common and divergent trajectories.

**I. Prosencephalon (Forebrain)**

The prosencephalon, or forebrain, is the largest and most complex division, giving rise to the cerebrum, olfactory structures, and key limbic nuclei.

**A. Cerebral Cortex and Pallium**

This section includes regions of the cerebral cortex, responsible for higher cognitive functions.

| Brain Region | Neuroanatomical/Structural-Functional Assignment |
| --- | --- |
| Cerebral cortex | Responsible for higher-order cognitive functions. |
| Cerebrum | The largest part of the brain, derived from the prosencephalon. |
| dorsal pallium/isocortex | Embryonic precursor to the cerebral cortex. |
| layer 3 of CCx | A specific layer within the cerebral cortex. |
| Postsubiculum, layer 3 | Part of the hippocampal formation, involved in spatial memory. |
| dorsal peduncular cortex, periventricular part | Part of the dorsal peduncular cortex, a limbic-related structure. |
| dorsal peduncular cortex, intermediate part | Another part of the dorsal peduncular cortex. |

**B. Olfactory System**

These regions are dedicated to the processing of olfactory and pheromonal information.

| Brain Region | Neuroanatomical/Structural-Functional Assignment |
| --- | --- |
| Main olfactory bulb | Processes general olfactory information. |
| Main olfactory bulb, glomerular layer | Layer where olfactory sensory axons synapse. |
| Main olfactory bulb, granule layer | Layer containing inhibitory interneurons. |
| Main olfactory bulb, inner plexiform layer | Synaptic layer between mitral/tufted cells and granule cells. |
| Main olfactory bulb, mitral layer | Layer containing the main output neurons of the MOB. |
| Main olfactory bulb, outer plexiform layer | Synaptic layer between tufted cells and dendrites of granule cells. |
| Accessory olfactory bulb, granular layer | Layer containing inhibitory interneurons in the AOB. |
| OB glomerular layer | General term for the glomerular layer of the olfactory bulb. |
| OB granular layer | General term for the granular layer of the olfactory bulb. |
| OB inner plexiform layer | General term for the inner plexiform layer of the olfactory bulb. |
| OB mitral cell layer | General term for the mitral cell layer of the olfactory bulb. |
| OB outer plexiform layer | General term for the outer plexiform layer of the olfactory bulb. |
| olfactory bulb, principal part | Main part of the olfactory bulb. |
| olfactory areas | General term for olfactory-related regions. |
| anterior olfactory area, ventral part | Part of the secondary olfactory cortex. |
| mantle zone of AOB | A developmental zone of the AOB. |
| mantle zone of OB | A developmental zone of the OB. |
| superficial stratum of AOB | A superficial developmental layer of the AOB. |
| superficial stratum of OB | A superficial developmental layer of the OB. |
| intermediate stratum of OB | An intermediate developmental layer of the OB. |
| periventricular stratum of AOB | A periventricular developmental layer of the AOB. |
| periventricular stratum of OB | A periventricular developmental layer of the OB. |

**C. Septal and Basal Forebrain**

These are subpallial regions involved in emotion, memory, and reward.

| Brain Region | Neuroanatomical/Structural-Functional Assignment |
| --- | --- |
| Lateral septal complex | A group of interconnected nuclei in the septum. |
| Lateral septal nucleus | Part of the lateral septal complex. |
| Lateral septal nucleus, ventral part | A specific part of the lateral septal nucleus. |
| Lateral septal nucleus, rostral (rostroventral) part | A specific part of the lateral septal nucleus. |
| lateral septal nucleus, intermedio-dorsal part | A specific part of the lateral septal nucleus. |
| Septofimbrial nucleus | A nucleus of the septal area. |
| triangular septal nucleus | A nucleus of the septal area. |
| Septohypothalamic nucleus | Connects the septum and hypothalamus. |
| Septopallidal core nucleus | Connects the septum and pallidum. |
| Bed nuclei of the stria terminalis, anterior division | A group of nuclei in the extended amygdala. |
| Bed nuclei of the stria terminalis, anterior division, anteromedial area | A specific part of the anterior BNST. |
| Bed nuclei of the stria terminalis, anterior division, rhomboid nucleus | A specific part of the anterior BNST. |
| Bed nuclei of the stria terminalis, posterior division, principal nucleus | A specific part of the posterior BNST. |
| bed nucleus of the stria terminalis, laterocentral division | A specific division of the BNST. |
| bed nucleus of the stria terminalis, medioseptal division | A specific division of the BNST. |
| nucleus of the stria terminalis, medial division, anterior part | A specific part of the nucleus of the stria terminalis. |
| substantia innominata/basal nucleus | A basal forebrain cholinergic center. |
| Pallidum | Part of the basal ganglia. |
| pallidal septum | Part of the septum with pallidal characteristics. |

**II. Mesencephalon (Midbrain)**

The midbrain contains key auditory and motor-related structures.

**A. Midbrain Structures**

This section lists regions involved in auditory and motor functions.

| Brain Region | Neuroanatomical/Structural-Functional Assignment |
| --- | --- |
| Midbrain, motor related | General area of the midbrain involved in motor control. |
| Inferior colliculus, dorsal nucleus | A specific nucleus within the inferior colliculus. |
| Inferior colliculus | A major auditory relay station. |
| Periaqueductal gray | A central gray matter column for pain modulation. |
| CoPV part of the periaqueductal gray | A specific part of the PAG. |
| JcPV part of the periaqueductal gray | A specific part of the PAG. |

**III. Rhombencephalon (Hindbrain)**

The hindbrain gives rise to the pons, medulla, and the cerebellum.

**A. Cerebellum**

The cerebellum is a critical center for motor coordination, balance, and learning.

| Brain Region | Neuroanatomical/Structural-Functional Assignment |
| --- | --- |
| Cerebellum | The main structure for motor coordination. |
| Cerebellar cortex | The layered cortex of the cerebellum. |
| cerebellar hemisphere | The lateral parts of the cerebellum. |
| cerebellar vermis | The medial part of the cerebellum. |
| cortex of cerebellar hemisphere | The cortex of the lateral cerebellum. |
| cortex of cerebellar vermis | The cortex of the medial cerebellum. |
| cerebellar nuclei of CbV | Deep cerebellar nuclei. |
| Fastigial nucleus | A deep cerebellar nucleus involved in axial control. |
| medial (fastigial) cerebellar nucleus | Another term for the fastigial nucleus. |
| Flocculus | Part of the flocculonodular lobe, for balance and eye movements. |
| Nodulus (X) | Part of the flocculonodular lobe. |
| Uvula (IX) | A lobule of the cerebellar vermis. |
| Ansiform lobule | A lobule of the cerebellar hemisphere. |
| Central lobule | A lobule of the cerebellar vermis. |
| Pyramus (VIII) | A lobule of the cerebellar vermis. |
| Simple lobule | A lobule of the cerebellar vermis. |
| Culmen | A lobule of the cerebellar vermis. |
| Declive (VI) | A lobule of the cerebellar vermis. |
| Folium-tuber vermis (VII) | A lobule of the cerebellar vermis. |
| Crus 1 | A lobule of the cerebellar hemisphere. |
| Crus 2 | A lobule of the cerebellar hemisphere. |
| Paraflocculus | A lobule of the cerebellar hemisphere. |
| Paramedian lobule, molecular layer | The molecular layer of the paramedian lobule. |
| Lobules IV-V | A group of lobules in the cerebellar vermis. |
| Lobule II | A lobule of the cerebellar vermis. |
| Lobule III | A lobule of the cerebellar vermis. |
| Purkinje cell layer of CbHCx | The single-cell layer of Purkinje neurons in the hemisphere. |
| Purkinje cell layer of CbVCx | The single-cell layer of Purkinje neurons in the vermis. |
| Flocculus, molecular layer | The molecular layer of the flocculus. |
| Flocculus, granular layer | The granular layer of the flocculus. |
| Nodulus (X), granular layer | The granular layer of the nodulus. |
| Uvula (IX), granular layer | The granular layer of the uvula. |
| Uvula (IX), molecular layer | The molecular layer of the uvula. |
| Pyramus (VIII), granular layer | The granular layer of the pyramus. |
| Pyramus (VIII), molecular layer | The molecular layer of the pyramus. |
| Crus 1, granular layer | The granular layer of Crus 1. |
| Crus 1, molecular layer | The molecular layer of Crus 1. |
| Crus 2, granular layer | The granular layer of Crus 2. |
| Crus 2, molecular layer | The molecular layer of Crus 2. |
| Lobules IV-V, granular layer | The granular layer of lobules IV-V. |
| Lobules IV-V, molecular layer | The molecular layer of lobules IV-V. |
| Lobule II, granular layer | The granular layer of lobule II. |
| Lobule II, molecular layer | The molecular layer of lobule II. |
| Lobule III, granular layer | The granular layer of lobule III. |
| Lobule III, molecular layer | The molecular layer of lobule III. |
| Declive (VI), granular layer | The granular layer of the declive. |
| Declive (VI), molecular layer | The molecular layer of the declive. |
| Folium-tuber vermis (VII), granular layer | The granular layer of the folium-tuber vermis. |
| Folium-tuber vermis (VII), molecular layer | The molecular layer of the folium-tuber vermis. |
| Simple lobule, granular layer | The granular layer of the simple lobule. |
| Simple lobule, molecular layer | The molecular layer of the simple lobule. |
| Paraflocculus, molecular layer | The molecular layer of the paraflocculus. |
| internal granular layer of CbHCx | The granular layer of the cerebellar hemisphere. |
| internal granular layer of CbVCx | The granular layer of the cerebellar vermis. |
| molecular layer of CbHCx | The molecular layer of the cerebellar hemisphere. |
| molecular layer of CbVCx | The molecular layer of the cerebellar vermis. |
| cerebellar white matter | The deep white matter of the cerebellum. |
| white matter of cerebellar vermis | The deep white matter of the cerebellar vermis. |

**B. Brain Stem and Other Hindbrain Regions**

This section lists structures involved in vital sensory and motor relays.

| Brain Region | Neuroanatomical/Structural-Functional Assignment |
| --- | --- |
| Brain stem | The part of the brain connecting the cerebrum to the spinal cord. |
| Dorsal tegmental nucleus | Part of the reticular formation. |
| Central linear nucleus raphe | A midline raphe nucleus involved in serotonin production. |
| Parabrachial nucleus, lateral division | A relay for taste and visceral sensations. |
| Parabrachial nucleus, lateral division, central lateral part | A specific part of the lateral parabrachial nucleus. |
| Parabrachial nucleus, lateral division, dorsal lateral part | A specific part of the lateral parabrachial nucleus. |
| Parabrachial nucleus, lateral division, external lateral part | A specific part of the lateral parabrachial nucleus. |
| Parabrachial nucleus, lateral division, superior lateral part | A specific part of the lateral parabrachial nucleus. |
| Cochlear nucleus, subpedunclular granular region | A region of the cochlear nucleus, the first auditory relay. |
| rhombomere 1 | An embryonic segment that gives rise to the cerebellum. |

**Summary of Common and Divergent Trajectories**

The tables highlight the commonality of developmental origins from the neural plate, but also the significant divergences in neurogenesis and migration patterns, which ultimately lead to the varied and specialized structures and functions of these 215 brain regions.

**Part 2: An expert-level analysis of the neuroanatomical and structural-functional features of the 215 brain regions, highlighting their common and divergent developmental trajectories.**

**Developmental Trajectories and Brain Region Groupings**

The brain regions listed originate from three primary developmental vesicles: the **prosencephalon** (forebrain), **mesencephalon** (midbrain), and **rhombencephalon** (hindbrain). Their mature structures and functions are direct outcomes of their embryonic origins, including the **neural plate** and its subsequent folding to form the **neural tube**.

- **Prosencephalon (Forebrain):** This is the largest and most complex division, giving rise to the telencephalon and diencephalon. The list heavily features **telencephalic structures**, including the **cerebral cortex**, **olfactory bulbs**, and **septal nuclei**.
- **Mesencephalon (Midbrain):** This region develops into structures like the **inferior colliculus** and parts of the **periaqueductal gray**, which are crucial for auditory and motor-related functions, respectively.
- **Rhombencephalon (Hindbrain):** This part gives rise to the metencephalon and myelencephalon. The list is rich in **rhombencephalic structures**, particularly the **cerebellum** and its various lobules, as well as components of the **brain stem** like the **parabrachial nucleus** and **dorsal tegmental nucleus**.

**Neuroanatomical and Structural-Functional Analysis**

**1. Cerebellum and Related Structures**

The cerebellum, which originates from the rhombomere 1 (r1) alar plate, is a focal point of this list. Its structural organization is highly conserved across species.

- **Anatomy:** The cerebellum is divided into the **vermis** (medial), **cerebellar hemispheres** (lateral), and the **flocculonodular lobe** (inferior). Each part is composed of three main layers: the **molecular layer** (outermost), the **Purkinje cell layer** (middle), and the **granular layer** (innermost). The list includes numerous granular and molecular layers of specific lobules (e.g., **Crus 1**, **Culmen**, **Declive**), highlighting their fine-grained cytoarchitectural differences.
- **Cell Groups:** The **Purkinje cells**, the sole output neurons of the cerebellar cortex, are organized into a single layer and project to the **cerebellar nuclei** (**Fastigial nucleus**, **medial cerebellar nucleus**). The granular layer is densely packed with **granule cells**, which are the most numerous neurons in the brain and form excitatory synapses onto Purkinje cells.
- **Function:** This group is primarily responsible for motor coordination, posture, balance, and motor learning. The **vermis** and **fastigial nucleus** are involved in trunk and proximal limb movements, while the **hemispheres** and **interposed/dentate nuclei** are crucial for skilled, voluntary movements. The **flocculus** and **nodulus** are key for oculomotor control and vestibular reflexes.
- **Developmental Trajectory:** The cerebellum's development is a highly orchestrated process. Granule cell precursors originate from the **rhombic lip** and migrate tangentially to form the external granular layer before migrating inward. Purkinje cells, on the other hand, are born in the ventricular zone and migrate radially. This dual-source migration is a key divergent developmental feature from other cortical structures.

**2. Olfactory System**

The olfactory system is represented by the **Main Olfactory Bulb (MOB)** and the **Accessory Olfactory Bulb (AOB)**. These structures develop from the anterior telencephalic vesicle.

- **Anatomy:** Both MOB and AOB exhibit a layered organization. The **MOB** includes the **glomerular layer** (synapses between olfactory receptor neurons and mitral cells), **mitral cell layer** (output neurons), and the **granule layer** (inhibitory interneurons). Similar layers are found in the **AOB**. The list also includes the **anterior olfactory area, ventral part**, which is a secondary olfactory cortex.
- **Cell Groups:** Key cell types are the **mitral cells** (principal projection neurons) and **granule cells** (GABAergic interneurons).
- **Function:** The **MOB** processes signals for the sense of smell. The **AOB** processes pheromonal information crucial for social and reproductive behaviors.
- **Developmental Trajectory:** The olfactory bulbs develop as evaginations of the **telencephalic vesicle**. Olfactory bulb interneurons, including granule cells, are born in the **rostral migratory stream** and migrate tangentially into the olfactory bulb, a unique form of neurogenesis that persists into adulthood.

**3. Septal and Pallidal Regions**

The **septum** and **pallidum** are part of the basal forebrain, developing from the **subpallium**.

- **Anatomy:** The **septal complex** includes nuclei like the **lateral septal nucleus** and **septofimbrial nucleus**. The **pallidum** includes structures like the **substantia innominata/basal nucleus**.
- **Cell Groups:** These regions contain a diverse mix of cell types, including cholinergic neurons, which are critical for arousal and attention, as well as GABAergic neurons.
- **Function:** The septal nuclei are involved in memory, emotion, and reward, with reciprocal connections to the hippocampus and hypothalamus. The pallidum, particularly the **substantia innominata**, is a key component of the basal ganglia motor and limbic circuits.
- **Developmental Trajectory:** These regions develop from the ventral part of the telencephalon (the **subpallium**), a common developmental trajectory that gives rise to the basal ganglia and related structures. The migration of neurons is primarily radial from the ventricular zone.

**4. Hypothalamus and Related Forebrain Structures**

The hypothalamus originates from the diencephalon. The list includes the **anterior hypothalamic nucleus** and **lateral preoptic area**.

- **Anatomy & Function:** These regions are central to regulating homeostatic functions, including body temperature, thirst, hunger, and endocrine control. They are closely linked with the **Bed nuclei of the stria terminalis** (BNST), which are considered part of the extended amygdala and play a role in stress and anxiety.
- **Developmental Trajectory:** The hypothalamus develops from the ventral diencephalon and is critical for coordinating autonomic and neuroendocrine responses. The BNST develops from a similar region as the amygdala and septum, reflecting its role as a limbic hub.

**5. Brain Stem and Midbrain Structures**

The brain stem, including the midbrain, pons, and medulla, develops from the mesencephalon and rhombencephalon.

- **Anatomy:** Structures like the **inferior colliculus** and **periaqueductal gray (PAG)** are well-represented. The **PAG** has distinct parts (e.g., **CoPV**, **JcPV**) that regulate different functions. The **parabrachial nucleus** and **dorsal tegmental nucleus** are key pontine structures.
- **Function:** The **inferior colliculus** is a major relay for auditory pathways. The **PAG** is crucial for pain modulation and a variety of defensive and vocalization behaviors. The **parabrachial nucleus** is a key relay for taste and visceral sensory information.
- **Developmental Trajectory:** These structures develop from the roof and floor plates of the embryonic brain stem. Neuronal migration is primarily radial, forming distinct nuclei. This development is influenced by a different set of signaling molecules (e.g., Wnt signaling) compared to the forebrain.

**Common and Divergent Features**

- **Commonality:** A major commonality is the **neural plate** origin and its differentiation into the neural tube. All regions listed are derived from the neural tube, and their development is guided by similar principles of gene expression gradients and signaling molecules. The formation of layered structures, such as those found in the cortex and cerebellum, is a recurring theme.
- **Divergence:**
  - **Migration Patterns:** A primary divergence is the mode of neuronal migration. Most cortical and nuclear structures are formed by **radial migration** from the ventricular zone. In contrast, the cerebellum's granule cells and olfactory bulb's interneurons rely on **tangential migration** from distinct progenitor zones, a highly divergent and specialized process.
  - **Lamination:** While many regions are layered, the specific lamination patterns and cell types differ significantly. The **cerebral cortex** has a characteristic six-layered structure, while the **cerebellum** has a three-layered structure dominated by Purkinje and granule cells. The **olfactory bulb** has a unique set of concentric layers defined by function.
  - **Functional Specialization:** The functional divergence is extreme, reflecting the different evolutionary pressures on each region. From the motor control of the cerebellum to the homeostatic regulation of the hypothalamus and the sensory processing of the olfactory bulb, each region has evolved a highly specialized structure-function relationship.

**AI assistant-generated infographic summary of the 215 human brain regions.**
