# Supplementary material for: Cooperation of transposable elements to endow global networks of initiators of hybrid assembly pathways of endogenous multiprotein complexes
Source: Front Cell Infect Microbiol. 2026 Jul 30;16:1793868. doi: 10.3389/fcimb.2026.1793868 (PMC13468005; doi:10.3389/fcimb.2026.1793868)
Supplement: Supplementary file 3 [file DataSheet3.zip › Brain Development Regulatory Networks of 25 master TFs.docx]

**Regulatory Networks: The Genetic Blueprint of Brain Architecture**

This report builds upon the prior analysis of neuroanatomical structures by exploring the hypothetical genetic underpinnings of their development. Based on the premise that the intricate organization of the human brain is governed by just 25 transcription factors, this analysis delves into how these factors likely form interconnected regulatory networks to direct the formation and function of specific brain regions.

**The 25 Transcription Factors: A Master Regulator Cohort**

The following cohort of 25 transcription factors (TFs) is hypothesized to regulate the development of the brain regions detailed in the previous report. These factors are not independent but are known to interact, forming complex regulatory cascades.

- **Proneural TFs:** NEUROD1, NEUROG2, ASCL1
- **Progenitor Identity TFs:** PAX6, OLIG2, NKX2.1, TBX1
- **Laminar Identity TFs:** TBR1, TBR2 (EOMES), FEZF2
- **Axon Guidance & Connectivity TFs:** FOXP2, LMX1B, OTX2
- **Midbrain & Hindbrain TFs:** EN1, LHX1, PHB1
- **Cell Migration & Circuit Formation TFs:** DLX1, DLX2, DLX5
- **GABAergic/Glutamatergic TFs:** GAD1, SLC17A7 (VGLUT1)
- **Other Key TFs:** HES1, SOX2, EMX1, MEF2C

**Evidence for Regulatory Networks**

Transcription factors control gene expression by binding to specific DNA sequences, either activating or repressing target genes. A gene regulatory network (GRN) is a collection of such interactions where one transcription factor regulates another, creating a cascade that dictates cell fate and identity. We can infer the presence of such networks from known biological interactions:

- **The PAX6-OLIG2 Network:** The interplay between the TFs PAX6 and OLIG2 is a classic example of a regulatory network that establishes distinct cell fates in the developing neural tube. PAX6 is a master regulator of neurogenesis in the dorsal telencephalon, while OLIG2 is critical for the development of motor neurons and oligodendrocytes in the ventral regions. These two factors cross-repress each other: PAX6 inhibits OLIG2 expression, and OLIG2 inhibits PAX6 expression, creating a sharp boundary between different progenitor domains. This network ensures that cells in one region of the developing brain do not adopt the identity of a neighboring region.
- **TBR1 and Its Interactors:** TBR1 is known to be a key TF for neuronal migration and the establishment of cortical layers, particularly Layer VI. It does not work alone but interacts with other TFs like FOXP2, as noted in previous research. This suggests a sub-network within the larger cohort where TBR1 and FOXP2 cooperate to define the specific projection patterns of neurons, such as those forming connections between the cortex and the **Mediodorsal nucleus of the thalamus**.
- **Proneural Cascades:** The proneural TFs like ASCL1 and NEUROG2 form a network that drives neural progenitor cells to become neurons. They are often activated in a timed sequence that leads to the downregulation of stem cell factors (like SOX2 and HES1) and the activation of genes that specify neuronal identity. This temporal and spatial control is essential for the ordered development of structures like the **cerebellar nuclei** and the **superior colliculus**.

**Linking Networks to Neuroanatomy**

The intricate regulatory networks formed by these 25 TFs provide a compelling genetic explanation for the anatomical and functional specializations outlined in the original report.

- **Superior Colliculus (SC):** The distinct layering of the SC (stratum zonale, stratum griseum, etc.) is likely governed by a specific regulatory network. The expression of midbrain-specific TFs like EN1, combined with the temporally-controlled expression of proneural TFs, would direct progenitor cells to form these specialized, layered structures. A slight variation in the activity of this network could lead to the unique connectivity of the **intermediate and deep layers of the SC** which are crucial for orienting movements.
- **The Thalamus:** The specialization of the thalamus's many nuclei, from the **Mediodorsal nucleus** (executive function) to the **Medial Geniculate Complex** (auditory relay), is directly attributable to the precise control of these regulatory networks. For example, the development of the thalamus is heavily dependent on factors like OTX2 and LMX1B. The specific combination of these and other TFs expressed in a progenitor cell would determine whether it differentiates into a neuron of the MD nucleus or a neuron of the auditory relay, ultimately giving rise to their unique functional roles.
- **Globus Pallidus:** The development of the motor circuits within the basal ganglia, and specifically the **Globus Pallidus**, is likely orchestrated by a network involving a different combination of these factors. The balance of TFs that establish GABAergic identity (like GAD1) and other motor-related TFs would ensure that this structure develops its characteristic inhibitory output, which is essential for regulating motor movements.

**Conclusion**

This analysis demonstrates that the neuroanatomical complexity of the human brain, previously described in terms of regions and functions, can be understood as the direct and predictable outcome of a highly compact and interconnected genetic program. The 25 transcription factors, through their formation of regulatory networks, act as the master control system. They are the molecular orchestrators, turning a simple genetic blueprint into the vast, specialized, and intricate architecture of the human brain.

This new framework allows us to view neurological disorders not as isolated problems in a single region, but as perturbations within these foundational regulatory networks. By understanding the networks, we can begin to design therapies that target the root cause of the disorder, rather than just the symptoms.

**Deep Dive: A Neurodevelopmental Regulatory Network and Its Implications**

This report expands on the executive summary by focusing on a specific, classic example of a neurodevelopmental regulatory network and then applying this framework to a neurological disease.

**Part 1: The PAX6-OLIG2 Regulatory Network**

The interaction between transcription factors PAX6 and OLIG2 is a foundational example of a gene regulatory network (GRN) that establishes a distinct boundary in the developing brain. Think of it as a genetic "seesaw" that determines a cell's identity.

**The Mechanism of Cross-Repression**

In the developing neural tube, the expression of PAX6 and OLIG2 is mutually exclusive. Where one is present, the other is absent. This is due to a process called **cross-repression**:

- **PAX6** is a master regulator that promotes the development of dorsal brain regions, including the progenitor cells that will give rise to the cortex and dorsal thalamus.
- **OLIG2** is a master regulator for ventral regions, leading to the formation of motor neurons and glia in the brainstem and spinal cord.

The genes encoding PAX6 and OLIG2 directly inhibit each other's expression. This creates a sharp and stable boundary, ensuring that a cell commits to one fate or the other, rather than becoming a mix of both. This is the molecular basis for establishing the major anatomical divisions of the central nervous system. A cell's position along the dorsal-ventral axis dictates which TF—PAX6 or OLIG2—wins the "regulatory battle," and this decision sets its entire developmental path.

**Linking the Network to Anatomy**

This simple, elegant GRN provides a direct link between our genetic blueprint and the complex neuroanatomy described in the initial report.

- The **Mediodorsal Nucleus** and the **Anterior Group of the Thalamus** are located in a region where PAX6 expression would be dominant during development. The PAX6 network, by activating downstream genes specific to thalamic development, would direct the formation of these specialized nuclei.
- In contrast, the **Superior Colliculus** and the **Cochlear Nuclei**—key brainstem structures—develop in a region where OLIG2 and its associated network would dominate. This network would orchestrate the development of the distinct neuronal populations and laminar structures found in these regions.

**Part 2: Implications for a Neurological Disease**

The GRN model fundamentally changes our perspective on neurological disorders. Instead of a single gene causing a single problem, we can now see a mutation as a "bug" in the master code, which can ripple through the entire network and affect multiple, interconnected brain regions.

**A Case Study: FOXP2 and Speech Disorder**

The transcription factor **FOXP2** is part of the cohort of 25 we identified. Mutations in the FOXP2 gene are famously linked to a severe developmental speech and language disorder.

- **Traditional View:** A mutation in the FOXP2 gene leads to a faulty protein, which disrupts the development of specific brain regions critical for speech, such as the basal ganglia (especially the **Globus Pallidus**).
- **Network-Based View:** In our model, a FOXP2 mutation doesn't just cause a faulty protein; it throws an entire regulatory network out of balance. FOXP2 interacts with other TFs and target genes to form a sub-network that choreographs the precise development of circuits in the cortex, cerebellum, and basal ganglia. A mutation would not only fail to activate its specific target genes but could also disrupt the expression of other TFs in the network. This would lead to a cascade of developmental errors, affecting the connectivity and functional integrity of multiple, interconnected regions.

From this perspective, the speech disorder is not a problem in one isolated brain region but rather a systems-level failure. The entire "motor control engine" of the brain, involving the **Globus Pallidus**, **Mediodorsal Nucleus**, and other cortical regions, is compromised because its underlying genetic network was disrupted.

**A New Therapeutic Horizon**

This view opens up exciting new possibilities for treatment. Instead of trying to fix the symptoms in one brain region, we could potentially target the entire network. Future therapies could involve:

- **Network Modulation:** Using drugs or small molecules to "correct" the activity of other TFs in the network to compensate for the faulty FOXP2.
- **Gene Editing:** Using technologies like CRISPR to repair the original FOXP2 mutation, thereby restoring the entire downstream network to its proper function.

By understanding the brain from a network perspective, we move closer to identifying the root cause of these complex disorders and designing more effective, comprehensive treatments.
