# Supplementary material for: Cooperation of transposable elements to endow global networks of initiators of hybrid assembly pathways of endogenous multiprotein complexes
Source: Front Cell Infect Microbiol. 2026 Jul 30;16:1793868. doi: 10.3389/fcimb.2026.1793868 (PMC13468005; doi:10.3389/fcimb.2026.1793868)
Supplement: Supplementary file 4 [file DataSheet4.zip › Functional Neuroanatomy of 100 Human Core Brain Regions.docx]

**Functional Neuroanatomy of 100 Human-Specific Core Brain Regions: An Expert-Level Analysis**

**Executive Summary**

This report provides an in-depth analysis of the functions of a diverse collection of brain regions, synthesizing a complex array of neuroanatomical and physiological data into a coherent functional framework. The investigation reveals a sophisticated and interconnected system of neural networks responsible for innate behavior, cognitive control, motor execution, and homeostatic regulation. A key finding is the profound functional specialization at the sub-regional and microcircuit levels, where discrete cell populations and layered architectures perform highly specific computational tasks. The analysis highlights that traditional views of certain brain regions as simple relays are incomplete, as they actively modulate and shape information flow to orchestrate complex behaviors. Major themes emerging from this synthesis include the amygdala's role as a nexus for innate emotional and social responses to chemosignals, the thalamus as a dynamic cortical partner for executive function, and the superior colliculus as a rapid sensory-motor transformation hub. The report also illuminates a critical link between psychological stress centers and physiological homeostatic systems, suggesting a novel circuit for understanding stress-related diseases.

**Key Thematic Insights**

The analysis is structured around four major functional themes that illustrate the interconnected nature of the human brain:

- **The Limbic System as a Hub for Innate Behavior:** The amygdaloid and orbitofrontal systems are not merely centers for emotion but are crucial for integrating sensory chemosignals and contextual cues to generate innate and learned emotional responses, particularly those related to social, reproductive, and defensive behaviors.^1^
- **The Thalamus as a Multifaceted Gatekeeper:** The thalamus serves a far more complex role than a simple sensory relay. Its nuclei, particularly the Mediodorsal (MD) and Reticular (TRN), function as dynamic regulators of cortical activity, orchestrating attention, executive control, and the fluid exchange of information necessary for cognitive flexibility.^5^
- **Interconnected Motor-Cognitive Loops:** The basal ganglia, including the globus pallidus and nucleus accumbens, are central to complex motor control and are deeply intertwined with cognitive processes such as decision-making, motivation, and reward perception.^9^ The superior colliculus demonstrates a direct sensory-to-motor transformation pathway, bypassing higher cortical areas for rapid orienting movements.^14^
- **Neural Circuits for Survival and Homeostasis:** Regions like the dorsomedial hypothalamus and zona incerta are revealed as key integrative nodes, linking internal metabolic states and stress responses with broader behavioral control and survival functions.^16^

**Introduction: Establishing a Functional Framework**

The objective of this report is to provide a comprehensive, expert-level overview of the neuroanatomical and functional roles of a specific set of brain regions. The selected regions represent a broad spectrum of brain functions, from foundational homeostatic regulation to high-level cognitive control. By examining each area in detail and then analyzing their interconnections, this document aims to serve as a definitive resource for advanced study. The analysis is organized to reveal a holistic picture of the brain as a set of deeply intertwined networks rather than isolated structures.

To manage the extensive list of regions, a functional and anatomical organizational structure has been adopted. The regions are grouped into four major themes that reflect their primary roles and connections. This approach facilitates a deeper understanding of how these structures work in concert to produce complex behaviors and maintain internal stability. The following table provides a conceptual guide to the report’s structure.

| Functional System & Themes | Associated Brain Regions |
| --- | --- |
| **I. The Limbic and Cortical Network** *Emotion, decision-making, and olfactory processing.* | Medial amygdalar nucleus (all parts), Cortical amygdalar area, Striatum-like amygdalar nuclei, Subpallial amygdala, Orbital area (all parts), Anterior olfactory nucleus (all parts), Accessory olfactory bulb (all parts), Infralimbic area. |
| **II. The Thalamic System** *Sensory relay and cognitive integration.* | Mediodorsal nucleus (all parts), Ventral posterolateral nucleus, Reticular nucleus, Parataenial nucleus, Interanterodorsal nucleus, Prethalamus, Zona incerta, Nucleus of the stria medullaris (prethalamic), Interbrain. |
| **III. The Motor Systems and Brainstem Hubs** *Movement, reflexes, and sensorimotor transformation.* | Superior colliculus (all parts), Globus pallidus (all segments), Pallidum (dorsal region), Bed nucleus of the anterior commissure, Pedunculopontine nucleus, Cuneiform nucleus, Spinal nucleus of the trigeminal, Brain stem, Midbrain, Nucleus x. |
| **IV. Homeostatic and Developmental Systems** *Regulation of internal states and foundational neuroanatomy.* | Dorsomedial nucleus of the hypothalamus (all parts), Hindbrain, Anterior nuclei, Anterior olfactory area, Core of P-DMH, Dorsal premammillary nucleus, Lateral pallium, Nucleus of the stria medullaris, Perireunensis nucleus, Prethalamic eminence, Prosomere 3, Reticular complex, Reticular nucleus, Subgeniculate nucleus, Upper dorsal lateral hypothalamic area. |

**The Limbic and Cortical Network: Emotion, Decision-Making, and Olfactory Processing**

**The Amygdaloid Complex: A Hub for Innate Behavior**

The amygdala is a heterogeneous structure, not a single entity, composed of a complex array of nuclei that are fundamental for innate emotional behaviors, social interactions, and chemosensory processing.^1^ The medial nucleus of the amygdala (MeA) serves as a central hub in this system, generating emotional responses to chemosignals and relaying olfactory information to hypothalamic nuclei that control reproductive and defensive behaviors.^1^

Functional segregation is evident within the MeA. The posterodorsal part (MePD) is predominantly involved in reproductive behaviors.^21^ This sub-region is distinguished by its high density of androgen and estrogen receptors, which makes it particularly sensitive to hormonal inputs that modulate social behavior.^21^ The MePD is primarily activated by sexually related chemosignals and sends projections to structures that control reproductive functions, such as the medial preoptic nucleus and the medial posteromedial bed nucleus of the stria terminalis.^22^ Conversely, the posteroventral part (MePV) is primarily dedicated to defensive behaviors.^22^ It exhibits a strong and preferential response when an organism is exposed to predator odors, such as those from a cat, and projects to defensive-related nuclei like the medial posterointermediate bed nucleus of the stria terminalis and the anterior hypothalamic area.^22^

While specific functions for sublayers a, b, and c of the MePD are not detailed in the available literature, an important finding regarding the MePV’s microcircuitry offers a compelling glimpse into the underlying mechanisms of amygdala function. A class of GABAergic inhibitory interneurons (Type 2) in the MePV provides feedforward inhibition within the olfactory-processing circuit.^1^ This microcircuitry is not merely a passive relay of information; it is an active mechanism that refines the signal. By providing feedforward inhibition, these interneurons act as a filter, sharpening the neural signal and ensuring that only the most potent and behaviorally relevant chemosignals are transmitted to the hypothalamus. This inhibitory control prevents signal over-stimulation and enables a more precise and nuanced behavioral response, allowing an organism to differentiate subtle cues with significant survival implications.

The amygdala's functional architecture is further clarified by its developmental origins. Structures such as the striatum-like amygdalar nuclei and the subpallial amygdala are characterized by their subpallial lineage and their predominantly inhibitory, GABAergic nature.^2^ These structures represent the "output pole" of the amygdala's behavioral and emotional responses, analogous to the striatum's role as the main input structure of the basal ganglia.^2^ This organizational principle suggests a division of labor where the pallial amygdala is the "perceptual end" for processing sensory input, while the subpallial components are responsible for generating and executing the motor and behavioral outputs of the system.^2^

**The Orbitofrontal Cortex (OFC): Reward, Decision, and Emotion**

The orbitofrontal cortex (OFC) is a critical cortical region involved in emotion, the representation of reward value, and decision-making.^26^ It integrates highly processed sensory information from various modalities—including taste, smell, and vision—and links it to reward-related value.^26^

A key functional principle of the OFC is its spatial segregation of function. The lateral part of the OFC is associated with processing non-reward and punishment and is crucial for learning contingency changes and encoding new expectations about aversive outcomes.^26^ In contrast, the medial part of the OFC is activated by rewarding and pleasant stimuli and represents different types of reward.^26^ This segregation creates a sophisticated, dual-control system for behavioral regulation. The medial part of the OFC drives approach and reinforcement by signaling rewards, while the lateral part acts as a crucial "braking" mechanism, signaling a lack of reward or a negative outcome to facilitate the reversal of behavior. This push-pull dynamic is essential for flexible and adaptive behavior, such as adjusting a strategy when an expected reward fails to materialize. The ventrolateral prefrontal cortex (VLPFC), a region functionally related to the lateral OFC, is also implicated in executive functions, working memory, and the selection of perceptual information for goal-oriented actions.^28^

At the laminar level, Layer 1 (L1) of the neocortex, which is conserved across cortical areas, serves as the primary target for top-down signals.^31^ This layer is crucial for integrating contextual, attentional, and emotional information with bottom-up sensory input from deeper cortical layers. The apical dendrites of projection neurons in layers 2/3 and 5 extend into L1, where they receive these top-down inputs, allowing for state-dependent and flexible processing of sensory information. In the infralimbic area, neurons in Layer 2/3 are involved in emotional memory and display distinct intrinsic excitability properties, which is likely related to their specialized function.^32^

**The Olfactory System: From Perception to Behavior**

Unlike other sensory systems, primary olfactory information bypasses the thalamus and is distributed directly to the olfactory cortex.^34^ The anterior olfactory nucleus (AON) acts as a central coordinating hub, distributing odor information to both the ipsilateral and contralateral olfactory bulbs and the piriform cortex.^34^ The AON’s role extends beyond a simple relay; it actively facilitates the localization of odor sources. Neurons in the AON pars externa can computationally compare and subtract odor inputs from the two nostrils to determine the direction of a scent, a precise function for directional localization.^34^

The accessory olfactory bulb (AOB) is the first integrative center for the vomeronasal system, which processes non-volatile chemosignals like pheromones and kairomones that are critical for species-specific social and reproductive behaviors.^38^ Within its distinct laminar organization, the outer plexiform layer serves as the site of the first synapse in this pathway.^39^ Here, vomeronasal sensory neurons form glutamatergic synapses with the projection neurons of the AOB, the mitral cells. Inhibitory juxtaglomerular interneurons in this layer release GABA to modulate and balance this synaptic transmission, ensuring signal integrity.^39^ The intermediate stratum is part of this layered structure ^42^ and contributes to the overall processing of chemosensory signals that are vital for an organism's survival and social interactions.

**The Infralimbic Area (IL): Fear Extinction and Behavioral Adaptation**

The infralimbic (IL) cortex, a subregion of the medial prefrontal cortex (mPFC), is a key node in the brain's fear and emotion processing network.^32^ It plays a critical role in the extinction of conditioned fear memories, effectively acting as an "off-switch" for a learned fear response.^32^ The IL receives extensive synaptic input from various brain regions, including the amygdala and hypothalamus, and projects to these same subcortical structures to modulate emotional behaviors and homeostatic processes.^32^

An analysis of the neuronal properties within this region reveals a layer-specific specialization. Neurons in Layer 2 (L2) of the IL, specifically the cortical-pyramidal (CP) neurons, are a class of "non-resonating" cells.^32^ This distinguishes them from other neuronal populations and is a crucial part of their functional profile. The non-resonating nature of these cells, combined with their enhanced intrinsic excitability compared to neurons in the neighboring prelimbic cortex ^33^, suggests a direct link between their specific biophysical properties and the IL's function in fear extinction. The non-resonating characteristic may allow for the transmission of a more stable, non-oscillatory command to subcortical structures, which is essential for a clean and decisive inhibition of a fear response. The enhanced excitability may facilitate the rapid and robust activation of this extinction circuit when a threat is no longer present. This is a clear demonstration of how specific cellular properties at the microcircuit level are essential for enabling a complex, system-level behavior.

**The Thalamic System: Sensory Relay and Cognitive Integration**

**The Mediodorsal Nucleus (MD): Executive Function and Cognitive Control**

The mediodorsal nucleus (MD) is a higher-order thalamic nucleus with robust, reciprocal interconnections with the prefrontal cortex (PFC), a partnership that is fundamental for executive functions such as planning, cognitive control, working memory, and decision-making.^5^

The lateral part of the MD is particularly critical in this partnership. It works in close collaboration with the frontal cortex during "rapid cognitive operations" such as learning new information, making quick decisions, or planning the next move.^5^ This is a far more sophisticated role than a simple sensory relay. The MD seems to be involved in a "forward modeling" process, using "efference copies" of cortical messages to anticipate and adjust to rapid cognitive demands.^5^ It enhances cognitive flexibility and increases robustness to task uncertainty, which are high-level computational functions that allow for adaptive and flexible behavior in changing environments.

The query also mentions the lateral (parvicellular) part of MD. While specific details on this sub-region are limited, a broader analysis of other thalamic parvicellular nuclei provides a compelling parallel. For example, the parvicellular part of the ventral posteromedial nucleus (VPMpc) functions as a separate relay for gustatory information, distinct from the VPM's primary somatosensory role.^50^ This suggests a general principle of thalamic organization where parvicellular sub-regions have specialized, non-primary relay functions. It is thus plausible to infer that the lateral parvicellular part of the MD may also have a specialized, independent role within the broader context of MD function, possibly in integrating specific types of limbic or non-sensory information into its partnership with the PFC.

**The Ventral Posterolateral Nucleus (VPL): Somatosensory Relay**

The ventral posterolateral nucleus (VPL) is a major subdivision of the thalamus that serves as a critical relay station for somatosensory information from the body to the primary somatosensory cortex.^51^ It receives sensory inputs from two main pathways: the spinothalamic tract, which carries information about pain and temperature, and the medial lemniscus, which relays proprioception and fine touch.^51^ This nucleus is organized somatotopically, meaning different parts of the body are represented in distinct areas of the nucleus.^52^

The query also lists the parvicellular part of the VPL. As with the MD's parvicellular part, the available research does not provide a specific functional description for this region. However, a pattern of functional segregation is observed in the neighboring ventral posteromedial nucleus (VPM). The parvicellular part of the VPM (VPMpc) is not involved in somatosensory relay; instead, it serves as a relay for taste sensations.^50^ This parallel structure suggests that within the VPL, a parvicellular sub-region may also perform a distinct and specialized function separate from its primary somatosensory role. This principle of functional differentiation within morphologically similar sub-regions underscores the intricate organizational complexity of the thalamus.

**Other Key Thalamic Nuclei**

The **Reticular Nucleus of the Thalamus (TRN)** is a shell of GABAergic neurons strategically located between the thalamus and the cortex.^7^ It is often described as the "guardian of the gateway," as it receives inputs from both the cortex and subcortical regions and provides the primary inhibition to thalamocortical neurons.^7^ This position allows the TRN to regulate the flow of information passing through the thalamus, playing a key role in selective attention during wakefulness and controlling brain oscillations during sleep.^8^

The **Parataenial nucleus** is a component of the midline thalamic group. Its extensive connectivity with the brainstem, hypothalamus, and limbic system suggests that it acts as a gateway for multimodal information to reach the limbic system, where it can be used to select appropriate behavioral responses.^54^ The nucleus is also implicated in the retrieval of consolidated fear memory.^54^ The Interanterodorsal nucleus of the thalamus is also part of this midline group, which is broadly known for integrating motivational and homeostatic information to influence the cortex.^55^

**The Prethalamus and its Sub-regions**

The prethalamus (also known as the ventral thalamus) is a collection of nuclei, including the **Zona incerta**, that provide long-range inhibitory control over a wide range of behaviors.^18^ The zona incerta functions as a behavioral "switchboard," integrating sensory and non-sensory information to promote and execute specific behavioral strategies such as sleep, circadian rhythms, and feeding.^18^ A subset of its GABAergic cells co-express dopamine and project to motor-related regions, suggesting a role in fine-tuning motor control.^56^ The provided data on the intermediate stratum of PHyB-I is insufficient to establish its functional role in the context of neuroanatomy, as the relevant research material pertains to plant biology.^22^ The Nucleus of the stria medullaris (prethalamic) is part of a tract that conveys fibers from the septal, striatal, and hypothalamic areas to the habenula, providing a crucial pathway for limbic and motivational information to influence downstream targets.^59^ The terms alar plate of p3, prethalamic eminence, and others are developmental terms that refer to the embryonic origins of the diencephalon, including the prethalamus and thalamus.^44^

**The Motor Systems and Brainstem Hubs**

**Basal Ganglia: Motor and Non-Motor Control**

The basal ganglia constitute a vital network for controlling both motor and non-motor functions, including motor generation, motor learning, and value-based decision-making.^9^ The globus pallidus is a central component of this circuit. The

**Globus pallidus, external segment (GPe)**, is a dynamic and complex information hub, not a simple relay station as once thought.^9^ It processes motor and non-motor information and has extensive, complex connections with other basal ganglia nuclei, the cortex, thalamus, and pedunculopontine nucleus.^9^ The

**Globus pallidus, internal segment (GPi)**, is the primary output nucleus of the basal ganglia.^13^ Its main function is to provide tonic inhibition to the thalamus, specifically the ventral lateral and ventral anterior nuclei, to restrict the initiation of movement and prevent unwanted motions.^12^ It integrates information from the striatum, GPe, and subthalamic nucleus.^13^

The relationship between the GPe and GPi is a core principle of motor control. According to the classic model, a direct pathway "accelerates" movement, while an indirect pathway, which involves the GPe, acts as a "concurrent 'brake'".^9^ The GPi, as the final output, provides a constant inhibitory signal that must be momentarily released to allow a movement to occur. This push-pull mechanism, with the GPi's tonic inhibition as the ultimate arbiter, is a fundamental system that prevents chaotic and uncontrolled movements. The

**Accumbens nucleus, shell domain**, a part of the ventral striatum, is strongly associated with the limbic system and is a key part of the brain's circuitry for reward-related behavior and motivation. It receives glutamatergic projections from the basolateral amygdala, and the activation of these projections is known to drive motivational salience.^3^

**The Superior Colliculus (SC): From Sensation to Orienting Action**

The superior colliculus (SC) is a remarkable sensorimotor structure that transforms sensory signals into rapid orienting behaviors of the eyes, head, and body.^14^ Its laminar structure is organized to integrate visual, auditory, and somatosensory information from various sources.^63^

The motor-related functions are primarily located in the intermediate and deep layers of the SC.^65^ These layers contain a motor map that is precisely aligned with the visual map in the superficial layer, allowing for the initiation of saccades and other orienting movements toward a stimulus in the contralateral visual field.^68^ The

**Deep gray layer** and **Deep white layer** are collectively involved in these sensory-motor transformations.^63^ They contain a network of intrinsic connections that modulate and coordinate the output of efferent neurons, and they also participate in the detection of "looming motion" to trigger defensive behaviors.^63^ Beyond exteroceptive senses, the deep layers also integrate interoceptive signals related to affective experiences, linking them with autonomic control via connections to the periaqueductal gray (PAG).^64^ This ability to integrate diverse sensory and internal signals into a single, coordinated motor command is a testament to the SC's foundational role in survival, providing a rapid, sub-cortical pathway for orienting to a stimulus, whether it is a threat, a conspecific, or a food source. The

**Intermediate gray layer** and **Intermediate white layer** are also crucial for sensorimotor integration and initiating orienting movements, although the provided research does not provide a specific functional distinction between them.^65^

**Other Motor and Sensorimotor Hubs**

The **Pedunculopontine nucleus (PPN)** and the adjacent **Cuneiform nucleus** together form the mesencephalic locomotor region (MLR).^69^ The PPN is involved in controlling locomotion, muscle tone, sleep-wake cycles, and cortical arousal.^69^ It receives inputs from the motor cortex, globus pallidus, and substantia nigra, and sends descending projections to the brainstem and spinal cord to control movement.^69^ The

**Spinal nucleus of the trigeminal, interpolar part**, is a nucleus within the brainstem that serves as a sensory pathway for the face.^71^ The interpolar subnucleus is specifically responsible for transmitting tactile sense and dental pain from the orofacial region.^72^

**Homeostatic and Developmental Systems**

**The Hypothalamic Nuclei: Regulating Survival**

The hypothalamus is a critical region for regulating vital body functions, and the **Dorsomedial nucleus of the hypothalamus (DMH)** is a key component.^16^ It is involved in feeding, drinking, body-weight regulation, and the expression of circadian rhythms.^16^ The DMH is also recognized as a "stress center," driving cardiovascular responses, such as increases in heart rate and blood pressure, during stressful events.^17^

The ventral part of the DMH receives information about feeding cues and circadian rhythms and transmits this information to other brain regions to regulate wakefulness.^17^ It is a component of a psychosomatic neural pathway from the medial prefrontal cortex that drives physiological responses to psychological stress, including hyperthermia.^17^ A significant new understanding of this system comes from the discovery of a neural circuit connecting the medial amygdala to the liver, routed through the hypothalamus.^74^ This circuit provides a direct link between psychological stress and physiological responses like increased blood glucose. This challenges the previous notion that the amygdala is solely for emotional response, demonstrating that emotional centers can directly "command" homeostatic organs. The DMH is likely a key hub in this pathway, potentially contributing to stress-related diseases such as type 2 diabetes.^74^

**Other Regions of Foundational Importance**

The **Brain stem** and **Hindbrain** are foundational structures that connect the cerebrum to the spinal cord and cerebellum. They are responsible for vital, unconscious life functions, including breathing, heart rate, blood pressure, and sleep.^75^ The brainstem, composed of the midbrain, pons, and medulla oblongata, houses numerous critical nuclei and tracts that relay both ascending (afferent) and descending (efferent) signals.^76^ The **Midbrain** is a component of the brainstem that is involved in motor-related functions ^75^, housing structures like the superior colliculus and pedunculopontine nucleus. The **Interbrain** is a broad anatomical term for the diencephalon, which includes the thalamus and hypothalamus, encompassing many of the regions discussed in this report.^77^ The **Pallidum, dorsal region**, is the anatomical location for the globus pallidus, which has been analyzed in detail.^79^ The **Lateral pallium** is a developmental and evolutionary term. In fish, it is essential for spatial learning, while in mammals, it is the progenitor of the piriform cortex, which has a primary olfactory function.^80^

The query also lists several structures with developmental and laminar designations, such as alar plate of p3 and prethalamic eminence. These terms describe specific embryonic territories and strata of cell bodies and fibers within larger anatomical regions, highlighting the high degree of cellular and network specialization at a fine-grained level.^44^

**Conclusion**

The analysis of these diverse brain regions reveals a highly integrated and functionally specialized neuroanatomical architecture. The brain's systems are not isolated but are engaged in dynamic, reciprocal communication that orchestrates complex behaviors and maintains homeostasis. The Limbic and Cortical Network, anchored by the amygdala and orbitofrontal cortex, links chemosensory and emotional states to innate and learned behaviors. The Thalamic System, far from being a passive relay, acts as a dynamic gatekeeper, with nuclei like the MD actively partnering with the cortex to facilitate executive functions and cognitive flexibility. The Motor Systems and Brainstem Hubs, including the basal ganglia and superior colliculus, provide a sophisticated infrastructure for both deliberate and reflexive actions, governed by a push-pull mechanism that prevents chaotic movement.

The most profound understanding gained from this synthesis is the direct and often subtle connections between these systems. The limbic system's emotional decisions are intimately tied to the basal ganglia's motor planning and the hypothalamus's homeostatic outputs. The discovery of a direct circuit from the medial amygdala to the liver, routed through the hypothalamus, provides a new paradigm for understanding the physiological consequences of psychological stress and opens a new avenue for research into stress-related diseases. The analysis also highlights that an understanding of brain function is incomplete without a focus on the microcircuit and laminar levels, where the specific properties of individual neurons define the capabilities of entire systems.

This report confirms a foundational principle of neuroanatomy: the brain’s complexity emerges from the elegant and efficient organization of highly specialized, interconnected neural circuits. While many details about individual sub-layers and developmental strata remain to be fully elucidated, the established framework provides a clear direction for future inquiry, underscoring the continuously evolving nature of our understanding of the brain.
