# Supplementary material for: Cooperation of transposable elements to endow global networks of initiators of hybrid assembly pathways of endogenous multiprotein complexes
Source: Front Cell Infect Microbiol. 2026 Jul 30;16:1793868. doi: 10.3389/fcimb.2026.1793868 (PMC13468005; doi:10.3389/fcimb.2026.1793868)
Supplement: Supplementary file 4 [file DataSheet4.zip › Report 48 Chimpanzee brain regions Text.docx]

**Expert-level analysis of 48 Chimpanzee-specific brain development regions.**

To perform an expert-level analysis of the provided brain regions, I'll group them by function and anatomical location to provide a clear, coherent report. The regions can be broadly categorized into the following functional systems: **Visual System**, **Cerebellar System**, **Limbic System**, **Amygdalar System**, and a few additional specialized areas.

**1. Visual System Areas**

This group of regions is primarily involved in processing visual information. The analysis includes various visual cortical areas and their specific layers.

- **Primary Visual Area (V1)**: The main hub for initial visual processing, located in the occipital lobe. It receives input from the retina via the thalamus and is responsible for detecting basic features like edges, lines, and orientation. The different layers play distinct roles:
  - **Layer 3**: Part of the superficial layers involved in **cortico-cortical communication**, sending signals to other visual areas for further processing.
  - **Layers 6a and 6b**: Deep layers that project back to the thalamus, modulating the visual information received. Layer 6b is also known for its developmental role.
- **Posterolateral Visual Area (PLV) & Anterolateral Visual Area (ALV)**: These are extrastriate visual areas that process more complex visual information, such as motion and spatial relationships. They receive input from V1 and project to other higher-order visual regions.
- **Posteromedial Visual Area (PMV)**: Located near the primary visual cortex, PMV is involved in complex visual tasks and likely processes motion and peripheral visual information. The specific layers (5, 6a, 6b) are part of the hierarchical processing within the visual cortex, with layer 5 often projecting to subcortical structures and layer 6 involved in feedback loops.
- **Occipital Cortex (OCx)**: The entire lobe dedicated to visual processing.
  - **Layers 3, 4, 5, 6b**: These are the main cortical layers. Layer 4 is the primary input layer from the thalamus, while layers 2 and 3 are for cortico-cortical connections. Layers 5 and 6 are the main output layers.
  - **Mantle Zone & Superficial Stratum**: These are developmental terms. The mantle zone is a deeper layer that develops into the gray matter (cortical layers), while the superficial stratum corresponds to the outer layers (cortical plate/marginal zone) that will mature into the superficial cortical layers.
- **Retrosplenial Cortex (RSC)**: This region is a part of the limbic system but has strong visual connections. It's crucial for spatial navigation and memory, integrating visual information with self-movement and orientation. Its layers (3, 4, 5, 6, 6b) contribute to its extensive connectivity with the hippocampus and other memory-related structures.

**2. Cerebellar System Areas**

The cerebellum is critical for motor control, coordination, and procedural memory. The analysis includes regions from the cerebellar hemispheres and their specific layers.

- **Crus 1 & Crus 2**: These are major lobules within the cerebellar hemispheres. They are part of the lateral cerebellum and are primarily involved in cognitive functions, planning, and executive tasks, in addition to their traditional motor roles.
  - **Granular Layer**: This is the main input layer of the cerebellum. It contains granule cells, the most numerous neurons in the brain, which receive input from mossy fibers originating from the brainstem.

**Molecular Layer**: The outermost layer, rich in dendrites of Purkinje cells and axons of granule cells. It is the site of synaptic integration and plasticity.

- **Flocculus & Paraflocculus**: These are part of the vestibulocerebellum, which is crucial for **balance, posture, and eye movements**. Their granular layers, like in Crus 1/2, receive vestibular input.
- **Cerebellar Hemisphere (CbH)**: The large lateral parts of the cerebellum, primarily involved in planning and executing complex, skilled movements and cognitive functions. The **mantle zone** is a developmental term for the developing gray matter of the cerebellum.

**3. Limbic & Memory System Areas**

This group of regions is key for memory, emotion, and spatial navigation.

- **Presubiculum (PrS) & Postsubiculum (PaS)**: These are parts of the hippocampal formation, a crucial system for memory formation and spatial navigation. They receive input from the retrosplenial cortex and are considered part of the "Papez circuit" for memory. Their layers (3, 5, 6a, 6b) are involved in transferring information to and from the hippocampus proper.
- **Subiculum**: A key output structure of the hippocampus. The **Postsubiculum, layer 3** is a part of this output pathway.

**4. Amygdalar System & Related Areas**

The amygdala is a central hub for emotional processing, particularly fear and anxiety.

- **Medial Amygdalar Nucleus, posterodorsal part**: This specific part of the medial amygdala is involved in the processing of **social and emotional cues**, particularly those related to social memory and reproductive behaviors.
- **Medial amygdala, posterodorsal part, sublayer a**: A further subdivision, indicating the intricate and layered organization of the amygdala, with different sublayers specializing in unique aspects of emotional processing.

**5. Other Specialized Brain Regions**

These regions have distinct roles and are not part of the larger functional groups above.

- **Dorsal nucleus raphe (DRN)**: A key brainstem nucleus. It is the primary source of **serotonin** projections to the forebrain. It plays a critical role in mood, sleep-wake cycles, and arousal.
- **dorsal cortical nucleus of inferior colliculus**: The inferior colliculus is a major midbrain structure in the auditory pathway, receiving input from various brainstem nuclei. The dorsal cortical nucleus is involved in **processing complex auditory stimuli**, such as sound localization and vocalizations.
- **liminal part of the r9 alar plate & mantle zone of r9Lim**: These are developmental regions from the hindbrain (rhombomere 9, r9). The alar plate is a region that gives rise to sensory neurons, and the mantle zone forms the gray matter. This suggests the regions are involved in the development of sensory nuclei in the lower brainstem.
