# Supplementary material for: Cooperation of transposable elements to endow global networks of initiators of hybrid assembly pathways of endogenous multiprotein complexes
Source: Front Cell Infect Microbiol. 2026 Jul 30;16:1793868. doi: 10.3389/fcimb.2026.1793868 (PMC13468005; doi:10.3389/fcimb.2026.1793868)
Supplement: Supplementary file 4 [file DataSheet4.zip › Sources used in the report.docx]

Carty JRE, Devarakonda K, O'Connor RM, Krek A, Espinoza D, Jimenez-Gonzalez M, Alvarsson A, Hampton RF, Li R, Qiu Y, Petri S, Shtekler A, Rajbhandari A, Conner K, Bayne M, Garibay D, Martin J, Lehmann V, Wang L, Beaumont K, Kurland I, Yuan GC, Kenny PJ, Stanley SA. Amygdala-liver signalling orchestrates glycaemic responses to stress. Nature. 2025 Sep 3. doi: 10.1038/s41586-025-09420-1. Epub ahead of print. PMID: 40903586.

**Sources used in the report**

[pmc.ncbi.nlm.nih.gov](https://pmc.ncbi.nlm.nih.gov/articles/PMC6608208/)

[Functional Properties and Projections of Neurons in the Medial Amygdala - PMC](https://pmc.ncbi.nlm.nih.gov/articles/PMC6608208/)

[Opens in a new window](https://pmc.ncbi.nlm.nih.gov/articles/PMC6608208/)

<https://www.frontiersin.org/journals/neuroanatomy/articles/10.3389/fnana.2022.837527/full" \t "_blank>

[frontiersin.org](https://www.frontiersin.org/journals/neuroanatomy/articles/10.3389/fnana.2022.837527/full)

[Integrated Behavioral, Genetic and Brain Circuit Visualization Methods to Unravel Functional Anatomy of Zebrafish Amygdala - Frontiers](https://www.frontiersin.org/journals/neuroanatomy/articles/10.3389/fnana.2022.837527/full)

[Opens in a new window](https://www.frontiersin.org/journals/neuroanatomy/articles/10.3389/fnana.2022.837527/full)

<https://en.wikipedia.org/wiki/Amygdala" \t "_blank>

[en.wikipedia.org](https://en.wikipedia.org/wiki/Amygdala)

[Amygdala - Wikipedia](https://en.wikipedia.org/wiki/Amygdala)

[Opens in a new window](https://en.wikipedia.org/wiki/Amygdala)

[scholarpedia.org](http://www.scholarpedia.org/article/Amygdala)

[Amygdala - Scholarpedia](http://www.scholarpedia.org/article/Amygdala)

[Opens in a new window](http://www.scholarpedia.org/article/Amygdala)

<https://www.frontiersin.org/journals/neuroscience/articles/10.3389/fnins.2018.00033/full" \t "_blank>

[frontiersin.org](https://www.frontiersin.org/journals/neuroscience/articles/10.3389/fnins.2018.00033/full)

[Cognitive Functions and Neurodevelopmental Disorders Involving the Prefrontal Cortex and Mediodorsal Thalamus - Frontiers](https://www.frontiersin.org/journals/neuroscience/articles/10.3389/fnins.2018.00033/full)

[Opens in a new window](https://www.frontiersin.org/journals/neuroscience/articles/10.3389/fnins.2018.00033/full)

[pmc.ncbi.nlm.nih.gov](https://pmc.ncbi.nlm.nih.gov/articles/PMC6198112/#:~:text=The%20mediodorsal%20thalamic%20nucleus%20is%20required%20for%20the%20rapid%20and,networks%20involving%20the%20prefrontal%20cortex.)

[pmc.ncbi.nlm.nih.gov](https://pmc.ncbi.nlm.nih.gov/articles/PMC6198112/#:~:text=The%20mediodorsal%20thalamic%20nucleus%20is%20required%20for%20the%20rapid%20and,networks%20involving%20the%20prefrontal%20cortex.)

[Opens in a new window](https://pmc.ncbi.nlm.nih.gov/articles/PMC6198112/#:~:text=The%20mediodorsal%20thalamic%20nucleus%20is%20required%20for%20the%20rapid%20and,networks%20involving%20the%20prefrontal%20cortex.)

[pmc.ncbi.nlm.nih.gov](https://pmc.ncbi.nlm.nih.gov/articles/PMC7394718/" \l ":~:text=The%20thalamic%20reticular%20nucleus%20(TRN,thalamocortical%20(TC)%20neurons4." \t "_blank)

[pmc.ncbi.nlm.nih.gov](https://pmc.ncbi.nlm.nih.gov/articles/PMC7394718/" \l ":~:text=The%20thalamic%20reticular%20nucleus%20(TRN,thalamocortical%20(TC)%20neurons4." \t "_blank)

[Opens in a new window](https://pmc.ncbi.nlm.nih.gov/articles/PMC7394718/" \l ":~:text=The%20thalamic%20reticular%20nucleus%20(TRN,thalamocortical%20(TC)%20neurons4." \t "_blank)

<https://www.frontiersin.org/journals/computational-neuroscience/articles/10.3389/fncom.2021.764153/full" \t "_blank>

[frontiersin.org](https://www.frontiersin.org/journals/computational-neuroscience/articles/10.3389/fncom.2021.764153/full)

[Analysis of the Neuron Dynamics in Thalamic Reticular Nucleus by a Reduced Model](https://www.frontiersin.org/journals/computational-neuroscience/articles/10.3389/fncom.2021.764153/full)

[Opens in a new window](https://www.frontiersin.org/journals/computational-neuroscience/articles/10.3389/fncom.2021.764153/full)

<https://www.frontiersin.org/journals/neural-circuits/articles/10.3389/fncir.2021.645287/full" \t "_blank>

[frontiersin.org](https://www.frontiersin.org/journals/neural-circuits/articles/10.3389/fncir.2021.645287/full)

[Connectivity and Functionality of the Globus Pallidus Externa Under Normal Conditions and Parkinson's Disease - Frontiers](https://www.frontiersin.org/journals/neural-circuits/articles/10.3389/fncir.2021.645287/full)

[Opens in a new window](https://www.frontiersin.org/journals/neural-circuits/articles/10.3389/fncir.2021.645287/full)

<https://en.wikipedia.org/wiki/Striatum" \t "_blank>

[en.wikipedia.org](https://en.wikipedia.org/wiki/Striatum)

[Striatum - Wikipedia](https://en.wikipedia.org/wiki/Striatum)

[Opens in a new window](https://en.wikipedia.org/wiki/Striatum)

<https://www.frontiersin.org/journals/neural-circuits/articles/10.3389/fncir.2021.645287/pdf" \t "_blank>

[frontiersin.org](https://www.frontiersin.org/journals/neural-circuits/articles/10.3389/fncir.2021.645287/pdf)

[Connectivity and Functionality of the Globus Pallidus Externa Under Normal Conditions and Parkinson's Disease - Frontiers](https://www.frontiersin.org/journals/neural-circuits/articles/10.3389/fncir.2021.645287/pdf)

[Opens in a new window](https://www.frontiersin.org/journals/neural-circuits/articles/10.3389/fncir.2021.645287/pdf)

<https://en.wikipedia.org/wiki/Internal_globus_pallidus" \l ":~:text=Function,-Direct%20and%20indirect&text=The%20GPi%20acts%20to%20tonically,initiation%20and%20prevents%20unwanted%20movements." \t "_blank>

[en.wikipedia.org](https://en.wikipedia.org/wiki/Internal_globus_pallidus#:~:text=Function,-Direct%20and%20indirect&text=The%20GPi%20acts%20to%20tonically,initiation%20and%20prevents%20unwanted%20movements.)

[en.wikipedia.org](https://en.wikipedia.org/wiki/Internal_globus_pallidus#:~:text=Function,-Direct%20and%20indirect&text=The%20GPi%20acts%20to%20tonically,initiation%20and%20prevents%20unwanted%20movements.)

[Opens in a new window](https://en.wikipedia.org/wiki/Internal_globus_pallidus#:~:text=Function,-Direct%20and%20indirect&text=The%20GPi%20acts%20to%20tonically,initiation%20and%20prevents%20unwanted%20movements.)

<https://pubmed.ncbi.nlm.nih.gov/17499112/" \t "_blank>

[pubmed.ncbi.nlm.nih.gov](https://pubmed.ncbi.nlm.nih.gov/17499112/)

[Globus pallidus internal segment - PubMed](https://pubmed.ncbi.nlm.nih.gov/17499112/)

[Opens in a new window](https://pubmed.ncbi.nlm.nih.gov/17499112/)

[pmc.ncbi.nlm.nih.gov](https://pmc.ncbi.nlm.nih.gov/articles/PMC3641825/)

[Motor Functions of the Superior Colliculus - PMC - PubMed Central](https://pmc.ncbi.nlm.nih.gov/articles/PMC3641825/)

[Opens in a new window](https://pmc.ncbi.nlm.nih.gov/articles/PMC3641825/)

<https://www.annualreviews.org/content/journals/10.1146/annurev-neuro-061010-113728" \t "_blank>

[annualreviews.org](https://www.annualreviews.org/content/journals/10.1146/annurev-neuro-061010-113728)

[Motor Functions of the Superior Colliculus | Annual Reviews](https://www.annualreviews.org/content/journals/10.1146/annurev-neuro-061010-113728)

[Opens in a new window](https://www.annualreviews.org/content/journals/10.1146/annurev-neuro-061010-113728)

<https://pubmed.ncbi.nlm.nih.gov/16491082/" \t "_blank>

[pubmed.ncbi.nlm.nih.gov](https://pubmed.ncbi.nlm.nih.gov/16491082/)

[The dorsomedial hypothalamic nucleus is critical for the expression of food-entrainable circadian rhythms - PubMed](https://pubmed.ncbi.nlm.nih.gov/16491082/)

[Opens in a new window](https://pubmed.ncbi.nlm.nih.gov/16491082/)

<https://en.wikipedia.org/wiki/Dorsomedial_hypothalamic_nucleus" \t "_blank>

[en.wikipedia.org](https://en.wikipedia.org/wiki/Dorsomedial_hypothalamic_nucleus)

[Dorsomedial hypothalamic nucleus - Wikipedia](https://en.wikipedia.org/wiki/Dorsomedial_hypothalamic_nucleus)

[Opens in a new window](https://en.wikipedia.org/wiki/Dorsomedial_hypothalamic_nucleus)

<https://pubmed.ncbi.nlm.nih.gov/36076337/" \t "_blank>

[pubmed.ncbi.nlm.nih.gov](https://pubmed.ncbi.nlm.nih.gov/36076337/)

[The caudal prethalamus: Inhibitory switchboard for behavioral control? - PubMed](https://pubmed.ncbi.nlm.nih.gov/36076337/)

[Opens in a new window](https://pubmed.ncbi.nlm.nih.gov/36076337/)

[pmc.ncbi.nlm.nih.gov](https://pmc.ncbi.nlm.nih.gov/articles/PMC7439563/" \l ":~:text=The%20zona%20incerta%20(ZI)%20is,2%5D%E2%80%93%5B9%5D." \t "_blank)

[pmc.ncbi.nlm.nih.gov](https://pmc.ncbi.nlm.nih.gov/articles/PMC7439563/" \l ":~:text=The%20zona%20incerta%20(ZI)%20is,2%5D%E2%80%93%5B9%5D." \t "_blank)

[Opens in a new window](https://pmc.ncbi.nlm.nih.gov/articles/PMC7439563/" \l ":~:text=The%20zona%20incerta%20(ZI)%20is,2%5D%E2%80%93%5B9%5D." \t "_blank)

[pmc.ncbi.nlm.nih.gov](https://pmc.ncbi.nlm.nih.gov/articles/PMC4580103/)

[The human medial amygdala: structure, diversity, and complexity of dendritic spines - PMC](https://pmc.ncbi.nlm.nih.gov/articles/PMC4580103/)

[Opens in a new window](https://pmc.ncbi.nlm.nih.gov/articles/PMC4580103/)

[pmc.ncbi.nlm.nih.gov](https://pmc.ncbi.nlm.nih.gov/articles/PMC10496856/)

[Dominance Status Modulates Activity in Medial Amygdala Cells with Projections to the Bed Nucleus of the Stria Terminalis - PMC - PubMed Central](https://pmc.ncbi.nlm.nih.gov/articles/PMC10496856/)

[Opens in a new window](https://pmc.ncbi.nlm.nih.gov/articles/PMC10496856/)

<https://www.frontiersin.org/journals/neuroanatomy/articles/10.3389/fnana.2012.00033/full" \t "_blank>

[frontiersin.org](https://www.frontiersin.org/journals/neuroanatomy/articles/10.3389/fnana.2012.00033/full)

[Differential efferent projections of the anterior ... - Frontiers](https://www.frontiersin.org/journals/neuroanatomy/articles/10.3389/fnana.2012.00033/full)

[Opens in a new window](https://www.frontiersin.org/journals/neuroanatomy/articles/10.3389/fnana.2012.00033/full)

<https://www.frontiersin.org/journals/neuroanatomy/articles/10.3389/fnana.2012.00033/text" \t "_blank>

[frontiersin.org](https://www.frontiersin.org/journals/neuroanatomy/articles/10.3389/fnana.2012.00033/text)

[Simple Text file - Frontiers](https://www.frontiersin.org/journals/neuroanatomy/articles/10.3389/fnana.2012.00033/text)

[Opens in a new window](https://www.frontiersin.org/journals/neuroanatomy/articles/10.3389/fnana.2012.00033/text)

<https://pubmed.ncbi.nlm.nih.gov/36265454/" \t "_blank>

[pubmed.ncbi.nlm.nih.gov](https://pubmed.ncbi.nlm.nih.gov/36265454/)

[Evolution and Development of Amygdala Subdivisions: Pallial, Subpallial, and Beyond - PubMed](https://pubmed.ncbi.nlm.nih.gov/36265454/)

[Opens in a new window](https://pubmed.ncbi.nlm.nih.gov/36265454/)

<https://www.researchgate.net/figure/Delineation-of-the-striatum-subdivisions-the-amygdala-nuclei-and-anterior-portion-of-the_fig1_265791967" \t "_blank>

[researchgate.net](https://www.researchgate.net/figure/Delineation-of-the-striatum-subdivisions-the-amygdala-nuclei-and-anterior-portion-of-the_fig1_265791967)

[Delineation of the striatum subdivisions, the amygdala nuclei and... | Download Scientific Diagram - ResearchGate](https://www.researchgate.net/figure/Delineation-of-the-striatum-subdivisions-the-amygdala-nuclei-and-anterior-portion-of-the_fig1_265791967)

[Opens in a new window](https://www.researchgate.net/figure/Delineation-of-the-striatum-subdivisions-the-amygdala-nuclei-and-anterior-portion-of-the_fig1_265791967)

[pmc.ncbi.nlm.nih.gov](https://pmc.ncbi.nlm.nih.gov/articles/PMC7749795/)

[The orbitofrontal cortex: reward, emotion and depression - PMC - PubMed Central](https://pmc.ncbi.nlm.nih.gov/articles/PMC7749795/)

[Opens in a new window](https://pmc.ncbi.nlm.nih.gov/articles/PMC7749795/)

<https://en.wikipedia.org/wiki/Orbitofrontal_cortex" \t "_blank>

[en.wikipedia.org](https://en.wikipedia.org/wiki/Orbitofrontal_cortex)

[Orbitofrontal cortex - Wikipedia](https://en.wikipedia.org/wiki/Orbitofrontal_cortex)

[Opens in a new window](https://en.wikipedia.org/wiki/Orbitofrontal_cortex)

<https://www.researchgate.net/publication/368837015_The_ventrolateral_prefrontal_cortex_is_part_of_the_modular_working_memory_system_A_functional_neuroanatomical_perspective" \t "_blank>

[researchgate.net](https://www.researchgate.net/publication/368837015_The_ventrolateral_prefrontal_cortex_is_part_of_the_modular_working_memory_system_A_functional_neuroanatomical_perspective)

[The ventrolateral prefrontal cortex is part of the modular working memory system: A functional neuroanatomical perspective - ResearchGate](https://www.researchgate.net/publication/368837015_The_ventrolateral_prefrontal_cortex_is_part_of_the_modular_working_memory_system_A_functional_neuroanatomical_perspective)

[Opens in a new window](https://www.researchgate.net/publication/368837015_The_ventrolateral_prefrontal_cortex_is_part_of_the_modular_working_memory_system_A_functional_neuroanatomical_perspective)

[pmc.ncbi.nlm.nih.gov](https://pmc.ncbi.nlm.nih.gov/articles/PMC10008902/)

[The ventrolateral prefrontal cortex is part of the modular working memory system: A functional neuroanatomical perspective](https://pmc.ncbi.nlm.nih.gov/articles/PMC10008902/)

[Opens in a new window](https://pmc.ncbi.nlm.nih.gov/articles/PMC10008902/)

<https://www.researchgate.net/profile/Oytun-Erbas/publication/363487909_Human_Prefrontal_Cortex_Regions_and_Functions/links/631fbef7071ea12e362ab9e9/Human-Prefrontal-Cortex-Regions-and-Functions.pdf" \t "_blank>

[researchgate.net](https://www.researchgate.net/profile/Oytun-Erbas/publication/363487909_Human_Prefrontal_Cortex_Regions_and_Functions/links/631fbef7071ea12e362ab9e9/Human-Prefrontal-Cortex-Regions-and-Functions.pdf)

[Human Prefrontal Cortex: Regions and Functions - ResearchGate](https://www.researchgate.net/profile/Oytun-Erbas/publication/363487909_Human_Prefrontal_Cortex_Regions_and_Functions/links/631fbef7071ea12e362ab9e9/Human-Prefrontal-Cortex-Regions-and-Functions.pdf)

[Opens in a new window](https://www.researchgate.net/profile/Oytun-Erbas/publication/363487909_Human_Prefrontal_Cortex_Regions_and_Functions/links/631fbef7071ea12e362ab9e9/Human-Prefrontal-Cortex-Regions-and-Functions.pdf)

[pmc.ncbi.nlm.nih.gov](https://pmc.ncbi.nlm.nih.gov/articles/PMC9012327/)

[Neocortical Layer 1: An Elegant Solution to Top-Down and Bottom-Up Integration - PMC](https://pmc.ncbi.nlm.nih.gov/articles/PMC9012327/)

[Opens in a new window](https://pmc.ncbi.nlm.nih.gov/articles/PMC9012327/)

[pmc.ncbi.nlm.nih.gov](https://pmc.ncbi.nlm.nih.gov/articles/PMC4412064/)

[Highly differentiated cellular and circuit properties of infralimbic pyramidal neurons projecting to the periaqueductal gray and amygdala - PubMed Central](https://pmc.ncbi.nlm.nih.gov/articles/PMC4412064/)

[Opens in a new window](https://pmc.ncbi.nlm.nih.gov/articles/PMC4412064/)

[pmc.ncbi.nlm.nih.gov](https://pmc.ncbi.nlm.nih.gov/articles/PMC5866461/)

[Layer- and subregion-specific differences in the neurophysiological properties of rat medial prefrontal cortex pyramidal neurons - PMC - PubMed Central](https://pmc.ncbi.nlm.nih.gov/articles/PMC5866461/)

[Opens in a new window](https://pmc.ncbi.nlm.nih.gov/articles/PMC5866461/)

<https://www.researchgate.net/publication/361738613_Functional_role_of_the_anterior_olfactory_nucleus_in_sensory_information_processing" \t "_blank>

[researchgate.net](https://www.researchgate.net/publication/361738613_Functional_role_of_the_anterior_olfactory_nucleus_in_sensory_information_processing)

[Functional role of the anterior olfactory nucleus in sensory information processing](https://www.researchgate.net/publication/361738613_Functional_role_of_the_anterior_olfactory_nucleus_in_sensory_information_processing)

[Opens in a new window](https://www.researchgate.net/publication/361738613_Functional_role_of_the_anterior_olfactory_nucleus_in_sensory_information_processing)

<https://en.wikipedia.org/wiki/Anterior_olfactory_nucleus" \l ":~:text=The%20AON%20distributes%20the%20information,where%20odor%20information%20processing%20occurs." \t "_blank>

[en.wikipedia.org](https://en.wikipedia.org/wiki/Anterior_olfactory_nucleus#:~:text=The%20AON%20distributes%20the%20information,where%20odor%20information%20processing%20occurs.)

[en.wikipedia.org](https://en.wikipedia.org/wiki/Anterior_olfactory_nucleus#:~:text=The%20AON%20distributes%20the%20information,where%20odor%20information%20processing%20occurs.)

[Opens in a new window](https://en.wikipedia.org/wiki/Anterior_olfactory_nucleus#:~:text=The%20AON%20distributes%20the%20information,where%20odor%20information%20processing%20occurs.)

<https://journals.physiology.org/doi/full/10.1152/jn.00715.2010" \t "_blank>

[journals.physiology.org](https://journals.physiology.org/doi/full/10.1152/jn.00715.2010)

[Membrane and synaptic properties of pyramidal neurons in the anterior olfactory nucleus | Journal of Neurophysiology | American Physiological Society](https://journals.physiology.org/doi/full/10.1152/jn.00715.2010)

[Opens in a new window](https://journals.physiology.org/doi/full/10.1152/jn.00715.2010)

<https://www.pnas.org/doi/10.1073/pnas.1003999107" \t "_blank>

[pnas.org](https://www.pnas.org/doi/10.1073/pnas.1003999107)

[Neurons in the anterior olfactory nucleus pars externa detect right or left localization of odor sources | PNAS](https://www.pnas.org/doi/10.1073/pnas.1003999107)

[Opens in a new window](https://www.pnas.org/doi/10.1073/pnas.1003999107)

<https://www.researchgate.net/figure/The-anterior-part-of-the-mouse-brain-showing-the-topography-of-the-AOB-in-serial_fig1_11868630" \t "_blank>

[researchgate.net](https://www.researchgate.net/figure/The-anterior-part-of-the-mouse-brain-showing-the-topography-of-the-AOB-in-serial_fig1_11868630)

[The anterior part of the mouse brain, showing the topography of the AOB... | Download Scientific Diagram - ResearchGate](https://www.researchgate.net/figure/The-anterior-part-of-the-mouse-brain-showing-the-topography-of-the-AOB-in-serial_fig1_11868630)

[Opens in a new window](https://www.researchgate.net/figure/The-anterior-part-of-the-mouse-brain-showing-the-topography-of-the-AOB-in-serial_fig1_11868630)

<https://www.researchgate.net/figure/Accessory-olfactory-bulb-AOB-neurons-display-voltage-dependent-bursts-of-action_fig3_291949902" \t "_blank>

[researchgate.net](https://www.researchgate.net/figure/Accessory-olfactory-bulb-AOB-neurons-display-voltage-dependent-bursts-of-action_fig3_291949902)

[Accessory olfactory bulb (AOB) neurons display voltage-dependent bursts... - ResearchGate](https://www.researchgate.net/figure/Accessory-olfactory-bulb-AOB-neurons-display-voltage-dependent-bursts-of-action_fig3_291949902)

[Opens in a new window](https://www.researchgate.net/figure/Accessory-olfactory-bulb-AOB-neurons-display-voltage-dependent-bursts-of-action_fig3_291949902)

<https://www.biorxiv.org/content/10.1101/2024.10.11.617790v1.full-text" \t "_blank>

[biorxiv.org](https://www.biorxiv.org/content/10.1101/2024.10.11.617790v1.full-text)

[Neuroanatomy of the Accessory Olfactory Bulb in the Fossorial Water Vole - bioRxiv](https://www.biorxiv.org/content/10.1101/2024.10.11.617790v1.full-text)

[Opens in a new window](https://www.biorxiv.org/content/10.1101/2024.10.11.617790v1.full-text)

<https://www.biorxiv.org/content/10.1101/2024.10.11.617790v1.full.pdf" \t "_blank>

[biorxiv.org](https://www.biorxiv.org/content/10.1101/2024.10.11.617790v1.full.pdf)

[Neuroanatomy of the Accessory Olfactory Bulb in the Fossorial Water Vole - bioRxiv](https://www.biorxiv.org/content/10.1101/2024.10.11.617790v1.full.pdf)

[Opens in a new window](https://www.biorxiv.org/content/10.1101/2024.10.11.617790v1.full.pdf)

<https://www.frontiersin.org/journals/neural-circuits/articles/10.3389/fncir.2020.561822/full" \t "_blank>

[frontiersin.org](https://www.frontiersin.org/journals/neural-circuits/articles/10.3389/fncir.2020.561822/full)

[Subpopulations of Projection Neurons in the Olfactory Bulb - Frontiers](https://www.frontiersin.org/journals/neural-circuits/articles/10.3389/fncir.2020.561822/full)

[Opens in a new window](https://www.frontiersin.org/journals/neural-circuits/articles/10.3389/fncir.2020.561822/full)

<https://en.wikipedia.org/wiki/Stratum_intermedium" \t "_blank>

[en.wikipedia.org](https://en.wikipedia.org/wiki/Stratum_intermedium)

[Stratum intermedium - Wikipedia](https://en.wikipedia.org/wiki/Stratum_intermedium)

[Opens in a new window](https://en.wikipedia.org/wiki/Stratum_intermedium)

<http://atlas.brain-map.org/atlas?atlas=181276151&structure=16251" \t "_blank>

[atlas.brain-map.org](http://atlas.brain-map.org/atlas?atlas=181276151&structure=16251)

[Developing Mouse, E15.5 - Allen Brain Atlas](http://atlas.brain-map.org/atlas?atlas=181276151&structure=16251)

[Opens in a new window](http://atlas.brain-map.org/atlas?atlas=181276151&structure=16251)

[pmc.ncbi.nlm.nih.gov](https://pmc.ncbi.nlm.nih.gov/articles/PMC6369015/)

[Infralimbic prefrontal cortex structural and functional connectivity with the limbic forebrain: a combined viral genetic and optogenetic analysis - PMC - PubMed Central](https://pmc.ncbi.nlm.nih.gov/articles/PMC6369015/)

[Opens in a new window](https://pmc.ncbi.nlm.nih.gov/articles/PMC6369015/)

<https://en.wikipedia.org/wiki/Infralimbic_cortex" \t "_blank>

[en.wikipedia.org](https://en.wikipedia.org/wiki/Infralimbic_cortex)

[Infralimbic cortex - Wikipedia](https://en.wikipedia.org/wiki/Infralimbic_cortex)

[Opens in a new window](https://en.wikipedia.org/wiki/Infralimbic_cortex)

<https://www.frontiersin.org/journals/neuroscience/articles/10.3389/fnins.2018.00033/full" \l ":~:text=The%20mediodorsal%20nucleus%20of%20the,the%20prefrontal%20cortex%20(PFC)." \t "_blank>

[frontiersin.org](https://www.frontiersin.org/journals/neuroscience/articles/10.3389/fnins.2018.00033/full" \l ":~:text=The%20mediodorsal%20nucleus%20of%20the,the%20prefrontal%20cortex%20(PFC)." \t "_blank)

[www.frontiersin.org](https://www.frontiersin.org/journals/neuroscience/articles/10.3389/fnins.2018.00033/full" \l ":~:text=The%20mediodorsal%20nucleus%20of%20the,the%20prefrontal%20cortex%20(PFC)." \t "_blank)

[Opens in a new window](https://www.frontiersin.org/journals/neuroscience/articles/10.3389/fnins.2018.00033/full" \l ":~:text=The%20mediodorsal%20nucleus%20of%20the,the%20prefrontal%20cortex%20(PFC)." \t "_blank)

<https://www.researchgate.net/figure/Mediodorsal-thalamus-establishes-numerous-connections-with-the-prefrontal-cortex-medial_fig2_344241881" \t "_blank>

[researchgate.net](https://www.researchgate.net/figure/Mediodorsal-thalamus-establishes-numerous-connections-with-the-prefrontal-cortex-medial_fig2_344241881)

[Mediodorsal thalamus establishes numerous connections with the... - ResearchGate](https://www.researchgate.net/figure/Mediodorsal-thalamus-establishes-numerous-connections-with-the-prefrontal-cortex-medial_fig2_344241881)

[Opens in a new window](https://www.researchgate.net/figure/Mediodorsal-thalamus-establishes-numerous-connections-with-the-prefrontal-cortex-medial_fig2_344241881)

<https://www.researchgate.net/figure/Connectivity-of-the-MD-in-Primates-A-On-the-left-side-connections-between-the_fig1_327764852" \t "_blank>

[researchgate.net](https://www.researchgate.net/figure/Connectivity-of-the-MD-in-Primates-A-On-the-left-side-connections-between-the_fig1_327764852)

[Connectivity of the MD in Primates. (A) On the left side - ResearchGate](https://www.researchgate.net/figure/Connectivity-of-the-MD-in-Primates-A-On-the-left-side-connections-between-the_fig1_327764852)

[Opens in a new window](https://www.researchgate.net/figure/Connectivity-of-the-MD-in-Primates-A-On-the-left-side-connections-between-the_fig1_327764852)

<https://en.wikipedia.org/wiki/Ventral_posteromedial_nucleus" \t "_blank>

[en.wikipedia.org](https://en.wikipedia.org/wiki/Ventral_posteromedial_nucleus)

[Ventral posteromedial nucleus - Wikipedia](https://en.wikipedia.org/wiki/Ventral_posteromedial_nucleus)

[Opens in a new window](https://en.wikipedia.org/wiki/Ventral_posteromedial_nucleus)

<https://en.wikipedia.org/wiki/Ventral_posterolateral_nucleus" \t "_blank>

[en.wikipedia.org](https://en.wikipedia.org/wiki/Ventral_posterolateral_nucleus)

[Ventral posterolateral nucleus - Wikipedia](https://en.wikipedia.org/wiki/Ventral_posterolateral_nucleus)

[Opens in a new window](https://en.wikipedia.org/wiki/Ventral_posterolateral_nucleus)

<https://library.fiveable.me/key-terms/anatomy-physiology/ventral-posterolateral-nucleus" \t "_blank>

[library.fiveable.me](https://library.fiveable.me/key-terms/anatomy-physiology/ventral-posterolateral-nucleus)

[Ventral Posterolateral Nucleus - (Anatomy and Physiology I) - Vocab, Definition, Explanations | Fiveable](https://library.fiveable.me/key-terms/anatomy-physiology/ventral-posterolateral-nucleus)

[Opens in a new window](https://library.fiveable.me/key-terms/anatomy-physiology/ventral-posterolateral-nucleus)

<https://taylorandfrancis.com/knowledge/Medicine_and_healthcare/Neurology/Ventral_posterolateral_nucleus/" \t "_blank>

[taylorandfrancis.com](https://taylorandfrancis.com/knowledge/Medicine_and_healthcare/Neurology/Ventral_posterolateral_nucleus/)

[Ventral posterolateral nucleus – Knowledge and References - Taylor & Francis](https://taylorandfrancis.com/knowledge/Medicine_and_healthcare/Neurology/Ventral_posterolateral_nucleus/)

[Opens in a new window](https://taylorandfrancis.com/knowledge/Medicine_and_healthcare/Neurology/Ventral_posterolateral_nucleus/)

<https://en.wikipedia.org/wiki/Paratenial_nucleus" \t "_blank>

[en.wikipedia.org](https://en.wikipedia.org/wiki/Paratenial_nucleus)

[Paratenial nucleus - Wikipedia](https://en.wikipedia.org/wiki/Paratenial_nucleus)

[Opens in a new window](https://en.wikipedia.org/wiki/Paratenial_nucleus)

<https://www.frontiersin.org/journals/behavioral-neuroscience/articles/10.3389/fnbeh.2021.634618/full" \t "_blank>

[frontiersin.org](https://www.frontiersin.org/journals/behavioral-neuroscience/articles/10.3389/fnbeh.2021.634618/full)

[The Contribution of Thalamic Nuclei in Salience Processing - Frontiers](https://www.frontiersin.org/journals/behavioral-neuroscience/articles/10.3389/fnbeh.2021.634618/full)

[Opens in a new window](https://www.frontiersin.org/journals/behavioral-neuroscience/articles/10.3389/fnbeh.2021.634618/full)

<https://www.biorxiv.org/content/10.1101/2024.09.06.611701v1" \t "_blank>

[biorxiv.org](https://www.biorxiv.org/content/10.1101/2024.09.06.611701v1)

[Brain-wide projections of mouse dopaminergic zona incerta neurons - bioRxiv](https://www.biorxiv.org/content/10.1101/2024.09.06.611701v1)

[Opens in a new window](https://www.biorxiv.org/content/10.1101/2024.09.06.611701v1)

<https://www.uniprot.org/uniprotkb/P14713/entry" \t "_blank>

[uniprot.org](https://www.uniprot.org/uniprotkb/P14713/entry)

[PHYB - Phytochrome B - Arabidopsis thaliana (Mouse-ear cress) | UniProtKB | UniProt](https://www.uniprot.org/uniprotkb/P14713/entry)

[Opens in a new window](https://www.uniprot.org/uniprotkb/P14713/entry)

<https://academic.oup.com/plcell/article/28/11/2698/6098247" \t "_blank>

[academic.oup.com](https://academic.oup.com/plcell/article/28/11/2698/6098247)

[Do Phytochromes and Phytochrome-Interacting Factors Need to Interact? | The Plant Cell | Oxford Academic](https://academic.oup.com/plcell/article/28/11/2698/6098247)

[Opens in a new window](https://academic.oup.com/plcell/article/28/11/2698/6098247)

<https://en.wikipedia.org/wiki/Stria_medullaris" \t "_blank>

[en.wikipedia.org](https://en.wikipedia.org/wiki/Stria_medullaris)

[Stria medullaris - Wikipedia](https://en.wikipedia.org/wiki/Stria_medullaris)

[Opens in a new window](https://en.wikipedia.org/wiki/Stria_medullaris)

[pmc.ncbi.nlm.nih.gov](https://pmc.ncbi.nlm.nih.gov/articles/PMC6665203/)

[The Development of the Thalamic Motor Learning Area Is Regulated by Fgf8 Expression](https://pmc.ncbi.nlm.nih.gov/articles/PMC6665203/)

[Opens in a new window](https://pmc.ncbi.nlm.nih.gov/articles/PMC6665203/)

<https://journals.plos.org/plosbiology/article?id=10.1371/journal.pbio.0050069" \t "_blank>

[journals.plos.org](https://journals.plos.org/plosbiology/article?id=10.1371/journal.pbio.0050069)

[The Prethalamus Is Established during Gastrulation and Influences Diencephalic Regionalization | PLOS Biology - Research journals](https://journals.plos.org/plosbiology/article?id=10.1371/journal.pbio.0050069)

[Opens in a new window](https://journals.plos.org/plosbiology/article?id=10.1371/journal.pbio.0050069)

<https://www.ncbi.nlm.nih.gov/books/NBK557755/" \l ":~:text=The%20primary%20function%20of%20the,sends%20information%20to%20the%20thalamus." \t "_blank>

[ncbi.nlm.nih.gov](https://www.ncbi.nlm.nih.gov/books/NBK557755/#:~:text=The%20primary%20function%20of%20the,sends%20information%20to%20the%20thalamus.)

[www.ncbi.nlm.nih.gov](https://www.ncbi.nlm.nih.gov/books/NBK557755/#:~:text=The%20primary%20function%20of%20the,sends%20information%20to%20the%20thalamus.)

[Opens in a new window](https://www.ncbi.nlm.nih.gov/books/NBK557755/#:~:text=The%20primary%20function%20of%20the,sends%20information%20to%20the%20thalamus.)

<https://www.cambridge.org/core/product/2E33BA303746341F4525262CA315C34E" \t "_blank>

[cambridge.org](https://www.cambridge.org/core/product/2E33BA303746341F4525262CA315C34E)

[Intrinsic circuitry in the deep layers of the cat superior colliculus ...](https://www.cambridge.org/core/product/2E33BA303746341F4525262CA315C34E)

[Opens in a new window](https://www.cambridge.org/core/product/2E33BA303746341F4525262CA315C34E)

<https://www.biorxiv.org/content/10.1101/2022.12.09.519812v1.full-text" \t "_blank>

[biorxiv.org](https://www.biorxiv.org/content/10.1101/2022.12.09.519812v1.full-text)

[The role of human superior colliculus in affective experiences during visual and somatosensory stimulation | bioRxiv](https://www.biorxiv.org/content/10.1101/2022.12.09.519812v1.full-text)

[Opens in a new window](https://www.biorxiv.org/content/10.1101/2022.12.09.519812v1.full-text)

<https://www.kenhub.com/en/library/anatomy/superior-colliculus" \t "_blank>

[kenhub.com](https://www.kenhub.com/en/library/anatomy/superior-colliculus)

[Superior colliculus: Anatomy and function - Kenhub](https://www.kenhub.com/en/library/anatomy/superior-colliculus)

[Opens in a new window](https://www.kenhub.com/en/library/anatomy/superior-colliculus)

<https://www.ncbi.nlm.nih.gov/books/NBK544224/" \t "_blank>

[ncbi.nlm.nih.gov](https://www.ncbi.nlm.nih.gov/books/NBK544224/)

[Neuroanatomy, Superior Colliculus - StatPearls - NCBI Bookshelf](https://www.ncbi.nlm.nih.gov/books/NBK544224/)

[Opens in a new window](https://www.ncbi.nlm.nih.gov/books/NBK544224/)

<http://braininfo.rprc.washington.edu/centraldirectory.aspx?ID=2848" \t "_blank>

[braininfo.rprc.washington.edu](http://braininfo.rprc.washington.edu/centraldirectory.aspx?ID=2848)

[middle white layer of the superior colliculus - BrainInfo - University of Washington](http://braininfo.rprc.washington.edu/centraldirectory.aspx?ID=2848)

[Opens in a new window](http://braininfo.rprc.washington.edu/centraldirectory.aspx?ID=2848)

[pmc.ncbi.nlm.nih.gov](https://pmc.ncbi.nlm.nih.gov/articles/PMC10115501/)

[What Are the Functions of the Superior Colliculus and Its Involvement in Neurologic Disorders? - PMC - PubMed Central](https://pmc.ncbi.nlm.nih.gov/articles/PMC10115501/)

[Opens in a new window](https://pmc.ncbi.nlm.nih.gov/articles/PMC10115501/)

<https://www.neurology.org/doi/10.1212/WNL.0b013e3182886a76" \t "_blank>

[neurology.org](https://www.neurology.org/doi/10.1212/WNL.0b013e3182886a76)

[Pedunculopontine nucleus - Neurology.org](https://www.neurology.org/doi/10.1212/WNL.0b013e3182886a76)

[Opens in a new window](https://www.neurology.org/doi/10.1212/WNL.0b013e3182886a76)

<https://pubmed.ncbi.nlm.nih.gov/30981899/" \l ":~:text=Good%20evidence%20relates%20the%20PPN,and%20physiology%20of%20the%20nucleus." \t "_blank>

[pubmed.ncbi.nlm.nih.gov](https://pubmed.ncbi.nlm.nih.gov/30981899/#:~:text=Good%20evidence%20relates%20the%20PPN,and%20physiology%20of%20the%20nucleus.)

[pubmed.ncbi.nlm.nih.gov](https://pubmed.ncbi.nlm.nih.gov/30981899/#:~:text=Good%20evidence%20relates%20the%20PPN,and%20physiology%20of%20the%20nucleus.)

[Opens in a new window](https://pubmed.ncbi.nlm.nih.gov/30981899/#:~:text=Good%20evidence%20relates%20the%20PPN,and%20physiology%20of%20the%20nucleus.)

<https://www.ncbi.nlm.nih.gov/books/NBK539729/" \t "_blank>

[ncbi.nlm.nih.gov](https://www.ncbi.nlm.nih.gov/books/NBK539729/)

[Neuroanatomy, Spinal Trigeminal Nucleus - StatPearls - NCBI Bookshelf](https://www.ncbi.nlm.nih.gov/books/NBK539729/)

[Opens in a new window](https://www.ncbi.nlm.nih.gov/books/NBK539729/)

<https://en.wikipedia.org/wiki/Spinal_trigeminal_nucleus" \t "_blank>

[en.wikipedia.org](https://en.wikipedia.org/wiki/Spinal_trigeminal_nucleus)

[Spinal trigeminal nucleus - Wikipedia](https://en.wikipedia.org/wiki/Spinal_trigeminal_nucleus)

[Opens in a new window](https://en.wikipedia.org/wiki/Spinal_trigeminal_nucleus)

<https://en.wikipedia.org/wiki/Dorsomedial_hypothalamic_nucleus" \l ":~:text=The%20dorsomedial%20hypothalamic%20nucleus%20receives,body%20temperature%20and%20corticosteroid%20secretion." \t "_blank>

[en.wikipedia.org](https://en.wikipedia.org/wiki/Dorsomedial_hypothalamic_nucleus#:~:text=The%20dorsomedial%20hypothalamic%20nucleus%20receives,body%20temperature%20and%20corticosteroid%20secretion.)

[en.wikipedia.org](https://en.wikipedia.org/wiki/Dorsomedial_hypothalamic_nucleus#:~:text=The%20dorsomedial%20hypothalamic%20nucleus%20receives,body%20temperature%20and%20corticosteroid%20secretion.)

[Opens in a new window](https://en.wikipedia.org/wiki/Dorsomedial_hypothalamic_nucleus#:~:text=The%20dorsomedial%20hypothalamic%20nucleus%20receives,body%20temperature%20and%20corticosteroid%20secretion.)

<https://www.mountsinai.org/about/newsroom/2025/mount-sinai-study-discovers-potential-link-between-stress-and-type-2-diabetes" \t "_blank>

[mountsinai.org](https://www.mountsinai.org/about/newsroom/2025/mount-sinai-study-discovers-potential-link-between-stress-and-type-2-diabetes)

[Mount Sinai Study Discovers Potential Link Between Stress and Type 2 Diabetes](https://www.mountsinai.org/about/newsroom/2025/mount-sinai-study-discovers-potential-link-between-stress-and-type-2-diabetes)

[Opens in a new window](https://www.mountsinai.org/about/newsroom/2025/mount-sinai-study-discovers-potential-link-between-stress-and-type-2-diabetes)

<https://my.clevelandclinic.org/health/body/21598-brainstem" \l ":~:text=is%20the%20brainstem%3F-,Your%20brainstem%20connects%20your%20brain%20to%20your%20spinal%20cord.,your%20balance%2C%20coordination%20and%20reflexes." \t "_blank>

[my.clevelandclinic.org](https://my.clevelandclinic.org/health/body/21598-brainstem#:~:text=is%20the%20brainstem%3F-,Your%20brainstem%20connects%20your%20brain%20to%20your%20spinal%20cord.,your%20balance%2C%20coordination%20and%20reflexes.)

[my.clevelandclinic.org](https://my.clevelandclinic.org/health/body/21598-brainstem#:~:text=is%20the%20brainstem%3F-,Your%20brainstem%20connects%20your%20brain%20to%20your%20spinal%20cord.,your%20balance%2C%20coordination%20and%20reflexes.)

[Opens in a new window](https://my.clevelandclinic.org/health/body/21598-brainstem#:~:text=is%20the%20brainstem%3F-,Your%20brainstem%20connects%20your%20brain%20to%20your%20spinal%20cord.,your%20balance%2C%20coordination%20and%20reflexes.)

<https://www.ncbi.nlm.nih.gov/books/NBK544297/" \t "_blank>

[ncbi.nlm.nih.gov](https://www.ncbi.nlm.nih.gov/books/NBK544297/)

[Neuroanatomy, Brainstem - StatPearls - NCBI Bookshelf](https://www.ncbi.nlm.nih.gov/books/NBK544297/)

[Opens in a new window](https://www.ncbi.nlm.nih.gov/books/NBK544297/)

<https://www.kenhub.com/en/library/anatomy/hypothalamus" \t "_blank>

[kenhub.com](https://www.kenhub.com/en/library/anatomy/hypothalamus)

[Hypothalamus: Structure and functions | Kenhub](https://www.kenhub.com/en/library/anatomy/hypothalamus)

[Opens in a new window](https://www.kenhub.com/en/library/anatomy/hypothalamus)

<https://esmed.org/MRA/mra/article/view/740/584" \t "_blank>

[esmed.org](https://esmed.org/MRA/mra/article/view/740/584)

[The Thalamus: A Review of its Functional Anatomy](https://esmed.org/MRA/mra/article/view/740/584)

[Opens in a new window](https://esmed.org/MRA/mra/article/view/740/584)

<https://www.ncbi.nlm.nih.gov/books/NBK557755/" \l ":~:text=The%20globus%20pallidus%20lies%20at,control%20conscious%20and%20proprioceptive%20movements." \t "_blank>

[ncbi.nlm.nih.gov](https://www.ncbi.nlm.nih.gov/books/NBK557755/#:~:text=The%20globus%20pallidus%20lies%20at,control%20conscious%20and%20proprioceptive%20movements.)

[www.ncbi.nlm.nih.gov](https://www.ncbi.nlm.nih.gov/books/NBK557755/#:~:text=The%20globus%20pallidus%20lies%20at,control%20conscious%20and%20proprioceptive%20movements.)

[Opens in a new window](https://www.ncbi.nlm.nih.gov/books/NBK557755/#:~:text=The%20globus%20pallidus%20lies%20at,control%20conscious%20and%20proprioceptive%20movements.)

<https://pubmed.ncbi.nlm.nih.gov/19463910/" \t "_blank>

[pubmed.ncbi.nlm.nih.gov](https://pubmed.ncbi.nlm.nih.gov/19463910/)

[What are the functions of fish brain pallium? - PubMed](https://pubmed.ncbi.nlm.nih.gov/19463910/)

[Opens in a new window](https://pubmed.ncbi.nlm.nih.gov/19463910/)

<https://en.wikipedia.org/wiki/Pallium_(neuroanatomy)" \t "_blank>

[en.wikipedia.org](https://en.wikipedia.org/wiki/Pallium_(neuroanatomy))

[Pallium (neuroanatomy) - Wikipedia](https://en.wikipedia.org/wiki/Pallium_(neuroanatomy))

[Opens in a new window](https://en.wikipedia.org/wiki/Pallium_(neuroanatomy))

Sources read but not used in the report
