# Supplementary material for: Cooperation of transposable elements to endow global networks of initiators of hybrid assembly pathways of endogenous multiprotein complexes
Source: Front Cell Infect Microbiol. 2026 Jul 30;16:1793868. doi: 10.3389/fcimb.2026.1793868 (PMC13468005; doi:10.3389/fcimb.2026.1793868)
Supplement: Supplementary file 4 [file DataSheet4.zip › Summary Functional Neuroanatomy of 100 Brain Regions.docx]

**Functional Neuroanatomy: An Expert-Level Analysis**

**Executive Summary**

This analysis synthesizes neuroanatomical and physiological data of various brain regions, revealing a sophisticated, interconnected system for innate behavior, cognitive control, motor execution, and homeostatic regulation. A key finding is the profound functional specialization at the sub-regional and microcircuit levels. The report highlights the amygdala's role as a nexus for innate emotional responses, the thalamus as a dynamic cortical partner for executive function, and the superior colliculus as a hub for rapid sensory-motor transformation. A critical link between psychological stress centers and physiological systems is also illuminated.

**Key Thematic Insights**

The analysis is structured around four major functional themes:

- **The Limbic System as a Hub for Innate Behavior**: The amygdaloid and orbitofrontal systems integrate sensory information, particularly chemosignals, to generate innate and learned emotional responses related to social, reproductive, and defensive behaviors.
- **The Thalamus as a Multifaceted Gatekeeper**: Thalamic nuclei, such as the Mediodorsal (MD) and Reticular (TRN), actively regulate cortical activity, orchestrating attention, executive control, and information exchange for cognitive flexibility.
- **Interconnected Motor-Cognitive Loops**: The basal ganglia, including the globus pallidus and nucleus accumbens, are crucial for complex motor control and are intertwined with cognitive processes like decision-making, motivation, and reward perception. The superior colliculus enables rapid sensory-to-motor transformation.
- **Neural Circuits for Survival and Homeostasis**: Regions like the dorsomedial hypothalamus and zona incerta act as key integrative nodes, linking internal metabolic states and stress responses with broader behavioral control and survival functions.

**Functional Systems and Associated Regions**

**I. The Limbic and Cortical Network**

This network is key for emotion, decision-making, and olfactory processing. It includes the **medial amygdalar nucleus** (involved in innate behaviors like reproduction and defense) and the **orbitofrontal cortex** (OFC), which processes reward value and decision-making. The lateral OFC processes non-reward and punishment, while the medial OFC handles pleasant stimuli, creating a "push-pull" system for behavioral regulation. The **anterior olfactory nucleus** and **accessory olfactory bulb** are central to olfactory processing, bypassing the thalamus to distribute scent information directly. The **infralimbic area** is critical for fear extinction, acting as an "off-switch" for learned fear responses.

**II. The Thalamic System**

This system handles sensory relay and cognitive integration. The **mediodorsal nucleus (MD)** is a higher-order nucleus that partners with the prefrontal cortex for executive functions. The **ventral posterolateral nucleus (VPL)** is a major relay for somatosensory information. The **reticular nucleus (TRN)** acts as a "guardian of the gateway," regulating information flow between the thalamus and cortex, which is key for selective attention. Other nuclei like the **Parataenial nucleus** integrate limbic and motivational information.

**III. The Motor Systems and Brainstem Hubs**

This group is involved in movement, reflexes, and sensorimotor transformation. The **globus pallidus** is a central component of the basal ganglia, controlling both motor and non-motor functions. The **external segment (GPe)** is a complex hub, while the **internal segment (GPi)** is the primary output, providing tonic inhibition to prevent unwanted movements. The **superior colliculus (SC)** is a remarkable structure that transforms sensory signals into rapid orienting behaviors, with its deep layers integrating diverse sensory and internal signals. The **pedunculopontine nucleus (PPN)** and **cuneiform nucleus** form the mesencephalic locomotor region, which controls movement and arousal.

**IV. Homeostatic and Developmental Systems**

This section covers the regulation of internal states and foundational neuroanatomy. The **dorsomedial nucleus of the hypothalamus (DMH)** is a key component for regulating feeding, body weight, and circadian rhythms. It also functions as a "stress center" driving cardiovascular responses. This system includes a circuit that directly links the amygdala to the liver, showing a direct pathway from psychological stress to physiological responses. Other foundational structures like the **brain stem** and **hindbrain** are responsible for vital, unconscious life functions such as breathing and heart rate.
