# Supplementary material for: Cooperation of transposable elements to endow global networks of initiators of hybrid assembly pathways of endogenous multiprotein complexes
Source: Front Cell Infect Microbiol. 2026 Jul 30;16:1793868. doi: 10.3389/fcimb.2026.1793868 (PMC13468005; doi:10.3389/fcimb.2026.1793868)
Supplement: Supplementary file 5 [file DataSheet5.zip › AI model use description.docx]

During the final rounds of the revision of this contribution, the need has emerged for downstream expert-level analyses of identified in this contribution 25 transcription factors that are significantly differentially enriched in binding to human brain development regulatory regions (BDRRs) vis-à-vis chimpanzee BDRRs. Similarly, the expert-level follow-up analyses appear necessary of the neuroanatomical and structural-functional features, as well as developmental timelines of identified in this contribution 202 human brain regions in which the increased expression of these 25 transcription factors was significantly enriched. To accomplish these tasks, the Artificial Intelligence (AI) Large Language Model (LLM) was employed.

These analyses and reports were conceived, prompted, and developed by the author using Google's Gemini (version 1.5 Pro) in September, 2025 (Google. (2025). *Gemini* (Version 1.5 Pro) [Large language model]. <https://gemini.google.com/> . Outputs was reviewed, analyzed, edited, and refined by the author, who are solely responsible for the content.

**A catalogue of the peports**

**1. Executive Summary: Neuroanatomical and Structural-Functional Report of 202 Human Brain Regions**

**2. Expert-Level Analysis of Human Neuroanatomy and Structural-Functional Features of 202 Human Brain Regions**

**3. Analysis of the top 25 transcription factors that are differentially enriched in human versus chimpanzee BDRRs**

**4. Inference of structural-functional implications of distinct developmental trajectories of 202 brain regions for human and chimpanzee brain development and functions**

**5. The Chronological Development of Brain Regions**

**6. Artificial Intelligence assessment of potential significance of reported observations suggesting interconnected patterns of developmental regulation of identified brain regions by 25 transcription factors**

**7. Sources used in the analyses and reports**
