# Supplementary material for: Cooperation of transposable elements to endow global networks of initiators of hybrid assembly pathways of endogenous multiprotein complexes
Source: Front Cell Infect Microbiol. 2026 Jul 30;16:1793868. doi: 10.3389/fcimb.2026.1793868 (PMC13468005; doi:10.3389/fcimb.2026.1793868)
Supplement: Supplementary file 5 [file DataSheet5.zip › 1. AI Executive Summary OnePage.docx]

**1. Executive Summary: Neuroanatomical and Structural-Functional Report**

This report provides an Executive Summary of comprehensive analyses of the neuroanatomy and functional roles of over 200 human brain regions, with a particular focus on subcortical and brainstem structures. The analysis reveals the brain's intricate, hierarchical organization, where specialized nuclei and layers work in concert to govern everything from basic motor function to complex cognitive and emotional processes.

**Key Findings by System**

**1. The Thalamus: The Brain's Central Relay Hub** The analysis confirmed the thalamus as a critical relay station for sensory, motor, and limbic information. Different nuclei within the thalamus are highly specialized:

- **Mediodorsal Nucleus (MD):** A key player in executive function, working memory, and decision-making, with direct links to the prefrontal cortex.
- **Anterior Group:** A central component of the Papez circuit, fundamental for memory formation and spatial navigation.
- **Intralaminar Nuclei:** Crucial for arousal, attention, and pain perception, integrating signals from the brainstem.
- **Geniculate Complexes:** Serve as the primary sensory relays for vision (Lateral Geniculate Complex) and hearing (Medial Geniculate Complex), channeling information to the respective cortices.

**2. Brain Stem and Midbrain: Command Centers for Fundamental Processes** These regions are not merely signal conduits but active processing centers.

- **Superior Colliculus:** A multi-layered structure vital for orienting the head and eyes towards visual and auditory stimuli.
- **Inferior Colliculus:** The central hub for auditory processing in the midbrain, essential for sound localization and frequency analysis.
- **Cochlear Nuclei:** The initial brainstem processing centers for auditory input from the ear, critical for decoding sound characteristics.

**3. Basal Ganglia: The Engine of Motor Control** The report highlights the Globus Pallidus as a core component of the motor system.

- **Globus Pallidus (Internal & External):** This structure acts as the main output regulator of the basal ganglia, tonically inhibiting the thalamus. Its modulated activity is necessary for the initiation and execution of voluntary movements.

**4. Limbic System and Amygdala: The Seat of Emotion and Motivation** The analysis underscores the limbic system's role in governing emotional and motivational behaviors.

- **Medial Amygdalar Nucleus:** This region is strongly implicated in innate social behaviors such as aggression and mating, receiving direct input related to pheromonal cues.
- **Bed Nucleus of the Stria Terminalis (BNST):** Part of the extended amygdala, this structure is crucial for the sustained, long-term response to stress and anxiety.

**Conclusion** This comprehensive neuroanatomical review demonstrates that the brain's subcortical structures are organized into highly specific, interconnected functional groups. The findings reinforce that the brain operates as a complex, dynamic network where each seemingly small region plays a critical, specialized role in the vast tapestry of human function.
