# Supplementary material for: Cooperation of transposable elements to endow global networks of initiators of hybrid assembly pathways of endogenous multiprotein complexes
Source: Front Cell Infect Microbiol. 2026 Jul 30;16:1793868. doi: 10.3389/fcimb.2026.1793868 (PMC13468005; doi:10.3389/fcimb.2026.1793868)
Supplement: Supplementary file 5 [file DataSheet5.zip › 2. AI Expert_Level Report 202 human brain regions.docx]

**Expert-Level Analysis of Human Neuroanatomy and Structural-Functional Features of 202 Human Brain Regions**

This report was conceived, prompted, and developed using Google's Gemini (version 1.5 Pro) in September, 2025 (Google. (2025). *Gemini* (Version 1.5 Pro) [Large language model]. <https://gemini.google.com/> . Its output was reviewed, analyzed, edited, and refined by the author, who are solely responsible for the content.

**I. Introduction: Foundational Principles of Brain Organization**

Modern molecular profiling-based prosomeric model of brain development postulates a set of 20 transverse prosomeres (plus a number of additional spinal cord units) as the developmental units from which all the cerebral parts are constructed and the particular typology of many different neuronal populations within the forebrain and the hindbrain emerged (Puelles L, 2024). The prosomeric model posits that the forebrain and hindbrain are constructed from a series of transverse segments, known as prosomeres and rhombomeres, each possessing a distinct molecular and genetic profile. Thus, according to this model, the brain is formed by an uninterrupted series of transverse subunits of the neural tube. For instance, the diencephalon, or "interbrain", is derived from prosomeres p1, p2, and p3. This shared lineage is crucial for comprehending the anatomical and functional associations between seemingly disparate adult structures, such as the thalamic reticular nucleus (TRN) and the zona incerta (ZI), both of which emerge from the alar plate of prosomere 3 (p3), or the prethalamus. By tracing their origins to a common embryonic source, their interconnected roles in modulating information flow become more apparent.

Wilhelm His longitudinal plate model subdivides the developing neural tube into four parallel, dorsoventral plates: the roof, alar, basal, and floor plates. This model provides a clear organizational framework for understanding how the central nervous system develops, with each plate giving rise to distinct functional components. This model provides a clear sensorimotor axis. The alar plate predominantly generates sensory neuronal columns, which receive and process sensory input, while the basal plate produces motor-related elements, such as motoneurons and associated interneurons. This functional segregation is preserved in the adult brain, as exemplified by the superior colliculus (SC). Its superficial layers, derived from the alar plate, are dedicated to sensory processing, whereas its deeper layers integrate this sensory information to generate motor outputs. This report uses these powerful models to illuminate the structural logic and functional relationships of the brain regions in question, presenting a coherent narrative of their neuroanatomical and physiological significance.

**II. The Diencephalon: The Thalamic Complex, a Central Hub of Integration**

The diencephalon, a major division of the forebrain, contains the thalamus and prethalamus, which are far more than simple relay stations. These structures act as dynamic, interactive hubs that integrate, filter, and distribute information throughout the central nervous system.

**2.1 The Mediodorsal Nucleus of the Thalamus (MD): A High-Order Executive and Limbic Integrator**

The mediodorsal nucleus (MD), also known as the dorsomedial nucleus, is the largest nucleus within the medial part of the thalamus. It is considered the main relay center for information destined for the prefrontal cortex (PFC), a relationship so prominent that it has been termed a PFC-MD loop. This loop is a fundamental component of the brain's executive networks. The query's mention of its lateral, central, and medial parts alludes to the known anatomical and functional heterogeneity of this structure. The MD's robust, bidirectional connections with the PFC are a cornerstone of its function, enabling a fluid, ongoing communication during rapid cognitive operations.

Beyond executive functions, the MD's extensive reciprocal connections with hypothalamic and other thalamic nuclei allow it to integrate a wide variety of sensory information with emotional and subjective states. Consequently, its function extends beyond cognition to encompass a range of physiological processes. These include thermogenesis, the stress response, regulation of plasma glucose levels, food intake, and the circadian rhythm. The integrative role of the MD suggests it is not merely passing signals but is actively shaping the informational context for higher cortical processing. The provided sources, however, do not offer specific functional or connectional details for the MD's lateral, central, and medial sub-parts, presenting a clear data gap in the provided material.

**2.2 The Anterior Thalamic Group: A Key Node in the Hippocampal-Diencephalic-Cingulate Circuit**

The anterior nuclei of the thalamus (ANT), comprised of subnuclei such as the anteromedial (AM) and anteroventral (AV) nuclei, form a vital node in the neural circuits that support spatial learning and episodic memory. This group's role in memory and cognition is deeply intertwined with its extensive connections to the hippocampus, mammillary bodies, and cingulate cortex.

The anteroventral nucleus (AV) is particularly significant for promoting synaptic plasticity within the hippocampal circuit. It possesses extensive reciprocal interactions with the subiculum and retrosplenial cortex and, alongside the medial mammillary nucleus, conveys the **theta rhythm activity** which is critical for this process. The rhythmic and synchronous firing of AV neurons with the hippocampal theta frequency (4–7 Hz) is initiated by hippocampal input and supports the mechanisms of synaptic plasticity in the hippocampal formation and retrosplenial cortex. This connection illustrates how a thalamic nucleus can directly contribute to the fundamental neural processes of memory formation. While the interanterodorsal nucleus is also listed in the query, the provided material does not contain specific data regarding its function or connections.

**2.3 The Reticular Nucleus of the Thalamus (TRN): The Thalamocortical Gatekeeper**

The thalamic reticular nucleus (TRN) is a unique and pivotal structure that reframes the understanding of thalamic function from a simple relay to an active modulator. Embryologically, the TRN originates from the prethalamic alar plate (p3), a common source it shares with the zona incerta. Functionally, it is distinct from most thalamic nuclei, as it is composed entirely of GABAergic neurons. It receives excitatory inputs from both corticothalamic and thalamocortical neurons and, in turn, sends inhibitory outputs back to the thalamus. This neurochemical profile and anatomical position—surrounding most of the rostrolateral surface of the thalamus—allows it to act as an anatomical and functional hub.

The TRN's strategic position enables it to modulate the flow of information between the cerebral cortex and thalamus. This is not a passive process; the TRN serves as a dynamic "gatekeeper". By receiving excitatory feedback from both cortical and thalamic areas and providing inhibitory outputs to the thalamus, the TRN can selectively filter, suppress, or amplify specific signals. This mechanism is crucial for regulating sensory attention, arousal, sleep, and cognition. The clinical significance of this gating function is underscored by the link between TRN dysfunction and disorders such as autism, attention deficit hyperactivity disorder (ADHD), and schizophrenia, where an inability to appropriately modulate information flow may contribute to behavioral and sensory deficits. This underscores that the integrity of this feedback loop is fundamental to normal neurological function.

**Table 1: The Thalamic Complex: A Functional Map**

| Region | Location/Anatomical Subdivisions | Key Afferent Connections | Key Efferent Connections | Primary Functions |
| --- | --- | --- | --- | --- |
| **Mediodorsal Nucleus (MD)** | Medial thalamus; Lateral, Central, Medial parts | Prefrontal cortex, hypothalamus, limbic system | Prefrontal cortex | Executive functions (planning, working memory), integration of sensory and emotional states, thermogenesis, stress response, circadian rhythms |
| **Anterior Group (ANT)** | Superior thalamus; Anteromedial (AM), Anteroventral (AV), Anterodorsal (AD) | Subiculum, retrosplenial cortex, mammillary bodies | Anterior cingulate cortex, orbitomedial prefrontal cortex | Episodic memory, spatial learning, emotional and executive functions |
| **Reticular Nucleus (TRN)** | Surrounds the rostrolateral thalamus | Corticothalamic and thalamocortical neurons | Thalamus (inhibitory) | Modulation of information flow, sensory attention, sleep, arousal, cognition |
| **Zona Incerta (ZI)** | Subthalamus; between thalamus and hypothalamus | Cerebral cortex (frontal, cingulate, parietal), cerebellum, brainstem | Cerebral cortex (layer I), thalamic intralaminar nuclei, hypothalamus, basal ganglia, spinal cord | Coordination of somatotopically organized information, motor control, wide-ranging neuromodulatory effects |
| **Nucleus of Reuniens (RE)** | Ventral midline thalamus | Hippocampus, medial prefrontal cortex | Medial prefrontal cortex, hippocampus | Mnemonic, executive, and affective functions; hub for HF-mPFC dialogue |

**2.4 The Prethalamus (Ventral Thalamus): The Proximal Motor Diencephalon**

The prethalamus, also known as the ventral thalamus, corresponds to prosomere 3 (p3). This region is patterned by Sonic hedgehog signaling and contains key nuclei such as the zona incerta (ZI) and the TRN.

The **zona incerta (ZI)** is a small, horizontally elongated nucleus located between the thalamus and hypothalamus. Its name, derived from a Latin term meaning "region of which nothing certain can be said," reflects its historical lack of clear definition. Modern analysis, however, has revealed its remarkable cellular and neurochemical diversity, containing up to 20 different types of neurochemically defined cells, including GABAergic and dopaminergic neurons. The ZI's connections are exceptionally widespread, projecting from the cerebral cortex down to the spinal cord. A notable feature of its connectivity is that it avoids the primary sensory relay nuclei of the thalamus. The ZI's projections are often "in register," meaning the representations of a specific body part from different brain regions converge on the same area of the ZI, suggesting a role in coordinating somatotopically organized information.

The **prethalamic eminence (PThE)** is the most dorsal subdomain of the prethalamus. It is known as a source of migratory neurons that populate telencephalic and hypothalamic regions. The query references the mantle zone of PThE and other mantle zone structures such as mantle zone of p2ZL, mantle zone of RtC, and mantle zone of p3ZL. According to the textbooks’ descriptions, during the embryonic development of neural tube, as the cells adjacent to the lumen continue to divide, the migrating cells form a second layer around the original neural tube. This layer becomes progressively thicker as more cells are added to it from the germinal neuroepithelium. This new layer is called the mantle (or intermediate) zone, and the germinal epithelium is now called the ventricular zone (and, later, the ependyma). The mantle zone cells differentiate into both neurons and glia. The neurons make connections among themselves and send forth axons away from the lumen, thereby creating a cell-poor marginal zone. Eventually, glial cells cover many of the axons in the marginal zone in myelin sheaths, giving them a whitish appearance. Hence, the mantle zone, containing the neuronal cell bodies, is often referred to as the gray matter; the axonal, marginal layer is often called the white matter. Thus, the accumulation of neuroblasts and glioblasts forms a zone of high cell density in the wall of the nerual tube, which is designated the mantle zone or the mantle layer. Cells remaining in the neural cavity lining are designated ependymal cells and they form an ependymal layer. A cell sparse zone where axons of neurons and some glial cells are present surrounds the mantle layer and it is designated the marginal layer. The mantle layer becomes gray matter and the marginal layer becomes white matter of the CNS. Mantle zone of p2ZL and mantle zone of p3ZL. The mantle zone of p2ZL is a crucial part of the brain's development, particularly during the formation of the cerebral cortex. It is where precursor cells that will become neurons and glial cells (neuroblasts) are generated. The mantle zone is characterized by a high cell density and is essential for the proper development of the CNS, specifically in the formation of the gray matter. The process of neurogenesis, where these precursor cells differentiate into mature neurons, occurs in the ventricular zone and then migrates to their final locations in the cortex. The mantle zone plays a significant role in this migration, as it provides a layer of support for the neurons as they travel to their designated layers in the cortex. See original source files and references for further details [Tissue Architecture of the Central Nervous System - Developmental Biology - NCBI Bookshelf](https://www.ncbi.nlm.nih.gov/books/NBK10047/)

Mantle zone of RtC. The mantle zone of the right cerebral hemisphere (RtC) brain development is a crucial layer that forms during the early stages of brain development. It is located between the pia mater and the ventricular zone and is characterized by a high cell density. The mantle zone is involved in the formation of the cerebral cortex, which is the outermost layer of the brain. The neuroblasts that migrate from the ventricular zone to the mantle zone are the first-born or pioneer neurons, primarily Cajal-Retzius cells. These cells play a key role in signaling for cell migration and organization during corticogenesis. The mantle zone also contains the precursor to the subplate, which is essential for the formation of the cortical plate. The process of corticogenesis, or the development of the cerebral cortex, occurs from embryonic day 10 to 17 in mice and between gestational weeks seven to 18 in humans. The cortex consists of up to six layers, with the mantle zone contributing to the formation of the cortical plate, which is the outermost layer of the cortex. <https://embryology.ch/en/organogenesis/nervous-system/fundamentals-of-cerebral-development/histogenesis-cerebral-cortex/>

**2.5 Midline and Intralaminar Thalamic Nuclei: Modulators of Arousal and Consciousness**

The midline and intralaminar nuclei of the thalamus, including the **nucleus of reuniens (RE)**, are central to cognitive and emotional regulation. The RE, a ventral midline nucleus, is distinguished by its strong reciprocal connections with the hippocampus (HF) and the medial prefrontal cortex (mPFC). A crucial functional implication of this connectivity is that the RE serves as the primary route of communication from the mPFC back to the hippocampus, bridging a major anatomical gap, as there are no direct mPFC-HF projections. This anatomical arrangement positions the RE as a critical "circuit hub" that facilitates a "dialogue" between these two key regions, especially during sleep-dependent memory consolidation. Disruption of the RE has been shown to impair synchronous oscillations between the hippocampus and mPFC, leading to deficits in working memory, executive functions, and affective behaviors, and has been linked to disorders like schizophrenia and epilepsy.

The **perireunensis nucleus** is a minor cell group in the thalamus located adjacent to the reuniens nucleus. It is a component of the medial group of the dorsal thalamus in rodents. The provided sources do not offer specific information on its function or connections in humans.

**III. The Basal Ganglia and Its Connections: Orchestrators of Movement and Behavior**

The basal ganglia, and particularly the pallidum, are more than simple initiators of movement. They are complex orchestrators that finely modulate voluntary action and a wide range of higher cortical functions.

**Table 2: The Basal Ganglia's Motor Control Pathways**

| Pathway | Key Structures | Primary Neurotransmitter(s) | Net Effect on Thalamus/Movement |
| --- | --- | --- | --- |
| **Direct Pathway** | Striatum → GPi/SNr → Thalamus | GABAergic inhibitory | Disinhibits the thalamus; initiates movement |
| **Indirect Pathway** | Striatum → GPe → STN → GPi/SNr → Thalamus | GABAergic inhibitory, glutamatergic excitatory | Increases inhibition of the thalamus; suppresses movement |
| **Hyperdirect Pathway** | Cortex → STN → GPi/SNr → Thalamus | Glutamatergic excitatory | Suppresses thalamic activity; increases inhibitory influences on upper motor neurons |

**3.1 The Dorsal Pallidum**

The dorsal pallidum is a broad anatomical term that includes the main parts of the globus pallidus and substantia nigra. This system is responsible for the fine-tuning of voluntary movements. It achieves this by receiving incipient movement commands from the cerebral cortex, processing and adjusting them, and then relaying the modified instructions back to the cortex via the thalamus. This circuit is also involved in other higher cognitive functions, including planning, decision-making, reward processing, and working memory.

**3.2 The Globus Pallidus: An Intrinsic Circuit Hub (GPe) and Output Center (GPi)**

The globus pallidus is composed of two primary segments with distinct functional roles: the external segment (GPe) and the internal segment (GPi).

The **external segment (GPe)** is an intrinsic nucleus that serves as a central hub within the multiple feedback loops of the basal ganglia circuits. Its primary function is to act as a relay for information. It is a key component of the indirect pathway, which has a net inhibitory effect on movement initiation. The GPe receives powerful excitatory input from the subthalamic nucleus (STN) and inhibitory input from the striatum. The continuous, high-frequency firing of GPe neurons (mean rate of 55 Hz in awake monkeys) is maintained by a tonic level of excitatory input from the STN. When this tonic glutamatergic input is removed, GPe activity ceases to be continuous and instead develops into alternating, long active and silent phases. This observation demonstrates that the tonic excitatory drive from the STN is fundamental to maintaining the normal, continuous firing pattern of pallidal neurons. This mechanism provides a physiological basis for the motor pathology seen in disorders like Parkinson's disease, where disruptions to this delicate balance of inputs can lead to pathological oscillations and motor deficits.

In contrast, the **internal segment (GPi)** is the primary output nucleus of the basal ganglia. It is the final common pathway for the direct and indirect circuits, sending its outputs to the thalamus to be relayed back to the cerebral cortex to modulate movement. Both GPe and GPi receive similar inputs and are subject to the same opposing effects from cortical stimulation: an early disynaptic excitation via the STN and a later disynaptic inhibition via the striatum. The GPi, unlike the GPe, does not have extensive local axon collaterals, which is consistent with its role as a final output station rather than a complex intrinsic processing hub.

**IV. The Midbrain and Brainstem: Sensory-Motor Transformation Centers**

These lower brain regions are responsible for fundamental and rapid processing, from sensory relay to reflexive motor control.

**4.1 The Superior Colliculus (SC): A Multi-Sensory Orienting and Decision-Making Hub**

The superior colliculus (SC), a paired structure in the rostral midbrain, is a highly laminated structure with seven distinct layers. It is a quintessential example of His's developmental principle, where a functional distinction exists between sensory and motor-related layers.

The superficial layers (stratum zonale, stratum griseum superficiale, and stratum opticum) are purely sensory, receiving input primarily from the retina and visual cortex. In contrast, the intermediate (stratum griseum intermedium and stratum album intermedium) and deep layers (stratum griseum profundum and stratum album profundum) are the core of the SC's sensorimotor function. They are not exclusively visual but receive multimodal sensory information from visual, auditory, and somatosensory pathways. The SC transforms these diverse sensory inputs into motor commands for orienting the eyes, head, and arms toward or away from a stimulus. This transformation occurs through a spatially aligned motor map in the deepest layer.

The intermediate gray layer and deep gray layer are central to this process. A particular finding reveals that neurons in a specific sublayer of the intermediate gray layer, SGIb (sublayer b), are less likely to respond to direct visual layer stimulation than other neurons in the intermediate layer, suggesting that they receive visual information indirectly. This indicates a complex intrinsic circuit for visuomotor integration, where information is not simply relayed but is processed in a multi-step manner. The intermediate white layer and deep white layer are composed primarily of fibers that convey these motor commands and projections.

The SC's ability to integrate different sensory modalities into a single, common spatial map for directed behavior is a fundamental evolutionary solution for rapid, adaptive responses. It allows for rapid orienting and evasive movements, a function critical for survival, by bypassing the more nuanced and slower cognitive appraisal of the forebrain.

**4.2 The Pretectal Region: The Visceral Visual System**

The pretectal area, or pretectum, is a bilateral group of nuclei situated at the junction of the midbrain and forebrain. It is a component of the subcortical visual system, responsible for mediating non-conscious, reflexive behavioral responses to acute changes in ambient light.

The primary function of the pretectum, particularly its olivary pretectal nucleus, is to mediate the **pupillary light reflex**. This reflex, which causes the constriction of pupils in response to light, is initiated by retinal input to the pretectum. The pretectum then relays this information to the Edinger-Westphal nucleus, which controls the pupillary sphincter muscles. Additionally, the anterior pretectal nucleus (NPA) mediates somatosensory and nociceptive information, a non-visual role that highlights the region's functional diversity.

**4.3 Auditory and Trigeminal Pathways in the Brainstem**

The brainstem is also home to critical sensory relays for hearing and facial sensation.

The **inferior colliculus (IC)** is the primary midbrain relay for auditory information traveling from the inner ear to the auditory cortex. Its largest and most central subdivision is the **central nucleus**, which is the main site for auditory inputs. This nucleus is tonotopically organized, meaning it preserves the spatial mapping of pitch from the cochlea. The IC is crucial for sound localization, pitch discrimination, and the mediation of the startle response to loud sounds.

The **spinal nucleus of the trigeminal** is a sensory tract in the lateral medulla. Its principal function is to relay pain, temperature, and touch sensations from the ipsilateral face. The nucleus is subdivided into three parts with a distinct inverted topographical map of the face. The **pars interpolaris** receives inputs from the ipsilateral face and, in conjunction with the pars oralis, forms the ascending anterior trigeminothalamic tract. This tract relays the sensory information to the thalamus, making the nucleus a pivotal component of the sensory pathway.

**Table 3: Sensorimotor Integration in the Midbrain**

| Structure | Subdivision | Inputs/Afferents | Outputs/Efferents | Primary Functions |
| --- | --- | --- | --- | --- |
| **Superior Colliculus** | **Superficial Layers** | Retina, visual cortex | N/A | Purely sensory, visual processing |
|  | **Intermediate/Deep Layers** | Visual, auditory, somatosensory pathways | Brainstem, spinal cord, thalamus, oculomotor nuclei | Sensorimotor transformation, orienting movements, saccade generation, detecting looming motion |
| **Inferior Colliculus** | **Central Nucleus** | Auditory cortex, cochlea | Medial geniculate nucleus (thalamus) | Auditory information relay, sound localization, pitch discrimination |
| **Spinal Trigeminal Nucleus** | **Pars Interporalis** | Ipsilateral face, Trigeminal nerve (CN V) branches | Ascending anterior trigeminothalamic tract (with pars oralis) | Relay of pain, temperature, and touch sensations from the face |

**4.4 The Brainstem Reticular Complex: Widespread Modulation**

The reticular formation is a complex, diffuse network of nuclei spanning the brainstem. Despite its lack of distinct cytoarchitectural boundaries, it is a major integration and relay center for many vital brain systems. It plays a modulatory and premotor role in functions such as arousal, consciousness, cardiovascular control, pain modulation, and the coordination of somatic motor movements.

The **pedunculopontine nucleus (PPN)** is a key component of this complex, situated in the mesencephalic locomotor region (MLR). The PPN is a crucial link between the basal ganglia and brainstem motor systems, and it is involved in the control of locomotion, posture, and gait. It receives excitatory input from the motor cortex and inhibitory input from the globus pallidus interna (GPi), underscoring its role in integrating basal ganglia output to control and initiate movement.

**V. The Limbic and Forebrain Systems: The Hubs of Emotion and Motivation**

This section examines the higher-order structures that are critical for processing innate, social, and emotional behaviors.

**5.1 The Medial Amygdalar Nucleus (MeA): A Chemosensory Hub for Innate Behaviors**

The medial amygdalar nucleus (MeA) is a central structure for innate emotional behaviors, particularly those triggered by chemosignals, such as pheromones and predator cues. The MeA receives direct olfactory information and processes it before relaying signals to hypothalamic nuclei that control defensive and reproductive behaviors.

The MeA demonstrates a clear functional compartmentalization between its subdivisions, particularly the posterodorsal (MePD) and posteroventral (MePV) parts. The MePD is primarily involved in social and reproductive behaviors, projecting to structures like the medial preoptic nucleus and ventrolateral aspect of the ventromedial hypothalamic nucleus. In contrast, the MePV is strongly implicated in defensive responses, particularly predator fear. It projects to the medial posterointermediate bed nucleus of the stria terminalis and the anterior hypothalamic area. This segregation highlights a fine-tuned, specialized neural architecture for instinctual behavioral responses. However, the provided material does not offer specific functional or connectional information for the sublayers (a, b, and c) of the posterodorsal part of the MeA, indicating a data gap for this level of micro-anatomical detail.

**5.2 The Septal Complex and Extended Amygdala: The Social and Stress Relays**

The septal complex and the extended amygdala, which includes the bed nucleus of the stria terminalis (BNST), function as critical relays for emotional, social, and stress-related information.

The **lateral septal nucleus (LS)**, a ventral forebrain structure, modulates a wide range of complex social and affective behaviors. It is topographically organized and is predominantly composed of GABAergic projection neurons. The LS integrates a wealth of information from key brain regions, including the hippocampus, prefrontal cortex, and amygdala, positioning it as a pivotal node in the limbic system for integrating environmental, emotional, and social context.

The **bed nucleus of the stria terminalis (BNST)** is a complex forebrain structure that acts as a crucial relay between limbic centers and hypothalamic/brainstem nuclei associated with autonomic and neuroendocrine functions. The BNST is a key player in stress- and anxiety-related behaviors. It receives connections from the amygdala and prefrontal cortex and projects to hypothalamic nuclei that mediate goal-oriented behaviors. Notably, different BNST subdivisions have opposing effects on autonomic function. For example, stimulation of the medial division increases arterial pressure, whereas stimulation of the lateral division decreases it. This suggests a fine-grained topographical organization of autonomic control within the BNST, with different parts modulating distinct physiological responses.

**VI. Conclusion: Synthesis, Integration, and Future Directions**

This expert-level analysis has demonstrated that the human brain regions examined are not isolated components but are intricately interconnected parts of larger, dynamically interacting systems. The report's analysis, guided by developmental principles, has moved beyond a simple listing of functions to reveal the sophisticated logic of neural circuitry.

Key findings include the re-conceptualization of the thalamus as an active modulator and integrator, rather than a passive relay, a function epitomized by the TRN's inhibitory gating and the nucleus of reuniens' unique role in bridging the hippocampus and prefrontal cortex. The superior colliculus was shown to be a multi-sensory hub for rapid sensorimotor transformation, a process fundamental to adaptive behavior. The analysis of the globus pallidus highlighted the critical importance of a tonic excitatory input from the subthalamic nucleus in maintaining its non-pathological firing rate, providing a physiological basis for understanding movement disorders.

**6.1 Identified Data Gaps and Areas for Future Research**

A significant finding in this report is the complete lack of neuroanatomical data for certain terms in the provided material. The query's mention of **Nucleus x** and the neuroanatomical **mantle zone** structures (e.g., p2ZL, p3ZL). During the embryonic development of neural tube, as the cells adjacent to the lumen continue to divide, the migrating cells form a second layer around the original neural tube. This layer becomes progressively thicker as more cells are added to it from the germinal neuroepithelium. This new layer is called the mantle (or intermediate) zone, and the germinal epithelium is now called the ventricular zone (and, later, the ependyma). The mantle zone cells differentiate into both neurons and glia. Nucleus x: Often refers to a specific nucleus in the brainstem, but its exact function would depend on context (e.g., vestibular nucleus x).

Furthermore, a recurring theme throughout the analysis is the absence of specific functional and connectional data for highly granular sublayers, such as sublayer b of the MeA posterodorsal part and the SC intermediate gray layer. This limitation reflects a current frontier in neuroscience, where imaging and electrophysiological techniques are still working to resolve function at this level of resolution in the living human brain. Future research should aim to elucidate these fine-grained distinctions and their behavioral consequences to provide a more complete picture of neural function.

Finally, while the core function of the cerebellum's **flocculus** and **paraflocculus** in smooth pursuit eye movements was established, the specific mechanisms and connectivity of their molecular layer were not detailed. This is another area for future research to deepen the understanding of how these structures perform their motor control functions.

**6.2 Summary of Key Analytical Findings**

1. **Thalamic Gating:** The TRN fundamentally transforms the view of the thalamus from a simple relay to an active filter that modulates and refines the flow of information between the cortex and subcortical structures.
2. **Multisensory Superior Colliculus:** The SC is a highly efficient hub for rapid sensorimotor integration, combining visual, auditory, and somatosensory inputs to generate directed behavioral responses.
3. **Dynamic Pallidal Firing:** The delicate balance of excitatory and inhibitory inputs to the globus pallidus, particularly the tonic excitatory drive from the STN, is essential for maintaining normal motor control and its disruption is a key mechanism in the pathophysiology of movement disorders.
4. **RE as a Cortical Connector:** The nucleus of reuniens performs a unique and critical role by providing the main reciprocal link between the hippocampus and the prefrontal cortex, which lack direct anatomical connections, thereby facilitating a crucial dialogue for memory and executive function.

This report demonstrates that a complete neuroanatomical analysis requires not only a detailed understanding of individual structures but also a systems-level perspective that integrates anatomy, connectivity, and developmental principles to reveal the elegant and often surprising logic of the brain's neural circuits.

**References**

Puelles L. Functional Implications of the Prosomeric Brain Model. Biomolecules. 2024 Mar 11;14(3):331. <https://doi.org/10.3390/biom14030331> . PMID: 38540751; PMCID: PMC10968462

References for additional sources utilized by the Gemini Pro 2.5 AI assistant during the preparation of the Report are listed in the Appendix.
