# Supplementary material for: Cooperation of transposable elements to endow global networks of initiators of hybrid assembly pathways of endogenous multiprotein complexes
Source: Front Cell Infect Microbiol. 2026 Jul 30;16:1793868. doi: 10.3389/fcimb.2026.1793868 (PMC13468005; doi:10.3389/fcimb.2026.1793868)
Supplement: Supplementary file 5 [file DataSheet5.zip › 3. AI Human BDRRs top 25 TFs analysis.docx]

**3. Analysis of the top 25 transcription factors that are differentially enriched in human versus chimpanzee BDRRs.**

Explorations of the roles of the top 25 human BDRR-enriched TFs in brain development revealed their potential impacts on the regulatory networks governing development of identified 202 brain regions in 3 main areas: i) Masters regulators of cell fate; ii) Regulators of regional specialization and Individual cell-type specification; iii) Regulatory cross-talks to homeostasis control and stress responses.

**Master Regulators of Cell Fate** comprise one group of the top 25 human BDRR-enriched TFs. This group includes TFs that are foundational to the earliest stages of embryonic development. Their main functions are not specific to a single brain region but rather govern the fundamental blueprint for the entire nervous system development. Any dysregulation within this group of TFs would have catastrophic, widespread effects often resulting in embryonic lethality phenotypes.

**SOX2 and POU5F1**, along with NANOG, form the core pluripotency network in embryonic stem cells. Their primary role is to maintain the cell's ability to become any cell type in the body. **SOX2** is a master regulator of neural progenitor cells throughout development, ensuring the formation of the central nervous system (CNS).

Licensed by Google

Continued SOX2 expression marks the progenitor state, allowing for the generation of neurons and glial cells in a coordinated manner. **POU5F1** (OCT4) is associated with early pluripotency and is typically downregulated as cells begin to differentiate. An improper shutdown of POU5F1 in neural precursors could lead to teratoma formation or a failure to generate a functional nervous system. **OTX2** is crucial for defining the boundary between the midbrain and forebrain, including the developing limbic system. It acts as a "selector gene," ensuring that cells in the rostral neural tube take on a forebrain identity. OTX2 also plays a role in the specification of specific midbrain neuronal populations, including dopaminergic neurons. A disruption here would directly impact the formation and function of the **substantia nigra** and other midbrain structures.

**Regulators of Regional Specialization and Individual Cell-Type Specification**

These transcription factors are active later in development, guiding the differentiation of progenitor cells into specific types of neurons and glia, and dictating the identity of distinct brain regions.

**LHX2 & LHX9:** Members of the LIM-homeodomain family, these TFs are critical for the formation of the forebrain and its intricate structures. **LHX2** is a key "selector gene" for the cerebral cortex and hippocampus. It not only promotes a cortical fate but also actively suppresses the formation of non-cortical structures. It's particularly important for the proper timing of the neuron-glia cell fate switch in the hippocampus, a brain region central to the emotional and memory networks we have previously analyzed. **LHX9** has a more limited role but is also involved in diencephalic (thalamus) and telencephalic (limbic system) development.

**PHOX2B:** This is a classic example of a TF with a very specific role. PHOX2B is essential for the development of autonomic neurons, including those in the brainstem that regulate breathing, heart rate, and blood pressure. While not directly a regulator of the limbic system, its dysfunction leads to autonomic nervous system disorders. Its role in the enteric nervous system also provides a direct link to the **gut-brain axis**, which is increasingly recognized as a key player in neurological and emotional health.

**ISL1, EOMES, & CDX2:** These TFs guide the differentiation of specific cell lineages. **ISL1** is involved in motor neuron and sensory neuron development and can also be found in some interneurons within the central nervous system. **EOMES** (Tbr2) is a key regulator of intermediate neural progenitors, which are critical for generating a large number of cortical projection neurons. **CDX2** is primarily a master regulator of posterior-to-anterior embryonic axis formation and gut development, providing another link to the gut-brain axis.

**Regulatory Cross-Talk to Homeostasis Control and Stress Response**

This group of transcription factors has dual roles, operating in both developmental contexts and in the mature, functioning brain, where they interact with the homeostatic networks we've been analyzing.

**ATF4:** This TF is a core component of the **Integrated Stress Response (ISR)**. While it has roles in development, its most significant interrelationship is with neurodegeneration. ATF4 is upregulated in response to various stressors, including oxidative stress and protein misfolding, which are hallmarks of neurodegenerative diseases. It acts as a complex switch, promoting either cell survival or death depending on the context. In the **substantia nigra**, elevated ATF4 can promote the death of dopaminergic neurons, directly impacting the progression of diseases like Parkinson's. This places ATF4 at the intersection of our analyses.

**SMAD2:** This TF is a key mediator of the **TGF-β signaling pathway**, which is involved in a vast array of cellular processes, including cell proliferation, differentiation, and apoptosis. In the developing brain, SMAD2 regulates the fate of neural stem cells. In the mature brain, it influences **hippocampal neuroplasticity**, impacting both neurogenesis and learning and memory. Its dysregulation can contribute to neuronal dysfunction and loss, and it provides a direct link between a major signaling pathway and the function of the limbic system.

**GATA6 & GATA4:** Like CDX2, these are primarily known for their roles in endoderm and mesoderm development, guiding the formation of the heart and gut. However, given the growing understanding of the **gut-brain axis**, their influence on gut function (gut microbiota, intestinal permeability, etc.) could indirectly impact the central nervous system and influence emotional and neurodegenerative states.

**TEAD4:** As a key effector of the **Hippo signaling pathway**, TEAD4 primarily regulates organ size and cell proliferation. While its canonical role is not in neuroscience, its involvement in cell growth and tissue homeostasis means it can impact neural progenitor proliferation and the cellular dynamics of the brain.

The interplay between these early developmental TFs and the later homeostatic networks is complex and can be seen as a two-part process. First, proper development, orchestrated by TFs like **OTX2** and **LHX2**, is a prerequisite for a healthy brain. If this initial scaffolding is faulty, it can predispose specific brain regions, such as the **limbic system** and **substantia nigra**, to later dysfunction. Second, TFs with dual functions, such as **ATF4** and **SMAD2**, act as a bridge, responding to stressors in the mature brain and influencing the homeostatic and emotional networks. On a developmental scale, the regulatory effects of these TFs on brain development and functions extends from the neural tube formation to adolescence and adulthood.
