# Supplementary material for: Cooperation of transposable elements to endow global networks of initiators of hybrid assembly pathways of endogenous multiprotein complexes
Source: Front Cell Infect Microbiol. 2026 Jul 30;16:1793868. doi: 10.3389/fcimb.2026.1793868 (PMC13468005; doi:10.3389/fcimb.2026.1793868)
Supplement: Supplementary file 5 [file DataSheet5.zip › 4. AI Inferred structural functional implications.docx]

**4. Inference of structural-functional implications of distinct developmental trajectories of 202 brain regions for human and chimpanzee brain development and functions**

This report was conceived, prompted, and developed using Google's Gemini (version 1.5 Pro) on September 21, 2025 (Google. (2025). *Gemini* (Version 1.5 Pro) [Large language model]. <https://gemini.google.com/> . Its output was reviewed, analyzed, edited, and refined by the author, who are solely responsible for the content.

While there's no comprehensive datasets for all 202 brain regions, we can draw some general and specific conclusions from publicly available research findings. One of the key takeaways is that human brains are not simply scaled-up versions of chimpanzee brains; they show **disproportionate enlargement** in specific regions and unique changes in cortical organization.

**Overall Size of Brain and Key Regions**

Human brains are roughly **three times larger** than chimpanzee brains. This size difference isn't uniform across all areas; it's driven by a significant expansion of the cerebral cortex, especially in areas associated with higher cognitive functions.

- **Prefrontal Cortex (PFC)**: The PFC is a prime example of disproportionate growth in humans. The proportion of the cortex occupied by the PFC is up to **1.9 times greater** in humans than in macaques and **1.2 times greater** than in chimpanzees. This is especially true for the underlying **white matter**, which is **1.7 times larger** in humans than in chimpanzees, suggesting a massive increase in connectivity.
- **Language-Related Areas**: Brain regions linked to language, such as **Broca's area**, are also significantly larger in humans. Studies have shown these regions to be up to **6 times larger** in humans than in their chimpanzee counterparts.
- **Primary Sensory and Motor Areas**: In contrast, primary motor cortex, primary somatosensory cortex, and primary visual cortex are **similar in size** between humans and chimpanzees relative to overall brain size. This suggests that the expansion in the human brain is not about making basic functions "bigger" but about enhancing higher-level association and processing.

**Cortical Thickness and Organization**

Human and chimpanzee brains also differ in the organization and thickness of the cerebral cortex.

- **Cortical Thickness**: Despite having a much larger surface area, human brains have a **thinner cortex** than chimpanzees overall, particularly in the frontal lobe. This seeming paradox is related to a phenomenon called **gyrification**. Humans have significantly **more folded (gyrified) brains**. The increased folding allows for a greater cortical surface area to be packed into the skull, which in turn leads to a thinner cortex as the folds deepen and stretch the surface.
- **Layer Organization**: Differences in cortical layers are also notable. For example, in the **entorhinal cortex**, while both species have a layered structure, there are species-specific differences in the thickness and appearance of various sublayers (e.g., Layer II and Layer III), reflecting different patterns of connectivity and information processing.

**Subcortical Regions**

While less data is available, some subcortical structures show unique evolutionary changes.

- **Amygdala**: The human amygdala is distinct from that of apes in its internal organization. While its overall size is proportionally similar, certain nuclei within the amygdala have undergone reorganization. For example, the **lateral nucleus**, which receives the most input from the neocortex, is disproportionately larger in humans. In contrast, the basal nucleus is the largest in chimpanzees. This suggests an evolutionary specialization in humans for processing a heightened amount of neocortical information related to social and emotional behavior.
- **Thalamus**: Specific thalamic nuclei, like the **mediodorsal nucleus**, are thought to be larger in humans. While a precise quantitative comparison is not available for all nuclei, the general trend is that regions with strong connections to the expanded prefrontal cortex are also likely to be larger and more complex.

**Conclusion:** Inferred functional implications based on the different developmental timelines of brain regions in humans and chimpanzees can be significant, suggesting that human cognitive abilities are not just a matter of having a bigger brain, but a brain with distinct organizational and maturational priorities.

**Limitations of publicly available chimpanzee brain development studies**

A detailed analysis of the developmental timelines for the 202 brain regions in chimpanzee seems not feasible at this time. The reason is that there is **no comprehensive, publicly available data set** or atlas that maps the precise developmental timelines for all these individual regions in chimpanzees.

Below is a general overview of chimpanzee brain development, based on what is known from comparative neurobiology and human studies.

**General Timeline of Brain Development**

Chimpanzee brain development is broadly similar to that of humans, but with some key differences in timing. The overall process can be broken down into three major phases: **prenatal**, **infancy and childhood**, and **adolescence**.

- **Prenatal Period**: This is the most rapid phase of brain growth, where the basic structure and major regions are formed.
  - **Neurogenesis**: The creation of new neurons. This process is largely complete by the time of birth.
  - **Migration**: Neurons move from their birthplace to their final location in the brain, forming the distinct brain regions listed in the prompt.
  - **Early Synaptogenesis**: The initial formation of synapses (connections between neurons) begins.
- **Infancy and Childhood**: This period is characterized by continued growth and the refinement of neural circuits.
  - **Synaptogenesis**: The brain undergoes a massive burst of synapse formation, reaching a peak.
  - **Synaptic Pruning**: The brain begins to eliminate unnecessary or weak synapses, a process that makes the neural network more efficient. This is crucial for specialization and is influenced by environmental experiences.
  - **Myelination**: The process of insulating nerve fibers with a fatty sheath (myelin) accelerates, improving the speed and efficiency of neural communication. Myelination often follows a pattern, with primary sensory and motor areas myelinating before higher-order cognitive areas.
- **Adolescence**: This is the final stage of significant brain maturation.
  - **Continued Pruning and Myelination**: Pruning continues, particularly in the prefrontal cortex, and myelination is completed in higher-level cognitive areas.
  - **Prefrontal Cortex Maturation**: The prefrontal cortex, which is responsible for executive functions like decision-making, planning, and impulse control, is one of the last areas to fully mature. This maturation process extends into the early twenties.

**Reasons why specific data for chimpanzee brain development are not available**

The primary reason for the lack of detailed data on these specific regions is the **ethical and logistical challenges** of conducting longitudinal neuroimaging studies on chimpanzees, especially for developmental purposes. While some post-mortem studies exist, they cannot provide the detailed, dynamic picture of a living brain that would be required to create a comprehensive developmental atlas like those available for humans.

Some aspects of developmental timelines could be inferred from chimpanzee brain organoid studies, particularly, early stages of brain development. However, there is no detailed comprehensive atlas of chimpanzee brain development. Studies using brain organoids have provided significant insights into the **early stages** of neurogenesis, particularly in comparing the process between humans and chimpanzees. However, these studies cannot provide a complete developmental timeline for the 202 specific, highly diverse, and complex brain regions, which mature later and are organized and function in a fully developed brain.

**Key Findings from Chimpanzee Brain Organoid Studies**

- **Slower Neurogenesis in Humans**: A key discovery is that **human brain organoids develop more slowly** than chimpanzee organoids. This is particularly evident during the early stages of cell division. A crucial phase of mitosis, called prometaphase-metaphase, is about 50% longer in human neural progenitor cells compared to chimpanzees. This extended period gives human progenitor cells more time to proliferate before differentiating into neurons, which is believed to contribute to the larger size of the human neocortex.
- **Gene Expression Differences**: Researchers have identified genes with **different expression patterns** between human and chimpanzee organoids. For example, the gene *ZEB2* is turned on earlier in chimpanzee organoids. This leads to an accelerated transition of progenitor cells into neurons, resulting in a smaller brain size. When this gene's effect is delayed in gorilla organoids, they develop more like human organoids, growing larger.
- **Recapitulating Early Development**: Brain organoids successfully mimic many of the early developmental processes found in a living brain, including the formation of different neural cell types (different types of neurons and glial cells) and the initial organization of brain tissue. This allows to study fundamental differences in the cellular "timing" of brain development that would otherwise be impossible to observe.

**Limitations of Organoid Studies**

While invaluable, brain organoids have limitations that prevent them from providing a complete timeline for the identified 202 brain regions.

- **Lack of Structure**: Organoids are small, three-dimensional clusters of cells that lack a complete vascular system and are not organized into the complex, interconnected regions of a full brain. They can't replicate the intricate pathways and circuits (like the thalamus or specific parts of the brainstem) that are crucial for later developmental stages.
- **Early Development Focus**: Most organoid studies focus on the very early stages of neurogenesis and the initial formation of neurons, typically spanning a few months in culture. They do not replicate the prolonged processes of **synaptic pruning** and **myelination** that occur over years and are critical for the final maturation of specific brain regions.
- **Limited Maturity**: Organoids do not achieve the full cellular maturity of a living brain. The regions you listed, such as the various parts of the globus pallidus or the different sublayers of the medial amygdalar nucleus, undergo complex maturation processes that involve interactions with other brain structures and environmental input that cannot be replicated in a dish.

**Summary of key structural-functional differences between human and chimpanzee brains**

**Disproportionate Expansion of the Cortex**

The human brain's overall size advantage over the chimpanzee's is primarily due to the expansion of the **cerebral cortex,** developmental origin of which could be traced to the mantle layer or mantle zone, and its underlying **white matter,** which is originated from the marginal layer or marginal zone. This is especially pronounced in the **prefrontal cortex**, which is involved in complex functions like **decision-making, planning, and abstract thought**. This suggests that human evolution prioritized the development of these "executive" functions. The larger prefrontal cortex and its increased connectivity (as evidenced by a greater volume of white matter) are key anatomical underpinnings for the cognitive abilities that set humans apart.

**Emotional and Social Intelligence**

Beyond sheer cognitive ability, a major functional implication of human brain differences lies in **emotional regulation and social cognition**. While primates have a "social brain" network, research suggests that connections between certain brain regions associated with these functions are uniquely human and especially pronounced when compared to chimpanzees. The different size and connectivity of regions like the **amygdala** and **orbitofrontal cortex** in humans may have led to an enhanced capacity for empathy, emotional control, and complex social interactions, which are critical for the formation of large, cooperative societies.

**Language and Hemispheric Specialization**

The evolution of language is a hallmark of human-specific brain function. This is reflected in the disproportionate size of language-related areas like **Broca's area** and the unique connectivity patterns of fiber tracts such as the **arcuate fasciculus**. Humans also show a much higher degree of **hemispheric specialization** (laterality) for functions like language and handedness. While chimpanzees show some lateralization, the multi-region, interconnected lateralized networks seen in the human brain are believed to be the neuroanatomical basis for our advanced verbal communication and tool-making abilities.

The video titled "The Evolution of the Cerebral Cortex: How Primates Changed the Brain Game" explains how the human cerebral cortex expanded and became more complex.

[The Evolution of the Cerebral Cortex: How Primates Changed the Brain Game](https://www.youtube.com/watch?v=h_rwRgD8yN4)
