# Supplementary material for: Cooperation of transposable elements to endow global networks of initiators of hybrid assembly pathways of endogenous multiprotein complexes
Source: Front Cell Infect Microbiol. 2026 Jul 30;16:1793868. doi: 10.3389/fcimb.2026.1793868 (PMC13468005; doi:10.3389/fcimb.2026.1793868)
Supplement: Supplementary file 5 [file DataSheet5.zip › 5. AI Chronological Development of Brain Regions.docx]

**5. The Chronological Development of Brain Regions**

Brain development is a complex and highly orchestrated process that unfolds over a long period, beginning in the embryonic stage and continuing into adulthood. The regions you've listed emerge and mature at different times, with a general **cranial-to-caudal** and **medial-to-lateral** progression. This means that regions closer to the top of the head (the forebrain) tend to develop later than those closer to the spinal cord (the hindbrain), and deeper, more medial structures often precede more superficial, lateral ones.

**Early Development (Embryonic and Early Fetal Periods)**

The earliest stages of brain development involve the formation of the **hindbrain**, **midbrain**, and **forebrain** from the neural tube. The hindbrain, which includes the brainstem, is one of the first areas to differentiate. It gives rise to regions essential for basic life-sustaining functions.

- **Hindbrain**: The **Brain stem** and the entire **Hindbrain** itself are among the first structures to form. Within the hindbrain, the **Midbrain** and **Cochlear nuclei** begin to differentiate early on.
- **Prosomeres**: The brain is initially segmented into prosomeres. **Prosomere 3**, which becomes the prethalamus, is a key early structure. Its various parts, such as the **alar plate of p3**, **prethalamic eminence**, and **prethalamus**, are foundational.
- **Neural Tube Segmentation**: The initial regionalization of the neural tube sets the stage for everything else. This includes the formation of the **alar plate of m1**, **alar plate of m3**, and the various zones within the prosomeres, such as the **p3 part of the ZL core** and the **prethalamic (p3) zona limitans domain**.

**Mid-Fetal Development**

As development progresses, the brain's major divisions expand and begin to form more specific nuclei and structures. The **midbrain** and **dorsal thalamus** start to develop in earnest, with many nuclei forming their distinct identities.

- **Thalamus and Midbrain**: The **Thalamus** and **Midbrain** develop and differentiate. The **Mediodorsal nucleus** and **Mediodorsal nucleus of the thalamus** are crucial, as are the various sub-parts like the **lateral part of the MD** and the **medial part of the Mediodorsal nucleus of the thalamus**. The **Reticular nucleus of the thalamus** and the **Reticular complex** also emerge, playing a key role in thalamic function.
- **Basal Ganglia and Related Structures**: The **Pallidum, dorsal region**, and the **Globus pallidus, external segment** begin to form. These are essential for motor control and will continue to mature later in life.
- **Amygdala**: The **Medial amygdalar nucleus** and its sub-parts (**posterodorsal part**, **sublayer a**, **sublayer b**, and **sublayer c**) begin their formation. The amygdala is central to emotional processing and develops significantly throughout childhood.

**Late Fetal and Postnatal Development**

This period is marked by the maturation of cortical regions and the final differentiation of many subcortical nuclei. **Synaptogenesis** and **myelination** occur at a rapid pace, refining the neural circuits.

- **Sensory and Motor Pathways**: The **Superior colliculus** and **Inferior colliculus**, and their various layers (**deep gray layer**, **intermediate gray layer**, **intermediate white layer**), mature to handle visual and auditory reflexes. The **Cochlear nuclei** and their **granular lamina** also mature, essential for hearing.
- **Cerebellum and Cerebellar Structures**: The **Flocculus** and **Paraflocculus**, with their respective molecular layers, develop. The cerebellum is vital for coordination and balance, and its maturation continues well into childhood.
- **Cortical Regions**: The **Entorhinal area, medial part, dorsal zone, layer 1** and **layer 2** begin to mature. The entorhinal cortex is a key part of the memory system, and its development is closely tied to the hippocampus.
- **Thalamocortical Connections**: The **Ventral posterolateral nucleus** and the **Thalamus, sensory-motor cortex related** structures mature. These nuclei are the primary relays for sensory information on its way to the cerebral cortex. Their development and connection with the cortex are crucial for sensation and motor control.

**Maturation into Adolescence and Adulthood**

Brain development doesn't stop after birth. Regions involved in complex functions, such as executive control, social cognition, and emotional regulation, continue to mature throughout childhood and adolescence. This is a period of "pruning" where unused synapses are eliminated and others are strengthened.

- **Frontal and Limbic Maturation**: The **accumbens nucleus, shell domain** and **bed nucleus of the stria terminalis, medioseptal division** continue to mature, playing key roles in motivation and stress response. The **Globus pallidus, internal segment** matures to complete the basal ganglia circuitry, allowing for finer motor control.
- **Complex Integrated Systems**: The final maturation of many of the nuclei and tracts listed, such as the **lateral septal nucleus, dorsal part**, and the **pedunculopontine nucleus**, solidify the complex, integrated systems that govern our behavior, thought, and emotion. The **reuniens nucleus, main part** and **retroreuniens nucleus** also mature, forming critical links between the hippocampus and prefrontal cortex, which are essential for memory and cognitive function.

While this timeline presents a general overview, it's important to remember that the development of different brain regions is highly interactive. The maturation of one area often depends on and influences the development of others, leading to the intricate and interconnected neural network that defines the human brain.

Below, this expert-level analysis of brain region development is cataloged into three tables based on their approximate chronological emergence during human brain development.

**Table 1: Early Development (Embryonic and Early Fetal Period)**

This period is marked by the initial formation of the neural tube and its primary divisions, the hindbrain, midbrain, and forebrain. These structures form the foundational scaffolding for the entire brain.

| Region | Description & Developmental Significance |
| --- | --- |
| Hindbrain | The most caudal and evolutionarily ancient part of the brain, responsible for basic, essential life functions. Its early emergence sets the stage for the rest of the nervous system. |
| Brain stem | A vital part of the hindbrain, responsible for breathing, heart rate, and consciousness. Its early formation ensures the survival of the developing organism. |
| Prosomere 3 | A fundamental segment of the embryonic forebrain (prethalamus), its early differentiation dictates the development of the thalamus and other diencephalic structures. |
| Alar plate of p3 | The dorsal part of prosomere 3, which gives rise to the prethalamus. Its early development is crucial for the formation of key thalamic relay nuclei. |
| Reticular nucleus of the thalamus | This nucleus forms early within the thalamus and plays a critical role in regulating thalamic activity and relaying information to the cortex. |
| Zona incerta | Part of the subthalamus, involved in motor control. Its development alongside other subthalamic structures establishes early motor circuits. |
| Cochlear nuclei | Located in the hindbrain, these nuclei are among the first to develop as part of the auditory pathway. Their early maturation is essential for processing sound. |
| Medulla, sensory related | This part of the brainstem handles sensory information from the body, and its early formation is crucial for basic sensory perception. |

**Table 2: Mid-Fetal Development**

During this phase, the major brain divisions expand and differentiate into specific nuclei and structures. Synapse formation begins, and the primary neural circuits are established.

| Region | Description & Developmental Significance |
| --- | --- |
| Thalamus, sensory-motor cortex related | The thalamus is a major relay station. Its mid-fetal development establishes crucial pathways for sensory and motor information to reach the cerebral cortex. |
| Mediodorsal nucleus | A key thalamic nucleus involved in executive function and memory. Its maturation during this period is critical for laying the groundwork for higher cognitive function. |
| Globus pallidus, external segment | A core component of the basal ganglia, crucial for motor control. Its development is part of the emerging motor system. |
| Superior colliculus, motor related | A midbrain structure involved in visual reflexes and orienting movements. Its layers develop sequentially, with the deep layers involved in motor output. |
| Inferior colliculus, central nucleus | The main auditory nucleus in the midbrain, its development during this period is essential for advanced auditory processing and reflexes. |
| Anterior pretectal nucleus | Part of the pretectal region, involved in visual reflexes. Its development is integrated with the maturation of other midbrain visual centers. |
| Medial amygdalar nucleus | A limbic structure critical for emotional processing. Its various sub-regions and sub-layers begin to differentiate, forming the basis for emotional responses. |
| Lateral habenula | A small but significant nucleus in the epithalamus, involved in reward processing and aversion. Its development lays the foundation for future motivational circuits. |
| Cerebellum | The cerebellum's overall development, including its subdivisions like the **Flocculus** and **Paraflocculus**, begins in this period. It is vital for motor coordination and balance. |

**Table 3: Late Fetal and Postnatal Development**

This is a period of intense synaptogenesis and pruning, where the brain refines its circuitry. Myelination begins, and structures responsible for complex cognitive and social functions mature. This process continues well into adolescence and early adulthood.

| Region | Description & Developmental Significance |
| --- | --- |
| Ventral posterolateral nucleus of the thalamus | This nucleus relays tactile and proprioceptive information to the cortex. Its late maturation refines sensory perception and body awareness. |
| Entorhinal area, medial part, dorsal zone | A crucial part of the memory system, providing a major input to the hippocampus. Its maturation is key for the development of episodic memory. |
| Nucleus of reuniens | Connects the hippocampus and the prefrontal cortex, a crucial link for memory consolidation and retrieval. Its postnatal maturation is vital for higher cognitive function. |
| Globus pallidus, internal segment | The output nucleus of the basal ganglia, responsible for inhibiting unwanted movements. Its full maturation is a late-stage process that allows for fine-tuned motor control. |
| Lateral septal nucleus, dorsal part | Involved in social behavior and stress response. Its maturation throughout childhood and adolescence influences the development of social cognition. |
| Pedunculopontine nucleus | Part of the brainstem, involved in arousal, attention, and motor control. Its maturation is integrated with the development of the broader motor and arousal systems. |
| Accumbens nucleus, shell domain | A key component of the reward system. Its late development contributes to the formation of goal-directed behaviors and motivation. |
