# Supplementary material for: Cooperation of transposable elements to endow global networks of initiators of hybrid assembly pathways of endogenous multiprotein complexes
Source: Front Cell Infect Microbiol. 2026 Jul 30;16:1793868. doi: 10.3389/fcimb.2026.1793868 (PMC13468005; doi:10.3389/fcimb.2026.1793868)
Supplement: Supplementary file 5 [file DataSheet5.zip › 6. AI Gemini assessment of significance.docx]

**6. Artificial Intelligence assessment of potential significance of reported observations suggesting interconnected patterns of developmental regulation of identified brain regions by 25 transcription factors.**

If all of these diverse and intricate brain regions—from the motor-related midbrain to the limbic Medial Amygdalar Nucleus—are interconnected and their development is governed by just 25 transcription factors, the significance of the original report's findings would be revolutionary on several levels.

This premise suggests a fundamental shift in our understanding of brain architecture and disease. It implies that the incredible complexity we see in neuroanatomy is not built from thousands of independent genetic instructions, but rather from a remarkably small, highly efficient, and reusable genetic "master blueprint."

Here is a breakdown of the potential significance as articulated by the Artificial Intelligence LLM Gemini (Google):

**3.4.1. Simplification of Biological Complexity**

The number one takeaway is that this discovery would dramatically simplify the study of neurodevelopment. Instead of needing to untangle the function of thousands of genes to understand how a brain region forms and connects, we could focus on a core set of 25 "master switches." These transcription factors would act as a genetic orchestra conductor, with different combinations and timing of their activation leading to the formation of distinct nuclei like the **Spinal nucleus of the trigeminal** or the **Globus pallidus**.

**3.4.2. A "Root Cause" Approach to Neurological Disease**

For neurodegenerative and neurodevelopmental disorders, this would offer a new, powerful therapeutic target. Many brain diseases, from Parkinson's (affecting **Midbrain, motor related** structures) to certain types of amnesia (linked to the **Anterior group of the dorsal thalamus**), are characterized by widespread network dysfunction. Instead of trying to fix a faulty circuit or a single protein, we could potentially target the 25 transcription factors that built the circuit in the first place. This would be like finding the single command in the source code that causes a bug, rather than just patching the bug's visible symptoms.

**3.4.3. The Rise of "Genomic Neuroanatomy"**

This would fundamentally bridge the gap between genetics and neuroanatomy. The traditional study of a neuroanatomical nucleus would no longer just involve its connections and function, but also its "transcription factor signature"—the unique combination of these 25 factors that define its identity. For example, the report noted the potential functional differences between the sublayers of the **Medial amygdalar nucleus, posterodorsal part**. This new information would suggest that these sublayers have distinct "molecular identities" based on the subtle variations in the activity of these 25 factors, which in turn dictate their connectivity and function.

**3.4.4. New Frontiers in Research and Computational Modeling**

The data from this report, when combined with this new genetic information, could serve as a "Rosetta Stone" for understanding the human brain. Researchers could use a systems biology approach to model how the interactions of these 25 factors give rise to the observed neuroanatomy. This would be a massive leap in our ability to computationally simulate and predict brain development and disease, potentially allowing us to test therapies in a virtual environment before ever touching a cell.
