# Supplementary material for: Cooperation of transposable elements to endow global networks of initiators of hybrid assembly pathways of endogenous multiprotein complexes
Source: Front Cell Infect Microbiol. 2026 Jul 30;16:1793868. doi: 10.3389/fcimb.2026.1793868 (PMC13468005; doi:10.3389/fcimb.2026.1793868)
Supplement: Supplementary file 5 [file DataSheet5.zip › 7. AI Reference to sources used in analyses.docx]

**7. Sources used in the analyses and reports**

[pmc.ncbi.nlm.nih.gov](https://pmc.ncbi.nlm.nih.gov/articles/PMC10968462/)

[Functional Implications of the Prosomeric Brain Model - PMC - PubMed Central](https://pmc.ncbi.nlm.nih.gov/articles/PMC10968462/)

[Opens in a new window](https://pmc.ncbi.nlm.nih.gov/articles/PMC10968462/)

<http://atlas.brain-map.org/atlas?atlas=181276130&structure=16318" \t "_blank>

[atlas.brain-map.org](http://atlas.brain-map.org/atlas?atlas=181276130&structure=16318)

[Developing Mouse, E13.5 - Allen Brain Atlas](http://atlas.brain-map.org/atlas?atlas=181276130&structure=16318)

[Opens in a new window](http://atlas.brain-map.org/atlas?atlas=181276130&structure=16318)

[pmc.ncbi.nlm.nih.gov](https://pmc.ncbi.nlm.nih.gov/articles/PMC11615174/)

[Regulation of neuronal fate specification and connectivity of the ...](https://pmc.ncbi.nlm.nih.gov/articles/PMC11615174/)

[Opens in a new window](https://pmc.ncbi.nlm.nih.gov/articles/PMC11615174/)

<https://www.ncbi.nlm.nih.gov/books/NBK544224/" \t "_blank>

[ncbi.nlm.nih.gov](https://www.ncbi.nlm.nih.gov/books/NBK544224/)

[Neuroanatomy, Superior Colliculus - StatPearls - NCBI Bookshelf](https://www.ncbi.nlm.nih.gov/books/NBK544224/)

[Opens in a new window](https://www.ncbi.nlm.nih.gov/books/NBK544224/)

[neuroanatomy.wisc.edu](https://neuroanatomy.wisc.edu/Bs97/TEXT/P23/intro.htm)

[BS 23. Superior Colliculus - Intro](https://neuroanatomy.wisc.edu/Bs97/TEXT/P23/intro.htm)

[Opens in a new window](https://neuroanatomy.wisc.edu/Bs97/TEXT/P23/intro.htm)

<https://www.imaios.com/en/e-anatomy/anatomical-structures/mediodorsal-nucleus-11090678812" \t "_blank>

[imaios.com](https://www.imaios.com/en/e-anatomy/anatomical-structures/mediodorsal-nucleus-11090678812)

[Mediodorsal nucleus - e-Anatomy - IMAIOS](https://www.imaios.com/en/e-anatomy/anatomical-structures/mediodorsal-nucleus-11090678812)

[Opens in a new window](https://www.imaios.com/en/e-anatomy/anatomical-structures/mediodorsal-nucleus-11090678812)

<https://www.researchgate.net/publication/344241881_The_Anatomical_and_Functional_Heterogeneity_of_the_Mediodorsal_Thalamus" \t "_blank>

[researchgate.net](https://www.researchgate.net/publication/344241881_The_Anatomical_and_Functional_Heterogeneity_of_the_Mediodorsal_Thalamus)

[The Anatomical and Functional Heterogeneity of the Mediodorsal Thalamus - ResearchGate](https://www.researchgate.net/publication/344241881_The_Anatomical_and_Functional_Heterogeneity_of_the_Mediodorsal_Thalamus)

[Opens in a new window](https://www.researchgate.net/publication/344241881_The_Anatomical_and_Functional_Heterogeneity_of_the_Mediodorsal_Thalamus)

<https://www.frontiersin.org/journals/neuroscience/articles/10.3389/fnins.2018.00033/full" \t "_blank>

[frontiersin.org](https://www.frontiersin.org/journals/neuroscience/articles/10.3389/fnins.2018.00033/full)

[Cognitive Functions and Neurodevelopmental Disorders Involving the Prefrontal Cortex and Mediodorsal Thalamus - Frontiers](https://www.frontiersin.org/journals/neuroscience/articles/10.3389/fnins.2018.00033/full)

[Opens in a new window](https://www.frontiersin.org/journals/neuroscience/articles/10.3389/fnins.2018.00033/full)

<https://www.neurology.org/doi/10.1212/01.wnl.0000436078.95856.56" \t "_blank>

[neurology.org](https://www.neurology.org/doi/10.1212/01.wnl.0000436078.95856.56)

[Anterior nucleus of the thalamus | Neurology](https://www.neurology.org/doi/10.1212/01.wnl.0000436078.95856.56)

[Opens in a new window](https://www.neurology.org/doi/10.1212/01.wnl.0000436078.95856.56)

[pmc.ncbi.nlm.nih.gov](https://pmc.ncbi.nlm.nih.gov/articles/PMC8363507/#:~:text=The%20anterior%20thalamic%20nuclei%20are,nuclei%20has%20been%20spatial%20processing.)

[pmc.ncbi.nlm.nih.gov](https://pmc.ncbi.nlm.nih.gov/articles/PMC8363507/#:~:text=The%20anterior%20thalamic%20nuclei%20are,nuclei%20has%20been%20spatial%20processing.)

[Opens in a new window](https://pmc.ncbi.nlm.nih.gov/articles/PMC8363507/#:~:text=The%20anterior%20thalamic%20nuclei%20are,nuclei%20has%20been%20spatial%20processing.)

[pmc.ncbi.nlm.nih.gov](https://pmc.ncbi.nlm.nih.gov/articles/PMC11615174/" \l ":~:text=The%20thalamic%20reticular%20nucleus%20(TRN,disturbance%20and%20multiple%20behavioral%20disorders." \t "_blank)

[pmc.ncbi.nlm.nih.gov](https://pmc.ncbi.nlm.nih.gov/articles/PMC11615174/" \l ":~:text=The%20thalamic%20reticular%20nucleus%20(TRN,disturbance%20and%20multiple%20behavioral%20disorders." \t "_blank)

[Opens in a new window](https://pmc.ncbi.nlm.nih.gov/articles/PMC11615174/" \l ":~:text=The%20thalamic%20reticular%20nucleus%20(TRN,disturbance%20and%20multiple%20behavioral%20disorders." \t "_blank)

<https://www.bionity.com/en/encyclopedia/Prethalamus.html" \t "_blank>

[bionity.com](https://www.bionity.com/en/encyclopedia/Prethalamus.html)

[Prethalamus - Bionity](https://www.bionity.com/en/encyclopedia/Prethalamus.html)

[Opens in a new window](https://www.bionity.com/en/encyclopedia/Prethalamus.html)

<https://www.biorxiv.org/content/10.1101/2024.09.06.611701v1.full-text" \l ":~:text=The%20zona%20incerta%20(ZI)%20is,%3B%20Nauta%20and%20Haymaker%201969)." \t "_blank>

[biorxiv.org](https://www.biorxiv.org/content/10.1101/2024.09.06.611701v1.full-text" \l ":~:text=The%20zona%20incerta%20(ZI)%20is,%3B%20Nauta%20and%20Haymaker%201969)." \t "_blank)

[www.biorxiv.org](https://www.biorxiv.org/content/10.1101/2024.09.06.611701v1.full-text" \l ":~:text=The%20zona%20incerta%20(ZI)%20is,%3B%20Nauta%20and%20Haymaker%201969)." \t "_blank)

[Opens in a new window](https://www.biorxiv.org/content/10.1101/2024.09.06.611701v1.full-text" \l ":~:text=The%20zona%20incerta%20(ZI)%20is,%3B%20Nauta%20and%20Haymaker%201969)." \t "_blank)

<https://en.wikipedia.org/wiki/Zona_incerta" \t "_blank>

[en.wikipedia.org](https://en.wikipedia.org/wiki/Zona_incerta)

[Zona incerta - Wikipedia](https://en.wikipedia.org/wiki/Zona_incerta)

[Opens in a new window](https://en.wikipedia.org/wiki/Zona_incerta)

<https://pubmed.ncbi.nlm.nih.gov/31901976/" \t "_blank>

[pubmed.ncbi.nlm.nih.gov](https://pubmed.ncbi.nlm.nih.gov/31901976/)

[Longitudinal developmental analysis of prethalamic eminence derivatives in the chick by mapping of Tbr1 in situ expression - PubMed](https://pubmed.ncbi.nlm.nih.gov/31901976/)

[Opens in a new window](https://pubmed.ncbi.nlm.nih.gov/31901976/)

<https://www.researchgate.net/figure/Tbr1-expression-in-the-prethalamic-eminence-PThE-a-Microphotograph-of-a-sagittal-brain_fig1_338395340" \t "_blank>

[researchgate.net](https://www.researchgate.net/figure/Tbr1-expression-in-the-prethalamic-eminence-PThE-a-Microphotograph-of-a-sagittal-brain_fig1_338395340)

[Tbr1 expression in the prethalamic eminence (PThE). a Microphotograph... - ResearchGate](https://www.researchgate.net/figure/Tbr1-expression-in-the-prethalamic-eminence-PThE-a-Microphotograph-of-a-sagittal-brain_fig1_338395340)

[Opens in a new window](https://www.researchgate.net/figure/Tbr1-expression-in-the-prethalamic-eminence-PThE-a-Microphotograph-of-a-sagittal-brain_fig1_338395340)

<https://www.mayoclinic.org/diseases-conditions/mantle-cell-lymphoma/symptoms-causes/syc-20584872" \t "_blank>

[mayoclinic.org](https://www.mayoclinic.org/diseases-conditions/mantle-cell-lymphoma/symptoms-causes/syc-20584872)

[Mantle cell lymphoma - Symptoms and causes - Mayo Clinic](https://www.mayoclinic.org/diseases-conditions/mantle-cell-lymphoma/symptoms-causes/syc-20584872)

[Opens in a new window](https://www.mayoclinic.org/diseases-conditions/mantle-cell-lymphoma/symptoms-causes/syc-20584872)

<https://www.mdanderson.org/cancer-types/non-hodgkin-lymphoma/mantle-cell-lymphoma.html" \t "_blank>

[mdanderson.org](https://www.mdanderson.org/cancer-types/non-hodgkin-lymphoma/mantle-cell-lymphoma.html)

[Mantle Cell Lymphoma - MD Anderson Cancer Center](https://www.mdanderson.org/cancer-types/non-hodgkin-lymphoma/mantle-cell-lymphoma.html)

[Opens in a new window](https://www.mdanderson.org/cancer-types/non-hodgkin-lymphoma/mantle-cell-lymphoma.html)

<https://www.leukaemia.org.au/blood-cancer/types-of-blood-cancer/lymphoma/non-hodgkin-lymphoma/mantle-cell-lymphoma/" \t "_blank>

[leukaemia.org.au](https://www.leukaemia.org.au/blood-cancer/types-of-blood-cancer/lymphoma/non-hodgkin-lymphoma/mantle-cell-lymphoma/)

[Mantle cell lymphoma - The Leukaemia Foundation](https://www.leukaemia.org.au/blood-cancer/types-of-blood-cancer/lymphoma/non-hodgkin-lymphoma/mantle-cell-lymphoma/)

[Opens in a new window](https://www.leukaemia.org.au/blood-cancer/types-of-blood-cancer/lymphoma/non-hodgkin-lymphoma/mantle-cell-lymphoma/)

<https://imagebank.hematology.org/image/62363/mantle-cell-lymphoma-with-mantle-zone-pattern1" \t "_blank>

[imagebank.hematology.org](https://imagebank.hematology.org/image/62363/mantle-cell-lymphoma-with-mantle-zone-pattern1)

[Mantle cell lymphoma with mantle zone pattern-1 - ASH Image Bank](https://imagebank.hematology.org/image/62363/mantle-cell-lymphoma-with-mantle-zone-pattern1)

[Opens in a new window](https://imagebank.hematology.org/image/62363/mantle-cell-lymphoma-with-mantle-zone-pattern1)

<https://www.cancerresearchuk.org/about-cancer/non-hodgkin-lymphoma/types/mantle-cell" \t "_blank>

[cancerresearchuk.org](https://www.cancerresearchuk.org/about-cancer/non-hodgkin-lymphoma/types/mantle-cell)

[Mantle cell lymphoma | non-Hodgkin lymphoma - Cancer Research UK](https://www.cancerresearchuk.org/about-cancer/non-hodgkin-lymphoma/types/mantle-cell)

[Opens in a new window](https://www.cancerresearchuk.org/about-cancer/non-hodgkin-lymphoma/types/mantle-cell)

[pure.johnshopkins.edu](https://pure.johnshopkins.edu/en/publications/mantle-cell-lymphoma-with-mantle-zone-growth-pattern-a-clinicopat)

[Mantle Cell Lymphoma with Mantle Zone Growth Pattern: A Clinicopathologic Study of 35 Cases - Johns Hopkins University](https://pure.johnshopkins.edu/en/publications/mantle-cell-lymphoma-with-mantle-zone-growth-pattern-a-clinicopat)

[Opens in a new window](https://pure.johnshopkins.edu/en/publications/mantle-cell-lymphoma-with-mantle-zone-growth-pattern-a-clinicopat)

[experiments.springernature.com](https://experiments.springernature.com/articles/10.1007/978-1-0716-2631-3_4)

[Nucleus Reuniens: Circuitry, Function, and Dysfunction | Springer ...](https://experiments.springernature.com/articles/10.1007/978-1-0716-2631-3_4)

[Opens in a new window](https://experiments.springernature.com/articles/10.1007/978-1-0716-2631-3_4)

<https://elifesciences.org/reviewed-preprints/90826v2" \t "_blank>

[elifesciences.org](https://elifesciences.org/reviewed-preprints/90826v2)

[The reuniens nucleus of the thalamus facilitates hippocampo-cortical dialogue during sleep](https://elifesciences.org/reviewed-preprints/90826v2)

[Opens in a new window](https://elifesciences.org/reviewed-preprints/90826v2)

<http://braininfo.rprc.washington.edu/centraldirectory.aspx?id=1965" \t "_blank>

[braininfo.rprc.washington.edu](http://braininfo.rprc.washington.edu/centraldirectory.aspx?id=1965)

[perireuniens nucleus - BrainInfo - University of Washington](http://braininfo.rprc.washington.edu/centraldirectory.aspx?id=1965)

[Opens in a new window](http://braininfo.rprc.washington.edu/centraldirectory.aspx?id=1965)

[bams1.org](https://www.bams1.org/ontology/viewer/Swanson-98-04/details/brainPart/Perireunensis-nucleus-3/)

[www.bams1.org](https://www.bams1.org/ontology/viewer/Swanson-98-04/details/brainPart/Perireunensis-nucleus-3/)

[Opens in a new window](https://www.bams1.org/ontology/viewer/Swanson-98-04/details/brainPart/Perireunensis-nucleus-3/)

<https://www.imaios.com/en/e-anatomy/anatomical-structures/dorsal-pallidum-133585420" \t "_blank>

[imaios.com](https://www.imaios.com/en/e-anatomy/anatomical-structures/dorsal-pallidum-133585420)

[Dorsal pallidum - e-Anatomy - IMAIOS](https://www.imaios.com/en/e-anatomy/anatomical-structures/dorsal-pallidum-133585420)

[Opens in a new window](https://www.imaios.com/en/e-anatomy/anatomical-structures/dorsal-pallidum-133585420)

<https://www.kenhub.com/en/library/anatomy/basal-ganglia" \t "_blank>

[kenhub.com](https://www.kenhub.com/en/library/anatomy/basal-ganglia)

[Basal ganglia: Gross anatomy and function | Kenhub](https://www.kenhub.com/en/library/anatomy/basal-ganglia)

[Opens in a new window](https://www.kenhub.com/en/library/anatomy/basal-ganglia)

<https://www.ncbi.nlm.nih.gov/books/NBK557755/" \l ":~:text=The%20primary%20function%20of%20the,sends%20information%20to%20the%20thalamus." \t "_blank>

[ncbi.nlm.nih.gov](https://www.ncbi.nlm.nih.gov/books/NBK557755/#:~:text=The%20primary%20function%20of%20the,sends%20information%20to%20the%20thalamus.)

[www.ncbi.nlm.nih.gov](https://www.ncbi.nlm.nih.gov/books/NBK557755/#:~:text=The%20primary%20function%20of%20the,sends%20information%20to%20the%20thalamus.)

[Opens in a new window](https://www.ncbi.nlm.nih.gov/books/NBK557755/#:~:text=The%20primary%20function%20of%20the,sends%20information%20to%20the%20thalamus.)

[pmc.ncbi.nlm.nih.gov](https://pmc.ncbi.nlm.nih.gov/articles/PMC3221766/)

[Functional connectivity and integrative properties of globus pallidus ...](https://pmc.ncbi.nlm.nih.gov/articles/PMC3221766/)

[Opens in a new window](https://pmc.ncbi.nlm.nih.gov/articles/PMC3221766/)

[pmc.ncbi.nlm.nih.gov](https://pmc.ncbi.nlm.nih.gov/articles/PMC8190998/)

[The tectum/superior colliculus as the vertebrate solution for spatial sensory integration and action - PMC - PubMed Central](https://pmc.ncbi.nlm.nih.gov/articles/PMC8190998/)

[Opens in a new window](https://pmc.ncbi.nlm.nih.gov/articles/PMC8190998/)

[pmc.ncbi.nlm.nih.gov](https://pmc.ncbi.nlm.nih.gov/articles/PMC6740636/)

[Functional Connectivity between the Superficial and Deeper Layers of the Superior Colliculus: An Anatomical Substrate for Sensorimotor Integration - PubMed Central](https://pmc.ncbi.nlm.nih.gov/articles/PMC6740636/)

[Opens in a new window](https://pmc.ncbi.nlm.nih.gov/articles/PMC6740636/)

<https://pubmed.ncbi.nlm.nih.gov/15010497/" \t "_blank>

[pubmed.ncbi.nlm.nih.gov](https://pubmed.ncbi.nlm.nih.gov/15010497/)

[Organization of the intermediate gray layer of the superior colliculus. I. Intrinsic vertical connections - PubMed](https://pubmed.ncbi.nlm.nih.gov/15010497/)

[Opens in a new window](https://pubmed.ncbi.nlm.nih.gov/15010497/)

<https://en.wikipedia.org/wiki/Superior_colliculus" \t "_blank>

[en.wikipedia.org](https://en.wikipedia.org/wiki/Superior_colliculus)

[Superior colliculus - Wikipedia](https://en.wikipedia.org/wiki/Superior_colliculus)

[Opens in a new window](https://en.wikipedia.org/wiki/Superior_colliculus)

<https://en.wikipedia.org/wiki/Pretectal_area" \t "_blank>

[en.wikipedia.org](https://en.wikipedia.org/wiki/Pretectal_area)

[Pretectal area - Wikipedia](https://en.wikipedia.org/wiki/Pretectal_area)

[Opens in a new window](https://en.wikipedia.org/wiki/Pretectal_area)

<https://taylorandfrancis.com/knowledge/Medicine_and_healthcare/Neurology/Pretectum/" \t "_blank>

[taylorandfrancis.com](https://taylorandfrancis.com/knowledge/Medicine_and_healthcare/Neurology/Pretectum/)

[Pretectum – Knowledge and References - Taylor & Francis](https://taylorandfrancis.com/knowledge/Medicine_and_healthcare/Neurology/Pretectum/)

[Opens in a new window](https://taylorandfrancis.com/knowledge/Medicine_and_healthcare/Neurology/Pretectum/)

<https://en.wikipedia.org/wiki/Inferior_colliculus" \l ":~:text=The%20inferior%20colliculi%20of%20the,cortex%20which%20is%20located%20laterally." \t "_blank>

[en.wikipedia.org](https://en.wikipedia.org/wiki/Inferior_colliculus#:~:text=The%20inferior%20colliculi%20of%20the,cortex%20which%20is%20located%20laterally.)

[en.wikipedia.org](https://en.wikipedia.org/wiki/Inferior_colliculus#:~:text=The%20inferior%20colliculi%20of%20the,cortex%20which%20is%20located%20laterally.)

[Opens in a new window](https://en.wikipedia.org/wiki/Inferior_colliculus#:~:text=The%20inferior%20colliculi%20of%20the,cortex%20which%20is%20located%20laterally.)

<https://www.ncbi.nlm.nih.gov/books/NBK554468/" \t "_blank>

[ncbi.nlm.nih.gov](https://www.ncbi.nlm.nih.gov/books/NBK554468/)

[Neuroanatomy, Inferior Colliculus - StatPearls - NCBI Bookshelf](https://www.ncbi.nlm.nih.gov/books/NBK554468/)

[Opens in a new window](https://www.ncbi.nlm.nih.gov/books/NBK554468/)

<https://www.ncbi.nlm.nih.gov/books/NBK539729/" \t "_blank>

[ncbi.nlm.nih.gov](https://www.ncbi.nlm.nih.gov/books/NBK539729/)

[Neuroanatomy, Spinal Trigeminal Nucleus - StatPearls - NCBI Bookshelf](https://www.ncbi.nlm.nih.gov/books/NBK539729/)

[Opens in a new window](https://www.ncbi.nlm.nih.gov/books/NBK539729/)

<https://radiopaedia.org/articles/spinal-nucleus-of-the-trigeminal-nerve" \t "_blank>

[radiopaedia.org](https://radiopaedia.org/articles/spinal-nucleus-of-the-trigeminal-nerve)

[Spinal nucleus of the trigeminal nerve | Radiology Reference Article | Radiopaedia.org](https://radiopaedia.org/articles/spinal-nucleus-of-the-trigeminal-nerve)

[Opens in a new window](https://radiopaedia.org/articles/spinal-nucleus-of-the-trigeminal-nerve)

<https://www.ncbi.nlm.nih.gov/books/NBK556102/" \t "_blank>

[ncbi.nlm.nih.gov](https://www.ncbi.nlm.nih.gov/books/NBK556102/)

[Neuroanatomy, Reticular Formation - StatPearls - NCBI Bookshelf](https://www.ncbi.nlm.nih.gov/books/NBK556102/)

[Opens in a new window](https://www.ncbi.nlm.nih.gov/books/NBK556102/)

<https://en.wikipedia.org/wiki/Reticular_formation" \t "_blank>

[en.wikipedia.org](https://en.wikipedia.org/wiki/Reticular_formation)

[Reticular formation - Wikipedia](https://en.wikipedia.org/wiki/Reticular_formation)

[Opens in a new window](https://en.wikipedia.org/wiki/Reticular_formation)

<https://pubmed.ncbi.nlm.nih.gov/30981899/" \l ":~:text=Good%20evidence%20relates%20the%20PPN,and%20physiology%20of%20the%20nucleus." \t "_blank>

[pubmed.ncbi.nlm.nih.gov](https://pubmed.ncbi.nlm.nih.gov/30981899/#:~:text=Good%20evidence%20relates%20the%20PPN,and%20physiology%20of%20the%20nucleus.)

[pubmed.ncbi.nlm.nih.gov](https://pubmed.ncbi.nlm.nih.gov/30981899/#:~:text=Good%20evidence%20relates%20the%20PPN,and%20physiology%20of%20the%20nucleus.)

[Opens in a new window](https://pubmed.ncbi.nlm.nih.gov/30981899/#:~:text=Good%20evidence%20relates%20the%20PPN,and%20physiology%20of%20the%20nucleus.)

<https://www.neurology.org/doi/10.1212/WNL.0b013e3182886a76" \t "_blank>

[neurology.org](https://www.neurology.org/doi/10.1212/WNL.0b013e3182886a76)

[Pedunculopontine nucleus - Neurology.org](https://www.neurology.org/doi/10.1212/WNL.0b013e3182886a76)

[Opens in a new window](https://www.neurology.org/doi/10.1212/WNL.0b013e3182886a76)

[pmc.ncbi.nlm.nih.gov](https://pmc.ncbi.nlm.nih.gov/articles/PMC6608208/)

[Functional Properties and Projections of Neurons in the Medial Amygdala - PMC](https://pmc.ncbi.nlm.nih.gov/articles/PMC6608208/)

[Opens in a new window](https://pmc.ncbi.nlm.nih.gov/articles/PMC6608208/)

<https://www.frontiersin.org/journals/neuroanatomy/articles/10.3389/fnana.2012.00033/text" \t "_blank>

[frontiersin.org](https://www.frontiersin.org/journals/neuroanatomy/articles/10.3389/fnana.2012.00033/text)

[www.frontiersin.org](https://www.frontiersin.org/journals/neuroanatomy/articles/10.3389/fnana.2012.00033/text)

[Opens in a new window](https://www.frontiersin.org/journals/neuroanatomy/articles/10.3389/fnana.2012.00033/text)

<http://braininfo.rprc.washington.edu/centraldirectory.aspx?ID=241" \t "_blank>

[braininfo.rprc.washington.edu](http://braininfo.rprc.washington.edu/centraldirectory.aspx?ID=241)

[medial amygdalar nucleus - BrainInfo - University of Washington](http://braininfo.rprc.washington.edu/centraldirectory.aspx?ID=241)

[Opens in a new window](http://braininfo.rprc.washington.edu/centraldirectory.aspx?ID=241)

<https://www.biorxiv.org/content/10.1101/2024.02.15.580381v1.full" \t "_blank>

[biorxiv.org](https://www.biorxiv.org/content/10.1101/2024.02.15.580381v1.full)

[Multimodal classification of neurons in the lateral septum - bioRxiv](https://www.biorxiv.org/content/10.1101/2024.02.15.580381v1.full)

[Opens in a new window](https://www.biorxiv.org/content/10.1101/2024.02.15.580381v1.full)

<https://orbi.uliege.be/bitstream/2268/266807/1/Menon%2C%202021%20Neurobiology%20of%20the%20lateral%20septum%20regulation%20of%20social%20behavior.pdf" \t "_blank>

[orbi.uliege.be](https://orbi.uliege.be/bitstream/2268/266807/1/Menon%2C%202021%20Neurobiology%20of%20the%20lateral%20septum%20regulation%20of%20social%20behavior.pdf)

[Neurobiology of the lateral septum: regulation of social behavior - ORBi](https://orbi.uliege.be/bitstream/2268/266807/1/Menon%2C%202021%20Neurobiology%20of%20the%20lateral%20septum%20regulation%20of%20social%20behavior.pdf)

[Opens in a new window](https://orbi.uliege.be/bitstream/2268/266807/1/Menon%2C%202021%20Neurobiology%20of%20the%20lateral%20septum%20regulation%20of%20social%20behavior.pdf)

<https://www.frontiersin.org/journals/neuroscience/articles/10.3389/fnins.2021.748186/full" \t "_blank>

[frontiersin.org](https://www.frontiersin.org/journals/neuroscience/articles/10.3389/fnins.2021.748186/full)

[Ontogeny of the Projections From the Dorsomedial Division of the Anterior Bed Nucleus of the Stria Terminalis to Hypothalamic Nuclei - Frontiers](https://www.frontiersin.org/journals/neuroscience/articles/10.3389/fnins.2021.748186/full)

[Opens in a new window](https://www.frontiersin.org/journals/neuroscience/articles/10.3389/fnins.2021.748186/full)

[pmc.ncbi.nlm.nih.gov](https://pmc.ncbi.nlm.nih.gov/articles/PMC3637669/)

[Mechanisms in the Bed Nucleus of the Stria Terminalis Involved in Control of Autonomic and Neuroendocrine Functions: A Review - PMC](https://pmc.ncbi.nlm.nih.gov/articles/PMC3637669/)

[Opens in a new window](https://pmc.ncbi.nlm.nih.gov/articles/PMC3637669/)

<https://www.bisusa.com/en/products/b/nucleus-x-bav-andptvcatheters.html" \t "_blank>

[bisusa.com](https://www.bisusa.com/en/products/b/nucleus-x-bav-andptvcatheters.html)

[NuCLEUS-X™ BAV and PTV Catheters - B. Braun Interventional Systems](https://www.bisusa.com/en/products/b/nucleus-x-bav-andptvcatheters.html)

[Opens in a new window](https://www.bisusa.com/en/products/b/nucleus-x-bav-andptvcatheters.html)

<https://www.numedforchildren.com/product/nucleus-xtm" \t "_blank>

[numedforchildren.com](https://www.numedforchildren.com/product/nucleus-xtm)

[NuCLEUS-X™ | NuMED For Children](https://www.numedforchildren.com/product/nucleus-xtm)

[Opens in a new window](https://www.numedforchildren.com/product/nucleus-xtm)

Sources read but not used in the report

<https://journals.physiology.org/doi/10.1152/jn.2000.83.1.13" \t "_blank>

[journals.physiology.org](https://journals.physiology.org/doi/10.1152/jn.2000.83.1.13)

[Cerebellar Flocculus and Paraflocculus Purkinje Cell Activity During Circular Pursuit in Monkey | Journal of Neurophysiology | American Physiological Society](https://journals.physiology.org/doi/10.1152/jn.2000.83.1.13)

[Opens in a new window](https://journals.physiology.org/doi/10.1152/jn.2000.83.1.13)

[pmc.ncbi.nlm.nih.gov](https://pmc.ncbi.nlm.nih.gov/articles/PMC11870495/)

[Neural circuit mechanisms to transform cerebellar population dynamics for motor control in monkeys - PubMed Central](https://pmc.ncbi.nlm.nih.gov/articles/PMC11870495/)

[Opens in a new window](https://pmc.ncbi.nlm.nih.gov/articles/PMC11870495/)

<https://esmed.org/MRA/mra/article/view/740/584" \t "_blank>

[esmed.org](https://esmed.org/MRA/mra/article/view/740/584)

[The Thalamus: A Review of its Functional Anatomy](https://esmed.org/MRA/mra/article/view/740/584)

[Opens in a new window](https://esmed.org/MRA/mra/article/view/740/584)

<http://zebrafishucl.org/forebrain-regions/emminentia-thalami" \t "_blank>

[zebrafishucl.org](http://zebrafishucl.org/forebrain-regions/emminentia-thalami)

[eminentia thalami/ thalamic eminence - Zebrafish UCL](http://zebrafishucl.org/forebrain-regions/emminentia-thalami)

[Opens in a new window](http://zebrafishucl.org/forebrain-regions/emminentia-thalami)

<https://nba.uth.tmc.edu/neuroscience/m/s4/chapter06.html" \t "_blank>

[nba.uth.tmc.edu](https://nba.uth.tmc.edu/neuroscience/m/s4/chapter06.html)

[Limbic System: Amygdala (Section 4, Chapter 6) Neuroscience Online - Department of Neurobiology & Anatomy](https://nba.uth.tmc.edu/neuroscience/m/s4/chapter06.html)

[Opens in a new window](https://nba.uth.tmc.edu/neuroscience/m/s4/chapter06.html)

<https://nba.uth.tmc.edu/neuroanatomy/L8/Lab08p25_index.html" \t "_blank>

[nba.uth.tmc.edu](https://nba.uth.tmc.edu/neuroanatomy/L8/Lab08p25_index.html)

[Neuroanatomy Online: Lab 8 (ƒ7) - Visual System - The Visual/Ocular Motor Pathways - Midbrain - Department of Neurobiology & Anatomy](https://nba.uth.tmc.edu/neuroanatomy/L8/Lab08p25_index.html)

[Opens in a new window](https://nba.uth.tmc.edu/neuroanatomy/L8/Lab08p25_index.html)
