# Supplementary material for: Cooperation of transposable elements to endow global networks of initiators of hybrid assembly pathways of endogenous multiprotein complexes
Source: Front Cell Infect Microbiol. 2026 Jul 30;16:1793868. doi: 10.3389/fcimb.2026.1793868 (PMC13468005; doi:10.3389/fcimb.2026.1793868)
Supplement: Supplementary file 6 [file DataSheet6.zip › Table of top 10 significant 1106CLA.docx]

**1106 hCLA marker genes. Table of top 10 significant p-values and q-values for Allen Brain Atlas up**

| **term** | **p-value** | **q-value** | **Claustrum marker genes** |
| --- | --- | --- | --- |
| caudal secondary prosencephalon | 1.386140e-24 | 1.012806e-21 | [CHRM1, THRB, CPNE5, MTMR12, FHL2, DUSP14, PCLO, MFSD4, B3GALT2, FGFR1OP2, ENC1, DLGAP3, EPHA4, MEF2C, ACTN1, TBR1, RTN4RL2, DUSP6, CACNB1, MN1, ZEB2, GPD2, PITPNM2, RASA2, SIDT1, CDH12, DCX, KCNQ5, PLCB1, PDZRN3, NGEF, PDE1A, SATB2, GPR88, KCNA4, GPR83, PRICKLE1, NLK, ARPP19, NPAS2, DDN, EXTL1, NEUROD6, GNG4, CECR6, PLXNA2, GSG1L, RGS20, DRD1, DRD2, CSMD1, CACNG3, EGR1, EGR2, EGR3, BCL11B, ARPP21, KCNB1, KCNIP2, PLK2, SYT16, MBOAT2, TTC9B, GPR6, ARHGAP25, HS3ST2, PPFIBP1, RGS12, ZNF697] |
| secondary prosencephalon | 1.386140e-24 | 1.012806e-21 | [CHRM1, THRB, CPNE5, MTMR12, FHL2, PTPRJ, DUSP14, PCLO, MFSD4, B3GALT2, RASGEF1C, ENC1, DLGAP3, EPHA4, MEF2C, ACTN1, TBR1, RTN4RL2, DUSP6, CACNB1, MN1, GPD2, PITPNM2, RASA2, SIDT1, CDH12, DCX, KCNQ5, PLCB1, PDZRN3, NGEF, PDE1A, SATB2, GPR88, KCNA4, GPR83, PRICKLE1, NLK, ARPP19, NPAS2, DDN, EXTL1, NEUROD6, GNG4, CECR6, PLXNA2, GSG1L, RGS20, DRD1, DRD2, CSMD1, CACNG3, EGR1, EGR3, BCL11B, ARPP21, KCNB1, KCNIP2, PLK2, SYT16, MBOAT2, TTC9B, CCK, GPR6, ARHGAP25, HS3ST2, PPFIBP1, RGS12, ZNF697] |
| telencephalic vesicle | 1.386140e-24 | 1.012806e-21 | [CHRM1, THRB, CPNE5, MTMR12, FHL2, DUSP14, PCLO, MFSD4, B3GALT2, FGFR1OP2, ENC1, DLGAP1, DLGAP3, EPHA4, MEF2C, PRKCB, ACTN1, TBR1, RTN4RL2, DUSP6, CACNB1, MN1, ZEB2, GPD2, PITPNM2, RASA2, SIDT1, CDH12, DCX, KCNQ5, PLCB1, PDZRN3, NGEF, PDE1A, SATB2, GPR88, KCNA4, GPR83, PRICKLE1, NLK, ARPP19, NPAS2, EXTL1, NEUROD6, GNG4, CECR6, PLXNA2, GSG1L, RGS20, DRD1, DRD2, CSMD1, EGR1, EGR2, EGR3, BCL11B, ARPP21, KCNB1, KCNIP2, PLK2, SYT16, MBOAT2, TTC9B, GPR6, ARHGAP25, HS3ST2, PPFIBP1, VIP, ZNF697] |
| forebrain | 7.413610e-24 | 4.062658e-21 | [THRB, CPNE5, MTMR12, NCAN, FHL2, PTPRJ, ICAM5, DUSP14, PCLO, MFSD4, B3GALT2, RASGEF1C, ENC1, DLGAP3, EPHA4, MEF2C, TBR1, RTN4RL2, DUSP6, MN1, PITPNM2, RASA2, KCNQ3, SIDT1, CDH12, DCX, KCNQ5, PLCB1, PDZRN3, KCTD16, PDE1A, SATB2, GPR88, KCNA4, GPR83, PRICKLE1, NLK, ARPP19, NPAS2, DDN, EXTL1, ADAMTS19, NEUROD6, GNG4, CECR6, PLXNA2, GSG1L, RGS20, DRD1, DRD2, CSMD1, CACNG3, EGR1, EGR3, BCL11B, ARPP21, KCNB1, KCNIP2, PLK2, SYT16, MBOAT2, TTC9B, GPR6, ARHGAP25, HS3ST2, PPFIBP1, RGS12, ZNF697] |
| alar plate of evaginated telencephalic vesicle | 3.887540e-23 | 1.704298e-20 | [CHRM1, THRB, CPNE5, MTMR12, FHL2, DUSP14, PCLO, MFSD4, B3GALT2, FGFR1OP2, ENC1, DLGAP3, EPHA4, MEF2C, PRKCB, ACTN1, TBR1, RTN4RL2, DUSP6, CACNB1, MN1, ZEB2, GPD2, PITPNM2, RASA2, SIDT1, CDH12, DCX, KCNQ5, PLCB1, PDZRN3, NGEF, PDE1A, SATB2, GPR88, KCNA4, GPR83, PRICKLE1, NLK, ARPP19, NPAS2, EXTL1, NEUROD6, GNG4, CECR6, PLXNA2, GSG1L, RGS20, DRD1, DRD2, CSMD1, EGR1, EGR2, EGR3, BCL11B, ARPP21, KCNB1, KCNIP2, PLK2, SYT16, MBOAT2, TTC9B, GPR6, ARHGAP25, HS3ST2, PPFIBP1, ZNF697] |
| Cerebrum | 4.969481e-21 | 1.815517e-18 | [CHRM1, THRB, MTMR12, FHL2, DUSP14, PCLO, MFSD4, FGFR1OP2, ENC1, PMEPA1, DLGAP3, MEF2A, EPHA4, KCNH3, MEF2C, ACTN1, TBR1, RTN4RL2, DUSP6, DIEXF, CACNB1, MN1, PITPNM2, RASA2, SIDT1, CDH12, DCX, KCNQ5, PLCB1, PDZRN3, NGEF, SATB2, GPR88, KCNA4, GPR83, PRICKLE1, NLK, ARPP19, C17ORF96, EXTL1, NEUROD6, GNG4, CECR6, PLXNA2, GSG1L, RGS20, EPS8L1, DRD1, DRD2, EGR1, EGR2, EGR3, BCL11B, ARPP21, KCNB1, KCNIP2, SYT16, MBOAT2, TTC9B, GPR6, HS3ST2, PPFIBP1, VIP, ZNF697] |
| central subpallium (classic basal ganglia) | 1.138331e-19 | 2.772468e-17 | [CPNE5, SERPINE2, SHB, RGS4, NETO1, PMEPA1, DLGAP3, RGS8, RBFOX1, PRKCB, ACTN1, TESC, SYTL5, CACNB1, MN1, ARHGEF9, TYRO3, KCNQ5, PLCB1, NGEF, DGKI, DGKH, NECAB2, GPR88, ITPR1, KCNA4, KCNA5, GPR83, ARPP19, NPAS2, AKAP1, CHST11, GNG7, KIAA1324, SPOCK3, CHST15, PLXNA2, GSG1L, RGS20, SLC17A8, DRD1, DRD2, WNT2, EGR1, EGR3, EGR4, BCL11B, ARPP21, KCNIP2, PLK2, GABRA4, ST8SIA3, SORBS1, FBXL16, ATP2B1, GPR6, BAIAP2, PDP1, ID4, GNB4, LHX6, YPEL2] |
| mantle zone of Str | 1.138331e-19 | 2.772468e-17 | [CPNE5, SERPINE2, SHB, RGS4, KCNT1, NETO1, PMEPA1, DLGAP3, RGS8, DLGAP2, RBFOX1, PRKCB, ACTN1, TESC, SYTL5, CACNB1, MN1, ARHGEF9, KCNQ5, PLCB1, NGEF, DGKI, DGKH, NECAB2, GPR88, ITPR1, KCNA4, KCNA5, GPR83, ARPP19, NPAS2, AKAP1, CHST11, GNG7, KIAA1324, SPOCK3, CHST15, GSG1L, RGS20, SLC17A8, DRD1, DRD2, MYH10, WNT2, EGR1, EGR3, EGR4, BCL11B, ARPP21, KCNIP2, PLK2, GABRA4, ST8SIA3, SORBS1, FBXL16, ATP2B1, GPR6, BAIAP2, C7ORF62, PDP1, ID4, YPEL2] |
| striatum (corpus striatum) | 1.138331e-19 | 2.772468e-17 | [CPNE5, SERPINE2, SHB, RGS4, KCNT1, NETO1, PMEPA1, DLGAP3, RGS8, DLGAP2, RBFOX1, PRKCB, ACTN1, TESC, SYTL5, CACNB1, MN1, ARHGEF9, KCNQ5, PLCB1, NGEF, DGKI, DGKH, NECAB2, GPR88, ITPR1, KCNA4, KCNA5, GPR83, ARPP19, NPAS2, AKAP1, CHST11, GNG7, KIAA1324, SPOCK3, CHST15, GSG1L, RGS20, SLC17A8, DRD1, DRD2, MYH10, WNT2, EGR1, EGR3, EGR4, BCL11B, ARPP21, KCNIP2, PLK2, GABRA4, ST8SIA3, SORBS1, FBXL16, ATP2B1, GPR6, BAIAP2, C7ORF62, PDP1, ID4, YPEL2] |
| subpallium | 5.279912e-19 | 1.157357e-16 | [CPNE5, SERPINE2, SHB, RGS4, ALCAM, NETO1, PMEPA1, DLGAP3, RBFOX1, PRKCB, ACTN1, TESC, SYTL5, CACNB1, MN1, ARHGEF9, RAP2B, TYRO3, KCNQ5, PLCB1, NGEF, DGKI, DGKH, NECAB2, GPR88, ITPR1, NEDD4L, KCNA4, KCNA5, GPR83, ARPP19, NPAS2, AKAP1, CHST11, GNG7, KIAA1324, SPOCK3, CHST15, GSG1L, RGS20, SLC17A8, DRD1, DRD2, WNT2, EGR1, EGR3, EGR4, BCL11B, ARPP21, KCNIP2, PLK2, GABRA4, ST8SIA3, OPRK1, SORBS1, FBXL16, GPR6, BAIAP2, ID4, GNB4, LHX6] |
