# Supplementary material for: Cooperation of transposable elements to endow global networks of initiators of hybrid assembly pathways of endogenous multiprotein complexes
Source: Front Cell Infect Microbiol. 2026 Jul 30;16:1793868. doi: 10.3389/fcimb.2026.1793868 (PMC13468005; doi:10.3389/fcimb.2026.1793868)
Supplement: Supplementary file 6 [file DataSheet6.zip › 244HUBs from35iniTFs Table top10 SynGo.docx]

**239 hCLA marker genes. Table of top 10 significant p-values and q-values for SynGO 2024**

| **term** | **p-value** | **q-value** | **overlap_genes** |
| --- | --- | --- | --- |
| Postsynaptic Cytosol (GO:0099524) CC | 2.203115e-11 | 1.630305e-09 | [UBE2I, SUMO1, NEDD4, SUMO2, PRKCD, CAMK2A, HTT, PIN1, CALM3, CALM1, MTOR] |
| Postsynaptic Density (GO:0014069) CC | 1.379623e-10 | 5.104606e-09 | [RPL5, HSPA8, GSK3B, ARF1, CAMK2A, STAT3, ARRB1, ARRB2, PRKCZ, PAK1, CASP3, ABL1, MDM2, RPS3, MAPK1, VHL] |
| Modulation Of Chemical Synaptic Transmission (GO:0050804) BP | 2.352992e-10 | 5.804046e-09 | [ITGB1, UBE2I, STAT3, ARRB2, DNM1, GNAI2, MAPK9, MAPK8, HNRNPK, FYN, PLCG1, JAK2, MAPK3] |
| Presynaptic Cytosol (GO:0099523) CC | 7.477225e-10 | 1.383287e-08 | [UBE2I, SUMO1, PRKCB, PRKCE, SUMO2, CAMK2A, HTT, PRKCA, CALM3, CALM1] |
| Postsynapse (GO:0098794) CC | 3.736499e-09 | 5.530018e-08 | [HSPA8, PTK2, RHOA, PPP1CA, EEF1A1, CDC42, PAK1, HNRNPK, APC, CDK5, CDH1, TRAF6, SUMO2, FLNA, RAC1, PRKACA, JAK2, ARF6] |
| Regulation Of Neurotransmitter Receptor Localization To Postsynaptic Specialization Membrane (GO:0098696) BP | 9.577810e-08 | 1.181263e-06 | [GSK3B, PAK1, TRAF6, CAMK2A, TNIK, PRKCZ, GABARAP] |
| Presynapse (GO:0098793) CC | 2.536775e-06 | 2.657821e-05 | [HSPA8, MAP1LC3B, SUMO2, GRB2, TNIK, SLC2A4, PRKACA, YWHAG, YWHAH, ARF6, GNAI2, PPP1CA] |
| Synapse (GO:0045202) CC | 2.873320e-06 | 2.657821e-05 | [YWHAE, RPL5, RPL3, RELA, PPP2CA, MAP1LC3A, YWHAQ, PPP2R1A, RPL27A, RPS3, RPS27A, HNRNPA1, SQSTM1, YWHAG, YWHAH] |
| Postsynaptic Specialization, Intracellular Component (GO:0099091) CC | 6.668429e-06 | 5.482931e-05 | [LYN, LCK, SRC, GABARAP] |
| Regulation Protein Catabolic Process At Postsynapse (GO:0140252) BP | 2.654239e-05 | 1.964137e-04 | [NEDD4, TRAF6, PIN1, VHL] |
