# Supplementary material for: Cooperation of transposable elements to endow global networks of initiators of hybrid assembly pathways of endogenous multiprotein complexes
Source: Front Cell Infect Microbiol. 2026 Jul 30;16:1793868. doi: 10.3389/fcimb.2026.1793868 (PMC13468005; doi:10.3389/fcimb.2026.1793868)
Supplement: Supplementary file 6 [file DataSheet6.zip › Table of top 10 significant 35iniTFs.docx]

**35 hCLA initiator TFs. Table of top 10 significant p-values and q-values for Allen Brain Atlas up**

| **term** | **p-value** | **q-value** | **Initiator TF genes** |
| --- | --- | --- | --- |
| telencephalic vesicle | 3.965645e-08 | 0.000013 | [EGR1, EGR2, MEF2C, EGR3, THRB, BCL11B, TBR1, NPAS2] |
| Cerebrum | 3.965645e-08 | 0.000013 | [MEF2A, EGR1, EGR2, MEF2C, EGR3, THRB, BCL11B, TBR1] |
| alar plate of evaginated telencephalic vesicle | 3.965645e-08 | 0.000013 | [EGR1, EGR2, MEF2C, EGR3, THRB, BCL11B, TBR1, NPAS2] |
| caudal secondary prosencephalon | 3.965645e-08 | 0.000013 | [EGR1, EGR2, MEF2C, EGR3, THRB, BCL11B, TBR1, NPAS2] |
| Cerebral nuclei | 7.641636e-07 | 0.000089 | [MEF2A, EGR1, EGR3, EGR4, BCL11B, LHX6, NPAS2] |
| Isocortex | 7.641636e-07 | 0.000089 | [EGR1, EGR2, MEF2C, EGR3, THRB, CUX2, TBR1] |
| forebrain | 7.641636e-07 | 0.000089 | [EGR1, MEF2C, EGR3, THRB, BCL11B, TBR1, NPAS2] |
| mantle zone of OCx | 7.641636e-07 | 0.000089 | [EGR1, EGR2, MEF2C, EGR3, THRB, CUX2, TBR1] |
| occipital cortex | 7.641636e-07 | 0.000089 | [EGR1, EGR2, MEF2C, EGR3, THRB, CUX2, TBR1] |
| secondary prosencephalon | 7.641636e-07 | 0.000089 | [EGR1, MEF2C, EGR3, THRB, BCL11B, TBR1, NPAS2] |
